# Influence of Halogen Atoms and the Reactivity of Nucleophiles on Reactions of Tetrahydropyran and Tetrahydrofuran Acetals with *C*‑Nucleophiles: Hyperconjugation and Inductive Effects

**DOI:** 10.1021/acs.joc.4c03128

**Published:** 2025-07-03

**Authors:** Krystyna M. Demkiw, Wouter A. Remmerswaal, Asma Sheikh, Ibrahim N. Sheikh, Collin H. Witt, Jeroen D. C. Codée, K. A. Woerpel

**Affiliations:** a Department of Chemistry, 5894 New York University, 100 Washington Square East, New York, New York 10003, United States; b Leiden Institute of Chemistry, 4496 Leiden University, Einsteinweg 55, Leiden 2300 RA, The Netherlands

## Abstract

Tetrahydropyran acetals bearing a fluorine atom adjacent to the acetal carbon atom can undergo highly stereoselective substitution reactions with nucleophilic alkenes to give the 1,2-*cis* products. By contrast, the chlorine- and bromine-substituted acetals give the 1,2-*trans* products. These results can be understood by considering oxocarbenium ion intermediates and their conformational preferences, which are dictated by hyperconjugative effects from axial substituents, with F ≪ H < Cl < Br. Reactions of the corresponding five-membered-ring acetals are also 1,2-*cis* selective in the case of fluorine and 1,2-*trans* selective with chlorine- and bromine-substituted acetals, but selectivities showed different trends of reactivity vs selectivity. The reactions with the five-membered-ring acetal were interpreted as requiring anomeric halides as reactive intermediates because of the conditions required to obtain substitution products.

## Graphical abstract

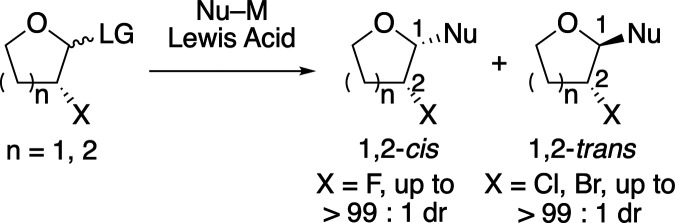

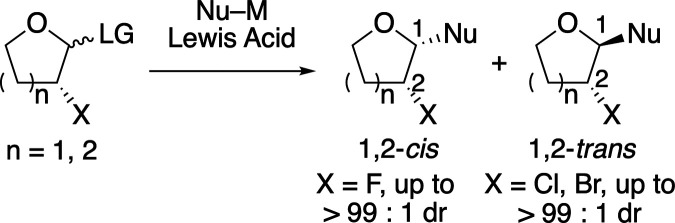

## Introduction

Fluorinated compounds have become important components of medicinal chemistry because of the unique properties that the fluorine atom confers to a molecule.^1^ For example, fluorinated sugars play important roles in developing various drugs and diagnostic tools.^2–5^ The presence of a fluorine atom in sugar systems,^4^ particularly at C-2, can dramatically change the reactivity of sugars. The presence of a fluorine atom slows the rate of hydrolysis of acetals (for example, eq 1 ^6^),^7^ likely because a fluorine atom can destabilize the developing positive charge in the transition state for hydrolysis.^8^ This property allows for the development of inhibitors of glycosidases^9^ and the development of molecules to examine the binding of carbohydrates.^10^ The role that an individual fluorine atom has on the reactivity and stereoselectivity of glycosylation reactions involving fluorosugars, however, can be difficult to deduce considering that its influence cannot be separated from the influences of other substituents.^8,11–13^



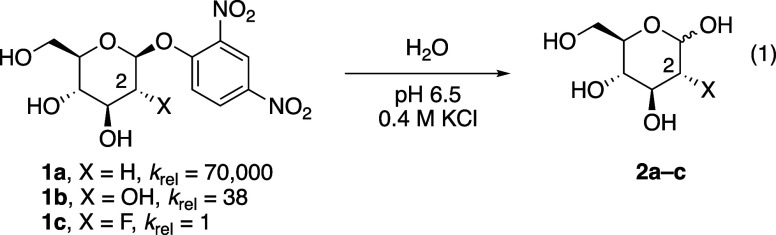

1



In this article, we examine how the presence of a single fluorine atom at C-2 of a tetrahydropyran or tetrahydrofuran acetal can influence the stereoselectivity of acetal substitution reactions. These stereoselectivities are compared to those observed for acetals with either a chlorine or a bromine atom. Earlier studies with alcohols as nucleophiles, which are relatively reactive, indicated that a single halogen atom on an acetal can control stereoselectivity, although stereoselectivity was only uniformly high in the case of bromine-substituted acetals.^14^ The trends observed in those experiments suggested that reactions with weaker nucleophiles would lead to higher stereoselectivities, even with fluorine-substituted acetals. To test this idea, we examined reactions of acetals with π-nucleophiles such as allylic silanes. The reactivity of these compounds is sensitive to their substitution pattern, which allowed us to use relatively weak, sterically small nucleophiles to establish the influence that reactivity imparts on stereoselectivity.^15,16^ These trends would provide evidence that the oxocarbenium ions involved in these reactions exhibit strong conformational preferences and therefore will react with high stereoselectivities.^14^

## Results and Discussion

The acetals required to determine the ability of halogen atoms to control the stereoselectivities of acetal substitution reactions were synthesized as shown in Scheme 1. The anomeric acetates and benzoates, as racemic mixtures, were formed from the corresponding enol ethers by halogenation in the presence of the appropriate carboxylic acid.^17,18^ Although the acetoxy group could be installed onto fluorine-substituted tetrahydropyran acetal **3**,^19^ this compound was resistant to further reactions. Attempts to force these reactions led to complex reaction mixtures that could not be analyzed, so acetal **3** was not examined further. These problems could be solved using the trichloroacetimidate **6** derived from fluorine-substituted tetrahydropyran **5**, which could be formed cleanly^13^ and handled without purification. This derivative could not be prepared for the corresponding fluorine-substituted tetrahydrofuran, however, presumably because of the general higher sensitivity of five-membered ring acetals to hydrolysis.^14,20^ The anomeric benzoate **10** was chosen instead because this compound possessed a high enough molecular weight to permit isolation, purification, and characterization. The chlorine- and bromine-containing acetates **7**, **8**, **11**, and **12** could be handled and used readily, considering their higher molecular weight and their more rapid activation under the reaction conditions.^14^

**Scheme 1:**
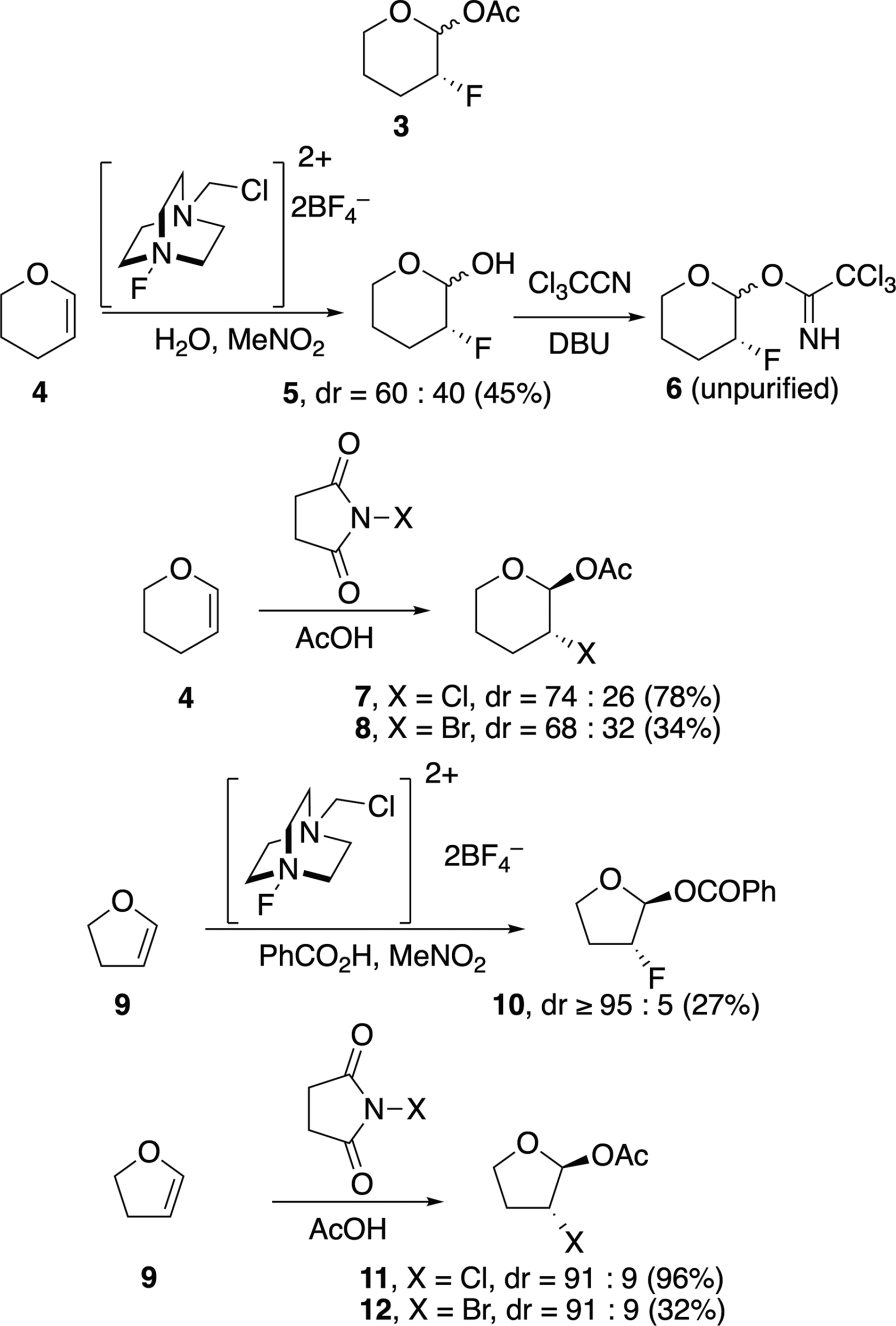
Synthesis of Substrates

Stereoselective substitution reactions of six-membered-ring trichloroacetimidate **6** demonstrated that the presence of one fluorine atom near the center undergoing substitution can lead to highly stereoselective reactions (eq 2, Table 1). Several of these reactions proceeded with low yield, likely because of the general difficulty of performing substitution reactions with a powerful electron-withdrawing fluorine atom near the electrophilic center.^6^ In addition, the products were volatile, so although stereoselectivities were reproducible, yields varied from experiment to experiment. The Lewis acid BF_3_·OEt_2_ was chosen because it is generally useful for activation of trichloroacetimidate electrophiles^21^ and the reactions involving this Lewis acid are less likely to proceed through covalent intermediates such as those that might form using Me_3_SiOTf. Although anomeric fluorides can be formed from the trichloroacetimidate donors,^22^ these intermediates are not necessarily involved in the stereochemistry-determining step. Diastereomeric mixtures of anomeric fluoride compounds can react with π-nucleophiles using BF_3_·OEt_2_, giving products with high stereoselectivity,^23^ suggesting that oxocarbenium ion intermediates are more likely involved. We have shown that the stereoselectivities of related reactions can be explained as involving oxocarbenium ion intermediates.^14^ The solvent and nucleophiles chosen for these experiments are unlikely to lead to S_N_2-like substitution reactions.^24–26^

**Table 1:**
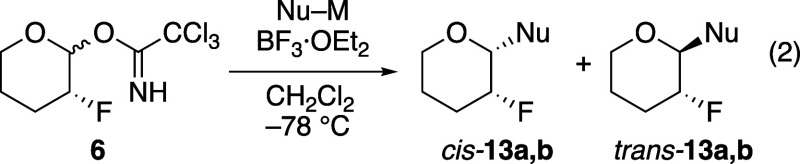

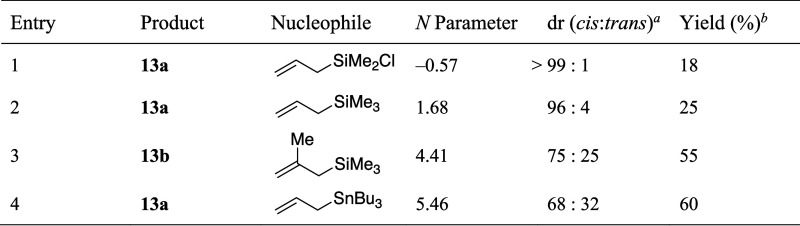
Substitutions of α-Fluorinated Tetrahydropyran Acetal **6** (eq 2)

a All diastereomeric ratios were determined by ^13^C­{^1^H} NMR spectroscopy^27^ and confirmed by ^1^H NMR spectroscopy.

b Isolated yield.

The diastereoselectivities of the reactions shown in eq 2 depended upon the reactivity of the nucleophile, as measured by the *N* parameter developed by Mayr and co-workers.^16^ With the weakest nucleophiles, the 1,2-*cis* product was strongly favored (entries 1 and 2). As nucleophilicity increased, however, the preference for the 1,2-*cis* product decreased (entries 3 and 4). This trend mirrors the trend observed with substitutions with alcohol nucleophiles, although, in that case, the 1,2-*trans* products were favored with low selectivity.^14^

Reactions of related chlorine- and bromine-substituted acetals **7** and **8** occurred with opposite stereoselectivity compared to reactions of their fluorine-containing counterpart (eqs 3 and 4, Table 2 and Table 3). In these cases, the tetrahydropyran acetate was sufficiently reactive to permit ionization, reflecting the less electron-withdrawing nature of chlorine and bromine atoms compared to fluorine atoms.^14^ As opposed to the preference for the 1,2-*cis* product with the fluorine-substituted acetal **6** (Table 1), the 1,2-*trans* product was favored when the substituent was chlorine or bromine (Table 2 and Table 3). The 1,2-*trans* stereoselectivity decreased as nucleophilicity increased, with the selectivity of the chlorine-substituted acetal **7** particularly sensitive to nucleophilicity. As anticipated, stereoselectivities were low using the strong nucleophile Me_3_SiCN, likely because reactions of the oxocarbenium ion with the nucleophile occurred at rates near the diffusion limit.^28^ In the case of reactions with allyldimethylchlorosilane, the hemiacetals **16** and **17** were also isolated, which reflects the low reactivity of these nucleophiles.

**Table 2:**
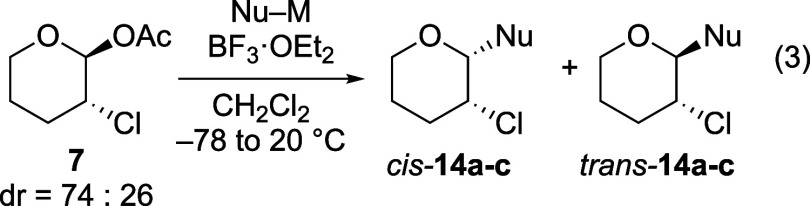

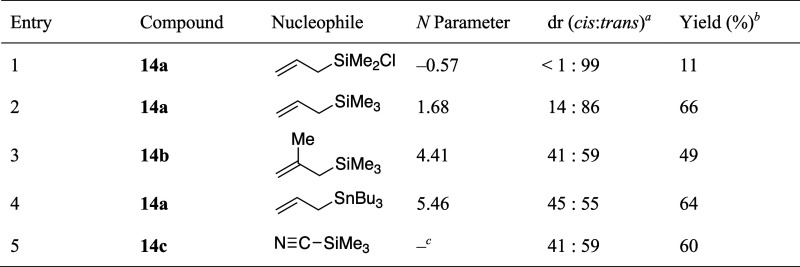
Substitutions of α-Chlorinated Tetrahydropyran Acetal **7** (eq 3)

a All diastereomeric ratios were determined by ^13^C­{^1^H} NMR spectroscopy^27^ and confirmed by ^1^H NMR spectroscopy.

b Isolated yield.

c *N* Parameter not reported.

**Table 3:**
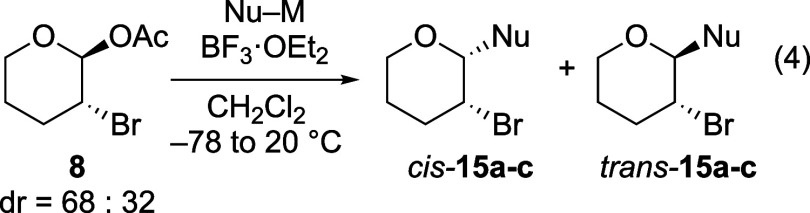

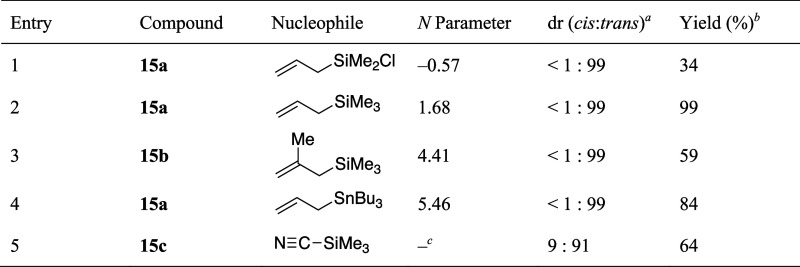
Substitutions of α-Brominated Tetrahydropyran Acetal **8** (eq 4)

a All diastereomeric ratios were determined by ^13^C­{^1^H} NMR spectroscopy^27^ and confirmed by ^1^H NMR spectroscopy.

b Isolated yield.

c *N* Parameter not reported.

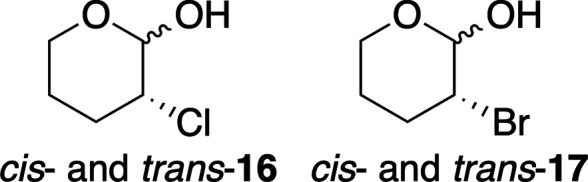



The stereoselectivities of reactions of the different halogen-substituted acetals are consistent with the conformational preferences of the oxocarbenium ions that are likely to be reactive intermediates in these reactions (Scheme 2). In the case of the fluorine-substituted tetrahydropyran oxocarbenium ion, the equatorial conformer **18** (X = F) is favored (as determined by computations using the coupled-cluster method) because hyperconjugation from the stronger electron donor, σ_C–H_, to π*_C=O+_ is maximized in this conformer.^14^ For the other halogen-substituted systems (X = Cl or Br), the oxocarbenium ion form is favored, not the corresponding three-membered-ring halonium ion.^14^ The axial conformer of the oxocarbenium ion (**19**) is favored because σ_C–X_ is a better donor by hyperconjugation than σ_C–H_ would be.^14^ Nucleophilic attack along the favored trajectory (that is, to form the product in a chair conformation, not a twist conformation^29,30^ ) would form the 1,2-*cis* product in the case of the fluorine-substituted oxocarbenium ion **18**, and the 1,2-*trans* product with the bromine-substituted one, **19** (X = Br). The carbon–carbon bond-forming step is stereochemistry-determining, and the additions are irreversible.^31^ The intermediate behavior of the chlorine-substituted oxocarbenium ion **19** (X = Cl) can be attributed to the lower preference for this group to adopt an axial orientation compared to the preference for the bromine atom. It is also likely that steric destabilization developing upon nucleophilic attack may lead to reactions through the somewhat higher energy equatorial conformer, in accordance with the Curtin–Hammett principle.^32,33^ The trend of decreasing selectivity as the reactivity of the nucleophile increases is consistent with addition to the oxocarbenium ion approaching the diffusion rate limit: as the rate of bond formation increases, at some point bond formation between the nucleophile and the electrophile becomes faster than separation of the components, leading to statistical mixtures of products.^25,26,28^ The divergent diastereoselectivity of these simple model systems is consistent with observations of fluorine-substituted sialic acid derivatives (1,2-*cis* selectivity) compared to their bromine-substituted analogues (1,2-*trans* selectivity),^34^ indicating the important role a single halogen atom can exert on stereoselectivity. The 1,2-*cis* diastereoselectivity with the fluorine-substituted acetal is not, however, consistent with the diastereoselectivity observed for intramolecular oxyfluorination reactions catalyzed by chiral phase-transfer catalysts.^35^

**Scheme 2:**
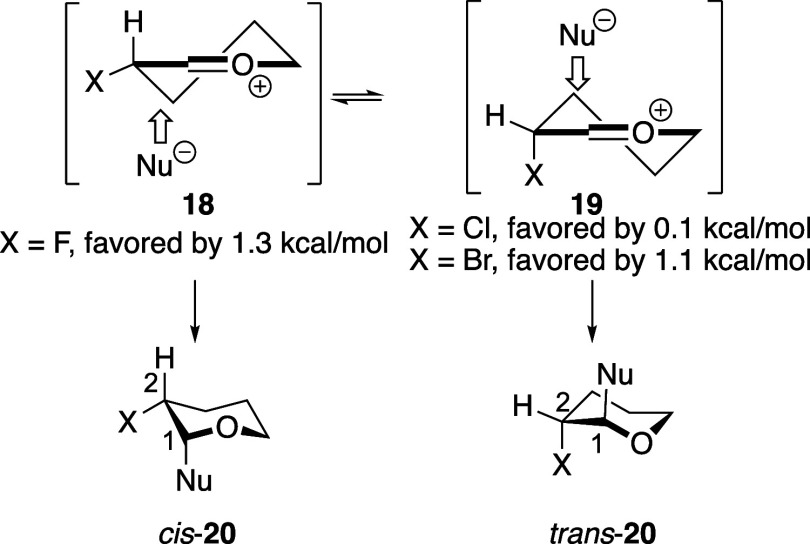
Origin of Stereoselectivity for Reactions of Pyran Acetals

The trend of stereoselectivities observed for reactions of the fluorine-substituted five-membered-ring acetal suggested that other explanations must be considered. In contrast to what had been observed in the fluorine-substituted tetrahydropyran acetal **6**, the selectivity with the corresponding tetrahydrofuran acetal **10** was higher with the more reactive nucleophile (eq 5, Table 4). While caution should be taken regarding results from reactions that proceed in low yield, the selectivities are reproducible experiment to experiment.

**Table 4:**
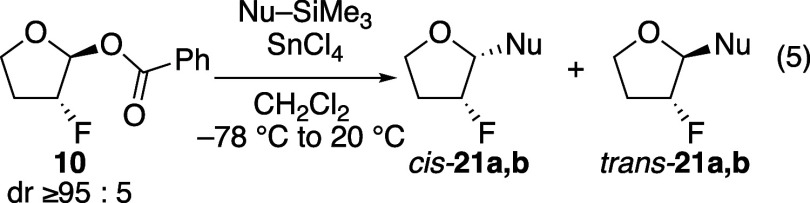

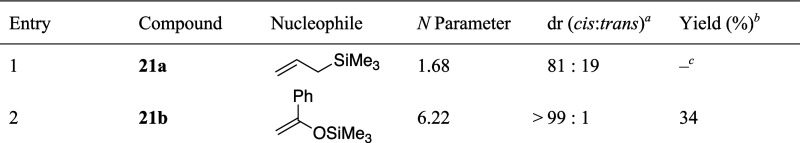
Substitutions of α-Fluorinated Tetrahydrofuran Acetal **10** (eq 5)

a All diastereomeric ratios were determined by ^13^C­{^1^H} NMR spectroscopy^27^ and confirmed by ^1^H and ^19^F­{^1^H} NMR spectroscopy unless otherwise noted.

b Isolated yield.

c These products were volatile, so all characterization was performed on materials that contained solvents as impurities.

Obtaining the results shown in Table 4 were experimentally challenging, however, which might have caused this different trend (vide infra). The use of the benzoate **10**, which was necessary to enable isolation of the substrate, limited the range of substitution reactions that could be examined. Ionization of benzoate **10** required using SnCl_4_ as the Lewis acid because the use of other Lewis acids (BF_3_·OEt_2_, Me_3_SiOTf, and TiCl_4_) led to recovery of starting material. The reaction also did not occur at –78 °C, so the reaction mixtures needed to warm to room temperature to see any conversion. The use of such a strong Lewis acid also limited the range of nucleophiles that could be used. An experiment using methallyltrimethylsilane gave considerable amounts of decomposition products along with small quantities of what might have been substitution products. The use of the highly nucleophilic allylic silane trimethyl­(2-phenyallyl)­silane (H_2_C=CHPhCH_2_SiMe_3_)^36^ gave only decomposition products. The requirement for using SnCl_4_ also precluded the use of allyltributylstannane, considering that this reagent is known to undergo rapid transmetalation with SnCl_4_.^37^

Efforts to use a different leaving group on the furan were unsuccessful. The volatile hemiacetal **22** (eq 6), which was prepared similarly to the tetrahydropyran hemiacetal **5** (Scheme 1), could not be converted cleanly to the trichloroacetimidate derivative, but it could be converted to the analogous trifluoroacetimidate^38^ **23**, from which a single stereoisomer could be isolated. This substrate, however, did not react cleanly under any substitution conditions. Only using SnCl_4_ as the Lewis acid could even a small amount of substitution product **21b** be observed, and the same major stereoisomer of product was formed. Too little product was obtained to determine a diastereomer ratio. Efforts to vary solvent and temperature and to use other nucleophiles did not lead to clean reactions.

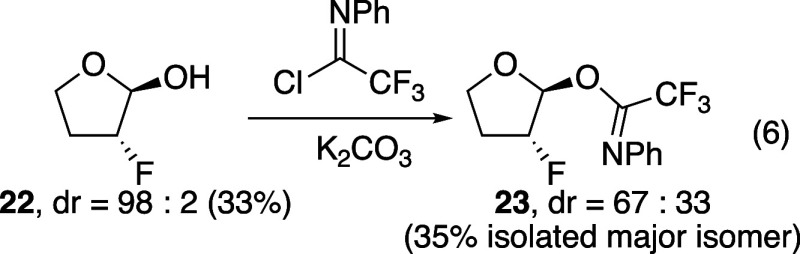

6



Determining the selectivities of substitutions reported in Table 4 was challenging considering the volatility of some products. The allylated products **21a** were volatile, so solutions of these materials could not be concentrated without losing all the product. As a result, these compounds were characterized as mixtures along with the solvents dichloromethane and methanol, which had been used in the isolation and handling of products. An isolable and purifiable product, ketone **21b**, could be formed using a silyl enol ether as the nucleophile. This product was crystalline, and X-ray crystallography of a single crystal of ketone **21b** allowed for assignment of its relative configuration.^39^ Comparing the spectroscopic properties of ketone **21b** (in particular, the similar coupling constants between protons) to those obtained from the alkene **21a** allowed for the assignment of stereochemistry of alkene **21a**.

The stereoselectivities observed using the fluorine-substituted benzoate **10** suggest that an oxocarbenium ion might not be the reactive intermediate in reactions in the nonpolar solvent CH_2_Cl_2_. A fluorine atom at C-2, as illustrated in eq 1, exerts strong inductive effects that destabilize oxocarbenium ion intermediates, although intermediate **18** has been observed by NMR spectroscopy in superacid medium.^19^ The instability of the oxocarbenium ion would lead to a greater role for contact ion pairs, as observed for other acetal substitution reactions.^40^

An experiment using a more polar solvent provided some insight into the origin of stereoselectivity of these acetals. Using a more charge-stabilizing solvent like acetonitrile (which may^41^ or may not^42^ be coordinating) led to the same 1,2-*cis* product, *cis-* **21b**, although the selectivity was lower (eq 7). The lower selectivity in this case is consistent with generating an oxocarbenium ion in this more ionizing medium, which suggests that the stereoselectivity of the reaction in CH_2_Cl_2_ (Table 4, entry 2) could involve a covalent intermediate. The intermediate could be an anomeric chloride generated from the acetal and the metal chloride,^43–45^ and that product could ionize in the more polar solvent. Alternatively, the reaction in acetonitrile could involve an intermediate involving the nitrile solvent,^41,42^ although it is less clear why that mechanism would result in the same stereoisomer of product. Regardless of the reason, these results differ only in the magnitude of the stereoselectivity, not the major product formed.

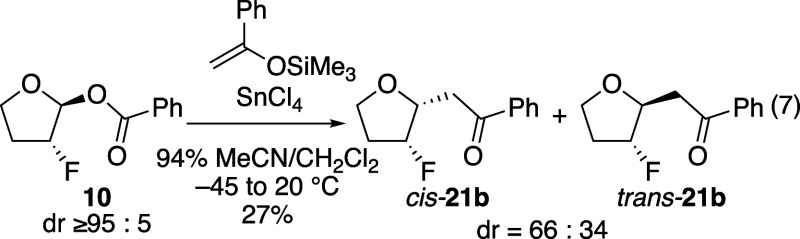

7



The stereoselectivities of reactions involving the chlorine- and bromine-substituted tetrahydrofuran acetals **11** and **12**, respectively (eqs 8 and 9, Table 5 and Table 6), follow the trends observed with the chlorine- and bromine-substituted tetrahydropyran acetals (Table 2 and Table 3). As the reactivity of the nucleophile increased, diastereoselectivity decreased. This trend was more dramatic with the chlorine-substituted acetal **11**, just as observed in the six-membered-ring series (Table 2). Just as with the pyran substrate (eq 3, Table 2), hemiacetal *trans-* **26** was formed as a side-product in reactions using the weakest nucleophile.

**Table 5:**
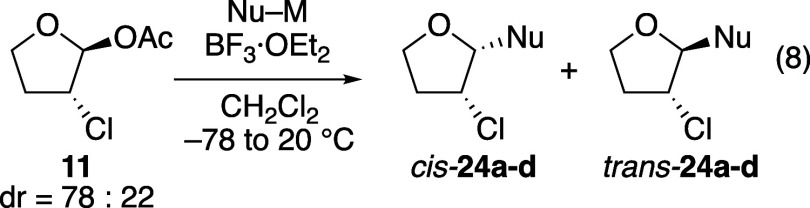

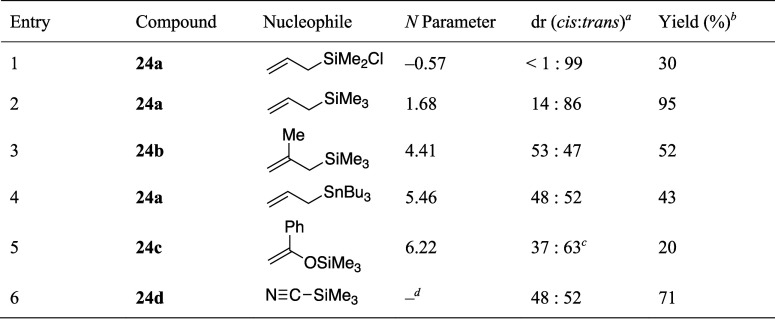
Substitutions of α-Chlorinated Tetrahydrofuran Acetal **11** (eq 8)

a All diastereomeric ratios were determined by ^13^C­{^1^H} NMR spectroscopy^27^ and confirmed by ^1^H NMR spectroscopy.

b Isolated yield.

c SnCl_4_ was used as the Lewis acid.

d *N* Parameter not reported.

**Table 6:**
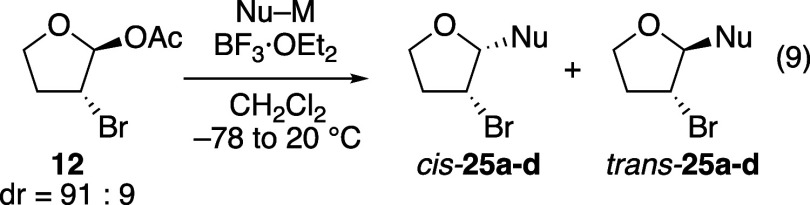

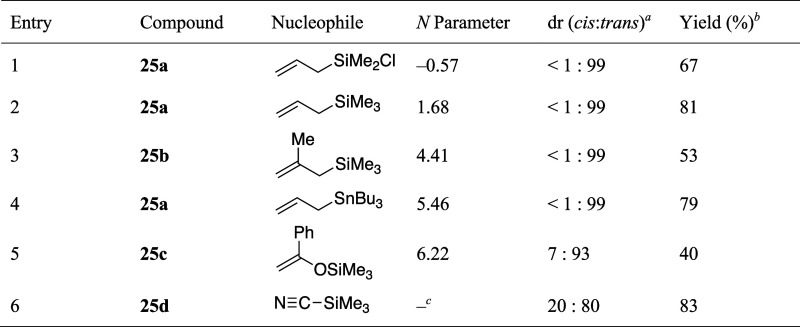
Substitutions of α-Brominated Tetrahydrofuran Acetal **12** (eq 9)

a All diastereomeric ratios were determined by ^13^C­{^1^H} NMR spectroscopy^27^ and confirmed by ^1^H NMR spectroscopy.

b Isolated yield.

c *N* Parameter not reported.

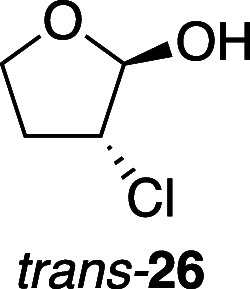



The trend of diastereoselectivity of nucleophilic substitution reactions with monosubstituted α-halogenated tetrahydrofu-rans can be explained by consideration of conformational preferences and stereoelectronic effects of reactions involving oxocarbenium ions (Scheme 3). Just as with the tetrahydropyran acetals, computational studies revealed that the fluorine-substituted oxocarbenium ion adopted the equatorial conformer **27** because of the poor donor ability of σ_C–F_ compared to σ_C–H_.^46^ By contrast, the oxocarbenium ions with chlorine and bromine atoms preferred to adopt axial conformers **28** to maximize hyperconjugative stabilization of the oxocarbenium ion.^14^ The diastereoselectivity can be rationalized by nucleophilic addition to the stereoelectronically favored inside face^47,48^ of the favored intermediates.

**Scheme 3:**
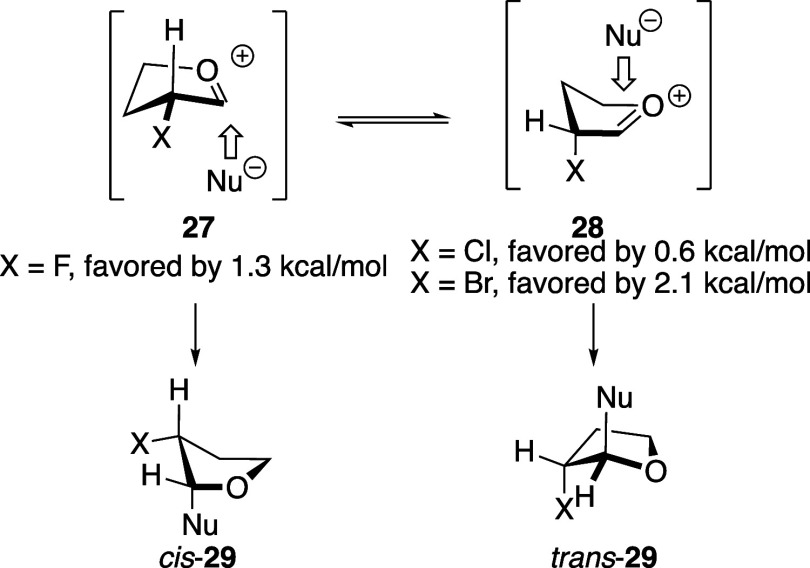
Origin of Stereoselectivity for Reactions of Furan Acetals

The reason for higher 1,2-*cis* selectivity in the case of the fluorine-substituted tetrahydrofuran acetal **10** with stronger nucleophiles (Table 4) is not immediately obvious. Had the reactions proceeded via contact ion pairs, the 1,2-*trans* product would have been favored, considering that the counterion generally occupies the face reserved for the nucleophile.^26,40^ In the case of weaker nucleophiles such as allyltrimethylsilane,^31^ it is unlikely that such an intermediate is involved,^26,40^ and the selectivity is consistent with reaction through an oxocarbenium ion.

With stronger nucleophiles, however, the higher selectivity observed may be a consequence of the constraints encountered on performing these reactions. The only substrate that reacted cleanly was the anomeric benzoate **10**, but this compound could only be activated with SnCl_4_. We hypothesized that the use of this Lewis acid in a nonpolar solvent could give rise to an anomeric chloride,^43–45^ considering that the fluorine atom would destabilize any oxocarbenium ion intermediates by destabilizing inductive effects.^3,6^ The anomeric chloride could ionize in the more polar solvent (eq 7), leading to reactions with outcomes consistent with oxocarbenium ions as reactive intermediates (Scheme 3).

Low temperature NMR studies provided evidence that an anomeric chloride was a reasonable reactive intermediate. Treatment of benzoate **10**, whose stereochemistry had been established by X-ray crystallography, with SnCl_4_ at low temperature gave a new product, assigned as *trans*-**30** (eq 10). The anomeric proton, H^a^, moved downfield from δ 6.55 ppm for benzoate **10** to δ 7.11 ppm for *trans*-**30**, which is consistent with the introduction of the more electron-withdrawing chlorine atom.^49–51^ The ^3^ *J*_HH_ coupling constant between H^a^ and H^b^ was ∼ 0 Hz in both compounds, which is consistent with H^a^ and H^b^ adopting equatorial positions in both compounds,^46,49,50^ with the benzoyloxy group of **10** and the chlorine atom of *trans*-**30** adopting an axial orientation to maximize anomeric stabilization.^46,52^ The ^3^ *J*_HF_ coupling constant of *trans*-**30** was similar to that observed for the *trans*-substituted benzoate **10** (8.8 Hz compared to 9.2 Hz, respectively), although this coupling constant is less diagnostic of stereochemistry for tetrahydrofurans than it is for tetrahydropyrans.^50,53^ The new compound was not formed cleanly, however, and attempts to isolate it were unsuccessful because it decomposed upon warming, which is consistent with the sensitivity observed for other simple cyclic chloroacetals.^54–56^



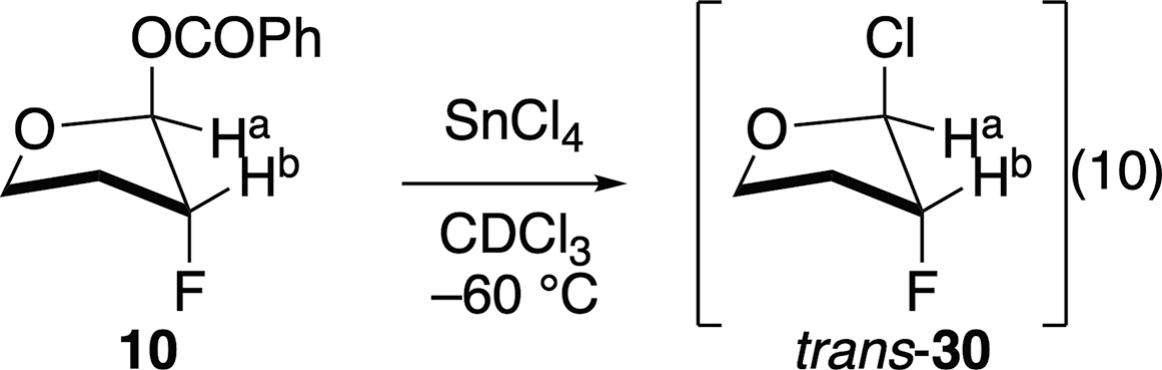

10



The hypothesis that anomeric chloride *trans-* **30** was involved in substitution reactions in less polar solvents would explain the increased *cis*-selectivity with stronger nucleophiles. This selectivity is consistent with reactions of strong nucleophiles with simple fluorine-substituted bromoacetals resembling **10**, which also occurred with high 1,2-*cis* selectivity.^57^ Based upon those studies, we propose that the anomeric chloride *trans-* **30** (Scheme 4) reacted through an oxocarbenium-ion-like S_N_2-like transition state resembling **31** to give *cis*-**32**. With weaker nucleophiles or in more ionizing solvents (eq 7), the anomeric chloride *trans*-**30** is in equilibrium with the corresponding oxocarbenium ion **27**, which would react with lower 1,2-*cis* selectivity (Scheme 3).

**Scheme 4:**
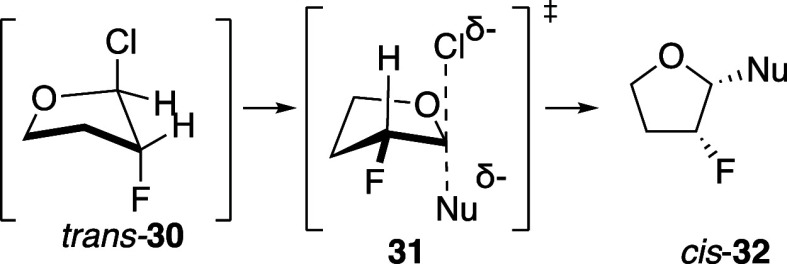
Reaction of Anomeric Chloride

## Conclusion

These results demonstrate that the presence of a single fluorine atom can exert a high degree of stereochemical control on substitution reactions. In the case of the six-membered-ring acetal, reactions with the weakest nucleophiles occurred to give the 1,2-*cis* product with ≥ 96 : 4 diastereoselectivity. These results are consistent with nucleophilic additions to the equatorially substituted oxocarbenium ion along the stereoelectronically preferred trajectory. The stereoselectivities are generally opposite to what are seen in the case of chlorine- and bromine-substituted acetals, which gave the 1,2-*trans* products with diastereoselectivities ranging from 86 : 14 to > 99 : 1 . In all cases, as the nucleophilicity increased, the diastereoselectivity decreased, which is consistent with attack of a strong nucleophile to the oxocarbenium ion being faster than these two components can dissociate from each other. In the case of a fluorine-substituted five-membered-ring acetal, the trend of reactivity and selectivity was opposite under the conditions examined: selectivity increased with increasing nucleophilicity. The opposite result was observed for the chlorine- and bromine-substituted acetals. The results with the fluorine-substituted acetal suggests that the presence of a fluorine atom strongly destabilized an oxocarbenium ion, so products were formed by direct displacement reactions of covalent intermediates. These observations suggest caution when analyzing the stereoselectivities of reactions involving fluorine-substituted acetals because the important reactive intermediates may be species such as anomeric chlorides, not oxocarbenium ions.

## Experimental Section

### General Methods

^1^H NMR and ^13^C­{^1^H} NMR spectra were obtained at room temperature using Bruker AVIII-400 (400 and 100 MHz, respectively), AVIIIHD-400 (400 and 100 MHz, respectively), AVNEO-500 (500 and 125 MHz, respectively), and AV-600 (600 and 150 MHz, respectively) spectrometers. ^19^F­{^1^H} NMR spectra were obtained at room temperature using an AVIII-400 (377 MHz) spectrometer. All spectroscopic data are reported as follows: chemical shifts are reported in ppm on the δ scale, ^1^H and ^13^C­{^1^H} NMR spectra are internally referenced to tetramethylsilane (^1^H NMR: CDCl_3_ δ 0.00; ^13^C­{^1^H} NMR: CDCl_3_ δ 0.00), ^19^F­{^1^H} NMR spectra are externally referenced to hexafluorobenzene (^19^F­{^1^H} NMR: CDCl_3_ δ – 164.9), multiplicity (br = broad, s = singlet, d = doublet, t = triplet, q = quartet, m = multiplet), coupling constants (Hz), and integration. Ratios of products were derived from one-pulse ^1^H NMR, ^13^C­{^1^H} NMR, or ^19^F­{^1^H} integrations using diagnostic peaks in the unpurified reaction mixture.^27^ Multiplicities of carbon peaks were defined using HSQC experiments. Infrared (IR) spectra were recorded using a Thermo Nicolet AVATAR Fourier Transform IR spectrometer using attenuated total reflectance (ATR). High-resolution mass spectra were acquired on an Agilent 6224 Accurate-Mass time-of-flight spectrometer and were obtained using peak matching. The ionization sources used were either atmospheric pressure chemical ionization (APCI) or electrospray ionization (ESI), as indicated. Liquid chromatography was performed using forced flow (flash chromatography) of the indicated solvent system on silica gel (SiO_2_) 60 (230–400 mesh). Dichloromethane and acetonitrile were dried and degassed using a solvent purification system before use. All dry reactions were run under a nitrogen atmosphere in glassware that had been flame-dried under reduced pressure. Unless otherwise noted, all reagents and substrates were commercially available. Hemiacetal **5** and acetals **7**, **8**, **9**, **12** were prepared using known methods.^14^ Compounds **13a**, **14a**, **15a**, **16**, **17**, **21a**, **22a**, and **26** are known in the literature.^14^

### Synthesis of Substrates

#### (2R*,3R*)-3-Fluorotetrahydrofuran-2-yl Benzoate (cis-**10**) and (2R*,3S*)-3-fluorotetrahydrofuran-2-yl Benzoate (trans-**10**)

A reported procedure^14^ was adapted to prepare benzoate **10**. To a cooled (0 °C) stirred suspension of benzoic acid (9.34 g, 76.5 mmol) in 2,3-dihydrofuran (2.0 mL, 27 mmol) and CH_3_NO_2_ (66.8 mL) was added Selectfluor (14.19 g, 40.05 mmol). After 1 h, the reaction mixture was warmed to 20 °C and stirred for an additional 18 h. The reaction mixture was then heated to reflux (110 °C) for 2 h, and then concentrated under vacuum to remove acetonitrile. Saturated aqueous NaHCO_3_ (100 mL) was then added, the layers were separated, and the aqueous layer was extracted with CH_2_Cl_2_ (3 × 150 mL). The combined organic layers were dried over Na_2_SO_4_, filtered, and concentrated *in vacuo*. ^13^C­{^1^H} NMR and ^19^F­{^1^H} NMR spectroscopic analysis of the unpurified reaction mixture revealed that benzoate **10** was formed as a 6:94 mixture of diastereomers (*cis-* **10**:*trans-* **10**). Purification by flash chromatography (10:90 EtOAc:hexanes) afforded a mixture of benzoate *cis-* **10** and benzoate *trans-* **10** as a white solid (1.50 g, 27%) with a diastereomeric ratio of 5 : ≥ 95, as confirmed by ^1^H NMR and ^19^F­{^1^H} NMR spectroscopic analysis. This mixture was used for characterization. X-ray quality crystals were grown by slow evaporation of a solution of benzoate **10** in MeOH. The relative stereochemical configuration of benzoate **10** was assigned by X-ray crystallographic analysis: mp = 45–50 °C; IR (ATR) 2900, 1716, 1599, 1454 cm^–1^; HRMS (ESI) *m/z:* [M + Na]^+^ Calcd for C_11_H_11_FO_3_Na 233.0587; Found 233.0584. *Major Diastereomer trans*-**10**: ^1^H NMR (500 MHz, CDCl_3_) δ 8.00–7.99 (m, 2H), 7.61–7.56 (m, 1H), 7.47–7.43 (m 2H), 6.55 (d, *J* = 9.3, 1H), 5.24 (br ddd, *J* = 51.6, 4.5, 0.6, 1H), 4.31–4.22 (m, 2H), 2.49–2.23 (m, 2H); ^13^C­{^1^H} NMR (125 MHz, CDCl_3_) δ 165.1 (C), 133.6 (CH), 129.9 (CH), 129.6 (CH), 128.6 (CH), 100.3–100.0 (br d, ^2^ *J*_C–F_ = 34.5, CH), 95.6–94.2 (br d, ^1^ *J*_C–F_ = 179.7, CH), 68.4 (CH_2_), 30.1–29.9 (br d, ^2^ *J*_C–F_ = 20.9, CH_2_); ^19^F­{^1^H} NMR (377 MHz, CDCl_3_) δ – 185.9 (s). *Minor Diastereomer cis*-**10**:^13^C­{^1^H} NMR (100 MHz, CDCl_3_, diagnostic peaks) δ 101.2–100.9 (br d, ^2^ *J*_C–F_ = 33.2, CH), 96.3–94.5 (br d, ^1^ *J*_C–F_ = 177.5, CH); ^19^F­{^1^H} NMR (377 MHz, CDCl_3_) δ – 185.4 (s).

### Nucleophilic Additions to Tetrahydropyran and Tetrahydrofuran Acetals

#### (2R*,3R*)-2-Allyl-3-fluorotetrahydro-2H-pyran (cis-**13a**)

To a cooled (0 °C) solution of hemiacetal **5** (0.162 g, 1.35 mmol) in CH_2_Cl_2_ (12 mL) were added trichloroacetonitrile (1.25 mL, 12.5 mmol) and DBU (20 μL, 0.13 mmol). After 5 min, the mixture was warmed to 20 °C and stirred for an additional 1 h. The reaction mixture was then concentrated *in vacuo* and trichloroacetimidate **6** was directly used without further purification. To a cooled (−78 °C) solution of trichloroacetimidate **6** (1.35 mmol) and allyldimethylchlorosilane (760 μL, 5.03 mmol) in CH_2_Cl_2_ (12 mL) was added BF_3_·OEt_2_ (315 μL, 2.51 mmol) dropwise over 2 min. After 1 h, Et_3_N (300 μL) was added, and the reaction mixture was concentrated *in vacuo*. ^1^H NMR, ^13^C­{^1^H} NMR, and ^19^F­{^1^H} NMR spectroscopic analysis of the unpurified reaction mixture revealed that alkene *cis*-**13a** was formed as single diastereomer (dr >99:1). Purification by flash chromatography (5:95 Et_2_O:pentanes) afforded the major diastereomer *cis*-**13a** as a colorless oil (0.035 g, 18%). The spectroscopic data (^1^H NMR, ^13^C­{^1^H} NMR, ^19^F­{^1^H} NMR, HRMS, IR) for alkene *cis*-**13a** are consistent with the data reported in the literature.^14^

#### (2R*,3R*)-2-Allyl-3-fluorotetrahydro-2H-pyran (cis-**13a**) and (2R*,3S*)-2-allyl-3-fluorotetrahydro-2H-pyran (trans-**13a**)

To a cooled (0 °C) solution of hemiacetal **5** (0.131 g, 1.09 mmol) in CH_2_Cl_2_ (18 mL) were added trichloroacetonitrile (1.1 mL, 11 mmol) and DBU (20 μL, 0.13 mmol). After 5 min, the mixture was warmed to 20 °C and stirred for an additional 2 h. The reaction mixture was then concentrated *in vacuo* and trichloroacetimidate **6** was directly used without further purification. To a cooled (−78 °C) solution of trichloroacetimidate **6** (1.09 mmol) and allyltrimethylsilane (690 μL, 4.34 mmol) in CH_2_Cl_2_ (9 mL) was added BF_3_·OEt_2_ (270 μL, 2.15 mmol) dropwise over 2 min. After 1 h, Et_3_N (100 μL) was added, and the reaction mixture was concentrated in vacuo. ^1^H NMR and ^13^C­{^1^H} NMR spectroscopic analysis of the unpurified reaction mixture revealed that alkene **13a** was formed as a 96:4 mixture of diastereomers (*cis*-**13a**:*trans*-**13a**). Purification by flash chromatography (5:95 Et_2_O:pentane) afforded the major diastereomer *cis*-**13a** as a colorless oil (0.039 g, 25%). The spectroscopic data (^1^H NMR, ^13^C­{^1^H} NMR, ^19^F­{^1^H} NMR, IR, HRMS) for alkenes *cis*-**13a** and *trans*-**13a** are consistent with the data reported in the literature.^14^ *Major Diastereomer cis*-**13a** *:* ^1^H NMR (400 MHz, CDCl_3_) δ 5.88–5.78 (m, 1H), 5.18–5.09 (m, 2H), 4.50 (dtd, *J* = 47.8, 2.7, 0.7 1H), 4.05–4.01 (m, 1H), 3.51–3.45 (m, 1H), 3.35 (dt, *J* = 29.2, 7.1, 1H), 2.48–2.32 (m, 2H), 2.21–2.14 (m, 1H), 2.05–1.93 (m, 1H), 1.76–1.56 (m, 1H), 1.45–1.39 (m, 1H); ^13^C­{^1^H} NMR (100 MHz, CDCl_3_) δ 134.1 (CH), 117.8 (CH_2_), 87.8–86.0 (br d, ^1^ *J*_C–F_ = 177.3, CH), 78.0–77.8 (br d, ^2^ *J*_C–F_ = 18.8, CH), 68.2 (CH_2_), 36.0 (br d, ^3^ *J*_C–F_ = 5.0, CH_2_), 28.7–28.4 (br d, ^2^ *J*_C–F_ = 22.0, CH_2_), 20.4 (CH_2_); ^19^F­{^1^H} NMR (377 MHz, CDCl_3_) δ – 191.4 (s); IR (ATR) 2949, 1643, 1093, 1052, 912, 889 cm^–1^; HRMS (APCI) *m/z:* [(M + H) – HF]^+^ Calcd for C_8_H_13_O 125.0961; Found 125.0966. *Minor Diastereomer trans*-**13a** *:* ^13^C­{^1^H} NMR (100 MHz, CDCl_3_, diagnostic peaks) δ 134.3, 36.2.

#### (2R*,3R*)-3-Fluoro-2-(2-methylallyl)­tetrahydro-2H-pyran (cis-**13b**) and (2R*,3S*)-3-fluoro-2-(2-methylallyl)­tetrahydro-2H-pyran (trans-**13b**)

To a cooled (0 °C) solution of hemiacetal **5** (0.156 g, 1.30 mmol) in CH_2_Cl_2_ (12 mL) were added trichloroacetonitrile (1.25 mL, 12.5 mmol) and DBU (20 μL, 0.13 mmol). After 5 min, the mixture was warmed to 20 °C and stirred for an additional 1 h. The reaction mixture was then concentrated *in vacuo* and trichloroacetimidate **6** was directly used without further purification. To a cooled (−78 °C) solution of trichloroacetimidate **6** (1.30 mmol) and 2-methallyltrimethylsilane (880 μL, 5.01 mmol) in CH_2_Cl_2_ (12 mL) was added BF_3_·OEt_2_ (315 μL, 2.51 mmol) dropwise over 2 min. After 1 h, Et_3_N (300 μL) was added, and the reaction mixture was concentrated *in vacuo*. ^1^H NMR and ^13^C­{^1^H} NMR spectroscopic analysis of the unpurified reaction mixture revealed that alkene **13b** was formed as a 75:25 mixture of diastereomers (*cis*-**13b**:*trans*-**13b**). Purification by flash chromatography (5:95 Et_2_O:pentanes) afforded the major diastereomer *cis*-**13b** as a colorless oil (0.076 g, 37%) and the minor diastereomer *trans*-**13b** as a colorless oil (0.037 g, 18%). The relative stereochemical configurations of the two compounds were assigned by ^1^H NMR coupling constants. *Major Diastereomer cis*-**13b** *:* ^1^H NMR (400 MHz, CDCl_3_) δ 4.83 (d, *J* = 16.1, 2H), 4.50 (dt, *J* = 47.5, 2.6, 1H), 4.05–4.01 (m, 1H), 3.52–3.41 (m, 2H), 2.42–2.37 (m, 1H), 2.33–2.28 (m, 1H), 2.23–2.13 (m, 1H), 2.05–1.93 (m, 1H), 1.77 (s, 3H), 1.74–1.57 (m, 1H), 1.45–1.40 (m, 1H); ^13^C­{^1^H} NMR (100 MHz, CDCl_3_) δ 141.6 (C), 113.1 (CH_2_), 87.0 (br d, ^1^ *J*_C–F_ = 177.1, CH), 76.4 (br d, ^2^ *J*_C–F_ = 18.6, CH), 68.0 (CH_2_), 39.7 (br d, ^3^ *J*_C–F_ = 4.6, CH_2_), 28.5 (br d, ^2^ *J*_C–F_ = 22.1, CH_2_), 22.7 (CH_3_), 20.3 (CH_2_); ^19^F­{^1^H} NMR (377 MHz, CDCl_3_) δ – 203.8 (s); IR (ATR) 2951, 1651, 1124, 1093, 986, 890 cm^–1^; HRMS (ESI) *m/z:* [M + H]^+^ Calcd for C_9_H_16_FO 159.1180; Found 159.1177. *Minor Diastereomer trans*-**13b** *:* ^1^H NMR (400 MHz, CDCl_3_) δ 4.85–4.79 (m, 2H), 4.16 (dddd, *J* = 48.8, 10.1, 8.9, 5.1, 1H), 3.93–3.87 (m, 1H), 3.42–3.29 (m, 2H), 2.57–2.52 (m, 1H), 2.27–2.15 (m, 2H), 1.78 (s, 3H), 1.76–1.59 (m, 3H); ^13^C­{^1^H} NMR (100 MHz, CDCl_3_) δ 142.3 (C), 112.5 (CH_2_), 90.3 (br d, ^1^ *J*_C–F_ = 177.3, CH), 78.2 (br d, ^2^ *J*_C–F_ = 22.8, CH), 67.4 (CH_2_), 40.1 (CH_2_), 30.0 (br d, ^2^ *J*_C–F_ = 18.7, CH_2_), 25.1 (br d, ^3^ *J*_C–F_ = 9.5, CH_2_), 22.7 (CH_3_); ^19^F­{^1^H} NMR (377 MHz, CDCl_3_) δ – 183.6 (s).

#### (2R*,3R*)-2-Allyl-3-fluorotetrahydro-2H-pyran (cis-**13a**) and (2R*,3S*)-2-allyl-3-fluorotetrahydro-2H-pyran (trans-**13a**)

A reported procedure^14^ was adapted to prepare trichloroacetimidate **6**. To a cooled (0 °C) solution of hemiacetal **5** (0.156 g, 1.30 mmol) in CH_2_Cl_2_ (12 mL) were added trichloroacetonitrile (1.25 mL, 12.5 mmol) and DBU (20 μL, 0.13 mmol). After 5 min, the mixture was warmed to 20 °C and stirred for an additional 1 h. The reaction mixture was then concentrated *in vacuo* and trichloroacetimidate **6** was directly used without further purification. To a cooled (−78 °C) solution of trichloroacetimidate **6** (1.30 mmol) and allyltributylstannane (1.550 mL, 5.00 mmol) in CH_2_Cl_2_ (12 mL) was added BF_3_·OEt_2_ (320 μL, 2.55 mmol) dropwise over 2 min. After 1 h, Et_3_N (300 μL) was added, and the reaction mixture was concentrated *in vacuo*. ^1^H NMR, ^13^C­{^1^H} NMR, and ^19^F­{^1^H} NMR spectroscopic analysis of the unpurified reaction mixture revealed that alkene **13a** was formed as an 68:32 mixture of diastereomers (*cis*-**13a**:*trans*-**13a**). Purification by flash chromatography (5:95 Et_2_O:pentanes) afforded the major diastereomer *cis*-**13a** as a colorless oil (0.125 g, 60%). The spectroscopic data (^1^H NMR, ^13^C­{^1^H} NMR, ^19^F­{^1^H} NMR, IR, HRMS) for alkenes *cis*-**13a** and *trans*-**13a** are consistent with the data reported in the literature.^14^

#### (2R*,3S*)-2-Allyl-3-chlorotetrahydro-2H-pyran (trans-**14a**), (2R*,3S*)-3-chlorotetrahydro-2H-pyran-2-ol (cis-16), and (2R*,3R*)-3-chlorotetrahydro-2H-pyran-2-ol (trans-**16**)

To a cooled (−78 °C) solution of acetal **7** (0.182 g, 1.07 mmol) and allylchlorodimethylsilane (500 μL, 3.31 mmol) in CH_2_Cl_2_ (10 mL) was added BF_3_·OEt_2_ (260 μL, 2.07 mmol) dropwise over 2 min. After 1 h, the mixture was warmed to 20 °C and stirred for an additional 2 h. Saturated aqueous NaHCO_3_ (5 mL) was then added, the layers were separated, and the aqueous layer was extracted with CH_2_Cl_2_ (3 × 10 mL). The combined organic layers were washed with brine (1 × 15 mL), dried over Na_2_SO_4_, filtered, and concentrated *in vacuo*. ^1^H NMR and ^13^C­{^1^H} NMR spectroscopic analysis of the unpurified reaction mixture revealed that alkene *trans*-**14a** was formed as a single diastereomer (dr >99:1; 11% yield). Analysis of the ^1^H NMR and ^13^C­{^1^H} NMR spectra of the unpurified reaction mixture also revealed the formation of hemiacetal **16** as a 54:46 mixture of diastereomers (*cis*-**16**:*trans*-**16**; 42% yield). Analysis of the ^1^H NMR and ^13^C­{^1^H} NMR spectra of the unpurified reaction mixture also revealed the formation of an uncharacterized compound as a 76:24 mixture of diastereomers (47% conversion). Purification by flash chromatography (2:98 EtOAc:hexanes) afforded alkene *trans*-**14a** as a light yellow oil (0.019 g, 11%). The relative stereochemical configurations of the compound was assigned by ^1^H NMR coupling constants. The spectroscopic data (^1^H NMR, ^13^C NMR, HRMS, IR) for alkene *trans*-**14a** are consistent with the data reported in the literature.^14^ The spectroscopic data (^1^H NMR, ^13^C­{^1^H} NMR, HRMS) for hemiacetal **16** are consistent with the data reported in literature.^17^

#### (2R*,3R*)-2-Allyl-3-chlorotetrahydro-2H-pyran (cis-**14a**) and (2R*,3S*)-2-allyl-3-chlorotetrahydro-2H-pyran (trans-**14a**)

To a cooled (−78 °C) solution of acetal **7** (0.182 g, 1.07 mmol) and allyltrimethylsilane (655 μL, 4.12 mmol) in CH_2_Cl_2_ (6 mL) was added BF_3_·OEt_2_ (260 μL, 2.07 mmol) dropwise over 2 min. After 1 h, the mixture was warmed to 20 °C and stirred for an additional 2 h. Saturated aqueous NaHCO_3_ (5 mL) was then added, the layers were separated, and the aqueous layer was extracted with CH_2_Cl_2_ (3 × 10 mL). The combined organic layers were washed with brine (1 × 15 mL), dried over Na_2_SO_4_, filtered, and concentrated *in vacuo*. ^1^H NMR and ^13^C­{^1^H} NMR spectroscopic analysis of the unpurified reaction mixture revealed that alkene **14a** was formed as a 14:86 mixture of diastereomers (*cis*-**14a**:*trans*-**14a**). Purification by flash chromatography (2:98 EtOAc:hexanes) afforded the major diastereomer *trans*-**14a** as a light yellow oil (0.114 g, 66%). The spectroscopic data (^1^H NMR, ^13^C NMR, HRMS, IR) for alkenes *trans*-**14a** and *cis*-**14a** are consistent with the data reported in the literature.^14^ *Major Diastereomer trans-* **14a** *:* ^1^H NMR (400 MHz, CDCl_3_) δ 5.93–5.82 (m, 1H), 5.18–5.08 (m, 2H), 4.00–3.93 (m, 1H), 3.64 (ddd, *J* = 10.8, 10.1, 4.7, 1H), 3.45–3.38 (m, 1H), 3.32 (ddd, *J* = 10.1, 7.6, 2.9, 1H), 2.72–2.64 (m, 1H), 2.37–2.27 (m, 2H), 1.84–1.64 (m, 3H); ^13^C­{^1^H} NMR (100 MHz, CDCl_3_) δ 134.1 (CH), 117.4 (CH_2_), 81.9 (CH), 68.1 (CH_2_), 58.5 (CH), 36.9 (CH_2_), 34.8 (CH_2_), 27.2 (CH_2_); IR (ATR) 3077, 2851, 1115, 1091, 913, 764 cm^–1^; HRMS (ESI) *m/z:* [(M + H) – HCl]^+^ Calcd for C_8_H_13_O 125.0961; Found 125.0963. *Minor Diastereomer cis-* **14a** *:* ^1^H NMR (400 MHz, CDCl_3_, diagnostic peaks) δ 5.82–5.72 (m, 1H), 4.06–4.02 (m, 1H); ^13^C­{^1^H} NMR (100 MHz, CDCl_3_) δ 133.6 (CH), 118.0 (CH_2_), 78.6 (CH), 68.6 (CH_2_), 59.3 (CH), 38.0 (CH_2_), 32.0 (CH_2_), 22.0 (CH_2_).

#### (2R*,3R*)-3-Chloro-2-(2-methylallyl)­tetrahydro-2H-pyran (cis-**14b**) and (2R*,3S*)-3-chloro-2-(2-methylallyl)­tetrahydro-2H-pyran (trans-**14b**)

To a cooled (−78 °C) solution of acetal **7** (0.177 g, 1.03 mmol) and methallyltrimethylsilane (725 μL, 4.13 mmol) in CH_2_Cl_2_ (6 mL) was added BF_3_·OEt_2_ (260 μL, 2.07 mmol) dropwise over 2 min. After 1 h, the mixture was warmed to 20 °C and stirred for an additional 2 h. Saturated aqueous NaHCO_3_ (5 mL) was then added, the layers were separated, and the aqueous layer was extracted with CH_2_Cl_2_ (3 × 10 mL). The combined organic layers were washed with brine (1 × 15 mL), dried over Na_2_SO_4_, filtered, and concentrated *in vacuo*. ^1^H NMR and ^13^C­{^1^H} NMR spectroscopic analysis of the unpurified reaction mixture revealed that alkene **14b** was formed as a 41:59 mixture of diastereomers (*cis*-**14b**:*trans*-**14b**). Purification by flash chromatography (2:98 EtOAc:hexanes) afforded the major diastereomer *trans*-**14b** as a light yellow oil (0.047 g, 26%) and the minor diastereomer *cis*-**14b** as a light yellow oil (0.042 g, 23%). The relative stereochemical configurations of the two compounds were assigned by ^1^H NMR coupling constants. *Major Diastereomer trans*-**14b** *:* ^1^H NMR (400 MHz, CDCl_3_) δ 4.82 (d, *J* = 19.7, 2H), 4.00–3.94 (m, 1H), 3.66–3.58 (ddd, *J* = 10.9, 9.8, 4.6, 1H), 3.45–3.36 (m, 2H), 2.71 (d, *J* = 14.7, 1H), 2.38–2.32 (m, 1H), 2.18 (dd, *J* = 14.7, 9.3, 1H), 1.86–1.65 (m, 6H); ^13^C­{^1^H} NMR (100 MHz, CDCl_3_) δ 142.4 (C), 112.6 (CH_2_), 80.9 (CH), 68.1 (CH_2_), 59.2 (CH), 41.0 (CH_2_), 34.9 (CH_2_), 27.2 (CH_2_), 22.7 (CH_3_); IR (ATR) 2945, 1124, 1089, 942, 888, 762 cm^–1^; HRMS (APCI) *m/z:* [(M + H) – HCl]^+^ Calcd for C_9_H_15_O 139.1117; Found 139.1115. Anal. Calcd for C_9_H_15_ClO: C, 61.89; H, 8.66. Found: C, 61.81; H, 8.69. *Minor Diastereomer cis*-**14b** *:* ^1^H NMR (400 MHz, CDCl_3_) δ 4.86–4.82 (m, 2H), 4.11–4.02 (m, 2H), 3.60 (td, *J* = 6.7, 1.2 Hz, 1H), 3.49 (td, *J* = 11.8, 2.4 Hz, 1H), 2.37 (dd, *J* = 14.2, 7.0 Hz, 1H), 2.28 (dd, *J* = 14.2, 6.4 Hz, 1H), 2.22–2.09 (m, 2H), 2.05–1.94 (m, 2H), 1.76 (s, 3H), 1.45–1.36 (m, 1H); ^13^C­{^1^H} NMR (100 MHz, CDCl_3_) δ 141.2 (C), 113.3 (CH_2_), 77.3 (CH), 68.7 (CH_2_), 59.7 (CH), 41.7 (CH_2_), 32.2 (CH_2_), 22.9 (CH_3_), 20.0 (CH_2_).

#### (2R*,3R*)-2-Allyl-3-chlorotetrahydro-2H-pyran (cis-**14a**) and (2R*,3S*)-2-allyl-3-chlorotetrahydro-2H-pyran (trans-**14a**)

To a cooled (−78 °C) solution of acetal **7** (0.211 g, 1.18 mmol) and allyltributylstannane (1.400 mL, 4.52 mmol) in CH_2_Cl_2_ (11 mL) was added BF_3_·OEt_2_ (280 μL, 2.23 mmol) dropwise over 2 min. After 1 h, the mixture was warmed to 20 °C and stirred for an additional 2 h. Saturated aqueous NaHCO_3_ (10 mL) was then added, the layers were separated, and the aqueous layer was extracted with CH_2_Cl_2_ (3 × 10 mL). The combined organic layers were washed with brine (1 × 15 mL), dried over Na_2_SO_4_, filtered, and concentrated *in vacuo*. ^1^H NMR and ^13^C­{^1^H} NMR spectroscopic analysis of the unpurified reaction mixture revealed that alkene **14a** was formed as a 45:55 mixture of diastereomers (*cis*-**14a**:*trans*-**14a**). Purification by flash chromatography (5:95 EtOAc:hexanes) afforded alkenes *cis*-**14a** and *trans*-**14a** as a colorless oil (0.122 g, 64%) with a diastereomeric ratio of 43:57. The spectroscopic data (^1^H NMR, ^13^C­{^1^H} NMR, HRMS, IR) for alkenes *cis*-**14a** and *trans*-**14a** are consistent with the data reported in the literature.^14^

#### (2R*,3R*)-3-Chlorotetrahydro-2H-pyran-2-carbonitrile (cis-**14c**) and (2R*,3S*)-3-chlorotetrahydro-2H-pyran-2-carbonitrile (trans-**14c**)

To a cooled (−78 °C) solution of acetal **7** (0.110 g, 0.616 mmol) and trimethylsilyl cyanide (250 μL, 2.00 mmol) in CH_2_Cl_2_ (6 mL) was added BF_3_·OEt_2_ (150 μL, 1.19 mmol) dropwise over 2 min. After 1 h, the mixture was warmed to 20 °C and stirred for an additional 2 h. Saturated aqueous NaHCO_3_ (5 mL) was then added, the layers were separated, and the aqueous layer was extracted with CH_2_Cl_2_ (3 × 10 mL). The combined organic layers were washed with brine (1 × 15 mL), dried over Na_2_SO_4_, filtered, and concentrated *in vacuo*. ^1^H NMR and ^13^C­{^1^H} NMR spectroscopic analysis of the unpurified reaction mixture revealed that nitrile **14c** was formed as a 41:59 mixture of diastereomers (*cis*-**14c**:*trans*-**14c**). Purification by flash chromatography (20:80 EtOAc:hexanes) afforded nitriles *cis*-**14c** and *trans*-**14c** as a colorless oil (0.054 g, 60%) with a diastereomeric ratio of 47:53. This mixture was used for characterization: IR (ATR) 2869, 1199, 1083, 941, 887, 760 cm^–1^. Repeated attempts to obtain mass spectrometry data by LC–MS (electrospray ionization) and GC–MS (electron ionization) failed to give enough of the expected ions to permit identification of the sample’s molecular weight. *Major Diastereomer trans*-**14c** *:* ^1^H NMR (400 MHz, CDCl_3_, diagnostic peaks) δ 4.36 (d, *J* = 7.0, 1H), 3.89–3.85 (m, 2H), 3.68–3.62 (m, 1H), 2.43–2.38 (m, 1H); ^13^C­{^1^H} NMR (100 MHz, CDCl_3_) δ 115.7 (C), 70.8 (CH), 67.1 (CH_2_), 54.6 (CH_2_), 31.2 (CH), 23.2 (CH_2_). *Minor Diastereomer cis-* **14c** *:* ^1^H NMR (400 MHz, CDCl_3_, diagnostic peaks) δ 4.85 (dd, *J* = 4.8, 1.3, 1H), 2.30–2.25 (m, 1H), 2.12–2.01 (m, 1H); ^13^C­{^1^H} NMR (100 MHz, CDCl_3_) δ 114.6 (C), 70.4 (CH), 64.3 (CH_2_), 53.0 (CH_2_), 30.2 (CH), 25.2 (CH_2_).

#### (2R*,3S*)-2-Allyl-3-bromotetrahydro-2H-pyran (trans-**15a**), (2R*,3S*)-3-bromotetrahydro-2H-pyran-2-ol (cis-**17**), and (2R*,3R*)-3-bromotetrahydro-2H-pyran-2-ol (trans-**17**)

To a cooled (−78 °C) solution of acetal **8** (0.099 g, 0.44 mmol) and allyldimethylchlorosilane (275 μL, 1.82 mmol) in CH_2_Cl_2_ (5 mL) was added BF_3_·OEt_2_ (115 μL, 0.916 mmol) dropwise over 2 min. After 1 h, the mixture was warmed to 20 °C and stirred for an additional 2 h. Saturated aqueous NaHCO_3_ (5 mL) was then added, the layers were separated, and the aqueous layer was extracted with CH_2_Cl_2_ (3 × 10 mL). The combined organic layers were washed with brine (1 × 15 mL), dried over Na_2_SO_4_, filtered, and concentrated *in vacuo*. ^1^H NMR and ^13^C­{^1^H} NMR spectroscopic analysis of the unpurified reaction mixture revealed that alkene *trans*-**15a** was formed as a single diastereomer (dr >99:1; 76% yield). Analysis of the ^1^H NMR and ^13^C­{^1^H} NMR spectra of the unpurified reaction mixture also revealed the formation of hemiacetal **17** as a 42:58 mixture of diastereomers (*cis*-**17**:*trans*-**17**; 24% conversion). Purification by flash chromatography (5:95 EtOAc:hexanes) afforded alkene *trans*-**15a** as a colorless oil (0.069 g, 34%). The spectroscopic data (^1^H NMR, ^13^C­{^1^H} NMR, HRMS, IR) for alkene *trans*-**15a** is consistent with the data reported in the literature.^14^ The spectroscopic data (^1^H NMR, ^13^C­{^1^H} NMR, HRMS, IR) for hemiacetal **17** are consistent with the data reported in the literature.^17^

#### (2R*,3S*)-2-Allyl-3-bromotetrahydro-2H-pyran (trans-**15a**)

To a cooled (−78 °C) solution of acetal **8** (0.313 g, 1.40 mmol) and allyltrimethylsilane (860 μL, 5.41 mmol) in CH_2_Cl_2_ (14 mL) was added BF_3_·OEt_2_ (340 μL, 2.71 mmol) dropwise over 2 min. After 1 h, the mixture was warmed to 20 °C and stirred for an additional 2 h. Saturated aqueous NaHCO_3_ (15 mL) was then added, the layers were separated, and the aqueous layer was extracted with CH_2_Cl_2_ (3 × 20 mL). The combined organic layers were washed with brine (1 × 20 mL), dried over Na_2_SO_4_, filtered, and concentrated *in vacuo*. ^1^H NMR and ^13^C­{^1^H} NMR spectroscopic analysis of the unpurified reaction mixture revealed that alkene *trans*-**15a** was formed as a single diastereomer (dr >99:1). Purification by flash chromatography (2:98 EtOAc:hexanes) afforded alkene *trans*-**15a** as a light yellow oil (0.285 g, 99%). The spectroscopic data (^1^H NMR, ^13^C­{^1^H} NMR, HRMS, IR) for alkene *trans*-**15a** are consistent with the data reported in the literature:^14 1^H NMR (400 MHz, CDCl_3_) δ 5.91–5.81 (m, 1H), 5.17–5.10 (m, 2H), 4.03–3.99 (m, 1H), 3.80 (ddd, *J* = 11.8, 10.0, 4.5, 1H), 3.49–3.42 (m, 2H), 2.74–2.69 (m, 1H), 2.47–2.31 (m, 2H), 2.03–1.93 (m, 1H), 1.82–1.70 (m, 1H), 1.65–1.61 (m, 1H); ^13^C­{^1^H} NMR (100 MHz, CDCl_3_) δ 134.0 (CH), 117.5 (CH_2_), 81.7 (CH), 68.3 (CH_2_), 51.5 (CH), 37.9 (CH_2_), 35.9 (CH_2_), 28.4 (CH_2_); IR (ATR) 2849, 1433, 1080, 1019, 913, 716 cm^–1^; HRMS (APCI) *m/z:* [(M + H) – HBr]^+^ Calcd for C_8_H_13_O 125.0961; Found 125.0960.

#### (2R*,3S*)-3-Bromo-2-(2-methylallyl)­tetrahydro-2H-pyran (trans-**15b**)

To a cooled (−78 °C) solution of acetal **8** (0.301 g, 1.35 mmol) and 2-methallyltrimethylsilane (950 μL, 5.41 mmol) in CH_2_Cl_2_ (13 mL) was added BF_3_·OEt_2_ (340 μL, 2.71 mmol) dropwise over 2 min. After 1 h, the mixture was warmed to 20 °C and stirred for an additional 2 h. Saturated aqueous NaHCO_3_ (15 mL) was then added, the layers were separated, and the aqueous layer was extracted with CH_2_Cl_2_ (3 × 20 mL). The combined organic layers were washed with brine (1 × 20 mL), dried over Na_2_SO_4_, filtered, and concentrated *in vacuo*. ^1^H NMR and ^13^C­{^1^H} NMR spectroscopic analysis of the unpurified reaction mixture revealed that alkene *trans*-**15b** was formed as a single diastereomer (dr >99:1). Purification by flash chromatography (2:98 EtOAc:hexanes) afforded alkene *trans*-**15b** as a light yellow oil (0.175 g, 59%). The relative stereochemical configuration of the compound was assigned by ^1^H NMR coupling constants: ^1^H NMR (400 MHz, CDCl_3_) δ 4.82 (d, *J* = 19.2, 2H), 4.03–3.99 (m, 1H), 3.79 (ddd, *J* = 11.6, 9.8, 4.5, 1H), 3.54 (td, *J* = 9.6, 2.4, 1H), 3.44 (td, *J* = 11.8, 2.2, 1H), 2.77 (d, *J* = 14.7, 1H), 2.49–2.42 (m, 1H), 2.22–2.16 (m, 1H), 2.06–1.95 (m, 1H), 1.83–1.72 (m, 4H), 1.67–1.62 (m, 1H); ^13^C­{^1^H} NMR (100 MHz, CDCl_3_) 142.5, 112.6, 80.8, 68.3, 52.4, 41.9, 36.0, 28.4, 22.6; IR (ATR) 3076, 2945, 1087, 1061, 889, 714 cm^–1^; HRMS (APCI) *m/z:* [(M + H) – HBr]^+^ Calcd for C_9_H_15_O 139.1117; Found 139.1114.

#### (2R*,3S*)-2-Allyl-3-bromotetrahydro-2H-pyran (trans-**15a**)

To a cooled (−78 °C) solution of acetal **8** (0.098 g, 0.44 mmol) and allyltributylstannane (560 μL, 1.81 mmol) in CH_2_Cl_2_ (5 mL) was added BF_3_·OEt_2_ (115 μL, 0.916 mmol) dropwise over 2 min. After 1 h, the mixture was warmed to 20 °C and stirred for an additional 2 h. Saturated aqueous NaHCO_3_ (5 mL) was then added, the layers were separated, and the aqueous layer was extracted with CH_2_Cl_2_ (3 × 10 mL). The combined organic layers were washed with brine (1 × 15 mL), dried over Na_2_SO_4_, filtered, and concentrated *in vacuo*. ^1^H NMR and ^13^C­{^1^H} NMR spectroscopic analysis of the unpurified reaction mixture revealed that alkene *trans*-**15a** was formed as a single diastereomer (dr >99:1). Purification by flash chromatography (5:95 EtOAc:hexanes) afforded alkene *trans*-**15a** as a light yellow oil (0.076 g, 84%). The spectroscopic data (^1^H NMR, ^13^C­{^1^H} NMR, HRMS, IR) for alkene *trans*-**15a** are consistent with the data reported in the literature.^14^

#### (2R*,3R*)-3-Bromotetrahydro-2H-pyran-2-carbonitrile (cis-**15c**) and (2R*,3S*)-3-bromotetrahydro-2H-pyran-2-carbonitrile (trans-**15c**)

To a cooled (−78 °C) solution of acetal **8** (0.115 g, 0.516 mmol) and trimethylsilyl cyanide (150 μL, 1.12 mmol) in CH_2_Cl_2_ (5 mL) was added BF_3_·OEt_2_ (150 μL, 0.955 mmol) dropwise over 2 min. After 1 h, the mixture was warmed to 20 °C and stirred for an additional 2 h. Saturated aqueous NaHCO_3_ (5 mL) was then added, the layers were separated, and the aqueous layer was extracted with CH_2_Cl_2_ (3 × 10 mL). The combined organic layers were washed with brine (1 × 15 mL), dried over Na_2_SO_4_, filtered, and concentrated *in vacuo*. ^1^H NMR and ^13^C­{^1^H} NMR spectroscopic analysis of the unpurified reaction mixture revealed that nitrile **15c** was formed as a 9:91 mixture of diastereomers (*cis*-**15c**:*trans*-**15c**). Purification by flash chromatography (15:85 EtOAc:hexanes) afforded nitriles *cis*-**15c** and *trans*-**15c** as a colorless oil (0.063 g, 64%) with a diastereomeric ratio of 9:91. This mixture was used for characterization. The relative stereochemical configurations of the compounds were assigned by ^1^H NMR coupling constants: IR (ATR) 2866, 1174, 1078, 1036, 937, 724 cm^–1^; HRMS (ESI) *m/z:* [(M + H) – HCN]^+^ Calcd for C_5_H_8_BrO 162.9753; Found 162.9753. *Major Diastereomer trans*-**15c** *:* ^1^H NMR (400 MHz, CDCl_3_) δ 4.43 (d, *J* = 7.4, 1H), 4.15 (ddd, *J* = 8.4, 7.6, 4.0, 1H); 4.05–4.00 (m, 1H), 3.69–3.63 (m, 1H); 2.50–2.43 (m, 1H), 2.04–1.88 (m, 2H), 1.78–1.69 (m, 1H); ^13^C­{^1^H} NMR (100 MHz, CDCl_3_) δ 115.8 (C), 70.9 (CH), 67.3 (CH_2_), 45.5 (CH), 32.1 (CH_2_), 24.4 (CH_2_). *Minor Diastereomer cis*-**15c** *:* ^1^H NMR (400 MHz, CDCl_3_, diagnostic peaks) δ 4.87 (dd, *J* = 4.8, 1.3, 1H), 2.38–2.30 (m, 1H), 2.26–2.15 (m, 1H); ^13^C­{^1^H} NMR (100 MHz, CDCl_3_, diagnostic peaks) δ 70.5 (CH), 64.2 (CH_2_), 43.6 (CH), 30.8 (CH_2_), 26.6 (CH_2_).

#### (2R*,3R*)-2-Allyl-3-fluorotetrahydrofuran (cis-**21a**) and (2R*,3S*)-2-allyl-3-fluorotetrahydrofuran (trans-**21a**)

A reported procedure^14^ was adapted to prepare alkene **21a**. To a cooled (−78 °C) solution of benzoate **10** (63.10 mg, 0.30 mmol) and allyltrimethylsilane (205 μL, 1.2 mmol) in CH_2_Cl_2_ (1.66 mL) was added SnCl_4_ (69 μL, 0.069 mmol, 1.0 M in CH_2_Cl_2_) dropwise over 2 min. After 1 h, the mixture was warmed to 20 °C and stirred for an additional 16 h. Saturated aqueous potassium sodium tartrate tetrahydrate (7 mL) was then added, the layers were separated, and the aqueous layer was extracted with CH_2_Cl_2_ (3 × 6 mL). The combined organic layers were washed with brine (1 × 10 mL), dried over Na_2_SO_4_, filtered, and concentrated *in vacuo*. ^13^C­{^1^H} NMR and ^19^F­{^1^H} NMR spectroscopic analysis of the unpurified reaction mixture revealed that alkene **21a** was formed as an 81:19 mixture of diastereomers (*cis*-**21a**:*trans*-**21a**). Purification by flash chromatography (1:99 MeOH:CH_2_Cl_2_) afforded alkene *cis*-**21a** and alkene *trans*-**21a** with a diastereomeric ratio of 86:14. This mixture was used for characterization. Due to the high volatility of the products, alkene **21a** was isolated and characterized with solvents as impurities. HRMS (ESI) *m/z:* [(M – H_2_O) + K]^+^ Calcd for C_7_H_9_FK 151.0325; Found 151.0318. *Major Diastereomer cis-* **21a** *:* ^13^C­{^1^H} NMR (125 MHz, CDCl_3_) δ 134.5 (CH), 117.3 (CH_2_), 94.3–92.8 (br d, ^1^ *J*_C–F_ = 182.5, CH), 82.2–82.0 (br d, ^2^ *J*_C–F_ = 19.6, CH), 66.1 (CH_2_), 33.7–33.5 (br d, ^2^ *J*_C–F_ = 21.8, CH_2_), 33.2–33.10 (^3^ *J*_C–F_ = 9.2, CH_2_); ^19^F­{^1^H} NMR (377 MHz, CDCl_3_) δ – 193.1 (s). *Minor Diastereomer trans-* **21a** *:* ^13^C­{^1^H} NMR (400 MHz, CDCl_3_) δ 133.7 (CH), 117.8 (CH_2_), 97.5–96.1 (br d, ^1^ *J*_C–F_ = 176.6, CH), 83.8–83.6 (br d, ^2^ *J*_C–F_ = 24.0, CH), 66.8 (CH_2_), 37.5–37.4 (^3^ *J*_C–F_ = 9.2, CH_2_), 33.14–33.0 (br d, ^2^ *J*_C–F_ = 21.7, CH_2_); ^19^F­{^1^H} NMR (377 MHz, CDCl_3_) δ – 177.0 (s).

#### 2-((2R*,3R*)-3-Fluorotetrahydrofuran-2-yl)-1-phenylethan-1-one (cis-**21b**)

A reported procedure^14^ was adapted to prepare ketone **21b**. To a cooled (−78 °C) solution of benzoate **10** (210.2 mg, 1.0 mmol) and trimethyl­((1-phenylvinyl)­oxy)­silane (820 μL, 4.0 mmol) in CH_2_Cl_2_ (5.55 mL) was added SnCl_4_ (468 μL, 0.468 mmol, 1.0 M in CH_2_Cl_2_) dropwise over 2 min. After 1 h, the mixture was warmed to 20 °C and stirred for an additional 16 h. Saturated aqueous potassium sodium tartrate tetrahydrate (10 mL) was then added, the layers were separated, and the aqueous layer was extracted with CH_2_Cl_2_ (3 × 10 mL). The combined organic layers were washed with brine (1 × 9 mL), dried over Na_2_SO_4_, filtered, and concentrated *in vacuo*. ^1^H NMR, ^13^C­{^1^H} NMR, and ^19^F­{^1^H} NMR spectroscopic analysis of the unpurified mixture revealed that ketone *cis*-**21b** was formed as a single diastereomer (dr ≥ 99:1). Purification by flash chromatography (15:85 EtOAc:hexanes) afforded ketone *cis*-**21b** as a yellow solid (70 mg, 34%). X-ray quality crystals were grown by slow evaporation of a solution of ketone *cis*-**21b** in MeOH. The relative stereochemical configuration of ketone *cis*-**21b** was assigned by X-ray crystallographic analysis: mp = 50–55 °C; IR (ATR) 2920, 1683, 1596, 1213, 798 cm^–1^; ^1^H NMR (500 MHz, CDCl_3_) δ 7.99 (d, *J* = 7.8, 2H), 7.57 (t, *J* = 7.3, 1H), 7.47 (t, *J* = 7.7, 2H), 5.39–5.27 (m, 1H), 4.42–4.31 (m, 1H), 4.10–4.06 (m, 1H), 3.88–3.84 (m, 1H), 3.49–3.37 (m, 2H), 2.33–2.22 (m, 2H); ^13^C­{^1^H} NMR (125 MHz, CDCl_3_) δ 197.8 (C), 136.8 (C), 133.7 (CH), 128.7 (CH), 128.2 (CH), 94.5–93.1 (br d, ^1^ *J*_C–F_ = 181.9, CH), 78.7–78.5 (br d, ^2^ *J*_C–F_ = 19.3, CH), 66.1 (CH_2_), 37.8–37.7 (br d, ^3^ *J*_C–F_ = 10.1, CH_2_), 33.7–33.6 (br d, ^2^ *J*_C–F_ = 22.9, CH_2_); ^19^F­{^1^H} NMR (377 MHz, CDCl_3_) δ – 191.4 (s); HRMS (ESI) *m/z:* [M + Na]^+^ Calcd for C_12_H_13_FO_2_Na 231.0792; Found 231.0802.

#### 2-((2R*,3R*)-3-Fluorotetrahydrofuran-2-yl)-1-phenylethan-1-one (cis-**21b**) and 2-((2R*,3S*)-3-fluorotetrahydrofuran-2-yl)-1-phenylethan-1-one (trans-**21b**)

A reported procedure^14^ was adapted to prepare ketone **21b**. To a cooled (−45 °C) solution of benzoate **10** (63.06 mg, 0.30 mmol) and trimethyl­((1-phenylvinyl)­oxy)­silane (73.8 μL, 0.36 mmol) in MeCN (1.67 mL) was added SnCl_4_ (69 μL, 0.069 mmol, 1.0 M in CH_2_Cl_2_) dropwise over 2 min (the composition of the solvent is 94% MeCN:6% CH_2_Cl_2_). After 1 h, the mixture was warmed to 20 °C and stirred for an additional 20 h. Saturated aqueous potassium sodium tartrate tetrahydrate (10 mL) was then added, the layers were separated, and the aqueous layer was extracted with CH_2_Cl_2_ (3 × 10 mL). The combined organic layers were washed with brine (1 × 10 mL), dried over Na_2_SO_4_, filtered, and concentrated *in vacuo*. ^1^H NMR, ^13^C­{^1^H} NMR, and ^19^F­{^1^H} NMR spectroscopic analysis of the unpurified mixture revealed that ketone **21b** was formed as a 68:32 mixture of diastereomers (*cis*-**21b**:*trans*-**21b**). Purification by flash chromatography (15:85 EtOAc:hexanes) afforded ketones *cis*-**21b** and *trans*-**21b** as a yellow solid (16.8 mg, 27%) with a diastereomeric ratio of 66:34. This mixture was used for characterization. The spectroscopic data (^1^H NMR, ^13^C­{^1^H} NMR, ^19^F­{^1^H} NMR, IR, HRMS) for *cis*-**21b** are consistent with the data reported for the same compound prepared from benzoate **10** and trimethyl­((1-phenylvinyl)­oxy)­silane in CH_2_Cl_2_. *Minor Diastereomer trans-* **21b** *:* ^1^H NMR (400 MHz, CDCl_3_, diagnostic peaks) δ 5.20–5.04 (m, 1H), 4.66–4.55 (m, 1H), 3.96–3.90 (m, 1H), 3.31–3.25 (m, 1H), 3.12–3.06 (m, 1H); ^13^C­{^1^H} NMR (100 MHz, CDCl_3_) δ 197.2 (C), 136.7 (C), 133.4 (CH), 128.7 (CH), 128.2 (CH), 96.9 (br d, ^1^ *J*_C–F_ = 178.3, CH), 80.7 (br d, ^2^ *J*_C–F_ = 26.8, CH), 66.8 (CH_2_), 41.6 (br d, ^3^ *J*_C–F_ = 8.8, CH_2_), 32.9 (br d, ^2^ *J*_C–F_ = 21.2, CH_2_); ^19^F­{^1^H} NMR (377 MHz, CDCl_3_) δ – 175.3 (s).

#### (2R*,3R*)-3-Fluorotetrahydrofuran-2-ol (**22**)

To a cooled solution (0 °C) of 2,3-dihydrofuran **9** (1.5 g, 22 mmol), Selectfluor (12 g, 33 mmol), and CH_3_NO_2_ (55 mL) was added H_2_O (11 mL). After 5 min, the mixture was warmed to 25 °C for 5 min and was then heated to 110 °C with an oil bath. After 2 h, the mixture was concentrated *in vacuo*. ^1^H NMR and ^13^C­{^1^H} NMR spectroscopic analysis of the unpurified reaction mixture revealed that alcohol **22** was formed as a 98:2 mixture of diastereomers (*trans*-**22**:*cis*-**22**). Purification by flash chromatography (60:40 EtOAc:hexanes) afforded the major diastereomer *trans*-**22** as a yellow oil (0.778 g, 33%): IR (ATR) 3399, 2981, 1210, 1070, 1026, 977 cm^–1^; ^1^H NMR (400 MHz, CDCl_3_) δ 5.52 (br d, *J* = 10.0, 1H), 5.01 (br dd, *J* = 52.7, 4.6 Hz, 1H), 4.17 – 4.12 (m, 2H), 2.93 – 2.81 (m, 1H), 2.39 – 2.08 (m, 2H); ^13^C­{^1^H} NMR (100 MHz, CDCl_3_) δ 100.3–100.0 (d, ^*2*^ *J*_C–F_ = 32.7, CH), 96.5–94.7 (d, ^*1*^ *J*_C–F_ = 176.3, CH), 66.8 (CH_2_), 29.9–29.7 (d, ^*2*^ *J*_C–F_ = 20.6, CH_2_); ^19^F­{^1^H} NMR (377 MHz, CDCl_3_) δ – 189.0 (s); MS (GCMS) *m/z:* [(M + H) – H_2_O]^+^ Calcd for C_4_H_7_FO_2_ 89.0403; Found 89.0.

#### (2R*,3S*)-3-Fluorotetrahydrofuran-2-yl (E)-2,2,2-trifluoro-*N*-phenylacetimidate (trans-**23**) and (2R*,3R*)-3-fluorotetrahydrofuran-2-yl (E)-2,2,2-trifluoro-*N*-phenylacetimidate (cis-**23**)

To a solution of (*E*)-2,2,2-trifluoro-*N*-phenylacetimidoyl chloride^58^ (1.0 mL, 6.2 mmol) in CH_2_Cl_2_ (10 mL) was added hemiacetal **22** (0.33 g, 3.1 mmol). After 1 min, K_2_CO_3_ (0.85 g, 6.2 mmol) was added, and the reaction mixture was stirred for an additional 12 h. The reaction mixture was then filtered through diatomaceous earth and concentrated *in vacuo*. ^1^H NMR and ^13^C­{^1^H} NMR spectroscopic analysis of the unpurified reaction mixture revealed that trifluoroacetimidate **23** was formed as a 67:33 mixture of diastereomers (*trans*-**23**:*cis*-**23**). Purification by flash chromatography (16:84 EtOAc:hexanes) afforded trifluoroacetimidate *trans*-**23** as a yellow oil (0.298 g, 35%): IR (ATR) 1712, 1344, 1208, 1148, 1112, 939 cm^–1^; ^1^H NMR (400 MHz, CDCl_3_) δ 7.34–7.28 (m, 2H), 7.12 (t, *J* = 7.4, 1H), 6.86 (d, *J* = 7.5, 2H), 6.42 (br s, 1H), 5.38–5.06 (m, 1H), 4.34–4.17 (m, 2H), 2.48–2.14 (m, 2H); ^13^C­{^1^H} NMR (100 MHz, CDCl_3_) δ 143.6 (CF), 129.4 (CH), 128.8 (CH), 124.4 (CH), 120.4 (CH), 119.6 (CH), 102.2 (CH), 95.3–93.6 (d, ^*1*^ *J*_C–F_ = 179.9, CH), 68.7 (CH_2_), 29.7–29.5 (d, ^*2*^ *J*_C–F_ = 20.6 Hz, CH_2_); ^19^F­{^1^H} NMR (377 MHz, CDCl_3_) δ – 189.0 (s), – 68.0 (s); HRMS (ESI) *m/z:* [M + Na]^+^ Calcd for C_12_H_11_F_4_NNaO_2_ 300.0624; Found 300.0624.

#### (2R*,3S*)-2-Allyl-3-chlorotetrahydrofuran (trans-**24a**) and (2R*,3R*)-3-chlorotetrahydrofuran-2-ol (trans-**26**)

To a cooled (−78 °C) solution of acetal **11** (0.204 g, 1.24 mmol) and allyldimethylchlorosilane (740 μL, 4.90 mmol) in CH_2_Cl_2_ (12 mL) was added BF_3_·OEt_2_ (310 μL, 2.47 mmol) dropwise over 2 min. After 1 h, the mixture was warmed to 20 °C and stirred for an additional 2 h. Saturated aqueous NaHCO_3_ (10 mL) was then added, the layers were separated, and the aqueous layer was extracted with CH_2_Cl_2_ (3 × 15 mL). The combined organic layers were washed with brine (1 × 15 mL), dried over Na_2_SO_4_, filtered, and concentrated *in vacuo*. ^1^H NMR and ^13^C­{^1^H} NMR spectroscopic analysis of the unpurified reaction mixture revealed that alkene *trans*-**24a** was formed as a single diastereomer (dr >99:1; 81% conversion). Analysis of the ^1^H NMR and ^13^C­{^1^H} NMR spectra of the unpurified reaction mixture also revealed the formation of hemiacetal *trans*-**26** as a single diastereomer (dr >99:1; 19% yield). Purification by flash chromatography (5:95 EtOAc:hexanes) afforded alkene *trans*-**24a** as a colorless oil (0.054 g, 30%). The spectroscopic data (^1^H NMR, ^13^C­{^1^H} NMR, HRMS, IR) for alkene *trans*-**24a** are consistent with the data reported in the literature.^14^ The spectroscopic data (^1^H NMR, ^13^C­{^1^H} NMR, HRMS, IR) for hemiacetal **26** are consistent with the data reported in literature.^14^

#### (2R*,3R*)-2-Allyl-3-chlorotetrahydrofuran (cis-**24a**) and (2R*,3S*)-2-allyl-3-chlorotetrahydrofuran (trans-**24a**)

To a cooled (−78 °C) solution of acetal **11** (1.014 g, 6.161 mmol) and allyltrimethylsilane (3.90 mL, 24.5 mmol) in CH_2_Cl_2_ (10 mL) was added BF_3_·OEt_2_ (1.50 mL, 11.9 mmol) dropwise over 2 min. After 1 h, the mixture was warmed to 20 °C and stirred for an additional 2 h. Saturated aqueous NaHCO_3_ (10 mL) was then added, the layers were separated, and the aqueous layer was extracted with CH_2_Cl_2_ (3 × 15 mL). The combined organic layers were washed with brine (1 × 15 mL), dried over Na_2_SO_4_, filtered, and concentrated *in vacuo*. ^1^H NMR and ^13^C­{^1^H} NMR spectroscopic analysis of the unpurified reaction mixture revealed that alkene **24a** was formed as a 14:86 mixture of diastereomers (*cis*-**24a**:*trans*-**24a**). Purification by flash chromatography (3:97 EtOAc:hexanes) afforded the major diastereomer *trans*-**24a** as a colorless oil (0.798 g, 88%) and the minor diastereomer *cis*-**24a** as a colorless oil (0.066 g, 7%). The spectroscopic data (^1^H NMR, ^13^C­{^1^H} NMR, HRMS, IR) for alkenes *trans*-**24a** and *cis*-**24a** are consistent with the data reported in the literature.^14^ *Major Diastereomer trans*-**24a** *:* ^1^H NMR (400 MHz, CDCl_3_) δ 5.88–5.78 (m, 1H), 5.17–5.11 (m, 2H), 4.03–3.93 (m, 4H), 2.45–2.36 (m, 2H), 2.33–2.26 (m, 1H), 2.17–2.09 (m, 1H); ^13^C­{^1^H} NMR (100 MHz, CDCl_3_) δ 133.6 (CH), 117.9 (CH_2_), 86.1 (CH), 66.5 (CH_2_), 59.4 (CH), 37.7 (CH_2_), 36.1 (CH_2_); IR (ATR) 2981, 1065, 997, 916, 836, 712 cm^–1^; HRMS (APCI) *m/z:* [(M + H) – C_4_H_8_]^+^ Calcd for C_4_H_6_ClO 105.0102; Found 105.0099. *Minor Diastereomer cis* **-24a** *:* ^1^H NMR (400 MHz, CDCl_3_) δ 5.88–5.78 (m, 1H), 5.22–5.09 (m, 2H), 4.43 (ddd, *J* = 4.8, 3.1, 1.2, 1H), 4.17–4.11 (m, 1H), 3.94–3.86 (m, 2H), 2.57–2.42 (m, 3H), 2.32–2.25 (m 1H); ^13^C­{^1^H} NMR (100 MHz, CDCl_3_, diagnostic peaks) δ 134.0 (CH), 117.7 (CH_2_), 81.8 (CH), 65.8 (CH_2_), 61.7 (CH), 36.9 (CH_2_), 35.7 (CH_2_).

#### (2R*,3R*)-3-Chloro-2-(2-methylallyl)­tetrahydrofuran (cis-**24b**) and (2R*,3S*)-3-chloro-2-(2-methylallyl)­tetrahydrofuran (trans-**24b**)

To a cooled (−78 °C) solution of acetal **11** (0.189 g, 1.15 mmol) and 2-methallytrimethylsilane (770 μL, 4.38 mmol) in CH_2_Cl_2_ (10 mL) was added BF_3_·OEt_2_ (280 μL, 2.23 mmol) dropwise over 2 min. After 1 h, the mixture was warmed to 20 °C and stirred for an additional 2 h. Saturated aqueous NaHCO_3_ (10 mL) was then added, the layers were separated, and the aqueous layer was extracted with CH_2_Cl_2_ (3 × 15 mL). The combined organic layers were washed with brine (1 × 15 mL), dried over Na_2_SO_4_, filtered, and concentrated *in vacuo*. ^1^H NMR and ^13^C­{^1^H} NMR spectroscopic analysis of the unpurified reaction mixture revealed that alkene **24b** was formed as a 53:47 mixture of diastereomers (*cis*-**24b**:*trans*-**24b**). Purification by flash chromatography (3:97 EtOAc:hexanes) afforded the major diastereomer *cis*-**24b** as a colorless oil (0.057 g, 31%) and the minor diastereomer *trans*-**24b** as a colorless oil (0.039 g, 21%). The relative stereochemical configuration of alkene **24b** was assigned by ^1^H NMR spectroscopic correlation to alkene **24a**. *Major Diastereomer cis*-**24b** *:* ^1^H NMR (400 MHz, CDCl_3_) δ 4.86–4.84 (m, 2H), 4.44 (ddd, *J* = 4.8, 3.1, 1.2, 1H), 4.17–4.11 (m, 1H), 4.04–4.00 (m, 1H), 3.94–3.89 (m, 1H), 2.52–2.39 (m, 3H), 2.32–2.26 (m, 1H), 1.79 (s, 3H); ^13^C­{^1^H} NMR (100 MHz, CDCl_3_) δ 142.2 (C), 112.7 (CH_2_), 80.8 (CH), 65.9 (CH_2_), 62.3 (CH), 39.3 (CH_2_), 37.1 (CH_2_), 23.2 (CH_3_); IR (ATR) 2939, 1245, 1066, 1016, 891, 711 cm^–1^; HRMS (ESI) *m/z:* [M + H]^+^ Calcd for C_8_H_14_ClO 161.0728; Found 161.0735. Anal. Calcd for C_8_H_13_ClO: C, 59.82; H, 8.16. Found: C, 60.05; H, 8.00. *Minor Diastereomer trans-* **24b** *:* ^1^H NMR (400 MHz, CDCl_3_) δ 4.86–4.80 (m, 2H), 4.15–3.94 (m, 4H), 2.46–2.37 (m, 1H), 2.32–2.21 (m, 2H), 2.17–2.10 (m, 1H), 1.79 (s, 3H); ^13^C­{^1^H} NMR (100 MHz, CDCl_3_) δ 141.9 (C), 113.0 (CH_2_), 85.2 (CH), 66.5 (CH_2_), 60.0 (CH), 41.9 (CH_2_), 35.9 (CH_2_), 22.7 (CH_3_).

#### (2R*,3R*)-2-Allyl-3-chlorotetrahydrofuran (cis-**24a**) and (2R*,3S*)-2-allyl-3-chlorotetrahydrofuran (trans-**24a**)

To a cooled (−78 °C) solution of acetal **11** (0.208 g, 1.26 mmol) and allyltributylstannane (1.500 mL, 4.84 mmol) in CH_2_Cl_2_ (12 mL) was added BF_3_·OEt_2_ (310 μL, 2.47 mmol) dropwise over 2 min. After 1 h, the mixture was warmed to 20 °C and stirred for an additional 2 h. Saturated aqueous NaHCO_3_ (10 mL) was then added, the layers were separated, and the aqueous layer was extracted with CH_2_Cl_2_ (3 × 15 mL). The combined organic layers were washed with brine (1 × 15 mL), dried over Na_2_SO_4_, filtered, and concentrated *in vacuo*. ^1^H NMR and ^13^C­{^1^H} NMR spectroscopic analysis of the unpurified reaction mixture revealed that alkene **24a** was formed as a 48:52 mixture of diastereomers (*cis*-**24a**:*trans*-**24a**). Purification by flash chromatography (5:95 EtOAc:hexanes) afforded the major diastereomer *trans*-**24a** as a colorless oil (0.034 g, 18%) and the minor diastereomer *cis*-**24a** as a colorless oil (0.046 g, 25%). The spectroscopic data (^1^H NMR, ^13^C­{^1^H} NMR, HRMS, IR) for alkenes *cis*-**24a** and *trans*-**24a** are consistent with the data reported in the literature.^14^

#### 2-((2R*,3S*)-3-Chlorotetrahydrofuran-2-yl)-1-phenylethan-1-one (cis-**24c**) and 2-((2R*,3R*)-3-chlorotetrahydrofuran-2-yl)-1-phenylethan-1-one (trans-**24c**)

A reported procedure^14^ was adapted to prepare ketone **24c**. To a cooled (−78 °C) solution of acetate **11** (37.0 mg, 0.225 mmol) and trimethyl­((1-phenylvinyl)­oxy)­silane (265 μL, 1.29 mmol) in CH_2_Cl_2_ (789 μL) was added SnCl_4_ (444 μL, 0.444 mmol, 1.0 M in CH_2_Cl_2_) dropwise over 2 min. After 1 h, the mixture was warmed to 20 °C and stirred for an additional 3 h. Saturated aqueous potassium sodium tartrate tetrahydrate (6 mL) was then added, the layers were separated, and the aqueous layer was extracted with CH_2_Cl_2_ (3 × 3 mL). The combined organic layers were washed with brine (1 × 9 mL), dried over Na_2_SO_4_, filtered, and concentrated *in vacuo*. ^1^H NMR and ^13^C­{^1^H} NMR spectroscopic analysis of the unpurified mixture revealed that ketone **24c** was formed as a 37:63 mixture of diastereomers (*cis*-**24c**:*trans*-**24c**). Purification by flash chromatography (20:80 EtOAc:hexanes) afforded ketone *cis*-**24c** and *trans*-**24c** as a yellow oil (10.0 mg, 20%) with a diastereomeric ratio of 14:86. This mixture was used for characterization: IR (ATR) 2882, 1680, 1596, 1447, 1302, 1211, 1055, 749 cm^–1^; HRMS (ESI) *m/z:* [M + Na]^+^ Calcd for C_12_H_13_ClO_2_Na 247.0507; Found 247.0496. Major Diastereomer *trans*-**24c**: ^1^H NMR (400 MHz, CDCl_3_) δ 8.00–7.98 (m, 2H), 7.60–7.56 (m, 1H), 7.49–7.46 (m, 2H), 4.80–4.78 (m, 1H), 4.50–4.47 (m, 1H), 4.16–4.11 (m, 1H), 3.96–3.92 (m, 1H), 3.50–3.48 (m, 2H), 2.60–2.53 (m, 1H), 2.35–2.31 (m, 1H); ^13^C­{^1^H} NMR (100 MHz, CDCl_3_) δ 197.6 (C), 136.7 (C), 133.4 (CH), 128.7 (CH), 128.1 (CH), 78.6 (CH), 65.6 (CH_2_), 62.1 (CH), 40.5 (CH_2_), 36.9 (CH_2_). Minor Diastereomer *cis*-**24c**: ^1^H NMR (400 MHz, CDCl_3_, diagnostic peaks) δ 4.21–4.17 (m, 1H), 3.99–3.96 (dd, *J* = 5.8, 7.9, 1H), 3.25–3.22 (m, 2H), 2.51–2.42 (m, 1H), 2.22–2.15 (m, 1H); ^13^C­{^1^H} NMR (100 MHz, CDCl_3_, diasgnostic peaks) 197.2 (C), 128.6 (CH), 128.3 (CH), 82.9 (CH), 66.6 (CH_2_), 59.8 (CH), 42.1 (CH_2_), 35.7 (CH_2_).

#### (2R*,3R*)-3-Chlorotetrahydrofuran-2-carbonitrile (cis-**24d**) and (2R*,3S*)-3-chlorotetrahydrofuran-2-carbonitrile (trans-**24d**)

To a cooled (−78 °C) solution of acetal **11** (0.104 g, 0.632 mmol) and trimethylsilyl cyanide (250 μL, 2.00 mmol) in CH_2_Cl_2_ (6 mL) was added BF_3_·OEt_2_ (150 μL, 1.19 mmol) dropwise over 2 min. After 1 h, the mixture was warmed to 20 °C and stirred for an additional 2 h. Saturated aqueous NaHCO_3_ (5 mL) was then added, the layers were separated, and the aqueous layer was extracted with CH_2_Cl_2_ (3 × 10 mL). The combined organic layers were washed with brine (1 × 15 mL), dried over Na_2_SO_4_, filtered, and concentrated *in vacuo*. ^1^H NMR and ^13^C­{^1^H} NMR spectroscopic analysis of the unpurified reaction mixture revealed that nitrile **24d** was formed as a 48:52 mixture of diastereomers (*cis*-**24d**:*trans*-**24d**). Purification by flash chromatography (20:80 EtOAc:hexanes) afforded the major diastereomer *trans*-**24d** as a colorless oil (0.040 g, 48%) and the minor diastereomer *cis*-**24d** as a colorless oil (0.019 g, 23%). *Major Diastereomer trans*-**24d** *:* ^1^H NMR (400 MHz, CDCl_3_) δ 4.89 (d, *J* = 5.5, 1H), 4.47–4.43 (m, 1H), 4.29–4.22 (m, 1H), 4.11–1.06 (m, 1H), 2.57–2.49 (m, 1H), 2.38–2.31 (m, 1H); ^13^C­{^1^H} NMR (100 MHz, CDCl_3_) δ 115.2 (C), 72.6 (CH), 67.8 (CH_2_), 55.2 (CH), 34.9 (CH_2_); IR (ATR) 2901, 1284, 1093, 1054, 914, 807 cm^–1^; HRMS (ESI) *m/z:* [(M + H) – HCN]^+^ Calcd for C_4_H_6_ClO 105.0102; Found 105.0099. *Minor Diastereomer cis*-**24d** *:* ^1^H NMR (400 MHz, CDCl_3_) δ 4.76 (d, *J* = 1.7, 1H), 4.65 (dt, *J* = 5.9, 2.1, 1H), 4.24–4.20 (m, 2H), 2.69–2.60 (m, 1H), 2.35–2.29 (m, 1H); ^13^C­{^1^H} NMR (100 MHz, CDCl_3_) δ 116.4 (C), 74.2 (CH), 68.3 (CH_2_), 59.6 (CH), 35.5 (CH_2_).

#### (2R*,3S*)-2-Allyl-3-bromotetrahydrofuran (trans-**25a**)

To a cooled (−78 °C) solution of acetal **12** (0.111 g, 0.531 mmol) and allyldimethylchlorosilane (290 μL, 1.92 mmol) in CH_2_Cl_2_ (5 mL) was added BF_3_·OEt_2_ (120 μL, 0.955 mmol) dropwise over 2 min. After 1 h, the mixture was warmed to 20 °C and stirred for an additional 16 h. Saturated aqueous NaHCO_3_ (5 mL) was then added, the layers were separated, and the aqueous layer was extracted with CH_2_Cl_2_ (3 × 10 mL). The combined organic layers were washed with brine (1 × 15 mL), dried over Na_2_SO_4_, filtered, and concentrated *in vacuo*. ^1^H NMR and ^13^C­{^1^H} NMR spectroscopic analysis of the unpurified reaction mixture revealed that alkene *trans*-**25a** was formed as a single diastereomer (dr >99:1). Purification by flash chromatography (5:95 EtOAc:hexanes) afforded alkene *trans*-**25a** as a light yellow oil (0.068 g, 67%). The spectroscopic data (^1^H NMR, ^13^C­{^1^H} NMR, HRMS, IR) for alkene *trans*-**25a** are consistent with the data reported in the literature.^14^

#### (2R*,3S*)-2-Allyl-3-bromotetrahydrofuran (trans-**25a**)

To a cooled (−78 °C) solution of acetal **12** (0.219 g, 1.05 mmol) and allyltrimethylsilane (640 μL, 4.03 mmol) in CH_2_Cl_2_ (10 mL) was added BF_3_·OEt_2_ (250 μL, 1.99 mmol) dropwise over 2 min. After 1 h, the mixture was warmed to 20 °C and stirred for an additional 2 h. Saturated aqueous NaHCO_3_ (10 mL) was then added, the layers were separated, and the aqueous layer was extracted with CH_2_Cl_2_ (3 × 15 mL). The combined organic layers were washed with brine (1 × 15 mL), dried over Na_2_SO_4_, filtered, and concentrated *in vacuo*. ^1^H NMR and ^13^C­{^1^H} NMR spectroscopic analysis of the unpurified reaction mixture revealed that alkene *trans*-**25a** was formed as a single diastereomer (dr >99:1). Purification by flash chromatography (3:97 EtOAc:hexanes) afforded alkene *trans*-**25a** as a light yellow oil (0.163 g, 81%). The spectroscopic data (^1^H NMR, ^13^C­{^1^H} NMR, HRMS, IR) for alkene *trans*-**25a** are consistent with the data reported in the literature:^14 1^H NMR (400 MHz, CDCl_3_) δ 5.88–5.78 (m, 1H), 5.17–5.11 (m, 2H), 4.10 (dt, *J* = 6.8, 5.6, 1H), 4.02–3.92 (m, 3H), 2.53–2.39 (m, 2H), 2.32–2.20 (m, 2H); ^13^C­{^1^H} NMR (100 MHz, CDCl_3_) δ 133.6 (CH), 117.9 (CH_2_), 86.2 (CH), 66.7 (CH_2_), 48.8 (CH), 37.6 (CH_2_), 36.5 (CH_2_); IR (ATR) 2980, 1181, 1062, 997, 916, 834 cm^–1^; HRMS (APCI) *m/z:* [(M + H) – HBr]^+^ Calcd for C_7_H_11_O 111.0804; Found 111.0807.

#### (2R*,3S*)-3-Bromo-2-(2-methylallyl)­tetrahydrofuran (trans-**25b**)

To a cooled (−78 °C) solution of acetal **12** (0.224 g, 1.07 mmol) and 2-methallyltrimethylsilane (705 μL, 4.01 mmol) in CH_2_Cl_2_ (10 mL) was added BF_3_·OEt_2_ (250 μL, 1.99 mmol) dropwise over 2 min. After 1 h, the mixture was warmed to 20 °C and stirred for an additional 2 h. Saturated aqueous NaHCO_3_ (10 mL) was then added, the layers were separated, and the aqueous layer was extracted with CH_2_Cl_2_ (3 × 15 mL). The combined organic layers were washed with brine (1 × 15 mL), dried over Na_2_SO_4_, filtered, and concentrated *in vacuo*. ^1^H NMR and ^13^C­{^1^H} NMR spectroscopic analysis of the unpurified reaction mixture revealed that alkene *trans*-**25b** was formed as a single diastereomer (dr >99:1). Purification through a silica plug (2:98 EtOAc:hexanes) afforded the major diastereomer *trans*-**25b** as a colorless oil (0.116 g, 53%). The relative stereochemical configuration of alkene *trans*-**25b** was assigned by ^1^H NMR spectroscopic correlation to alkene *trans*-**25a**: ^1^H NMR (400 MHz, CDCl_3_) δ 4.87–4.79 (m, 2H), 4.21 (dt, *J* = 7.7, 5.3, 1H), 4.03–3.93 (m, 3H), 2.54–2.45 (m, 1H), 2.37–2.18 (m, 3H), 1.79 (s, 3H); ^13^C­{^1^H} NMR (100 MHz, CDCl_3_) δ 141.9 (C), 113.0 (CH_2_), 85.3 (CH), 66.6 (CH_2_), 49.6 (CH), 41.8 (CH_2_), 36.4 (CH_2_), 22.7 (CH_3_); IR (ATR) 2937, 1440, 1073, 1013, 891, 839 cm^–1^; HRMS (APCI) *m/z:* [(M + H) – HBr]^+^ Calcd for C_8_H_13_O 125.0961; Found 125.0960.

#### (2R*,3S*)-2-Allyl-3-bromotetrahydrofuran (trans-**25a**)

To a cooled (−78 °C) solution of acetal **12** (0.100 g, 0.478 mmol) and allyltributylstannane (600 μL, 1.94 mmol) in CH_2_Cl_2_ (5 mL) was added BF_3_·OEt_2_ (120 μL, 0.955 mmol) dropwise over 2 min. After 1 h, the mixture was warmed to 20 °C and stirred for an additional 16 h. Saturated aqueous NaHCO_3_ (5 mL) was then added, the layers were separated, and the aqueous layer was extracted with CH_2_Cl_2_ (3 × 10 mL). The combined organic layers were washed with brine (1 × 15 mL), dried over Na_2_SO_4_, filtered, and concentrated *in vacuo*. ^1^H NMR and ^13^C­{^1^H} NMR spectroscopic analysis of the unpurified reaction mixture revealed that alkene *trans*-**25a** was formed as a single diastereomer (dr >99:1). Purification by flash chromatography (5:95 EtOAc:hexanes) afforded alkene *trans*-**25a** as a light yellow oil (0.072 g, 79%). The spectroscopic data (^1^H NMR, ^13^C­{^1^H} NMR, HRMS, IR) for alkene *trans*-**25a** are consistent with the data reported in the literature.^14^

#### 2-((2R*,3R*)-3-Bromotetrahydrofuran-2-yl)-1-phenylethan-1-one (cis-**25c**) and 2-((2R*,3S*)-3-bromotetrahydrofuran-2-yl)-1-phenylethan-1-one (trans-**25c**)

A reported procedure^14^ was adapted to prepare ketone **25c**. To a cooled (−78 °C) solution of acetal **12** (85.8 mg, 0.41 mmol) and trimethyl­((1-phenylvinyl)­oxy)­silane (328 μL, 1.6 mmol) in CH_2_Cl_2_ (2.2 mL) was added BF_3_·OEt_2_ (99 μL, 0.8 mmol) dropwise over 2 min. After 1 h, the mixture was warmed to 20 °C and stirred for an additional 1 h. Saturated aqueous potassium sodium tartrate tetrahydrate (6 mL) was then added, the layers were separated, and the aqueous layer was extracted with CH_2_Cl_2_ (3 × 6 mL). The combined organic layers were washed with brine (1 × 10 mL), dried over Na_2_SO_4_, filtered, and concentrated *in vacuo*. ^1^H NMR and ^13^C­{^1^H} NMR spectroscopic analysis of the unpurified mixture revealed that ketone **25c** was formed as a 7:93 mixture of diastereomers (*cis*-**25c**:*trans*-**25c**). Purification by flash chromatography (5:95 EtOAc:hexanes) afforded ketone *cis*-**25c** and *trans*-**25c** as a light yellow solid (44.0 mg, 40%) with a diastereomeric ratio of 7:93. This mixture was used for characterization. The relative stereochemical configurations of ketones *cis*-**25c** and *trans*-**25c** were assigned by ^1^H NMR spectroscopic correlation to alkene *trans*-**25a**: mp = 85–87 °C; IR (ATR) 2893, 1678, 1594, 1446, 1360, 1299, 1067, 754 cm^–1^; HRMS (ESI) *m/z:* [M + Na]^+^ Calcd for C_12_H_13_^79^BrO_2_ 290.9993; Found 290.9991; [M + Na]^+^ Calcd for C_12_H_13_^81^BrO_2_ 292.9972; Found 292.9972. *Major Diastereomer trans-* **25c** *:* ^1^H NMR (400 MHz, CDCl_3_) δ 7.98–7.95 (m, 2H), 7.60–7.56 (m, 1H), 7.49–7.45 (m, 2H), 4.59 (dt, *J* = 6.6, 5.6, 1H), 4.15 (dt, *J* = 7.4, 5.5, 1H), 4.03–3.99 (m, 2H), 3.26–3.23 (m, 2H), 2.59–2.50 (m, 1H), 2.33–2.25 (m, 1H); ^13^C­{^1^H} NMR (100 MHz, CDCl_3_) δ 197.2 (C), 136.8 (C), 133.4 (CH), 128.7 (CH), 128.3 (CH), 83.0 (CH), 66.9 (CH_2_), 48.9 (CH), 42.0 (CH_2_), 36.2 (CH_2_). *Minor Diastereomer cis-* **25c** *:* ^1^H NMR (400 MHz, CDCl_3_, diagnostic peaks) δ 4.88–4.86 (m, 1H), 4.32–4.28 (m, 1H), 3.57–3.43 (m, 2H), 2.76–2.66 (m, 1H), 2.49–2.43 (m, 1H); ^13^C­{^1^H} NMR (100 MHz, CDCl_3_, diagnostic peaks) δ 78.3 (CH), 65.7 (CH_2_), 55.1 (CH), 42.8 (CH_2_), 37.4 (CH_2_).

#### (2R*,3R*)-3-Bromotetrahydrofuran-2-carbonitrile (cis-**25d**) and (2R*,3S*)-3-bromotetrahydrofuran-2-carbonitrile (trans-**25d**)

To a cooled (−78 °C) solution of acetal **12** (0.101 g, 0.483 mmol) and trimethylsilyl cyanide (130 μL, 1.04 mmol) in CH_2_Cl_2_ (5 mL) was added BF_3_·OEt_2_ (120 μL, 0.955 mmol) dropwise over 2 min. After 1 h, the mixture was warmed to 20 °C and stirred for an additional 2 h. Saturated aqueous NaHCO_3_ (10 mL) was then added, the layers were separated, and the aqueous layer was extracted with CH_2_Cl_2_ (3 × 15 mL). The combined organic layers were washed with brine (1 × 15 mL), dried over Na_2_SO_4_, filtered, and concentrated *in vacuo*. ^1^H NMR and ^13^C­{^1^H} NMR spectroscopic analysis of the unpurified reaction mixture revealed that nitrile **25d** was formed as a 20:80 mixture of diastereomers (*cis*-**25d**:*trans*-**25d**). Purification by flash chromatography (15:85 EtOAc:hexanes) afforded the major diastereomer *trans*-**25d** as a colorless oil (0.057 g, 67%) and the minor diastereomer *cis*-**25d** as a colorless oil (0.014 g, 16%). The relative stereochemical configurations of the compounds were assigned by ^1^H NMR coupling constants. *Major Diastereomer trans*-**25d** *:* ^1^H NMR (400 MHz, CDCl_3_) δ 4.84 (d, *J* = 2.2, 1H), 4.59 (dt, *J* = 6.1, 2.5, 1H), 4.27–4.17 (m, 2H), 2.76–2.67 (m, 1H), 2.43–2.36 (m, 1H); ^13^C­{^1^H} NMR (100 MHz, CDCl_3_) δ 116.5 (C), 74.5 (CH), 68.4 (CH_2_), 47.1 (CH), 36.1 (CH_2_); IR (ATR) 2901, 1441, 1180, 1082, 908, 893 cm^–1^; HRMS (ESI) *m/z:* [(M + H) – HCN]^+^ Calcd for C_4_H_6_BrO 148.9597; Found 148.9598. *Minor Diastereomer cis*-**25d** *:* ^1^H NMR (400 MHz, CDCl_3_) δ 4.90 (d, *J* = 5.8, 1H), 4.34 (dt, *J* = 6.8, 6.1, 1H), 4.27–4.21 (m, 1H), 4.09–4.03 (m, 1H), 2.65–2.56 (m, 1H), 2.47–2.38 (m, 1H); ^13^C­{^1^H} NMR (100 MHz, CDCl_3_) δ 115.8 (C), 72.7 (CH), 68.0 (CH_2_), 42.8 (CH), 35.2 (CH_2_).

#### (3R*,2S*)-2-Chloro-3-fluorotetrahydrofuran (trans-**30**)

To a cooled solution (−60 °C) of benzoate **10** (0.005 g, 0.022 mmol) in CDCl_3_ (0.5 mL) was added SnCl_4_ (one drop, 1.0 M in CH_2_Cl_2_). The mixture stirred for 24 h at –60 °C. An ^1^H NMR spectrum was taken at –60 °C that identified the formation of anomeric chloride *trans*-**30** as a reasonable reactive intermediate (although the spectrum contained a number of impurities): ^1^H NMR (400 MHz, CDCl_3_, diagnostic peaks) δ 7.09 (d, *J* = 8.6, 1H), 6.29 (d, *J* = 9.3, 1H).

## Supplementary Material



## Data Availability

The data underlying this study are available in the published article and the Supporting Information.

## Notes

Stereochemical proofs; ^1^H, ^13^C­{^1^H},^19^F NMR, and HSQC spectra for new compounds; X-ray data; and crystallographic material (PDF)
