# Supplementary material for: Influence of Halogen Atoms and the Reactivity of Nucleophiles on Reactions of Tetrahydropyran and Tetrahydrofuran Acetals with C‑Nucleophiles: Hyperconjugation and Inductive Effects
Source: J Org Chem. 2025 Jul 3;90(28):9673–87. doi: 10.1021/acs.joc.4c03128 (PMC12281571; doi:10.1021/acs.joc.4c03128)
Supplement: Supplementary file 1 [file jo4c03128_si_001.pdf]

Influence of Halogen Atoms and the Reactivity of Nucleophiles on Reactions of Tetrahydropyran and Tetrahydrofuran Acetals with *C*-Nucleophiles: Hyperconjugation and Inductive Effects

Krystyna M. Demkiw,[1] Wouter A. Remmerswaal,[2] Asma Sheikh,[1] Ibrahim N. Sheikh,[1] Collin H. Witt,[1] Jeroen D. C. Codée,[2]* K. A. Woerpel[1]*

[1]Department of Chemistry, New York University
100 Washington Square East, New York, New York 10003
Email: kwoerpel@nyu.edu
[2]Leiden Institute of Chemistry, Leiden University
Einsteinweg 55, 2300 RA Leiden (The Netherlands)
Email: jcodee@chem.leidenuniv.nl

Supporting Information

Table of Contents

I. Stereochemical Correlations and Proofs S1

A. Assignments of Relative Stereochemical Configuration of Tetrahydropyrans by ^{1}H NMR Coupling Constants S1

B. Assignment of Relative Stereochemical Configurations of Tetrahydrofurans by ^{1}H NMR Coupling Constants S4

C. Assignment of Relative Stereochemical Configurations of Alkenes **24b** and **25b** and Ketone **25c** by ^{1}H NMR Correlations with Alkenes **24a** and **25a** S4

II. X-Ray Crystallographic Data S7

III. References S14

IV. Selected ^{1}H, ^{13}C{^{1}H}, ^{19}F{^{1}H}, and HSQC NMR Spectra S16

I. Stereochemical Correlations and Proofs

A. Assignments of Relative Stereochemical Configuration of Tetrahydropyrans by ^{1}H NMR Coupling Constants

Peaks were assigned in the ^{1}H NMR spectra by ^{1}H NMR chemical shifts and ^{1}H NMR coupling constants. The stereochemical configurations of the products were determined using ^{1}H NMR coupling constants because the coupling constants between protons on six-membered rings are well-documented.[6] Protons will either adopt an axial or equatorial orientation on the ring (or a mixture of these two conformers), and the coupling between adjacent protons (the coupling constant, *J*) will help to elucidate those orientations. The relative orientation between protons on the six-membered ring are represented by the coupling constant, and they are as follows: axial–

axial, 8 to 13 Hz; axial–equatorial, 2 to 6 Hz; equatorial–equatorial, 2 to 9 Hz.[6] A-values of substituents on six-membered rings provide insight on the positioning of the allyl group on the ring, which is expected to be equatorial in alkenes **13b**, **14b**, and **15b**,[7-9] and the positioning of the nitrile group on the ring, which is expected to be axial in nitrile **15c**, due to anomeric interactions.[10-12]

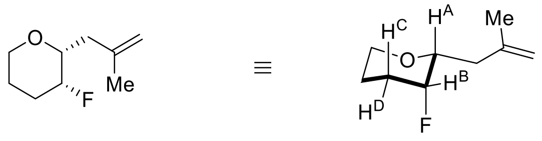

Alkene *cis*-13b (1,2-*cis*, major diastereomer):
^{1}H NMR (400 MHz, $CDCl_3$) δ 4.50 (H^B: dt, J = 47.5 (eq^B–F), 2.6 (eq^B–ax^A), 2.6 (eq^B–ax^C), 1H)
Note: The splitting pattern of H^B indicates that H^B is equatorial (dt, J = 47.5, 2.6, 2.6 Hz), which places the fluorine atom in an axial orientation. The structure is 1,2-*cis* because the larger allyl group should adopt an equatorial orientation, as noted in X-ray crystallographic structures of similar compounds.[13-15]

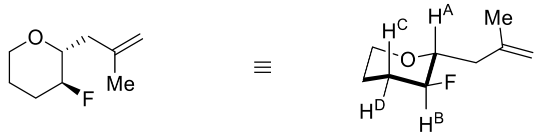

Alkene *trans*-13b (1,2-*trans*, minor diastereomer):
^{1}H NMR (400 MHz, $CDCl_3$) δ 4.16 (H^B: dddd, J = 48.8 (ax^B–F), 10.1 (ax^B–ax^A), 8.9 (ax^B–ax^C), 5.1 (ax^B–eq^D), 1H)
Note: The splitting pattern of H^B indicates that H^B is axial (dddd, J = 48.8, 10.1, 8.9, 5.1 Hz), which places the fluorine atom in an equatorial orientation. The structure is 1,2-*trans* because the larger allyl group should adopt an equatorial orientation, as noted in X-ray crystallographic structures of similar compounds.[13-15]

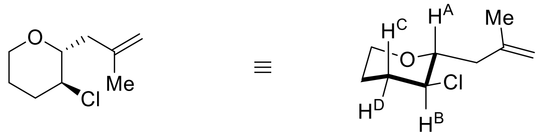

Alkene *trans*-14b (1,2-*trans*, major diastereomer):
^{1}H NMR (400 MHz, $CDCl_3$) δ 3.62 (H^B: ddd, J = 10.9 (ax^B–ax^A), 9.8 (ax^B–ax^C), 4.6 (ax^B–eq^D), 1H)
Note: The splitting pattern of H^B indicates that H^B is axial (ddd, J = 10.9, 9.8, 4.6 Hz), which places the chlorine atom in an equatorial orientation. The splitting pattern of H^B also indicates that H^A is axial, which places the allyl group in an equatorial orientation. The structure is 1,2-*trans* diequatorial because the larger allyl group should adopt an equatorial orientation, as noted in X-ray crystallographic structures of similar compounds.[13-15]

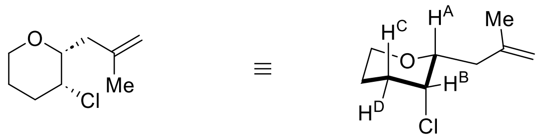

Alkene *cis*-14b (1,2-*cis*, minor diastereomer):
^{1}H NMR (400 MHz, $CDCl_3$) δ 3.60 (H^B: td, *J* = 6.7 (eq^B–ax^A), 6.7 (eq^B–ax^C), 1.2 (eq^B–eq^D), 1H)
Note: The splitting pattern of H^B indicates that H^B is equatorial (td, *J* = 6.7, 6.7, 1.2 Hz), which places the chlorine atom in an axial orientation. The structure is 1,2-*cis* because the larger allyl group should adopt an equatorial orientation, as noted in X-ray crystallographic structures of similar compounds.[13-15]

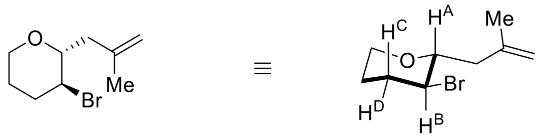

Alkene *trans*-15b (1,2-*trans*, major diastereomer):
^{1}H NMR (400 MHz, $CDCl_3$) δ 3.79 (H^B: ddd, *J* = 11.6 (ax^B–ax^A), 9.8 (ax^B–ax^C), 4.5 (ax^B–eq^D), 1H)
Note: The splitting pattern of H^B indicates that H^B is axial (ddd, *J* = 11.6, 9.8, 4.5 Hz), which places the bromine atom in an equatorial orientation. The splitting pattern of H^B also indicates that H^A is axial, which places the allyl group in an equatorial orientation. The structure is 1,2-*trans* diequatorial because the larger allyl group should adopt an equatorial orientation, as noted in X-ray crystallographic structures of similar compounds.[13-15]

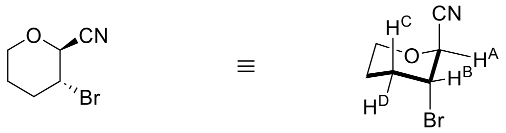

Alkene *trans*-15c (1,2-*trans*, major diastereomer):
^{1}H NMR (400 MHz, $CDCl_3$) δ 4.43 (H^A: d, *J* = 7.4 (eq^A–eq^B), 1H), 4.15 (H^B: ddd, *J* = 8.4 (eq^B–eq^D), 7.6 (eq^B–eq^A), 4.0 (eq^B–ax^C), 1H)
Note: The splitting pattern of H^B indicates that H^B is equatorial (ddd, *J* = 8.4, 7.6, 4.0 Hz), which places the bromine atom in an axial orientation. The splitting pattern of H^B also indicates that H^A is equatorial, which places the nitrile group in an axial orientation. The nitrile group at C-1 will adopt an axial orientation due to anomeric interactions,[16,17] which would consequently position the bromine atom at C-2 in an axial orientation.

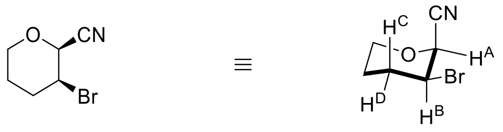

Alkene *cis*-15c (1,2-*cis*, minor diastereomer):
^{1}H NMR (400 MHz, $CDCl_3$) δ 4.87 (H^A: dd, *J* = 4.8 (eq^A–ax^B), 1.3 (eq^A–eq^D), 1H)
Note: The splitting pattern of H^A indicates that H^A is equatorial (dd, *J* = 4.8, 1.3 Hz). A similar *J*-value coupling constant between eq^A–ax^B was reported in a nitrile-containing compound with an axially oriented nitrile group at the anomeric center.[16,17] Such an interaction indicates that the bromine atom at C-2 is equatorially oriented.

B. Assignment of Relative Stereochemical Configurations of Tetrahydrofurans by ^{1}H NMR Coupling Constants

The tentative relative stereochemical configurations of nitrile **25d** were assigned by analysis of *J*-value coupling constants. For all conformations of the nitrile substituted furans, the nitrile substituent at C-1 should be in an axial orientation due to strong anomeric interaction between the lone pairs on the endocyclic oxygen atom and the exocyclic carbon–nitrogen anti-bonding orbital ($n_O \rightarrow \sigma^*_{C–N}$).[16,18] A small *J*-value coupling constant for the anomeric proton indicates a 1,2-*trans* configuration, with an equatorial–equatorial interaction of protons. Consequently, a larger coupling constant (*J*) for the anomeric proton are consistent with an axial–equatorial interaction of protons.[19]

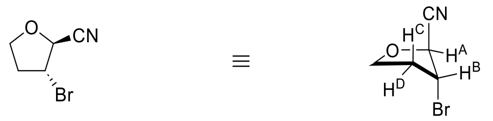

Nitrile *trans*-25d (1,2-*trans*, major diastereomer):

^{1}H NMR (400 MHz, $CDCl_3$) δ 4.59 (H^B: dt, *J* = 6.1 (eq^B–ax^C), 2.5 (eq^B–eq^A), 2.5 (eq^B–eq^D), 1H)
Note: The splitting pattern of H^B indicates that H^B is equatorial (dt, *J* = 6.1, 2.5, 2.5 Hz), which places the bromine atom in an axial orientation. The splitting pattern of H^B also indicates that H^A is equatorial, which places the nitrile group in an axial orientation. The structure is 1,2-*trans* diaxial because the nitrile group should adopt an axial orientation due to an anomeric effect.[20,21]

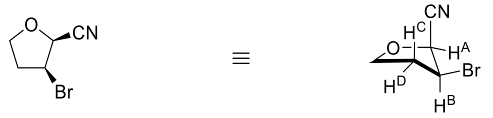

Nitrile *cis*-25d (1,2-*cis*, minor diastereomer):

^{1}H NMR (400 MHz, $CDCl_3$) δ 4.34 (H^B: dt, *J* = 6.8 (ax^B–eq^A), 6.8 (ax^B–eq^D), 6.1 (ax^B–ax^D), 1H)
Note: The splitting pattern of H^B indicates that H^B is axial (dt, *J* = 6.8, 6.1, 6.1 Hz), which places the bromine atom in an equatorial orientation. The splitting pattern of H^B also indicates that H^A is equatorial, which places the nitrile group in an axial orientation. The structure is 1,2-*cis*, where the nitrile group adopts an axial orientation due to an anomeric effect.[20,21]

C. Assignment of Relative Stereochemical Configurations of Alkenes 24b and 25b and Ketone 25c by ^{1}H NMR Correlations with Alkenes 24a and 25a

The stereochemical configurations of alkenes *trans*-**24a** and *trans*-**25a** were previously established in literature.[2] Comparisons between the ^{1}H NMR spectra of tetrahydrofurans **24a**, **25a**, **24b**, **25b**, and **25c** (i.e., chemical shifts and coupling constants) were used to determine the stereochemical configurations of tetrahydrofurans *trans*-**24b**, *cis*-**24b**, *trans*-**25b**, and *trans*-**25c**.

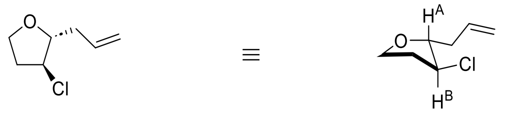

Alkene *trans*-24a (1,2-*trans*, major diastereomer):
^{1}H NMR (400 MHz, $CDCl_3$) δ 5.88–5.78 (m, 1H), 5.17–5.11 (m, 2H), 4.03–3.93 (m, 4H), 2.45–2.36 (m, 2H), 2.33–2.26 (m, 1H), 2.17–2.09 (m, 1H).

Note: H^A and H^B are not separate nor distinct within the ^{1}H NMR spectra. Rather, H^A and H^B are located within the multiplet from δ 4.03–3.93, which contains a total of 4H.

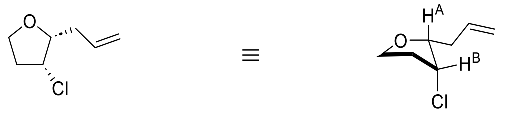

Alkene *cis*-24a (1,2-*cis*, minor diastereomer):
^{1}H NMR (400 MHz, $CDCl_3$) δ 5.88–5.78 (m, 1H), 5.22–5.09 (m, 2H), 4.43 (ddd, *J* = 4.8, 3.1, 1.2, 1H), 4.17–4.11 (m, 1H), 3.94–3.86 (m, 2H), 2.57–2.42 (m, 3H), 2.32–2.25 (m, 1H).

Note: H^B is distinct and identifiable in the ^{1}H NMR spectrum at δ 4.43 (ddd, *J* = 4.8, 3.1, 1.2, 1H). The chemical shift of H^B is located more downfield on the spectrum of 1,2-*cis* alkene *cis*-**24a** (δ 4.43) than it is in 1,2-*trans* alkene *trans*-**24a** (δ 4.03–3.93), and this generalized observation is also reported in literature.[22] Such notable characteristics in the ^{1}H NMR spectra appears indicative of a 1,2-*cis* stereochemical configuration of 1,2-substituted tetrahydrofurans.

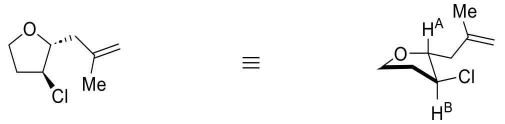

Alkene *trans*-24b (1,2-*trans*):
^{1}H NMR (400 MHz, $CDCl_3$) δ 4.86–4.80 (m, 2H), 4.15–3.94 (m, 4H), 2.46–2.37 (m, 1H), 2.32–2.21 (m, 2H), 2.17–2.10 (m, 1H), 1.79 (s, 3H).

Note: H^A and H^B are not separate nor distinct within the ^{1}H NMR spectra. Rather, H^A and H^B are located within the multiplet from δ 4.15–3.94, which contains a total of 4H. Such an observation indicates that the stereochemical configuration of alkene *trans*-**24b** is 1,2-*trans*.

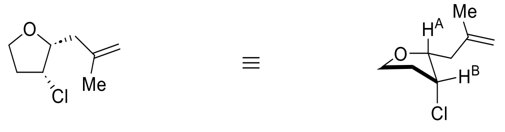

Alkene *cis*-24b (1,2-*cis*):

^{1}H NMR (400 MHz, $CDCl_3$) δ 4.86–4.84 (m, 2H), 4.44 (ddd, *J* = 4.8, 3.1, 1.2, 1H), 4.17–4.11 (m, 1H), 4.04–4.00 (m, 1H), 3.94–3.89 (m, 1H), 2.52–2.39 (m, 3H), 2.32–2.26 (m, 1H), 1.79 (s, 3H).

Note: H^B is distinct and identifiable in the ^{1}H NMR spectraum at δ 4.44 (ddd, *J* = 4.8, 3.1, 1.2, 1H). The chemical shift of H^B is located more downfield on the spectrum of 1,2-*cis* alkene *cis*-**24b** (δ 4.44) than it is in 1,2-*trans* alkene *trans*-**24b** (δ 4.15–3.94), and this generalized observation is also reported in literature.[22] Furthermore, the chemical shift and coupling constants of H^B of alkene *cis*-**24b** are very similar to the chemical shift and coupling constants of H^B of alkene *cis*-**24a**. Such an observation indicates that the stereochemical configuration of alkene *cis*-**24b** is 1,2-*cis*.

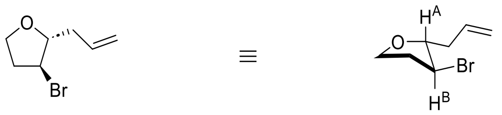

Alkene *trans*-25a (1,2-*trans*, major diastereomer):

^{1}H NMR (400 MHz, $CDCl_3$) δ 5.88–5.78 (m, 1H), 5.17–5.11 (m, 2H), 4.10 (dt, *J* = 6.8, 5.6, 1H), 4.02–3.92 (m, 3H), 2.53–2.39 (m, 2H), 2.32–2.20 (m, 2H).

Note: H^B is distinct and identifiable in the ^{1}H NMR spectrum at δ 4.10 (dt, *J* = 6.8, 5.6, 1H).

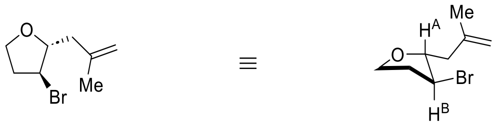

Alkene *trans*-25b (1,2-*trans*, major diastereomer):

^{1}H NMR (400 MHz, $CDCl_3$) δ 4.87–4.79 (m, 2H), 4.21 (dt, *J* = 7.7, 5.3, 1H), 4.03–3.93 (m, 3H), 2.54–2.45 (m, 1H), 2.37–2.18 (m, 3H), 1.79 (s, 3H).

Note: H^B is distinct and identifiable in the ^{1}H NMR spectrum at δ 4.21 (dt, *J* = 7.7, 5.3, 1H). The chemical shift and coupling constants of H^B of alkene *trans*-**25b** are similar to the chemical shift and coupling constants of H^B of alkene *trans*-**25a**. Such an observation indicates that the stereochemical configuration of alkene *trans*-**25b** is 1,2-*trans*.

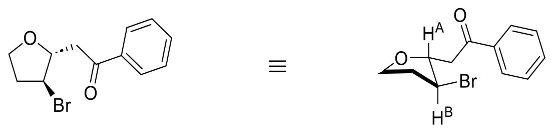

Alkene *trans*-25c (1,2-*trans*, major diastereomer):

^{1}H NMR (400 MHz, $CDCl_3$) δ 7.97–7.95 (m, 1H), 7.60–7.56 (m, 2H), 7.50–7.45 (m, 2H), 4.59 (dt, *J* = 6.7, 5.7, 1H), 4.15 (dt, *J* = 7.5, 5.7, 1H), 4.02–3.99 (m, 2H), 3.26–3.23 (m, 2H), 2.59–2.50 (m, 1H), 2.34–2.25 (m, 1H).

Note: H^B is distinct and identifiable in the ^{1}H NMR spectrum at δ 4.15 (dt, *J* = 7.5, 5.3, 1H). The chemical shift and coupling constants of H^B of alkene *trans*-**25c** are similar to the chemical shift and coupling constants of H^B of alkene *trans*-**25a**. Such an observation indicates that the stereochemical configuration of alkene *trans*-**25c** is 1,2-*trans*.

II. X-Ray Crystallographic Data

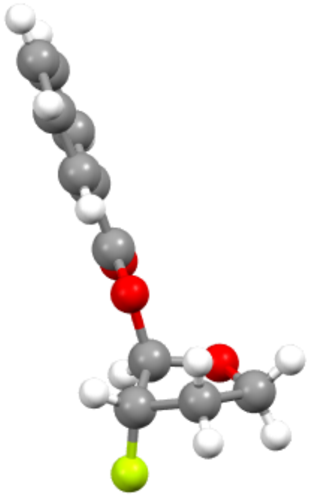

Figure S1. The molecular structure of ketone *trans*-**10** (ellipsoids set at 50% probability).

A clear colorless, needle-like specimen of $C_{11}H_{11}FO_3$, approximate dimensions 0.020 mm x 0.200 mm x 0.650 mm, was used for the X-ray crystallographic analysis. The X-ray intensity data were measured on a Bruker D8 SMART APEXII 'three-circle diffractometer' system equipped with a Incotec 'microfocus sealed X-ray tube' (MoKα, λ = 0.71073 Å) and a multilayer optics monochromator.

A total of 720 frames were collected. The total exposure time was 12.00 hours. The frames were integrated with the Bruker SAINT software package using a narrow-frame algorithm. The integration of the data using an orthorhombic unit cell yielded a total of 19375 reflections to a maximum θ angle of 28.71° (0.74 Å resolution), of which 2513 were independent (average redundancy 7.710, completeness = 98.9%, R_{int} = 5.22%, R_{sig} = 3.31%) and 2383 (94.83%) were greater than $2\sigma(F^2)$. The final cell constants of *a* = 16.7785(10) Å, *b* = 5.7894(4) Å, *c* = 20.2529(13) Å, volume = 1967.3(2) $Å^3$, are based upon the refinement of the XYZ-centroids of 9201 reflections above 20 σ(I) with 4.698° < 2θ < 57.14°. Data were corrected for absorption effects using the Multi-Scan method (SADABS). The ratio of minimum to maximum apparent transmission was 0.869. The calculated minimum and maximum transmission coefficients (based on crystal size) are 0.9290 and 0.9980.

The structure was solved and refined using the Bruker SHELXTL Software Package, using the space group P b c a, with Z = 8 for the formula unit, $C_{11}H_{11}FO_3$. The final anisotropic full-matrix least-squares refinement on F^2 with 136 variables converged at R1 = 6.76%, for the observed data and wR2 = 14.17% for all data. The goodness-of-fit was 1.249. The largest peak in the final difference electron density synthesis was 0.368 $e^-/Å^3$ and the largest hole was -0.278 $e^-/Å^3$ with an

RMS deviation of 0.065 $e^-/Å^3$. On the basis of the final model, the calculated density was 1.419 g/cm^3 and F(000), 880 e^-.

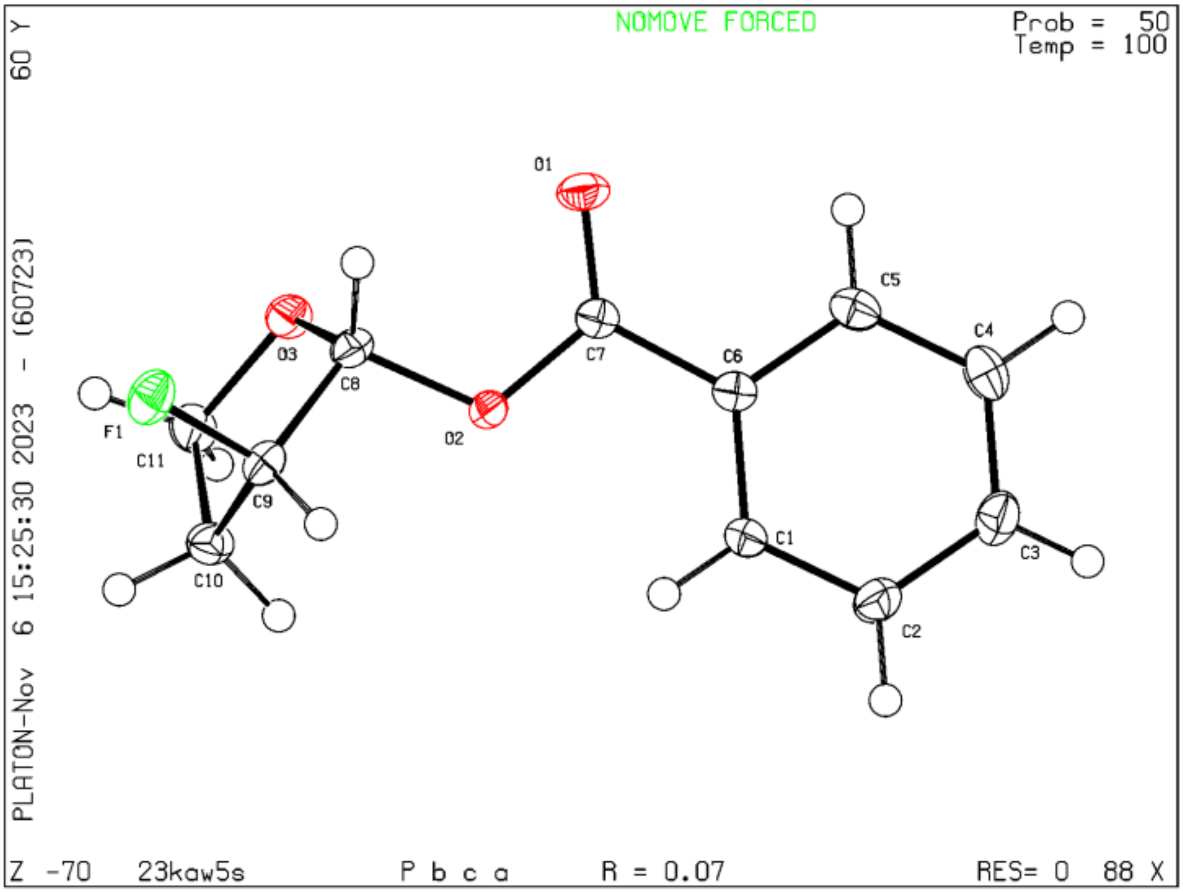

Figure S2. Thermal ellipsoid parameter figure of ketone *trans*-**10**.

Table S1. Sample and crystal data for ketone *trans*-**10**.

Identification code	23kaw5s
Chemical formula	$C_{11}H_{11}FO_3$
Formula weight	210.20 g/mol
Temperature	100(2) K
Wavelength	0.71073 Å
Crystal size	0.020 x 0.200 x 0.650 mm
Crystal habit	clear colorless needle
Crystal system	Orthorhombic

Space group	P b c a	
Unit cell dimensions	a = 16.7785(10) Å	α = 90°
	b = 5.7894(4) Å	β = 90°
	c = 20.2529(13) Å	γ = 90°
Volume	1967.3(2) Å^3	
Z	8	
Density (calculated)	1.419 g/cm^3	
Absorption coefficient	0.115 mm^{-1}	
F(000)	880	

Table S2. Data collection and structure refinement for ketone *trans*-**10**.

Diffractometer	Bruker D8 SMART APEXII ‘three-circle diffractometer’	
Radiation source	Incotec ‘microfocus sealed X-ray tube’ (MoKα, λ = 0.71073 Å)	
Theta range for data collection	2.01 to 28.71°	
Index ranges	-22<=h<=22, -7<=k<=7, -26<=l<=27	
Reflections collected	19375	
Independent reflections	2513 [R(int) = 0.0522]	
Coverage of independent reflections	98.9%	
Absorption correction	multi-scan	
Max. and min. transmission	0.9980 and 0.9290	
Structure solution technique	direct methods	
Structure solution program	SHELXT (Sheldrick 2015)	
Refinement method	Full-matrix least-squares on F^2	
Refinement program	SHELXL-2018/3 (Sheldrick, 2018)	
Function minimized	$\Sigma w(F_o^2 - F_c^2)^2$	
Data / restraints / parameters	2513 / 0 / 136	
Goodness-of-fit on F^2	1.249	
Final R indices	2383 data; I>2σ(I)	R1 = 0.0676<br>wR2 = 0.1395
	all data	R1 = 0.0716<br>wR2 = 0.1417

Weighting scheme	$w=1/[\sigma^2(F_o^2)+(0.0319P)^2+2.6422P]$<br>where $P=(F_o^2+2F_c^2)/3$
Largest diff. peak and hole	0.368 and -0.278 eÅ^{-3}
R.M.S. deviation from mean	0.0655 eÅ^{-3}

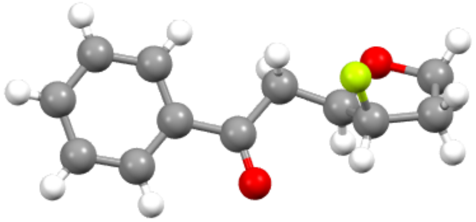

Figure S3. The molecular structure of ketone *cis*-**21b** (ellipsoids set at 50% probability).

A clear colorless, needle-like specimen of $C_{12}H_{13}FO_2$, approximate dimensions 0.110 mm x 0.150 mm x 0.450 mm, was used for the X-ray crystallographic analysis. The X-ray intensity data were measured on a Bruker D8 SMART APEXII 'three-circle diffractometer' system equipped with a Incotec 'microfocus sealed X-ray tube' (MoKα, λ = 0.71073 Å) and a multilayer optics monochromator.

A total of 1440 frames were collected. The total exposure time was 8.00 hours. The frames were integrated with the Bruker SAINT software package using a narrow-frame algorithm. The integration of the data using a monoclinic unit cell yielded a total of 14861 reflections to a maximum θ angle of 28.57° (0.74 Å resolution), of which 2534 were independent (average redundancy 5.865, completeness = 99.1%, R_{int} = 3.32%, R_{sig} = 2.39%) and 2309 (91.12%) were greater than 2σ(F^2). The final cell constants of *a* = 18.5636(8) Å, *b* = 5.4415(2) Å, *c* = 21.7444(9) Å, β = 114.155(2)°, volume = 2004.16(14) $Å^3$, are based upon the refinement of the XYZ-centroids of 7461 reflections above 20 σ(I) with 4.809° < 2θ < 57.07°. Data were corrected for absorption effects using the Multi-Scan method (SADABS). The ratio of minimum to maximum apparent transmission was 0.937. The calculated minimum and maximum transmission coefficients (based on crystal size) are 0.9540 and 0.9890.

The structure was solved and refined using the Bruker SHELXTL Software Package, using the space group C 1 2/c 1, with Z = 8 for the formula unit, $C_{12}H_{13}FO_2$. The final anisotropic full-matrix least-squares refinement on F^2 with 136 variables converged at R1 = 4.54%, for the observed data and wR2 = 11.16% for all data. The goodness-of-fit was 1.142. The largest peak in the final difference electron density synthesis was 0.561 $e^-/Å^3$ and the largest hole was -0.222 $e^-/Å^3$ with an RMS deviation of 0.080 $e^-/Å^3$. On the basis of the final model, the calculated density was 1.380 g/cm^3 and F(000), 880 e^-.

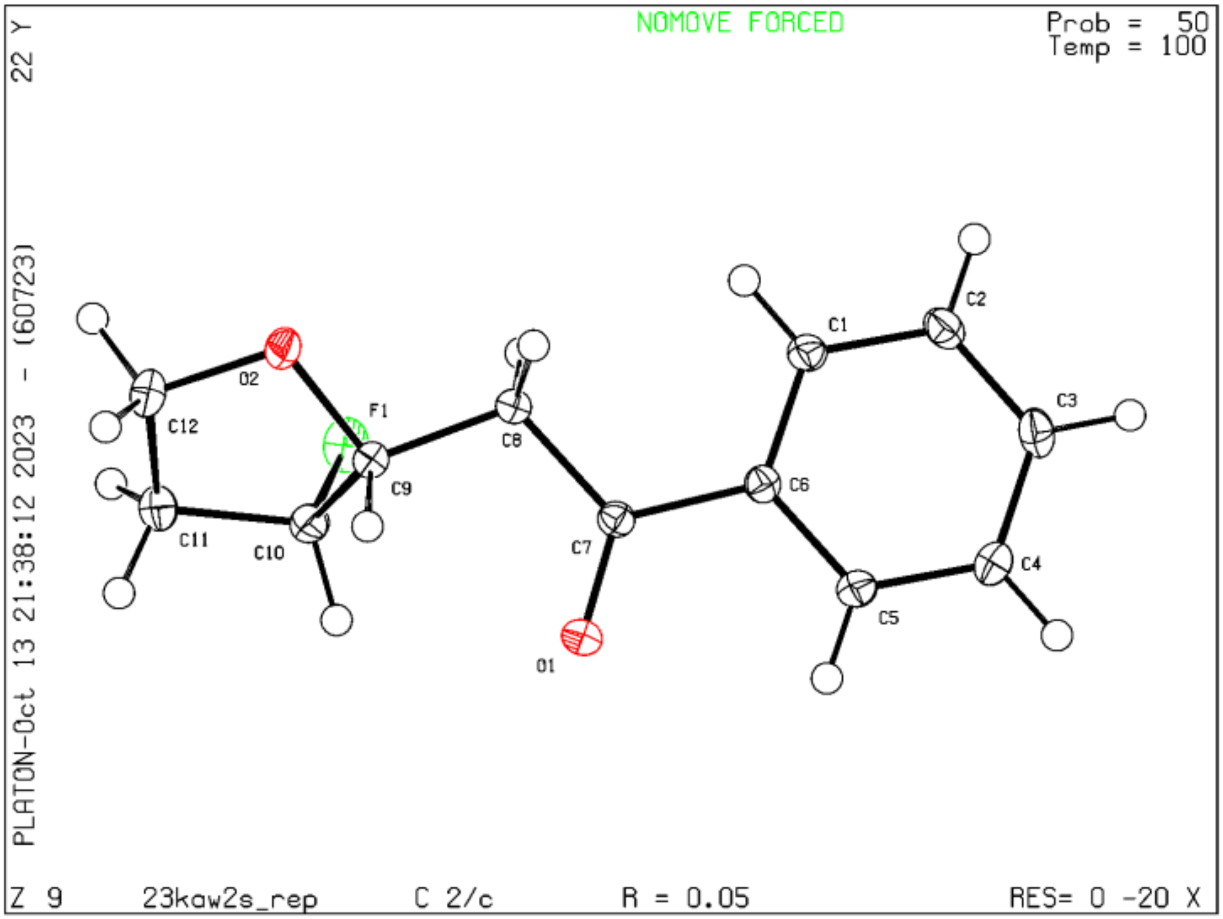

Figure S4. Thermal ellipsoid parameter figure of ketone *cis*-**21b**.

Table S3. Sample and crystal data for ketone *cis*-**21b**.

Identification code	23kaw2s	
Chemical formula	$C_{12}H_{13}FO_2$	
Formula weight	208.22 g/mol	
Temperature	100(2) K	
Wavelength	0.71073 Å	
Crystal size	0.110 x 0.150 x 0.450 mm	
Crystal habit	clear colorless plate	
Crystal system	monoclinic	
Space group	C 1 2/c 1	
Unit cell dimensions	a = 18.5636(8) Å	α = 90°
	b = 5.4415(2) Å	β = 114.155(2)°

	c = 21.7444(9) Å	γ = 90°
Volume	2004.16(14) $Å^3$	
Z	8	
Density (calculated)	1.380 g/cm^3	
Absorption coefficient	0.105 mm^{-1}	
F(000)	880	

Table S4. Data collection and structure refinement for ketone *cis*-**21b**.

Diffractometer	Bruker D8 SMART APEXII ‘three-circle diffractometer’	
Radiation source	Incotec ‘microfocus sealed X-ray tube’ (MoKα, λ = 0.71073 Å)	
Theta range for data collection	2.05 to 28.57°	
Index ranges	-24<=h<=24, -7<=k<=6, -29<=l<=29	
Reflections collected	14861	
Independent reflections	2534 [R(int) = 0.0332]	
Coverage of independent reflections	99.1%	
Absorption correction	multi-scan	
Max. and min. transmission	0.9890 and 0.9540	
Structure solution technique	direct methods	
Structure solution program	SHELXT (Sheldrick 2015)	
Refinement method	Full-matrix least-squares on F^2	
Refinement program	SHELXL-2019/1 (Sheldrick, 2019)	
Function minimized	$\Sigma w(F_o^2 - F_c^2)^2$	
Data / restraints / parameters	2534 / 0 / 136	
Goodness-of-fit on F^2	1.142	
Final R indices	2309 data; I>2σ(I)	R1 = 0.0454<br>wR2 = 0.1092
	all data	R1 = 0.0494<br>wR2 = 0.1116
Weighting scheme	$w=1/[\sigma^2(F_o^2)+(0.0449P)^2+2.2904P]$<br>where $P=(F_o^2+2F_c^2)/3$	
Largest diff. peak and hole	0.561 and -0.222 $eÅ^{-3}$	
R.M.S. deviation from mean	0.080 $eÅ^{-3}$	

III. References

(1) Otte, D. A. L.; Borchmann, D. E.; Lin, C.; Weck, M.; Woerpel, K. A. ^{13}C NMR Spectroscopy for the Quantitative Determination of Compound Ratios and Polymer End Groups. *Org. Lett.* **2014**, *16*, 1566.

(2) Demkiw, K. M.; Remmerswaal, W. A.; Hansen, T.; van der Marel, G. A.; Codée, J. D. C.; Woerpel, K. A. Halogen Atom Participation in Guiding the Stereochemical Outcomes of Acetal Substitution Reactions. *Angew. Chem., Int. Ed.* **2022**, e202209401.

(3) Demkiw, K. M.; Remmerswaal, W. A.; Hansen, T.; van der Marel, G. A.; Codee, J. D. C.; Woerpel, K. A. Halogen Atom Participation in Guiding the Stereochemical Outcomes of Acetal Substitution Reactions. *Angew. Chem. Int. Ed. Engl.* **2022**, *61*, e202209401.

(4) Durantie, E.; Bucher, C.; Gilmour, R. Fluorine-Directed β-Galactosylation: Chemical Glycosylation Development by Molecular Editing. *Chem. - Eur. J.* **2012**, *18*, 8208.

(5) Barbosa, T. M.; Viesser, R. V.; Abraham, R. J.; Rittner, R.; Tormena, C. F. Experimental and theoretical evaluation of *trans*-3-halo-2-hydroxy-tetrahydropyran conformational preferences. Beyond anomeric interaction. *RSC Adv.* **2015**, *5*, 35412.

(6) Haasnoot, C. A. G. Conformational Analysis of Six-Membered Rings in Solution: Ring Puckering Coordinates Derived from Vicinal NMR Proton–Proton Coupling Constants. *J. Am. Chem. Soc.* **1993**, *115*, 1460.

(7) Bushweller, C. H.; O'Neil, J. W. The *A*-Value of the Carbodiimido Group. Conformational Requirements of "sp^2-Hybridized" Lone Pairs. *J. Org. Chem.* **1970**, *35*, 276.

(8) Schneider, H. J.; Hoppen, V. Carbon-13 Nuclear Magnetic Resonance Substituent-Induced Shieldings and Conformational Equilibriums in Cyclohexanes. *J. Org. Chem.* **1978**, *43*, 3866.

(9) Bartolo, N. D.; Demkiw, K. M.; Valentín, E. M.; Hu, C. T.; Arabi, A. A.; Woerpel, K. A. Diastereoselective Additions of Allylmagnesium Reagents to α-Substituted Ketones When Stereochemical Models Cannot Be Used. *J. Org. Chem.* **2021**, *86*, 7203.

(10) Lis, E. C., Jr.; Salomon, R. J.; Sabat, M.; Myers, W. H.; Harman, W. D. Synthesis of 1-Oxadecalins from Anisole Promoted by Tungsten. *J. Am. Chem. Soc.* **2008**, *130*, 12472.

(11) Kini, G. D.; Petrie, C. R.; Hennen, W. J.; Dalley, N. K.; Wilson, B. E.; Robins, R. K. Improved and large-scale synthesis of certain glycosyl cyanides. Synthesis of 2,5-anhydro-5-thio-D-allononitrile. *Carbohydr. Res.* **1987**, *159*, 81.

(12) Jiang, X.; García-Fortanet, J.; De Brabander, J. K. Synthesis and Complete Stereochemical Assignment of Psymberin/Irciniastatin A. *J. Am. Chem. Soc.* **2005**, *127*, 11254.

(13) Källström, S.; Jagt, R. B. C.; Sillanpää, R.; Feringa, B. L.; Minnaard, A. J.; Leino, R. Highly Enantio- and Diastereoselective One-Pot Reactions in Aqueous Media: Combined Asymmetric Rh-Catalyzed Conjugate Addition/Metal-Mediated Allylation. *Eur. J. Org. Chem.* **2006**, *2006*, 3826.

(14) Mehta, G.; Likhite, N. S. Synthetic studies towards bioactive frondosins: rapid framework access and diversity creation. *Tetrahedron Lett.* **2009**, *50*, 5263.

(15) Reddy, C.; Babu, S. A.; Aslam, N. A.; Rajkumar, V. Construction of Functionalized Carbocycles Having Contiguous Tertiary Carbinol and All-Carbon Stereogenic Centers. *Eur. J. Org. Chem.* **2013**, 2362.

(16) Shenoy, S. R.; Smith, D. M.; Woerpel, K. A. Nucleophilic Additions of Trimethylsilyl Cyanide to Cyclic Oxocarbenium Ions: Evidence for the Loss of Stereoselectivity at the Limits of Diffusion Control. *J. Am. Chem. Soc.* **2006**, *128*, 8671.

(17) Somsák, L.; Szabó, M. Estimation of the Anomeric Effect of the Cyano Group. *J. Carbohydr. Chem.* **1990**, *9*, 755.

(18) Demkiw, K. M.; Hu, C. T.; Woerpel, K. A. Hyperconjugative Interactions of the Carbon–Halogen Bond that Influence the Geometry of Cyclic α-Haloacetals. *J. Org. Chem.* **2022**, *87*, 5315.

(19) Crombie, L.; Wyvill, R. D. β-Halogeno Ether Synthesis of Olefinic Alcohols: Stereochemistry and Conformation of 2-Substituted 3-Halogenotetrahydro-pyran and -furan Precursors. *J. Chem. Soc., Perkin Trans. 1* **1985**, 1971.

(20) Gharpure, S. J.; Niranjana, P.; Porwal, S. K. Stereoselective synthesis of linear oxa-triquinanes and oxa-diquinanes via Lewis acid mediated nucleophilic addition to oxonium ions: study of nucleophile-dependent selectivity. *ARKIVOC* **2023**, *2022*, 220.

(21) Niranjana, P.; Pati, A.; Porwal, S. K.; Ramkumar, V.; Gharpure, S. J.; Chand, D. K. Coordination polymers *via* self-assembly of silver(I) and *cis*-bis-nitrile-oxa-bowl derivatives. *CrystEngComm* **2013**, *15*, 9623.

(22) Pankiewicz, K. W.; Watanabe, K. A.; Takayanagi, H.; Itoh, T.; Ogura, H. Synthesis of 2′-Deoxy-2′-substituted- and 5′-Deoxy-5′-substituted-ψ-uridine Derivatives. Crystalline and Molecular Structure of 2′-Chloro-2′-deoxy-1,3-dimethyl-ψ-uridine. Studies Directed Toward the Synthesis of 2′-Deoxy-2′-substituted-*arabino*-Nucleosides. *J. Heterocycl. Chem.* **1985**, *22*, 1703.

IV. Selected ^{1}H, ^{13}C{^{1}H}, ^{19}F{^{1}H}, and HSQC NMR Spectra

The following pages contain ^{1}H, ^{13}C{^{1}H}, ^{19}F{^{1}H} NMR, and HSQC spectra for all new compounds. Unpurified ^{1}H, ^{13}C{^{1}H}, or ^{19}F{^{1}H} NMR spectra are also included if they were used to calculate diastereomeric ratios or product ratios in competition experiments. There are no resonances with chemical shifts beyond the shown area of the spectra.

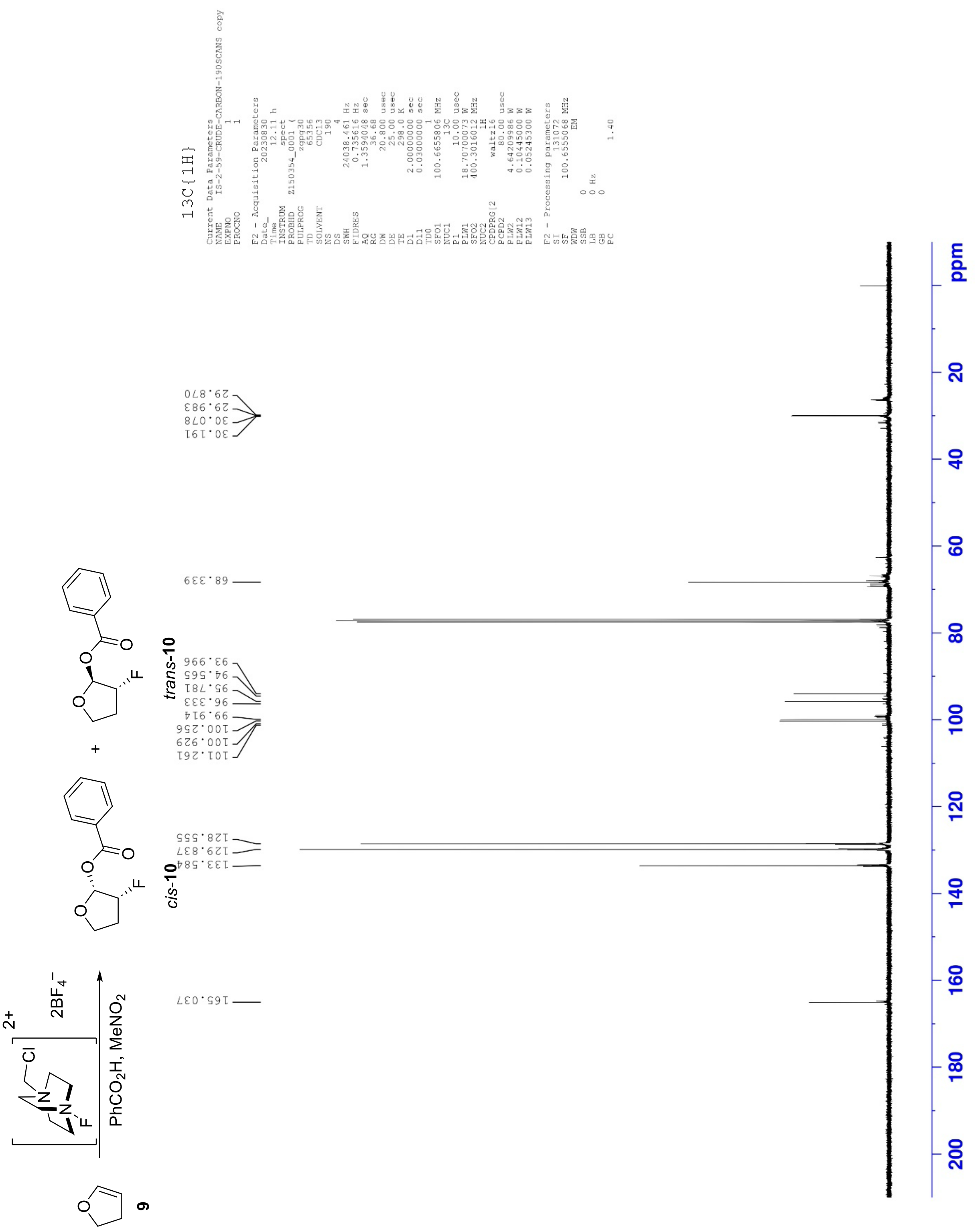
9
2BF4−
PhCO2H, MeNO2
cis-10
trans-10
13C{1H}
29.870
29.983
30.078
30.191
68.339
93.996
94.565
95.781
96.333
99.914
100.256
100.929
101.261
128.555
129.837
133.584
165.037
ppm

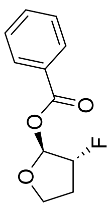

trans-**10**

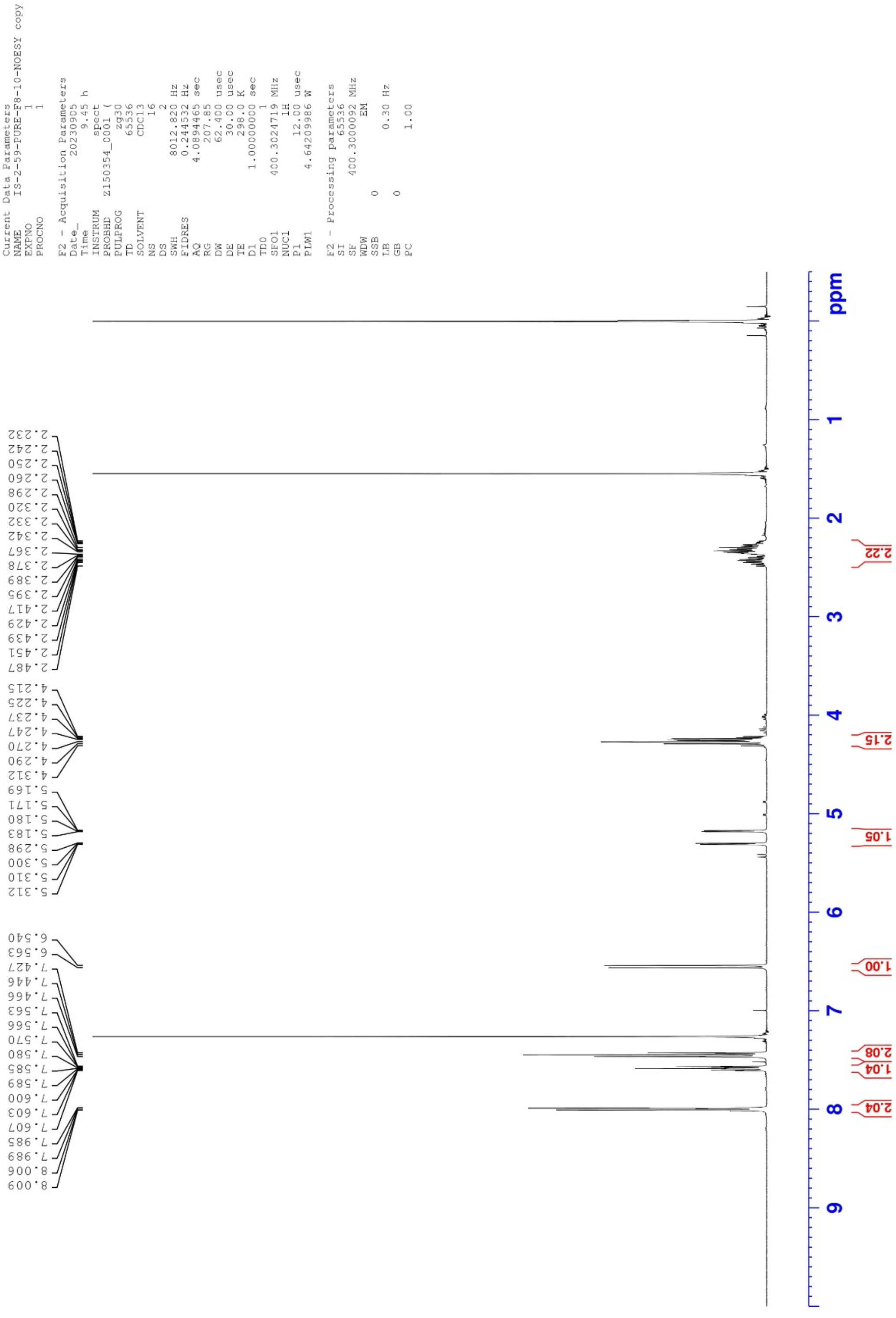

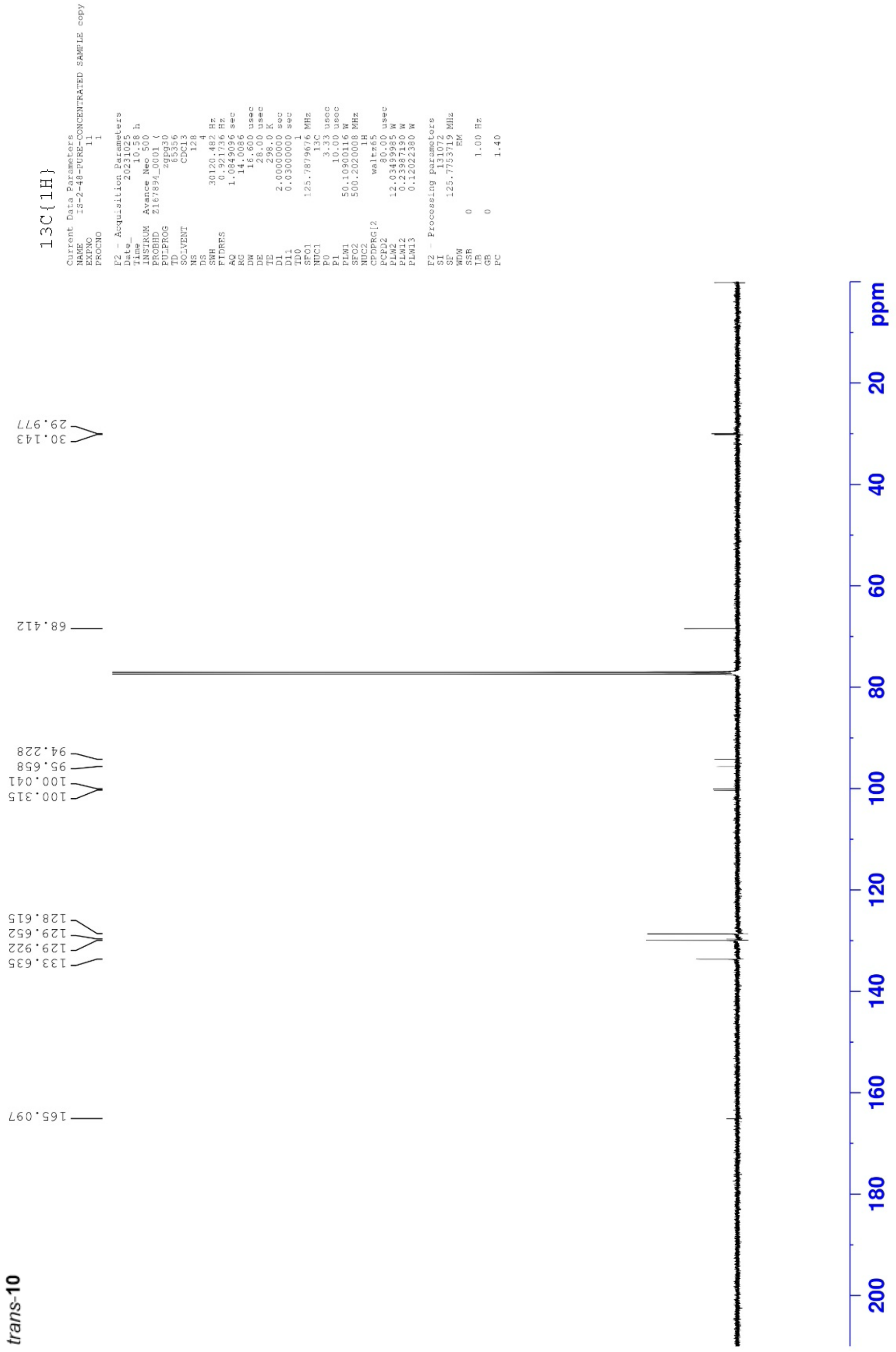
13C{1H}
29.977
30.143
68.412
94.228
95.658
100.041
100.315
128.615
129.652
129.922
133.635
165.097
ppm

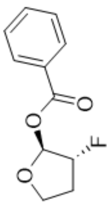

trans-**10**

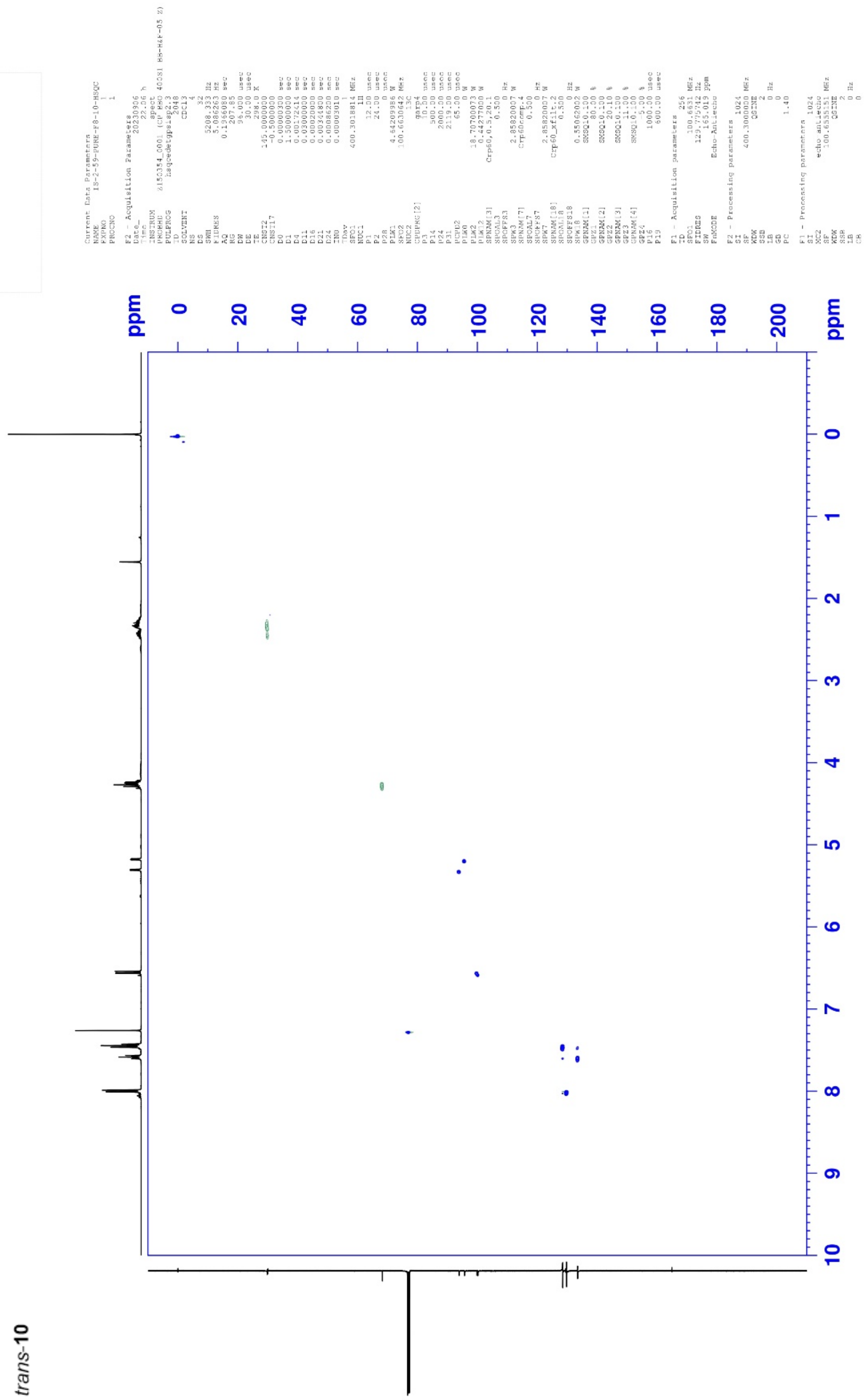

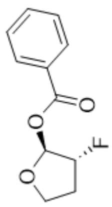

trans-**10**

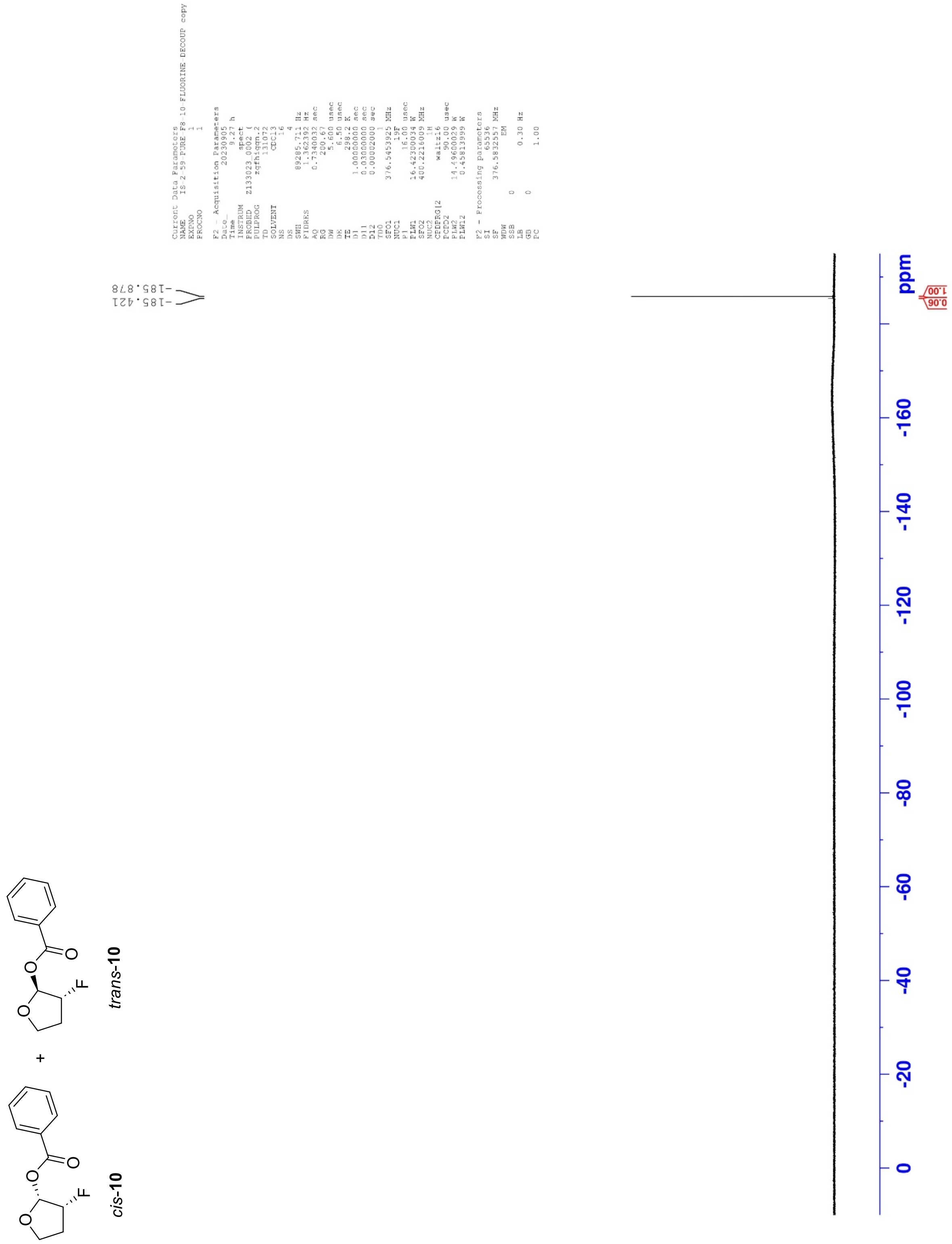
-185.878
-185.421
ppm
1.00
0.06
-160
-140
-120
-100
-80
-60
-40
-20
0
cis-10
+
trans-10
O
F

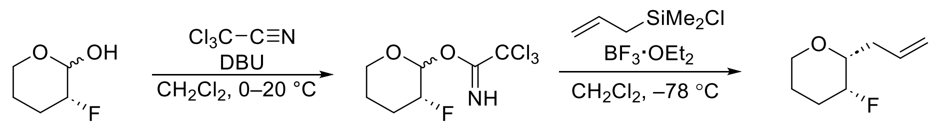

5 **6** *cis*-**13a**

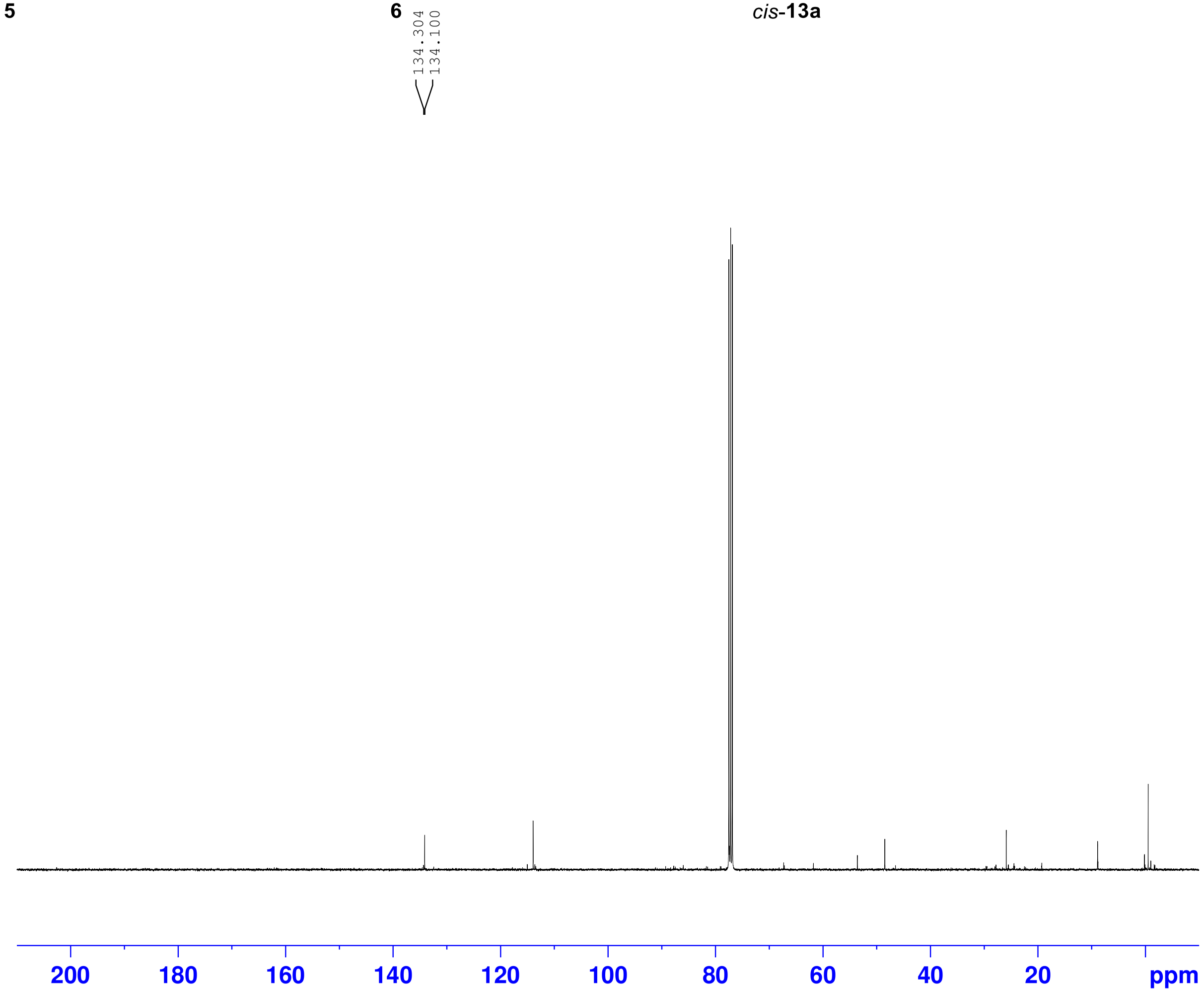


```
13C{1H}

Current Data Parameters
NAME             kmd-6-165
EXPNO                    3
PROCNO                   1

F2 - Acquisition Parameters
Date_             20220221
Time                 16.15 h
INSTRUM              spect
PROBHD    Z150354_0001 (
PULPROG            zgpg30
TD                  65356
SOLVENT             CDCl3
NS                    256
DS                      4
SWH             24038.461 Hz
FIDRES           0.735616 Hz
AQ              1.3594048 sec
RG                  29.21
DW                 20.800 usec
DE                  25.00 usec
TE                  298.0 K
D1             2.00000000 sec
D11            0.03000000 sec
TD0                     1
SFO1         100.6655806 MHz
NUC1                  13C
P1                  10.00 usec
PLW1          17.99699974 W
SFO2         400.3016012 MHz
NUC2                   1H
CPDPRG[2          waltz16
PCPD2               80.00 usec
PLW2           4.64209986 W
PLW12          0.10445000 W
PLW13          0.05245300 W

F2 - Processing parameters
SI                  32768
SF           100.6555016 MHz
WDW                    EM
SSB       0
LB                   1.00 Hz
GB        0
PC                   1.40
```

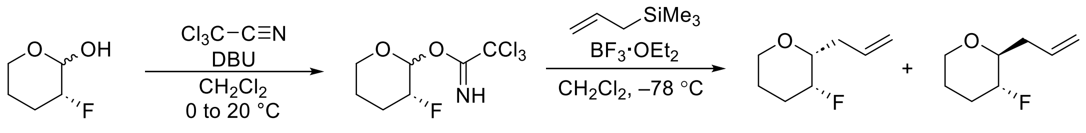

5 **6** *cis*-**13a** *trans*-**13a**

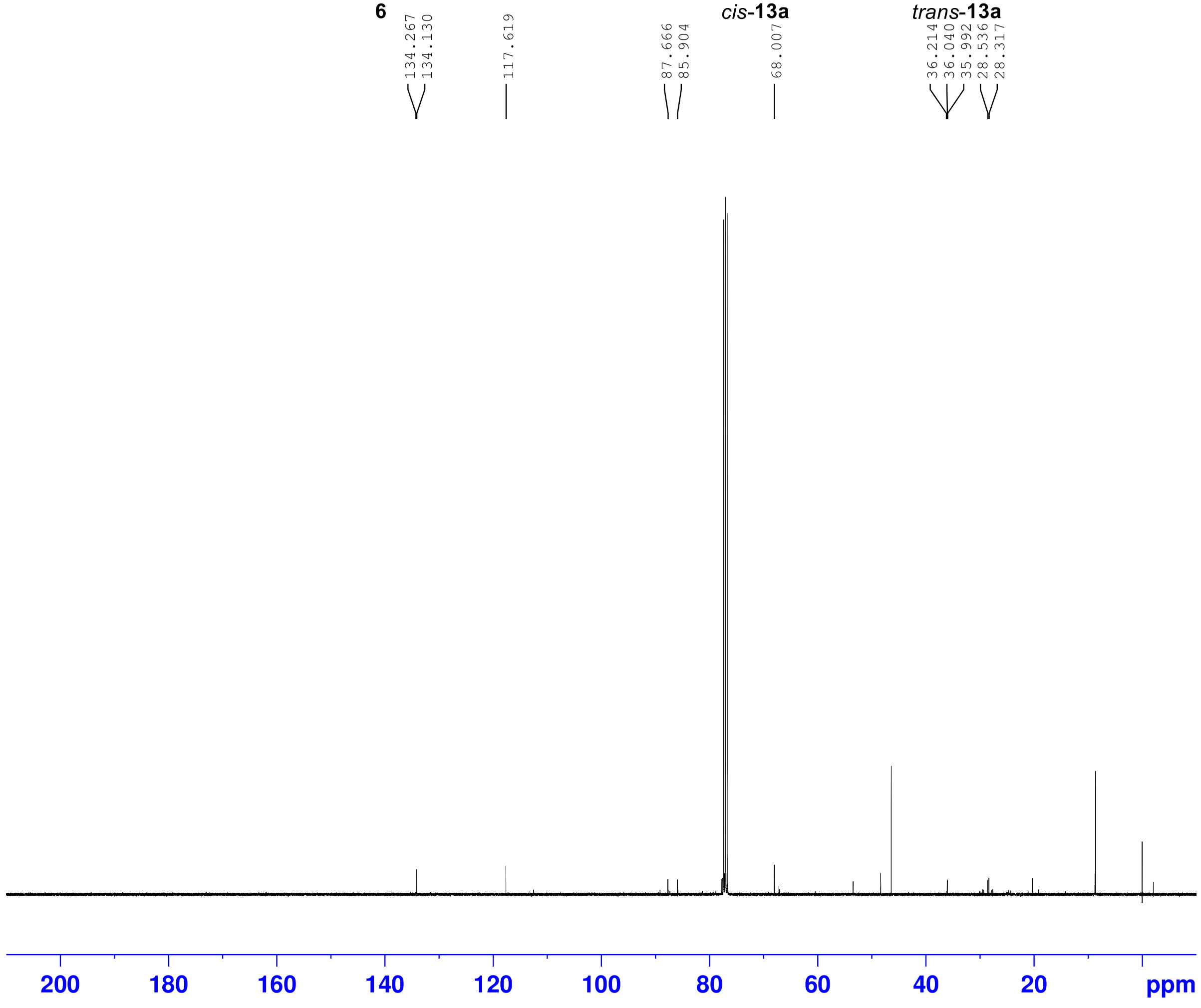


```
13C{1H}
Current Data Parameters
NAME             kmd-6-168
EXPNO                    4
PROCNO                   1

F2 - Acquisition Parameters
Date_             20220225
Time                  4.03 h
INSTRUM              spect
PROBHD    Z150354_0001 (
PULPROG            zgpg30
TD                   65356
SOLVENT              CDCl3
NS                    1024
DS                       4
SWH              24038.461 Hz
FIDRES            0.735616 Hz
AQ               1.3594048 sec
RG                   25.77
DW                  20.800 usec
DE                   25.00 usec
TE                   298.0 K
D1              2.00000000 sec
D11             0.03000000 sec
TD0                      1
SFO1           100.6655806 MHz
NUC1                   13C
P1                   10.00 usec
PLW1           17.99699974 W
SFO2           400.3016012 MHz
NUC2                    1H
CPDPRG[2           waltz16
PCPD2                80.00 usec
PLW2            4.64209986 W
PLW12           0.10445000 W
PLW13           0.05245300 W

F2 - Processing parameters
SI                  131072
SF             100.6555154 MHz
WDW                     EM
SSB        0
LB         0 Hz
GB         0
PC                    1.40
```

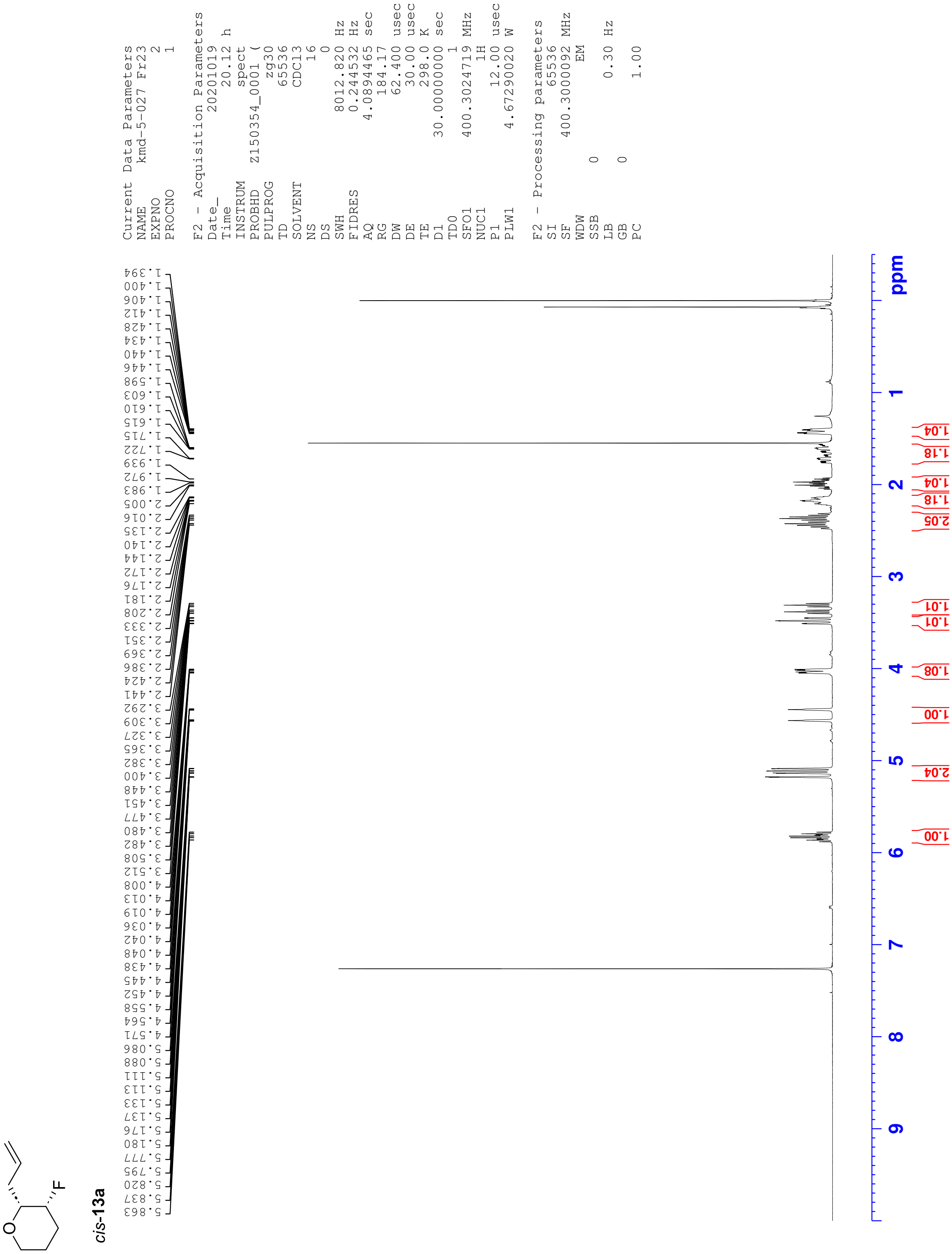
F
cis-13a
1.394
1.400
1.406
1.412
1.428
1.434
1.440
1.446
1.598
1.603
1.610
1.615
1.715
1.722
1.939
1.972
1.983
2.005
2.016
2.135
2.140
2.144
2.172
2.176
2.181
2.208
2.333
2.351
2.369
2.386
2.424
2.441
3.292
3.309
3.327
3.365
3.382
3.400
3.448
3.451
3.477
3.480
3.482
3.508
3.512
4.008
4.013
4.019
4.036
4.042
4.048
4.438
4.445
4.452
4.558
4.564
4.571
5.086
5.088
5.111
5.113
5.133
5.137
5.176
5.180
5.777
5.795
5.820
5.837
5.863
Current Data Parameters
NAME kmd-5-027 Fr23
EXPNO 2
PROCNO 1
F2 - Acquisition Parameters
Date_ 20201019
Time 20.12 h
INSTRUM spect
PROBHD Z150354_0001 (
PULPROG zg30
TD 65536
SOLVENT CDCl3
NS 16
DS 0
SWH 8012.820 Hz
FIDRES 0.244532 Hz
AQ 4.0894465 sec
RG 184.17
DW 62.400 usec
DE 30.00 usec
TE 298.0 K
D1 30.00000000 sec
TD0 1
SFO1 400.3024719 MHz
NUC1 1H
P1 12.00 usec
PLW1 4.67290020 W
F2 - Processing parameters
SI 65536
SF 400.3000092 MHz
WDW EM
SSB 0
LB 0.30 Hz
GB 0
PC 1.00
1.04
1.18
1.04
1.18
2.05
1.01
1.01
1.08
1.00
2.04
1.00
1
2
3
4
5
6
7
8
9
ppm

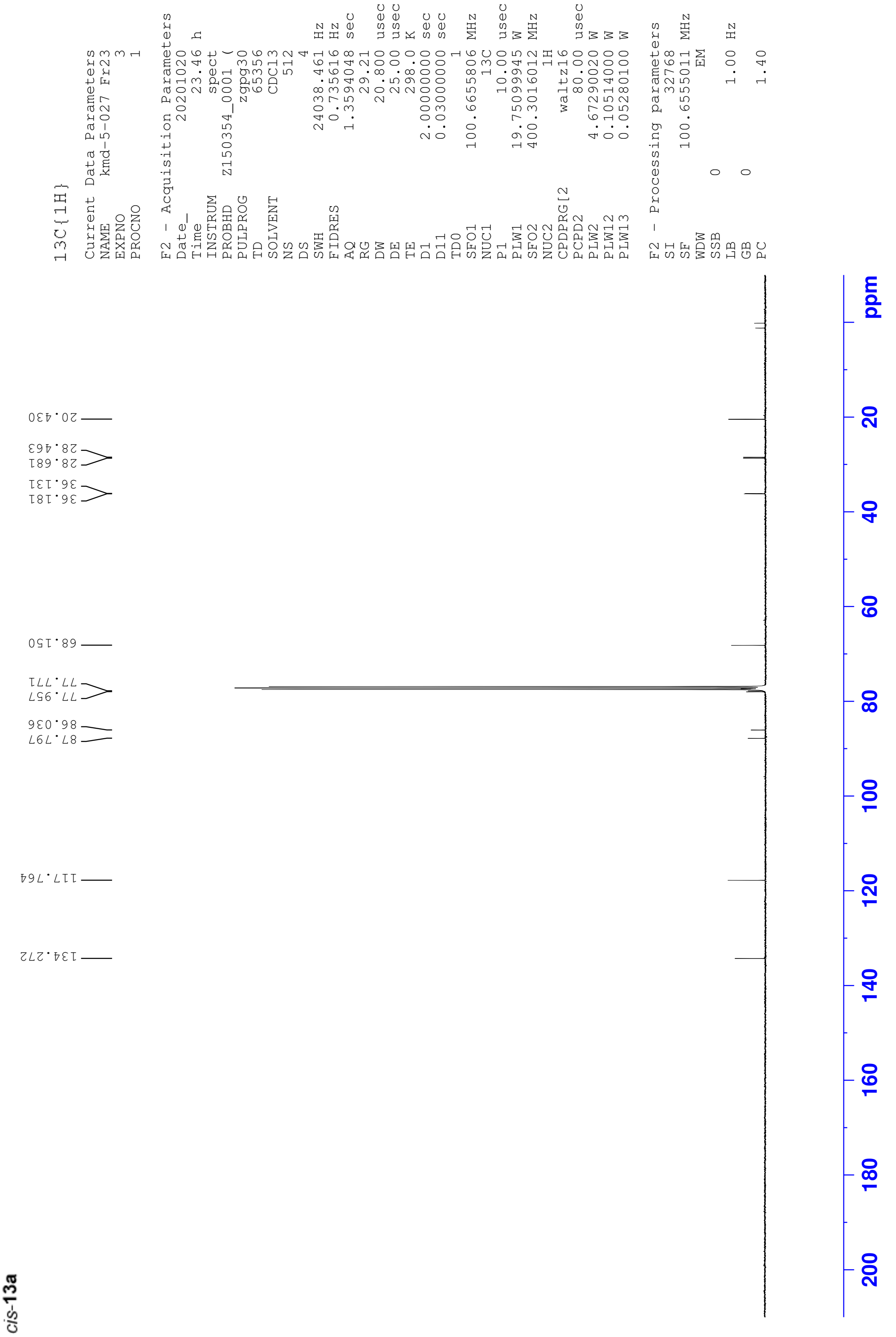
13C{1H}
Current Data Parameters
NAME kmd-5-027 Fr23
EXPNO 3
PROCNO 1
F2 - Acquisition Parameters
Date_ 20201020
Time 23.46 h
INSTRUM spect
PROBHD Z150354_0001 (
PULPROG zgpg30
TD 65356
SOLVENT CDCl3
NS 512
DS 4
SWH 24038.461 Hz
FIDRES 0.735616 Hz
AQ 1.3594048 sec
RG 29.21
DW 20.800 usec
DE 25.00 usec
TE 298.0 K
D1 2.00000000 sec
D11 0.03000000 sec
TD0 1
SFO1 100.6655806 MHz
NUC1 13C
P1 10.00 usec
PLW1 19.75099945 W
SFO2 400.3016012 MHz
NUC2 1H
CPDPRG[2 waltz16
PCPD2 80.00 usec
PLW2 4.67290020 W
PLW12 0.10514000 W
PLW13 0.05280100 W
F2 - Processing parameters
SI 32768
SF 100.6555011 MHz
WDW EM
SSB 0
LB 1.00 Hz
GB 0
PC 1.40
20.430
28.463
28.681
36.131
36.181
68.150
77.771
77.957
86.036
87.797
117.764
134.272
ppm
20
40
60
80
100
120
140
160
180
200

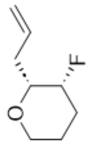
O
F

cis-**13a**

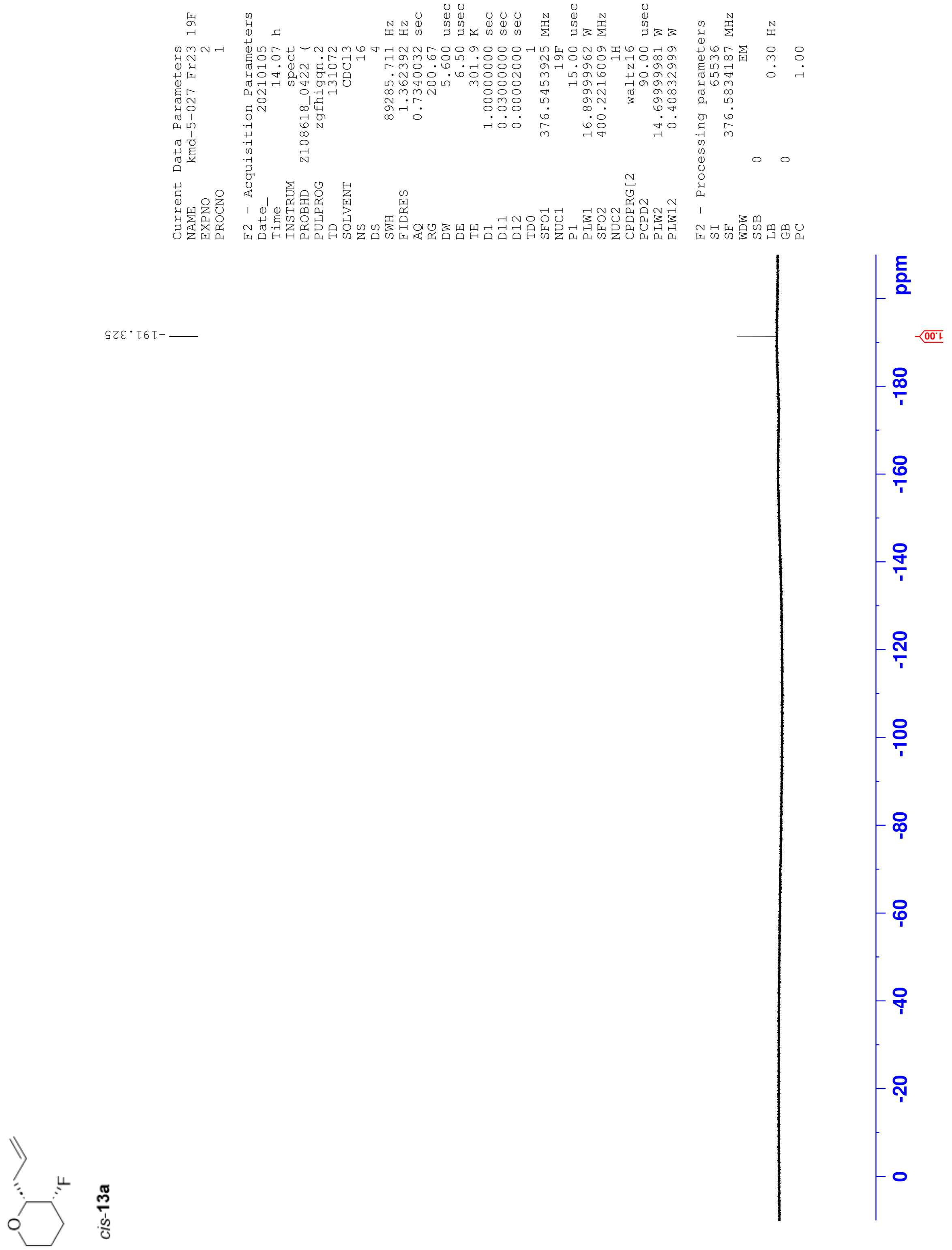
Current Data Parameters
NAME kmd-5-027 Fr23 19F
EXPNO 2
PROCNO 1
F2 - Acquisition Parameters
Date_ 20210105
Time_ 14.07 h
INSTRUM spect
PROBHD Z108618_0422 (
PULPROG zgfhigqn.2
TD 131072
SOLVENT CDCl3
NS 16
DS 4
SWH 89285.711 Hz
FIDRES 1.362392 Hz
AQ 0.7340032 sec
RG 200.67
DW 5.600 usec
DE 6.50 usec
TE 301.9 K
D1 1.00000000 sec
D11 0.03000000 sec
D12 0.00002000 sec
TD0 1
SFO1 376.5453925 MHz
NUC1 19F
P1 15.00 usec
PLW1 16.89999962 W
SFO2 400.2216009 MHz
NUC2 1H
CPDPRG[2 waltz16
PCPD2 90.00 usec
PLW2 14.69999981 W
PLW12 0.40832999 W
F2 - Processing parameters
SI 65536
SF 376.5834187 MHz
WDW EM
SSB 0
LB 0.30 Hz
GB 0
PC 1.00
-191.325
1.00
ppm
-180
-160
-140
-120
-100
-80
-60
-40
-20
0
O
F
cis-13a

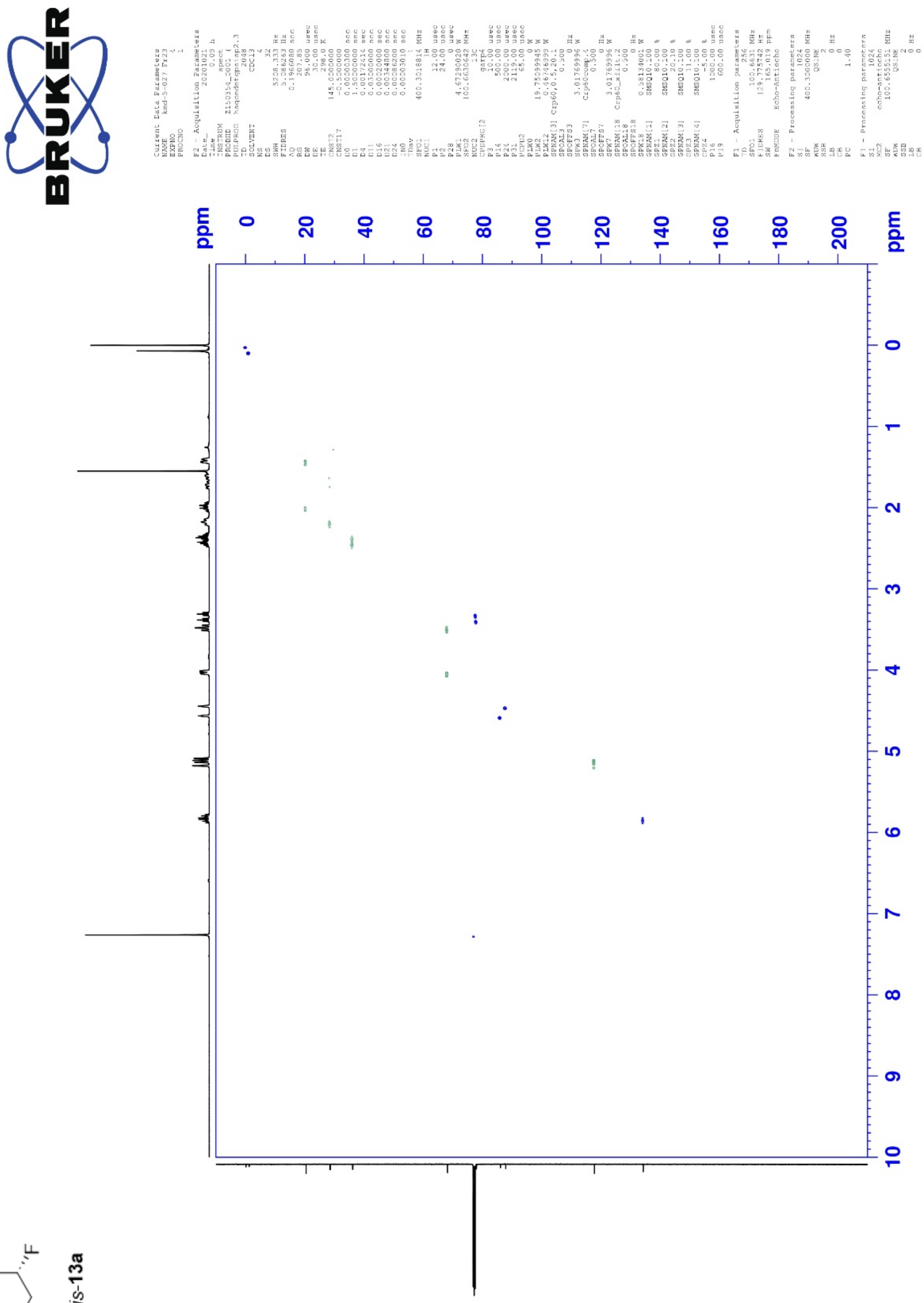

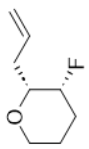

cis-**13a**

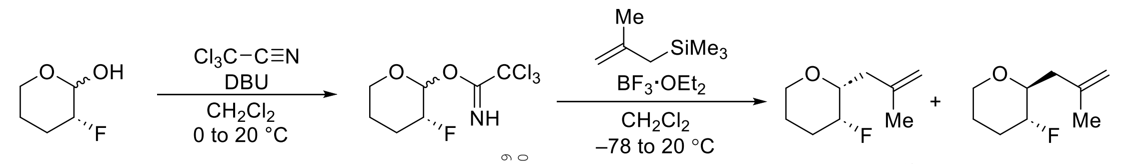

5 **6** *cis*-**13b** *trans*-**13b**

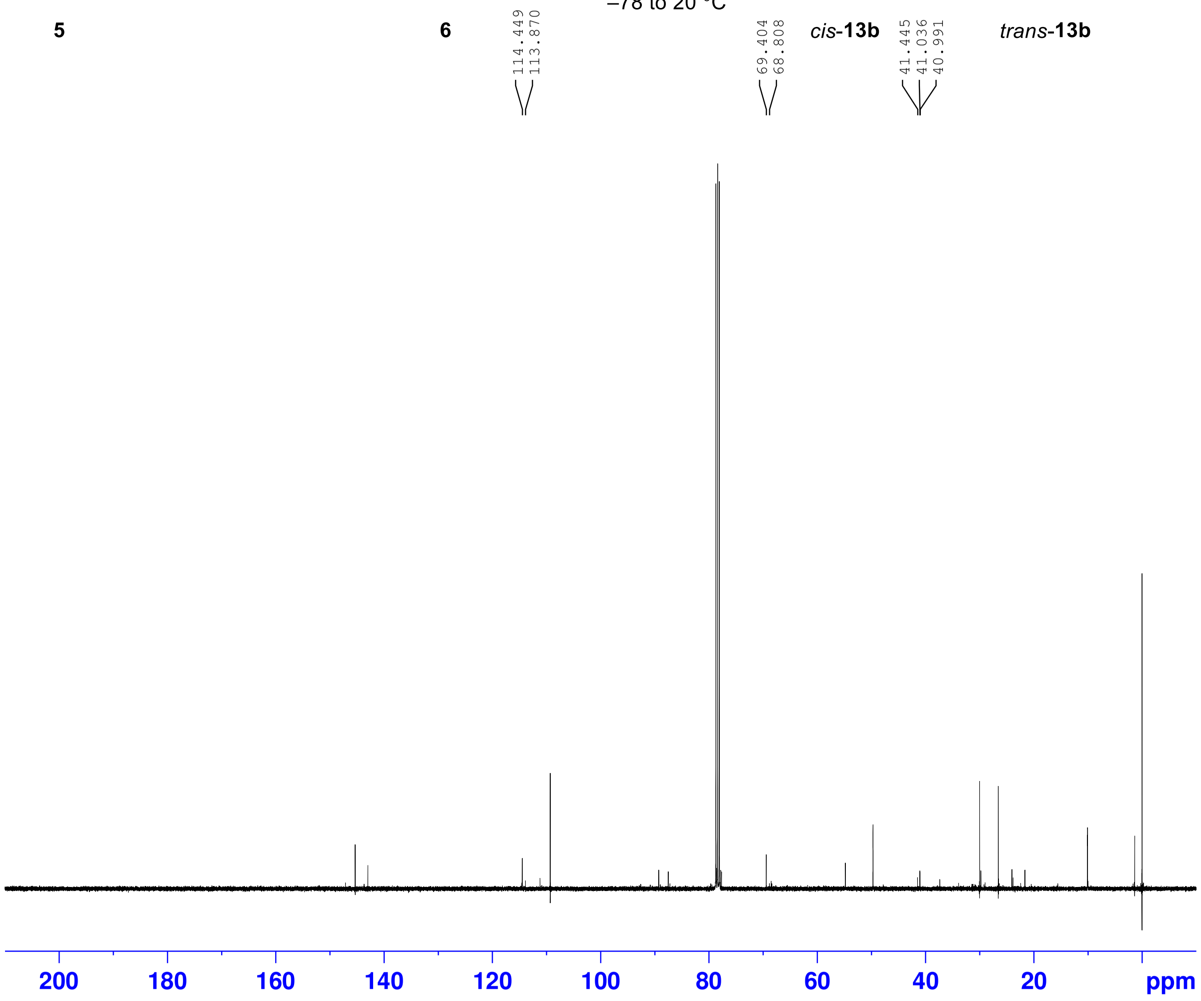


```
13C{1H}

Current Data Parameters
NAME            kmd-6-166
EXPNO                   3
PROCNO                  1

F2 - Acquisition Parameters
Date_            20220221
Time                14.43 h
INSTRUM             spect
PROBHD   Z150354_0001 (
PULPROG           zgpg30
TD                 65356
SOLVENT            CDCl3
NS                   256
DS                     4
SWH            24038.461 Hz
FIDRES          0.735616 Hz
AQ             1.3594048 sec
RG                 51.78
DW                20.800 usec
DE                 25.00 usec
TE                 298.0 K
D1            2.00000000 sec
D11           0.03000000 sec
TD0                    1
SFO1         100.6655806 MHz
NUC1                 13C
P1                 10.00 usec
PLW1         17.99699974 W
SFO2         400.3016012 MHz
NUC2                  1H
CPDPRG[2         waltz16
PCPD2              80.00 usec
PLW2          4.64209986 W
PLW12         0.10445000 W
PLW13         0.05245300 W

F2 - Processing parameters
SI                131072
SF           100.6553794 MHz
WDW                   EM
SSB       0
LB        0 Hz
GB        0
PC                  1.40
```

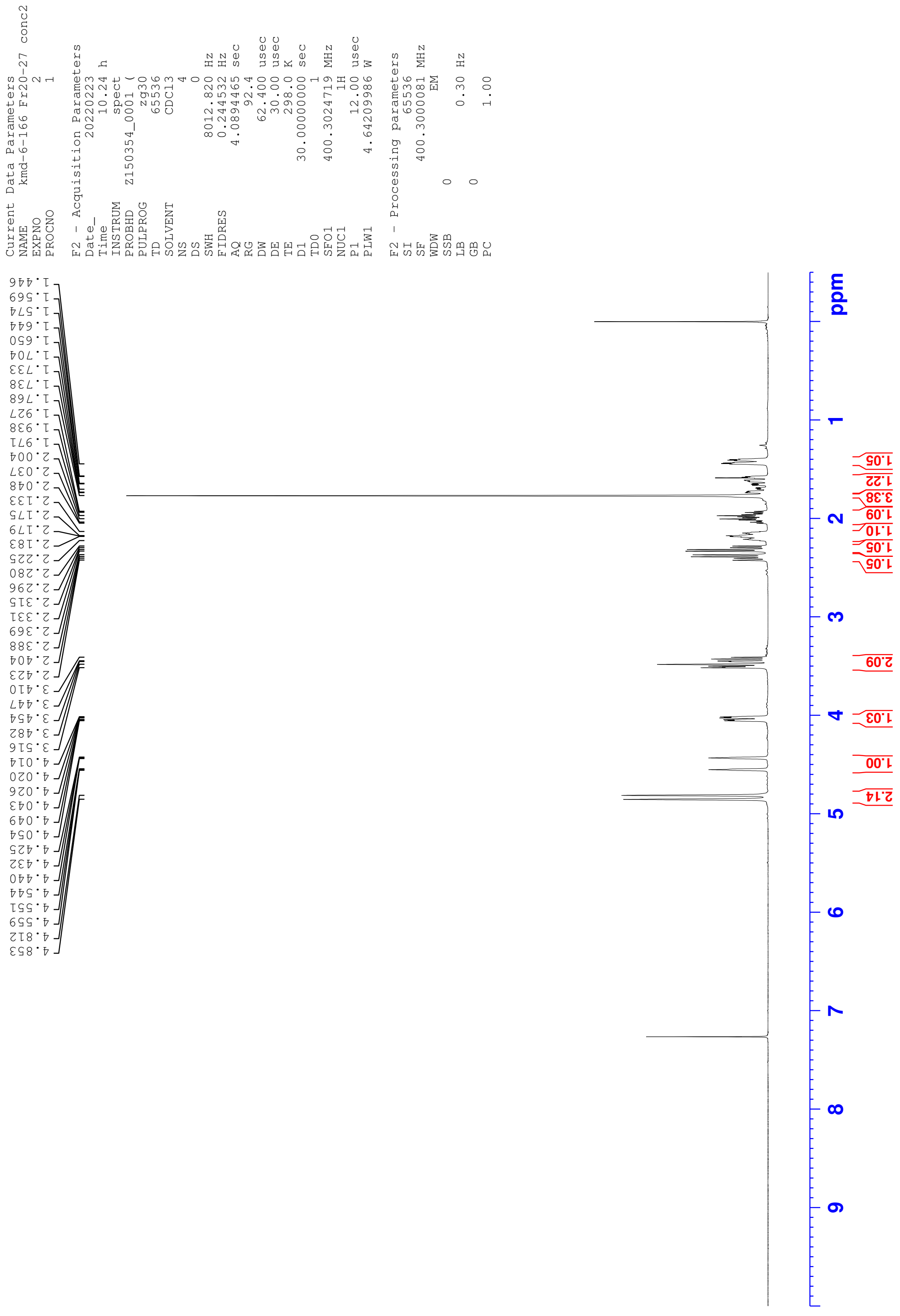
Current Data Parameters
NAME kmd-6-166 Fr20-27 conc2
EXPNO 2
PROCNO 1
F2 - Acquisition Parameters
Date_ 20220223
Time 10.24 h
INSTRUM spect
PROBHD Z150354_0001 (
PULPROG zg30
TD 65536
SOLVENT CDCl3
NS 4
DS 0
SWH 8012.820 Hz
FIDRES 0.244532 Hz
AQ 4.0894465 sec
RG 92.4
DW 62.400 usec
DE 30.00 usec
TE 298.0 K
D1 30.00000000 sec
TD0 1
SFO1 400.3024719 MHz
NUC1 1H
P1 12.00 usec
PLW1 4.64209986 W
F2 - Processing parameters
SI 65536
SF 400.3000081 MHz
WDW EM
SSB 0
LB 0.30 Hz
GB 0
PC 1.00
1.446
1.569
1.574
1.644
1.650
1.704
1.733
1.738
1.768
1.927
1.938
1.971
2.004
2.037
2.048
2.133
2.175
2.179
2.183
2.225
2.280
2.296
2.315
2.331
2.369
2.388
2.404
2.423
3.410
3.447
3.454
3.482
3.516
4.014
4.020
4.026
4.043
4.049
4.054
4.425
4.432
4.440
4.544
4.551
4.559
4.812
4.853
1.05
1.22
3.38
1.09
1.10
1.05
1.05
2.09
1.03
1.00
2.14
ppm
1
2
3
4
5
6
7
8
9

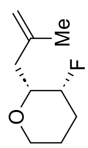
O
F
Me

cis-**13b**

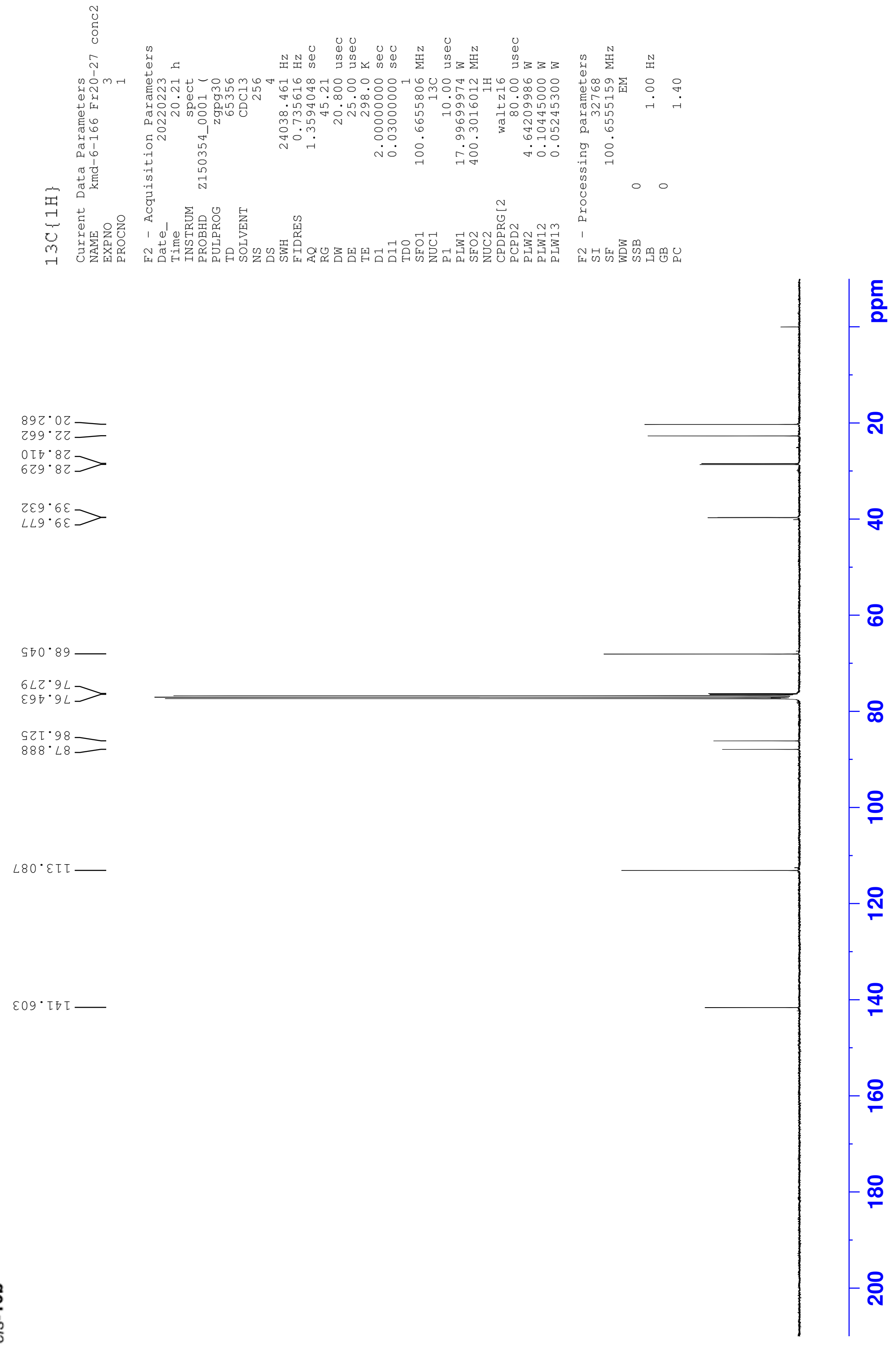
13C{1H}
Current Data Parameters
NAME kmd-6-166 Fr20-27 conc2
EXPNO 3
PROCNO 1
F2 - Acquisition Parameters
Date_ 20220223
Time 20.21 h
INSTRUM spect
PROBHD Z150354_0001 (
PULPROG zgpg30
TD 65536
SOLVENT CDCl3
NS 256
DS 4
SWH 24038.461 Hz
FIDRES 0.735616 Hz
AQ 1.3594048 sec
RG 45.21
DW 20.800 usec
DE 25.00 usec
TE 298.0 K
D1 2.00000000 sec
D11 0.03000000 sec
TD0 1
SFO1 100.6655806 MHz
NUC1 13C
P1 10.00 usec
PLW1 17.99699974 W
SFO2 400.3016012 MHz
NUC2 1H
CPDPRG[2 waltz16
PCPD2 80.00 usec
PLW2 4.64209986 W
PLW12 0.10445000 W
PLW13 0.05245300 W
F2 - Processing parameters
SI 32768
SF 100.6555159 MHz
WDW EM
SSB 0
LB 1.00 Hz
GB 0
PC 1.40
20.268
22.662
28.410
28.629
39.632
39.677
68.045
76.279
76.463
86.125
87.888
113.087
141.603
ppm
20
40
60
80
100
120
140
160
180
200

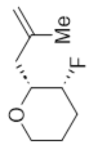
O
F
Me

cis-**13b**

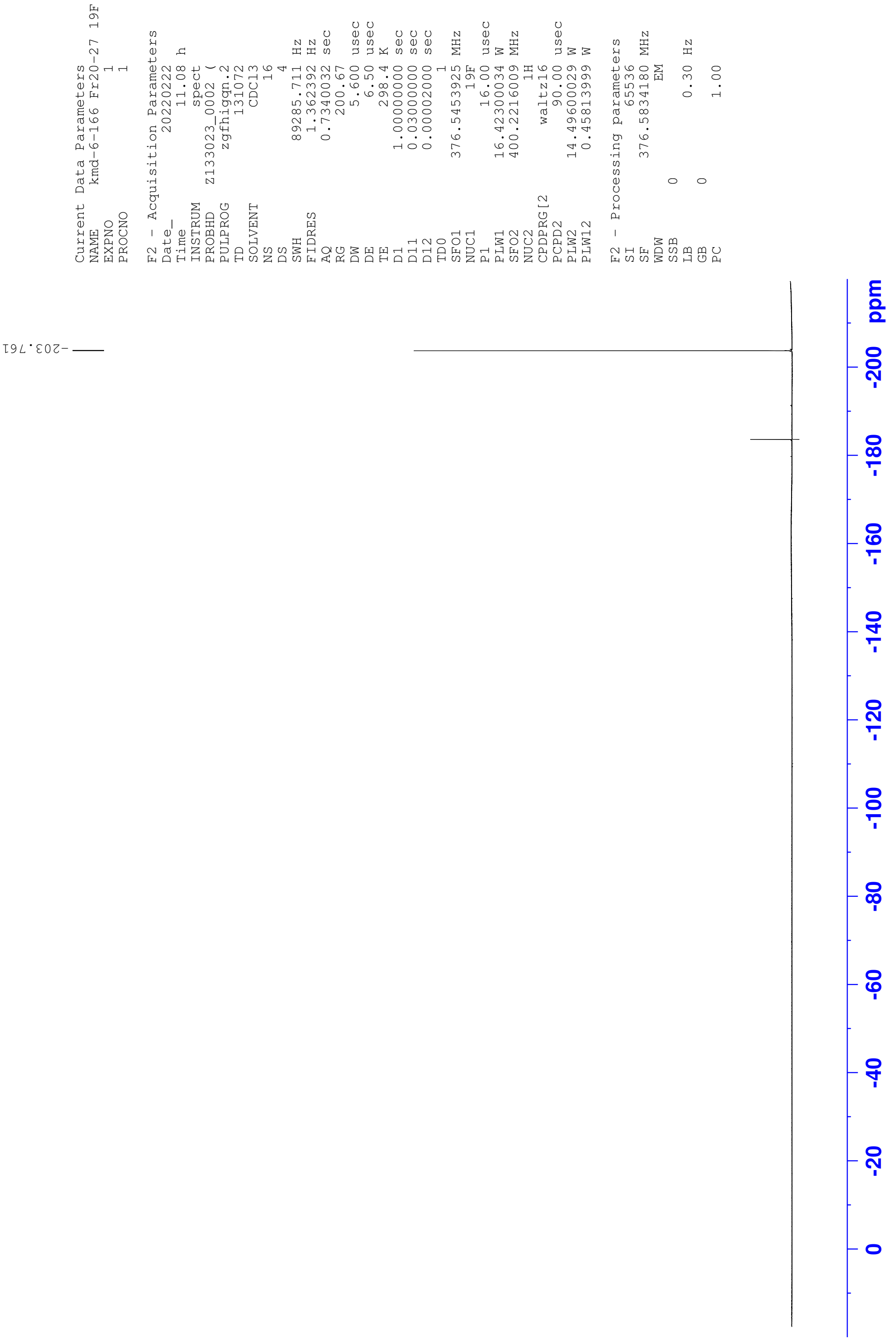
Current Data Parameters
NAME kmd-6-166 Fr20-27 19F
EXPNO 1
PROCNO 1
F2 - Acquisition Parameters
Date_ 20220222
Time 11.08 h
INSTRUM spect
PROBHD Z133023_0002 (
PULPROG zgfhigqn.2
TD 131072
SOLVENT CDCl3
NS 16
DS 4
SWH 89285.711 Hz
FIDRES 1.362392 Hz
AQ 0.7340032 sec
RG 200.67
DW 5.600 usec
DE 6.50 usec
TE 298.4 K
D1 1.00000000 sec
D11 0.03000000 sec
D12 0.00002000 sec
TD0 1
SFO1 376.5453925 MHz
NUC1 19F
P1 16.00 usec
PLW1 16.42300034 W
SFO2 400.2216009 MHz
NUC2 1H
CPDPRG[2 waltz16
PCPD2 90.00 usec
PLW2 14.49600029 W
PLW12 0.45813999 W
F2 - Processing parameters
SI 65536
SF 376.5834180 MHz
WDW EM
SSB 0
LB 0.30 Hz
GB 0
PC 1.00
-203.761
0
-20
-40
-60
-80
-100
-120
-140
-160
-180
-200
ppm

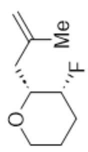
O
Me
F
cis-**13b**

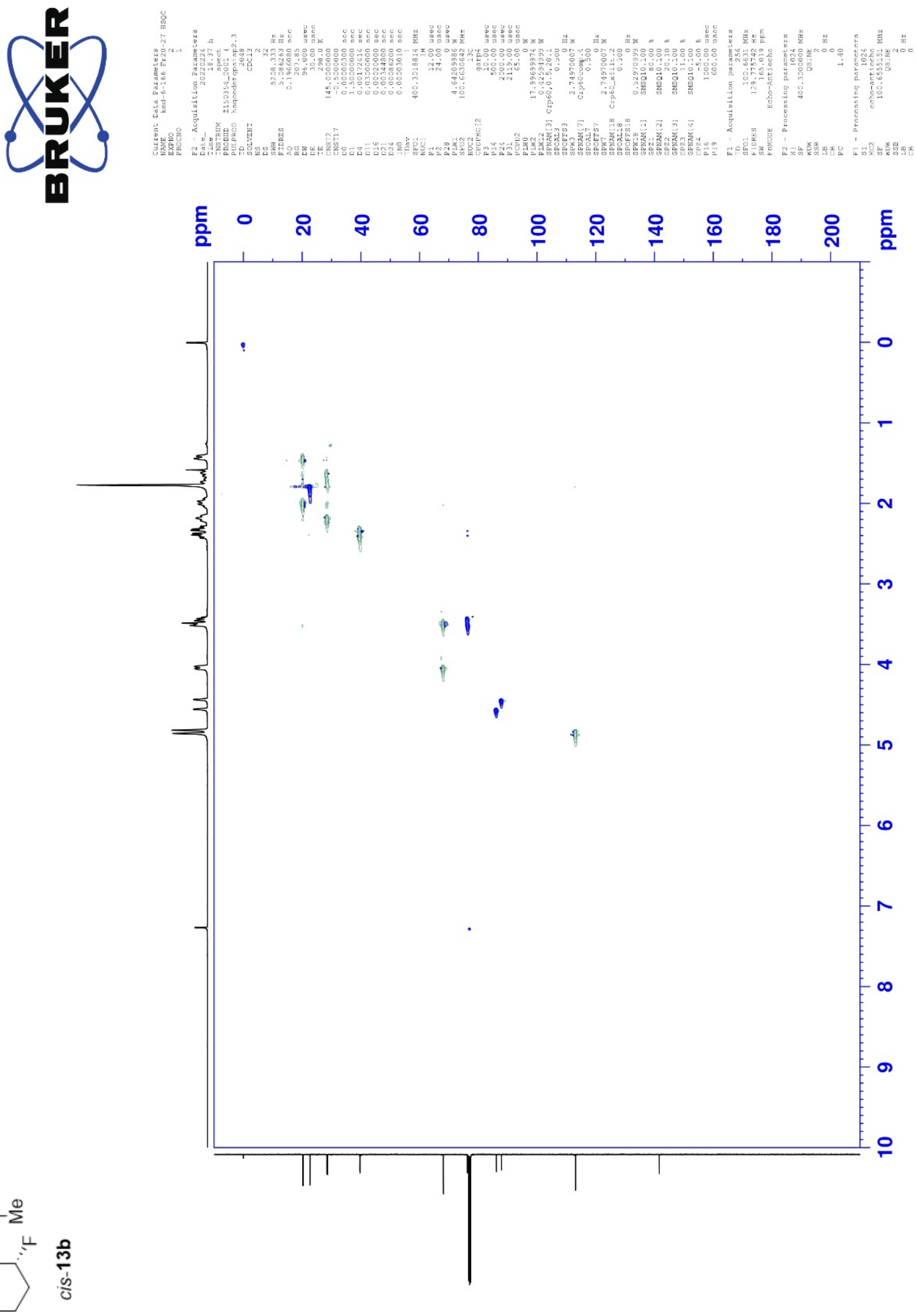

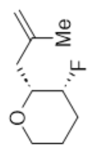

cis-**13b**

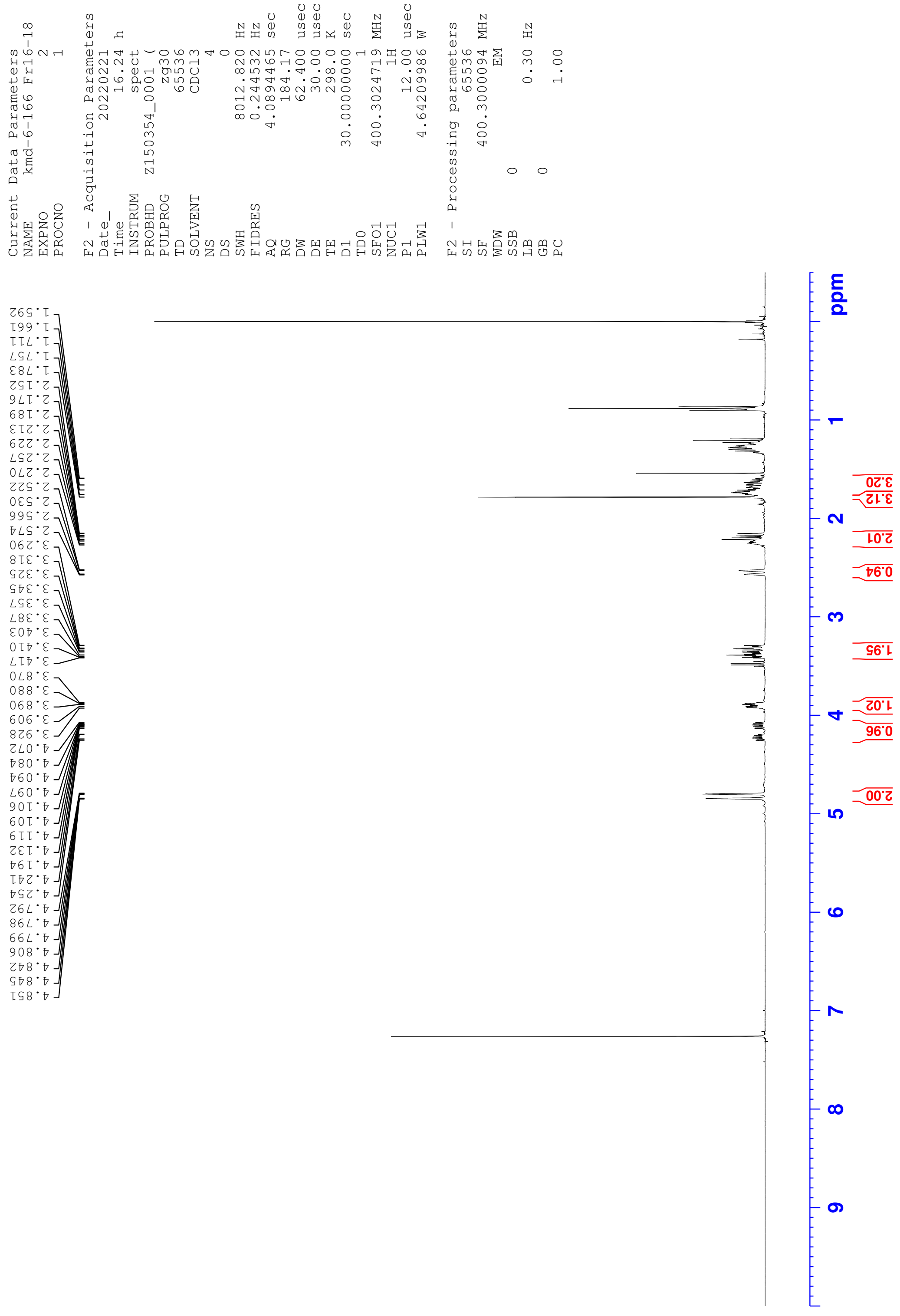
Current Data Parameters
NAME kmd-6-166 Fr16-18
EXPNO 2
PROCNO 1
F2 - Acquisition Parameters
Date_ 20220221
Time 16.24 h
INSTRUM spect
PROBHD Z150354_0001 (
PULPROG zg30
TD 65536
SOLVENT CDCl3
NS 4
DS 0
SWH 8012.820 Hz
FIDRES 0.244532 Hz
AQ 4.0894465 sec
RG 184.17
DW 62.400 usec
DE 30.00 usec
TE 298.0 K
D1 30.00000000 sec
TD0 1
SFO1 400.3024719 MHz
NUC1 1H
P1 12.00 usec
PLW1 4.64209986 W
F2 - Processing parameters
SI 65536
SF 400.3000094 MHz
WDW EM
SSB 0
LB 0.30 Hz
GB 0
PC 1.00
ppm

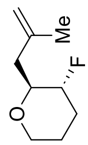
trans-**13b**

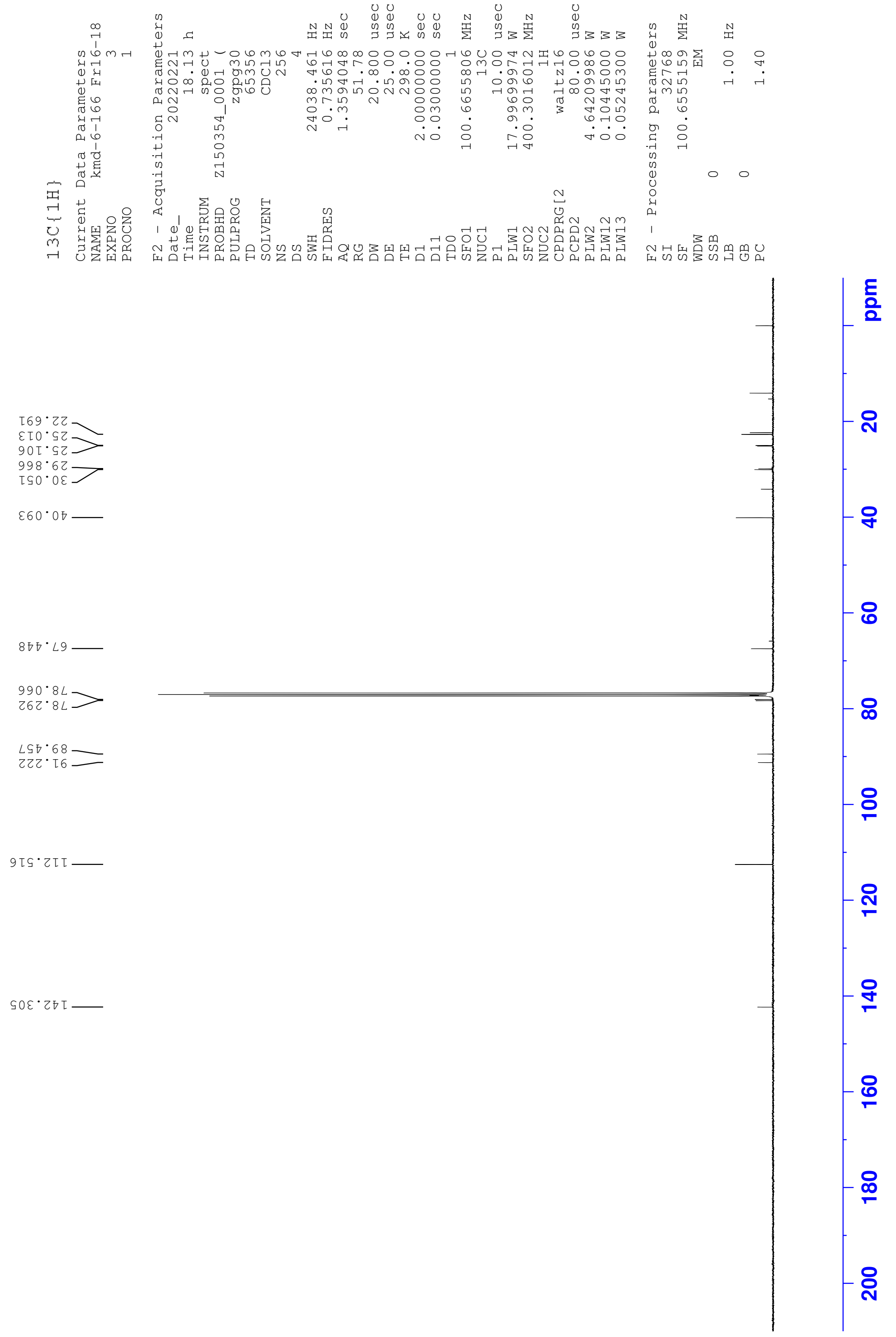
13C{1H}
Current Data Parameters
NAME kmd-6-166 Fr16-18
EXPNO 3
PROCNO 1
F2 - Acquisition Parameters
Date_ 20220221
Time_ 18.13 h
INSTRUM spect
PROBHD Z150354_0001 (
PULPROG zgpg30
TD 65356
SOLVENT CDCl3
NS 256
DS 4
SWH 24038.461 Hz
FIDRES 0.735616 Hz
AQ 1.3594048 sec
RG 51.78
DW 20.800 usec
DE 25.00 usec
TE 298.0 K
D1 2.00000000 sec
D11 0.03000000 sec
TD0 1
SFO1 100.6655806 MHz
NUC1 13C
P1 10.00 usec
PLW1 17.99699974 W
SFO2 400.3016012 MHz
NUC2 1H
CPDPRG[2 waltz16
PCPD2 80.00 usec
PLW2 4.64209986 W
PLW12 0.10445000 W
PLW13 0.05245300 W
F2 - Processing parameters
SI 32768
SF 100.6555159 MHz
WDW EM
SSB 0
LB 1.00 Hz
GB 0
PC 1.40
22.691
25.013
25.106
29.866
30.051
40.093
67.448
78.066
78.292
89.457
91.222
112.516
142.305
20
40
60
80
100
120
140
160
180
200
ppm

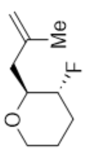
O
Me
F

trans-**13b**

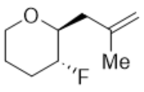

trans-**13b**

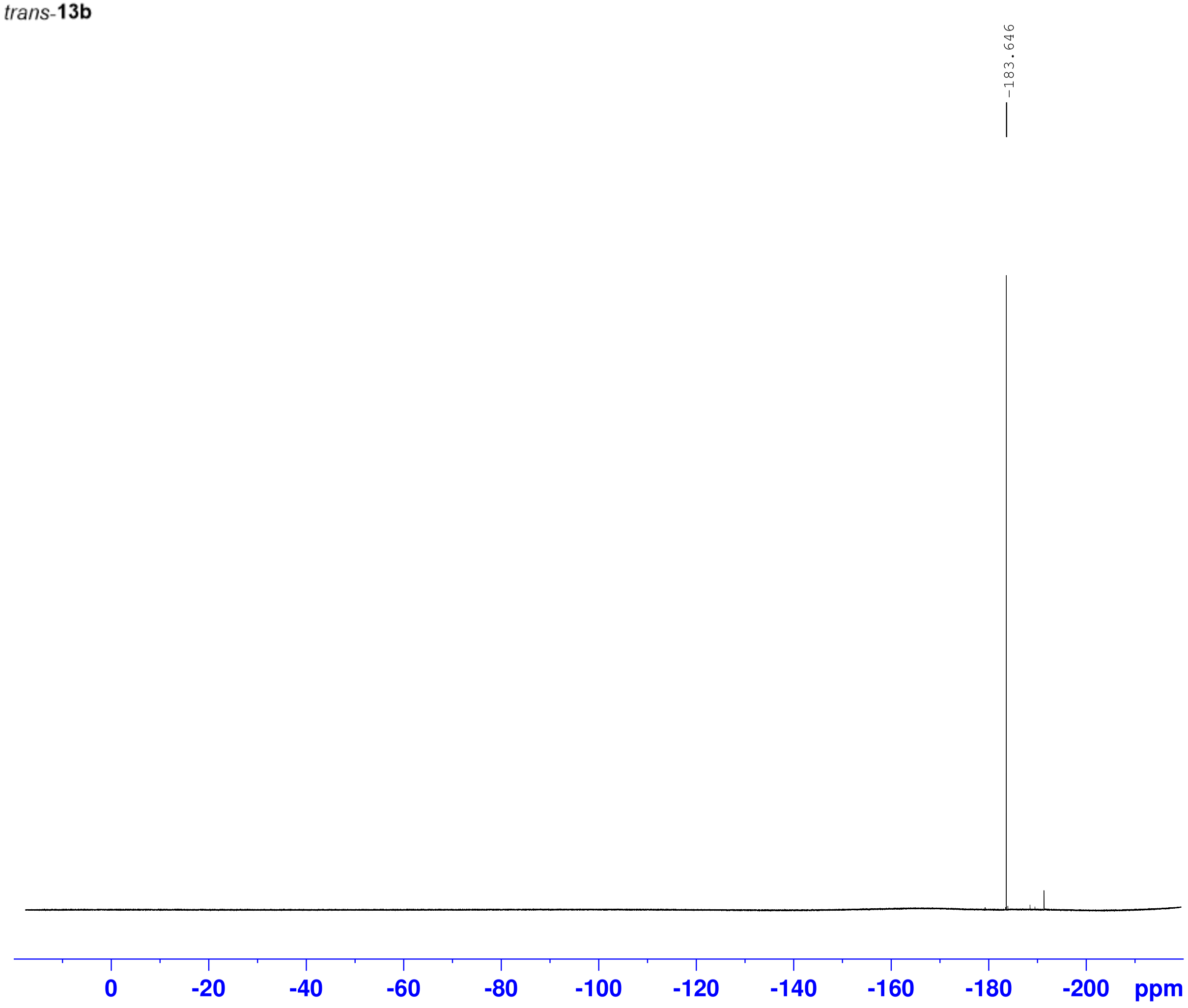


```
Current Data Parameters
NAME       kmd-6-166 Fr16-18 19F
EXPNO                      1
PROCNO                     1

F2 - Acquisition Parameters
Date_               20220222
Time                   11.03 h
INSTRUM                spect
PROBHD    Z133023_0002 (
PULPROG          zgfhigqn.2
TD                  131072
SOLVENT              CDCl3
NS                      16
DS                       4
SWH              89285.711 Hz
FIDRES            1.362392 Hz
AQ               0.7340032 sec
RG                  200.67
DW                   5.600 usec
DE                    6.50 usec
TE                   298.3 K
D1              1.00000000 sec
D11             0.03000000 sec
D12             0.00002000 sec
TD0                      1
SFO1          376.5453925 MHz
NUC1                   19F
P1                   16.00 usec
PLW1           16.42300034 W
SFO2          400.2216009 MHz
NUC2                    1H
CPDPRG[2           waltz16
PCPD2                90.00 usec
PLW2           14.49600029 W
PLW12           0.45813999 W

F2 - Processing parameters
SI                   65536
SF            376.5834166 MHz
WDW                     EM
SSB      0
LB                    0.30 Hz
GB       0
PC                    1.00
```

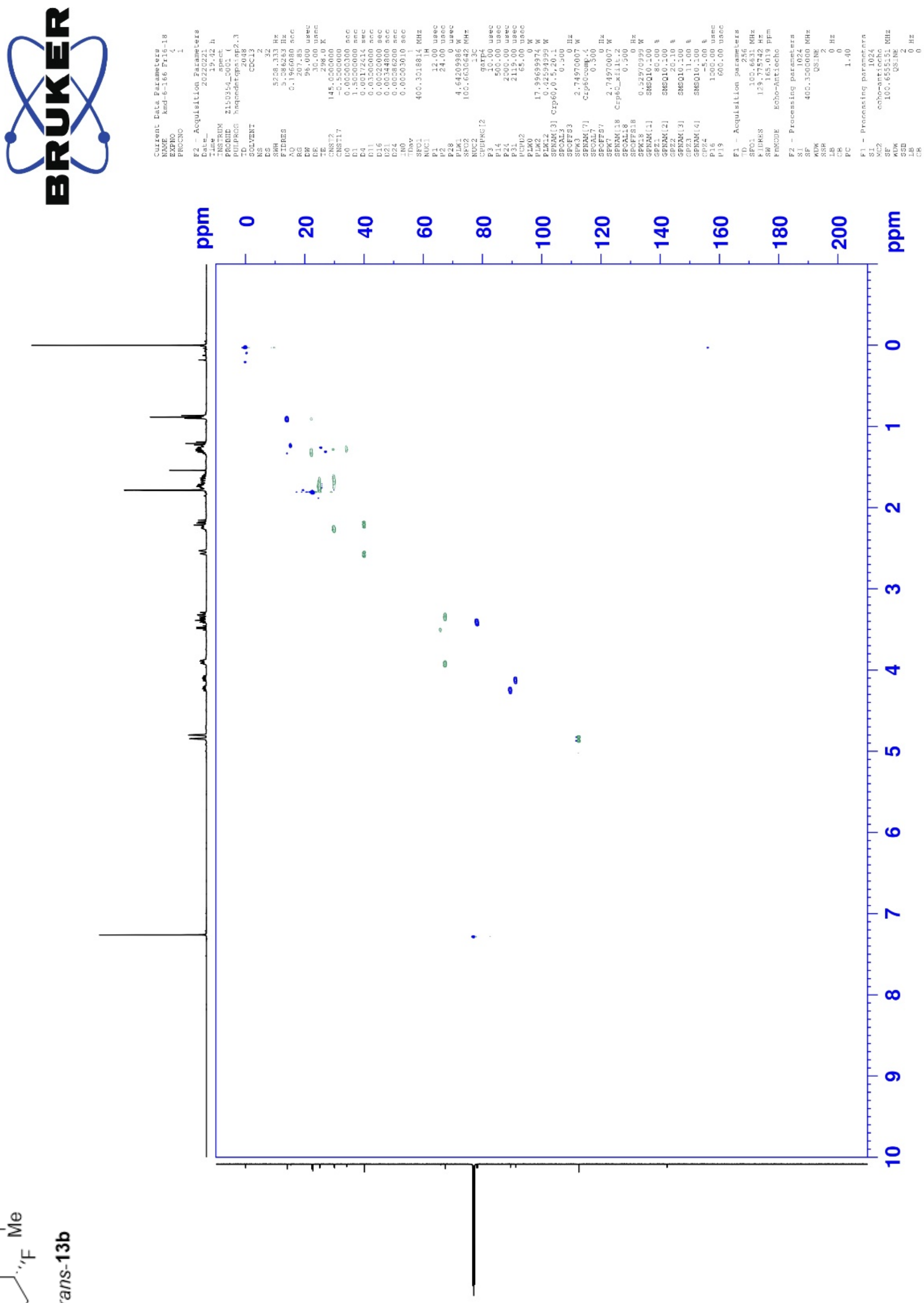

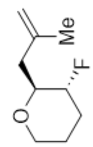

trans-**13b**

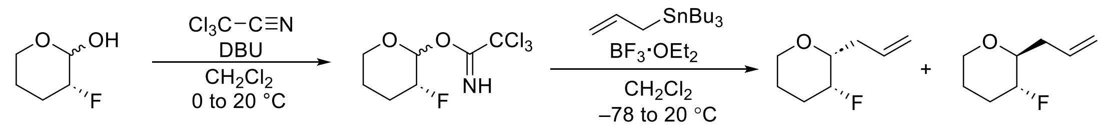

5 **6** *cis*-**13a** + *trans*-**13a**

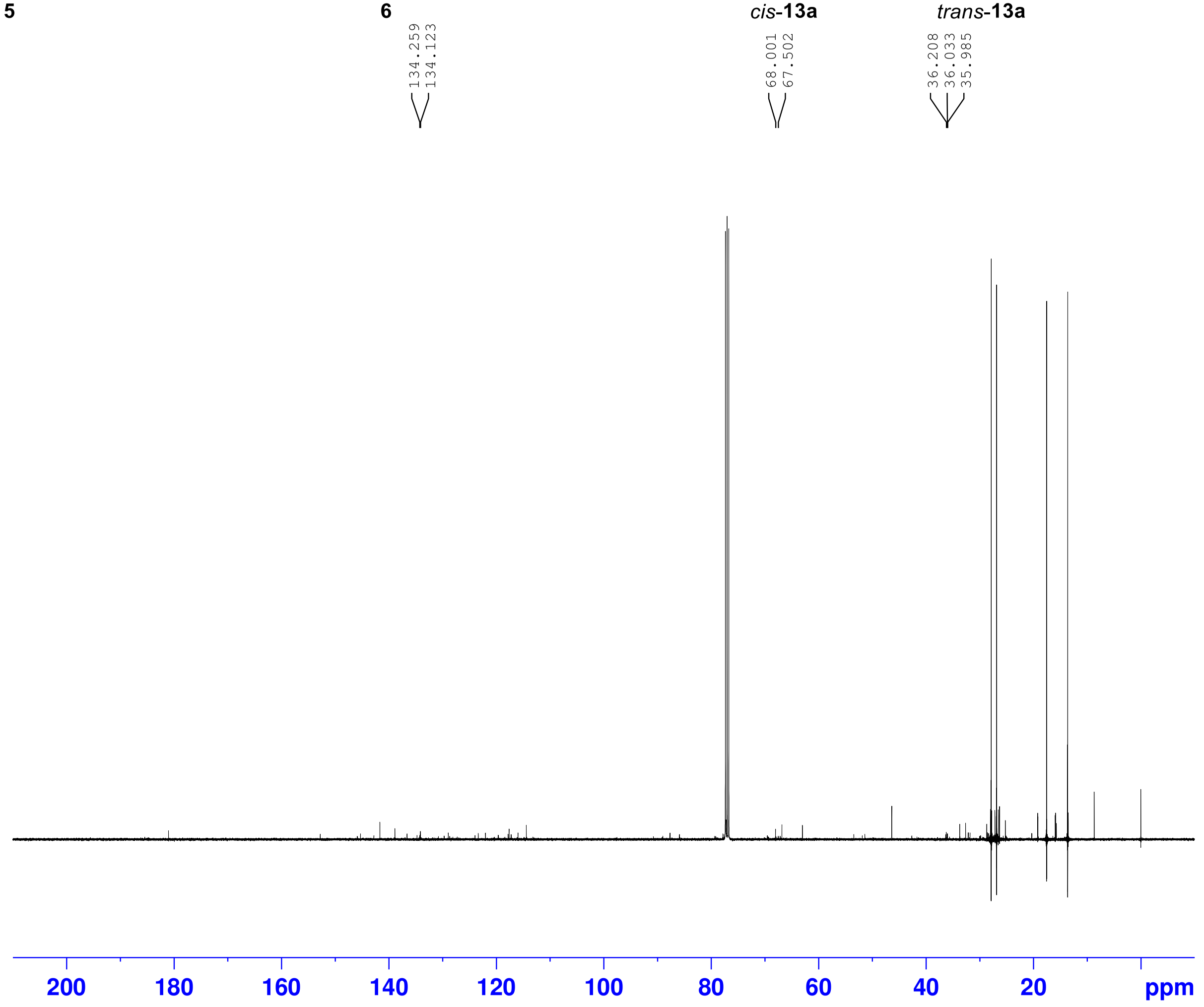

13C{1H}

```
Current Data Parameters
NAME        kmd-6-169 carbon
EXPNO                      1
PROCNO                     1

F2 - Acquisition Parameters
Date_               20220304
Time                  1.24 h
INSTRUM                spect
PROBHD     Z150354_0001 (
PULPROG              zgpg30
TD                     65356
SOLVENT                CDCl3
NS                      1024
DS                         4
SWH             24038.461 Hz
FIDRES           0.735616 Hz
AQ              1.3594048 sec
RG                     29.21
DW                    20.800 usec
DE                     25.00 usec
TE                     298.0 K
D1             2.00000000 sec
D11            0.03000000 sec
TD0                        1
SFO1         100.6655806 MHz
NUC1                     13C
P1                     10.00 usec
PLW1          17.99699974 W
SFO2         400.3016012 MHz
NUC2                      1H
CPDPRG[2             waltz16
PCPD2                  80.00 usec
PLW2           4.64209986 W
PLW12          0.10445000 W
PLW13          0.05245300 W

F2 - Processing parameters
SI                    131072
SF           100.6555160 MHz
WDW                       EM
SSB         0
LB          0 Hz
GB          0
PC                      1.40
```

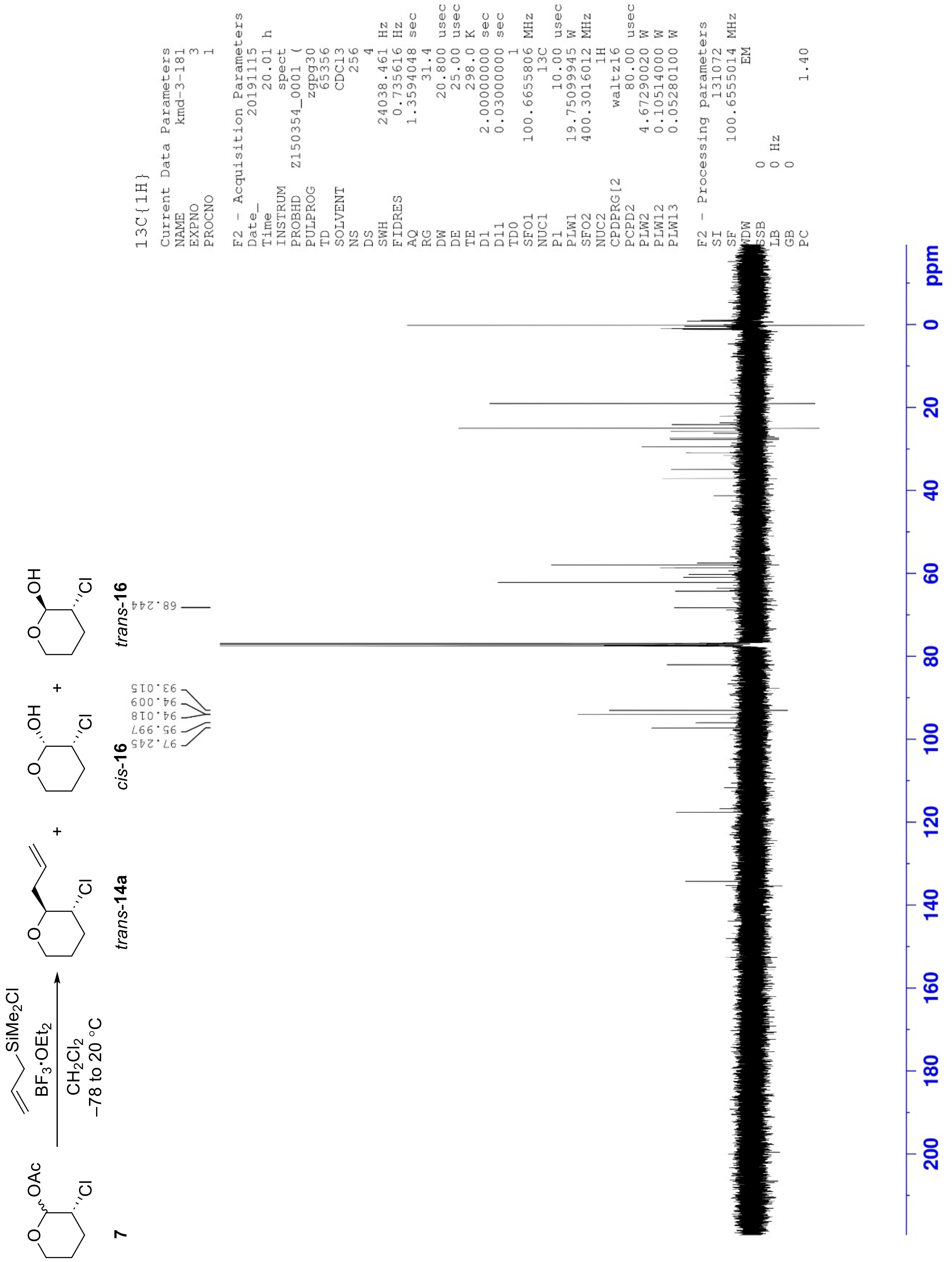
7
SiMe2Cl
BF3·OEt2
CH2Cl2
–78 to 20 °C
trans-14a
cis-16
trans-16
68.244
93.015
94.009
94.018
95.997
97.245
13C{1H}
Current Data Parameters
NAME kmd-3-181
EXPNO 3
PROCNO 1
F2 - Acquisition Parameters
Date_ 20191115
Time 20.01 h
INSTRUM spect
PROBHD Z150354_0001 (
PULPROG zgpg30
TD 65356
SOLVENT CDCl3
NS 256
DS 4
SWH 24038.461 Hz
FIDRES 0.735616 Hz
AQ 1.3594048 sec
RG 31.4
DW 20.800 usec
DE 25.00 usec
TE 298.0 K
D1 2.00000000 sec
D11 0.03000000 sec
TD0 1
SFO1 100.6655806 MHz
NUC1 13C
P1 10.00 usec
PLW1 19.75099945 W
SFO2 400.3016012 MHz
NUC2 1H
CPDPRG[2 waltz16
PCPD2 80.00 usec
PLW2 4.67290020 W
PLW12 0.10514000 W
PLW13 0.05280100 W
F2 - Processing parameters
SI 131072
SF 100.6555014 MHz
WDW EM
SSB 0
LB 0 Hz
GB 0
PC 1.40
ppm
0
20
40
60
80
100
120
140
160
180
200

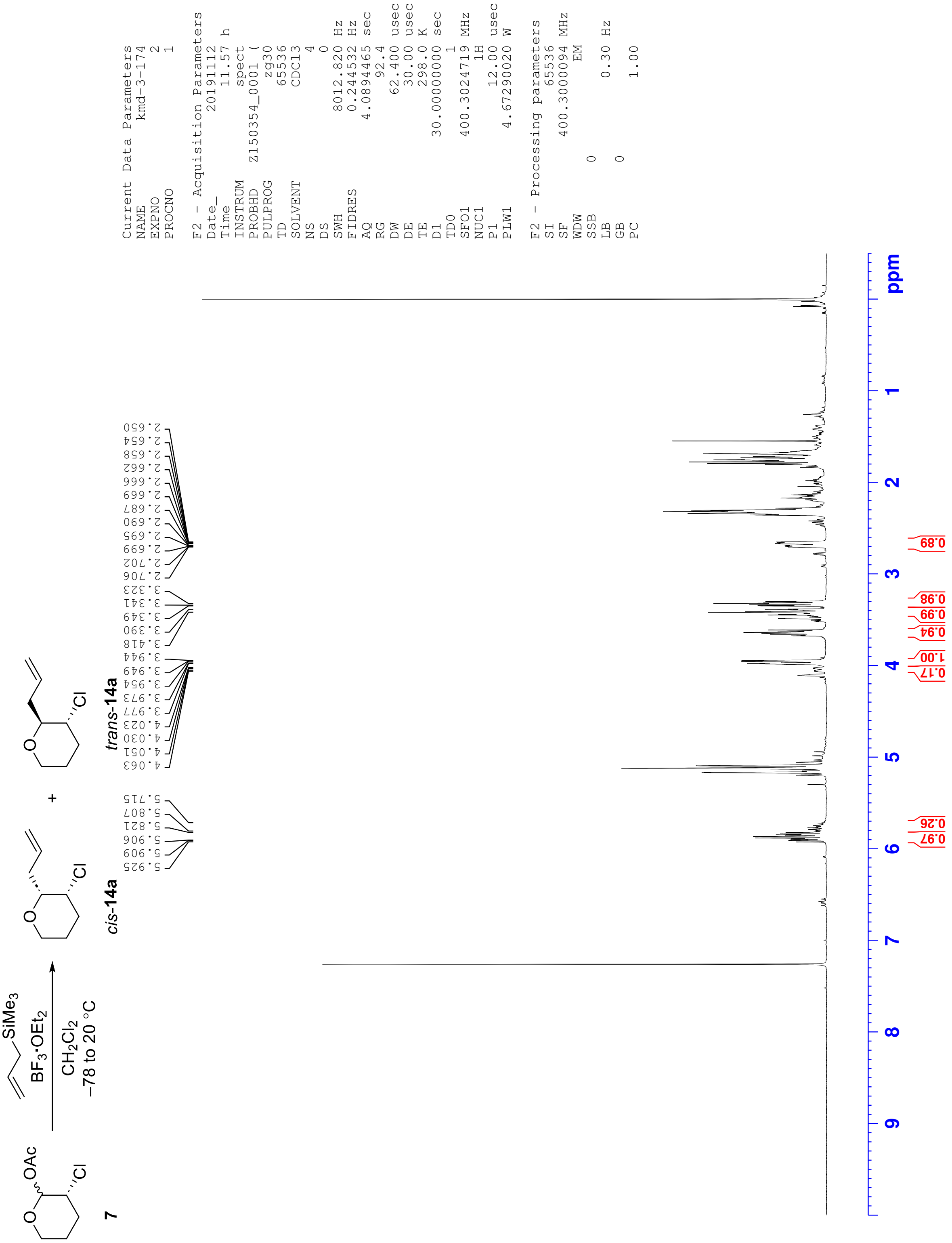
OAc
Cl
7
SiMe3
BF3·OEt2
CH2Cl2
–78 to 20 °C
cis-14a
+
trans-14a
Current Data Parameters
NAME kmd-3-174
EXPNO 2
PROCNO 1
F2 - Acquisition Parameters
Date_ 20191112
Time 11.57 h
INSTRUM spect
PROBHD Z150354_0001 (
PULPROG zg30
TD 65536
SOLVENT CDCl3
NS 4
DS 0
SWH 8012.820 Hz
FIDRES 0.244532 Hz
AQ 4.0894465 sec
RG 92.4
DW 62.400 usec
DE 30.00 usec
TE 298.0 K
D1 30.00000000 sec
TD0 1
SFO1 400.3024719 MHz
NUC1 1H
P1 12.00 usec
PLW1 4.67290020 W
F2 - Processing parameters
SI 65536
SF 400.3000094 MHz
WDW EM
SSB 0
LB 0.30 Hz
GB 0
PC 1.00
2.650
2.654
2.658
2.662
2.666
2.669
2.687
2.690
2.695
2.699
2.702
2.706
3.323
3.341
3.349
3.390
3.418
3.944
3.949
3.954
3.973
3.977
4.023
4.030
4.051
4.063
5.715
5.807
5.821
5.906
5.909
5.925
0.89
0.98
0.99
0.94
1.00
0.17
0.26
0.97
9
8
7
6
5
4
3
2
1
ppm

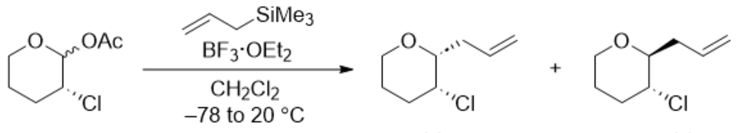

7   *cis*-**14a**   *trans*-**14a**

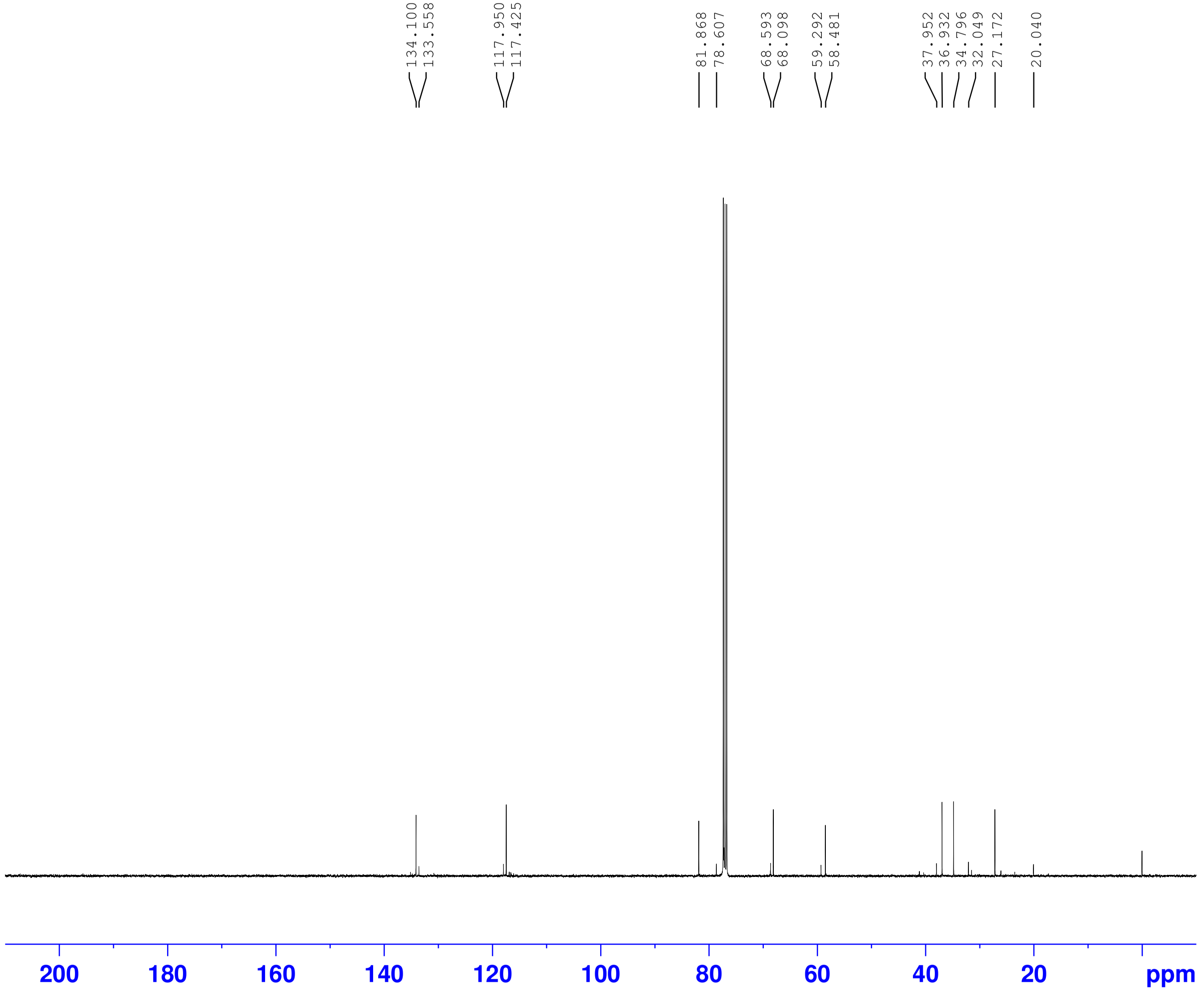

13C{1H}

```
Current Data Parameters
NAME             kmd-3-174
EXPNO                    3
PROCNO                   1

F2 - Acquisition Parameters
Date_             20191112
Time                 13.07 h
INSTRUM              spect
PROBHD    Z150354_0001 (
PULPROG            zgpg30
TD                  65356
SOLVENT             CDCl3
NS                    256
DS                      4
SWH             24038.461 Hz
FIDRES           0.735616 Hz
AQ              1.3594048 sec
RG                   31.4
DW                 20.800 usec
DE                  25.00 usec
TE                  298.0 K
D1             2.00000000 sec
D11            0.03000000 sec
TD0                     1
SFO1          100.6655806 MHz
NUC1                  13C
P1                  10.00 usec
PLW1          19.75099945 W
SFO2          400.3016012 MHz
NUC2                   1H
CPDPRG[2          waltz16
PCPD2               80.00 usec
PLW2           4.67290020 W
PLW12          0.10514000 W
PLW13          0.05280100 W

F2 - Processing parameters
SI                  32768
SF            100.6555157 MHz
WDW                    EM
SSB      0
LB                   1.00 Hz
GB       0
PC                   1.40
```

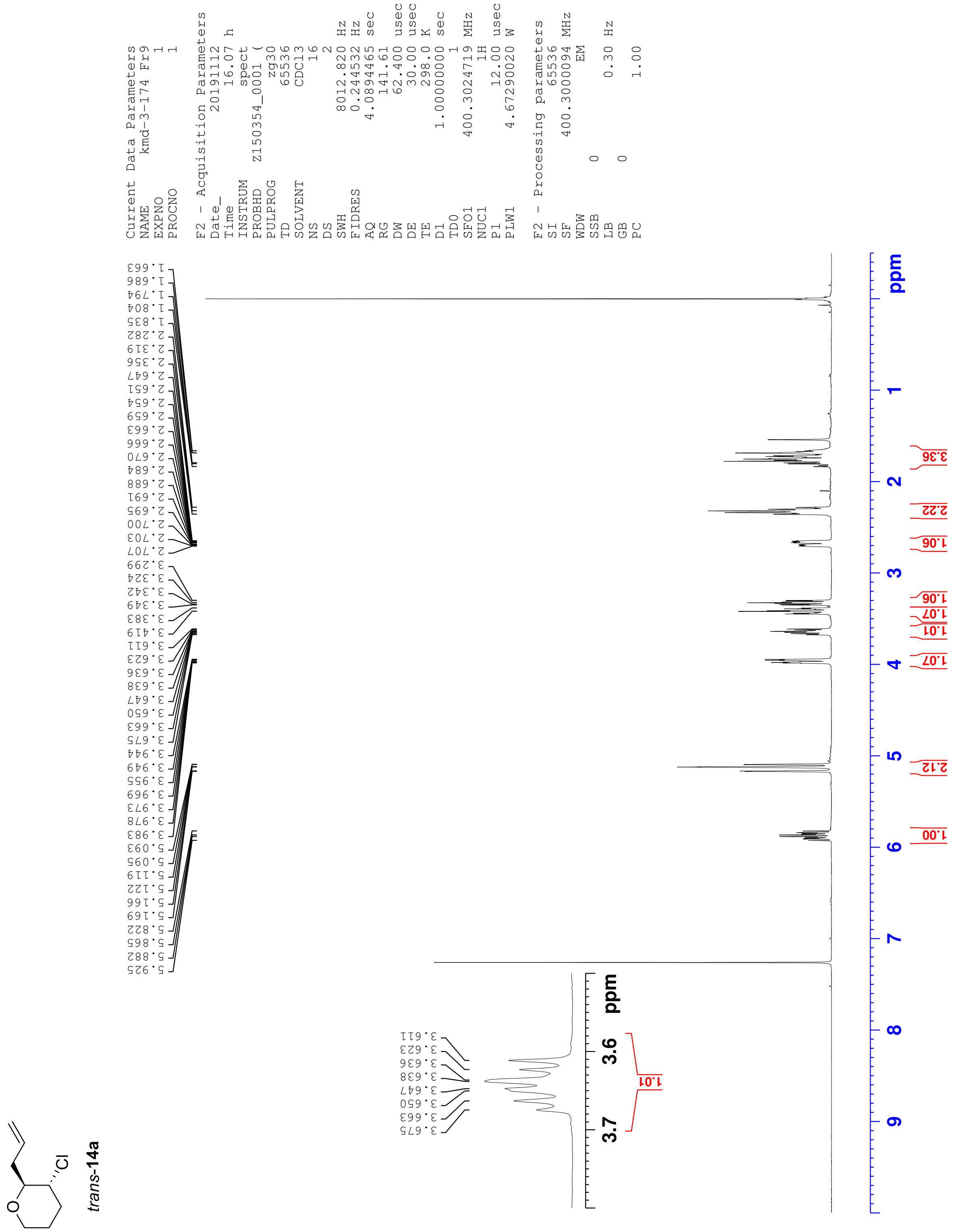
Current Data Parameters
NAME kmd-3-174 Fr9
EXPNO 1
PROCNO 1
F2 - Acquisition Parameters
Date_ 20191112
Time 16.07 h
INSTRUM spect
PROBHD Z150354_0001 (
PULPROG zg30
TD 65536
SOLVENT CDCl3
NS 16
DS 2
SWH 8012.820 Hz
FIDRES 0.244532 Hz
AQ 4.0894465 sec
RG 141.61
DW 62.400 usec
DE 30.00 usec
TE 298.0 K
D1 1.00000000 sec
TD0 1
SFO1 400.3024719 MHz
NUC1 1H
P1 12.00 usec
PLW1 4.67290020 W
F2 - Processing parameters
SI 65536
SF 400.3000094 MHz
WDW EM
SSB 0
LB 0.30 Hz
GB 0
PC 1.00
5.925 5.882 5.865 5.822 5.169 5.166 5.122 5.119 5.095 5.093 3.983 3.978 3.973 3.969 3.955 3.949 3.944 3.675 3.663 3.650 3.647 3.638 3.636 3.623 3.611 3.419 3.383 3.349 3.342 3.324 3.299 2.707 2.703 2.700 2.695 2.691 2.688 2.684 2.670 2.666 2.663 2.659 2.654 2.651 2.647 2.356 2.319 2.282 1.835 1.804 1.794 1.686 1.663
1.00
2.12
1.07
1.01
1.07
1.06
1.06
2.22
3.36
ppm
3.6
3.7
ppm
Cl
O
trans-14a

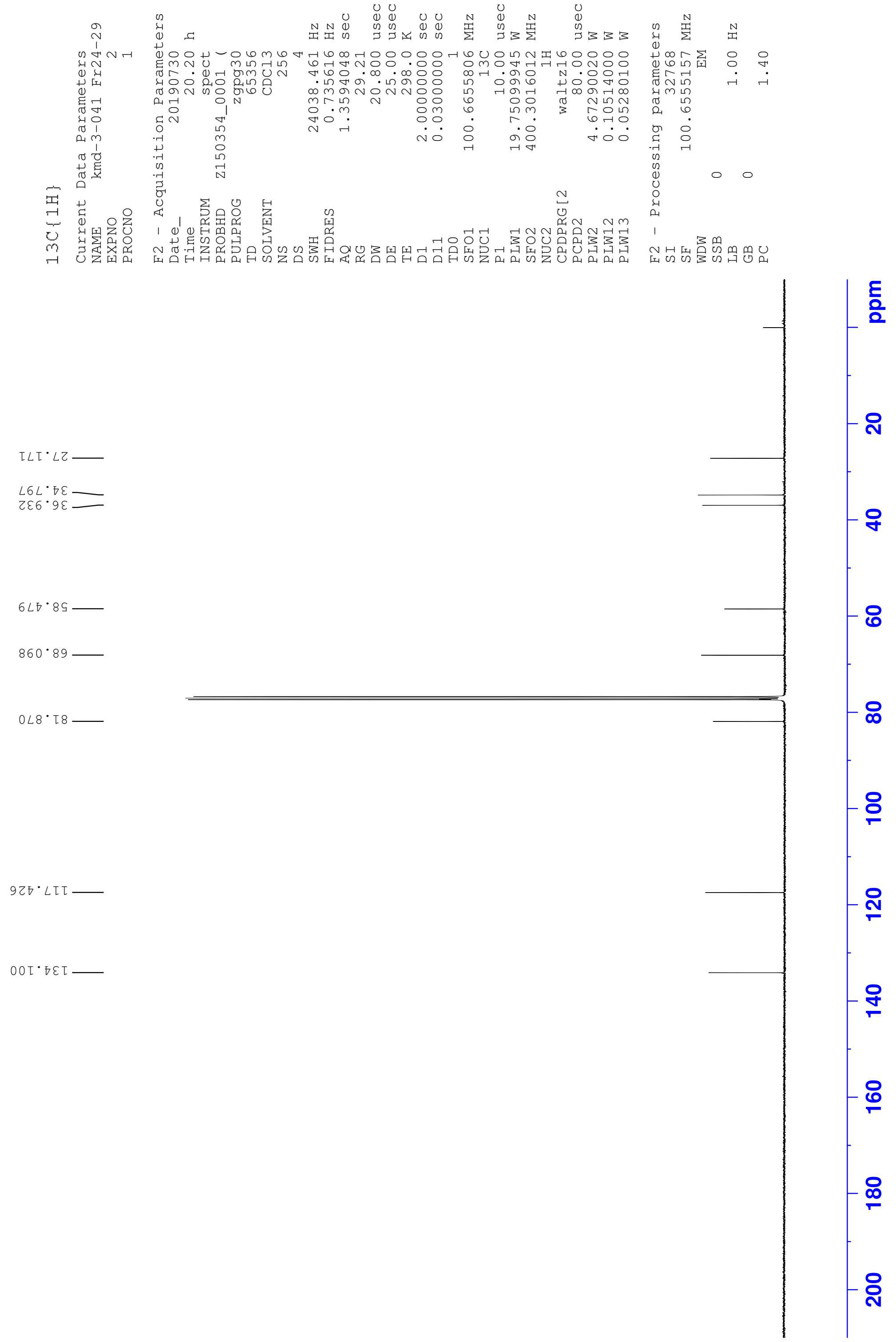
13C{1H}
Current Data Parameters
NAME kmd-3-041 Fr24-29
EXPNO 2
PROCNO 1
F2 - Acquisition Parameters
Date_ 20190730
Time 20.20 h
INSTRUM spect
PROBHD Z150354_0001 (
PULPROG zgpg30
TD 65536
SOLVENT CDCl3
NS 256
DS 4
SWH 24038.461 Hz
FIDRES 0.735616 Hz
AQ 1.3594048 sec
RG 29.21
DW 20.800 usec
DE 25.00 usec
TE 298.0 K
D1 2.00000000 sec
D11 0.03000000 sec
TD0 1
SFO1 100.6655806 MHz
NUC1 13C
P1 10.00 usec
PLW1 19.75099945 W
SFO2 400.3016012 MHz
NUC2 1H
CPDPRG[2 waltz16
PCPD2 80.00 usec
PLW2 4.67290020 W
PLW12 0.10514000 W
PLW13 0.05280100 W
F2 - Processing parameters
SI 32768
SF 100.6555157 MHz
WDW EM
SSB 0
LB 1.00 Hz
GB 0
PC 1.40
27.171
34.797
36.932
58.479
68.098
81.870
117.426
134.100
ppm
20
40
60
80
100
120
140
160
180
200

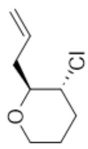
O
Cl

trans-**14a**

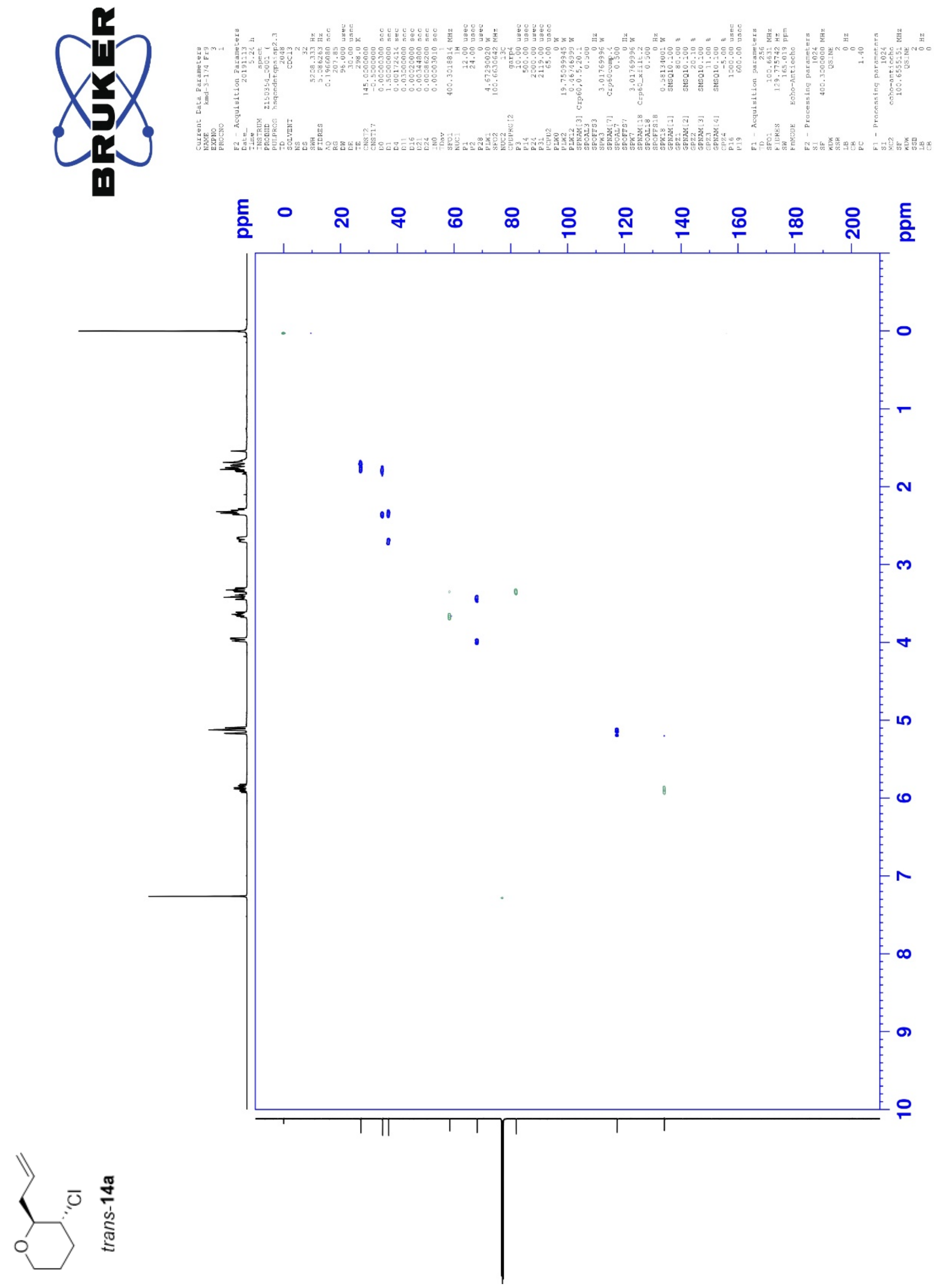

trans-**14a**

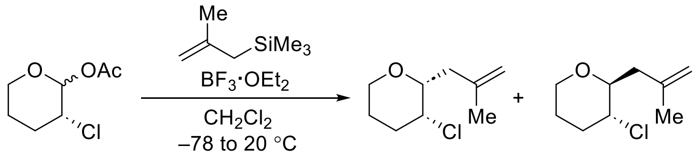

7 | *cis*-**14b** | *trans*-**14b**

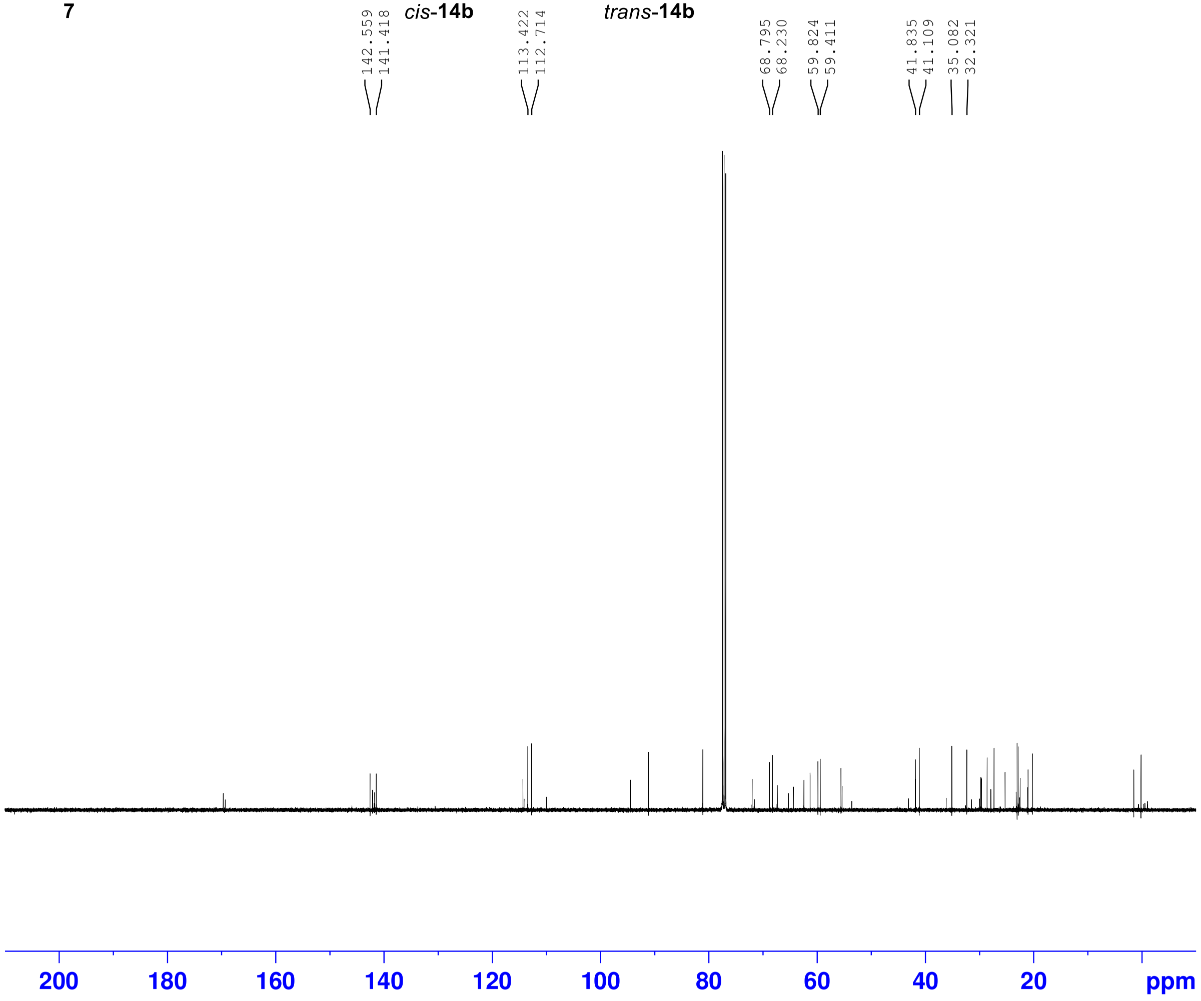


```
13C{1H}

Current Data Parameters
NAME             kmd-3-040
EXPNO                    2
PROCNO                   1

F2 - Acquisition Parameters
Date_             20190726
Time                  7.54 h
INSTRUM              spect
PROBHD   Z150354_0001 (
PULPROG            zgpg30
TD                   65356
SOLVENT              CDCl3
NS                     512
DS                       4
SWH              24038.461 Hz
FIDRES            0.735616 Hz
AQ               1.3594048 sec
RG                   36.68
DW                  20.800 usec
DE                   25.00 usec
TE                   298.0 K
D1              2.00000000 sec
D11             0.03000000 sec
TD0                      1
SFO1           100.6655806 MHz
NUC1                   13C
P1                   10.00 usec
PLW1           19.75099945 W
SFO2           400.3016012 MHz
NUC2                    1H
CPDPRG[2           waltz16
PCPD2                80.00 usec
PLW2            4.67290020 W
PLW12           0.10514000 W
PLW13           0.05280100 W

F2 - Processing parameters
SI                  131072
SF             100.6555014 MHz
WDW                     EM
SSB       0
LB        0 Hz
GB        0
PC                    1.40
```

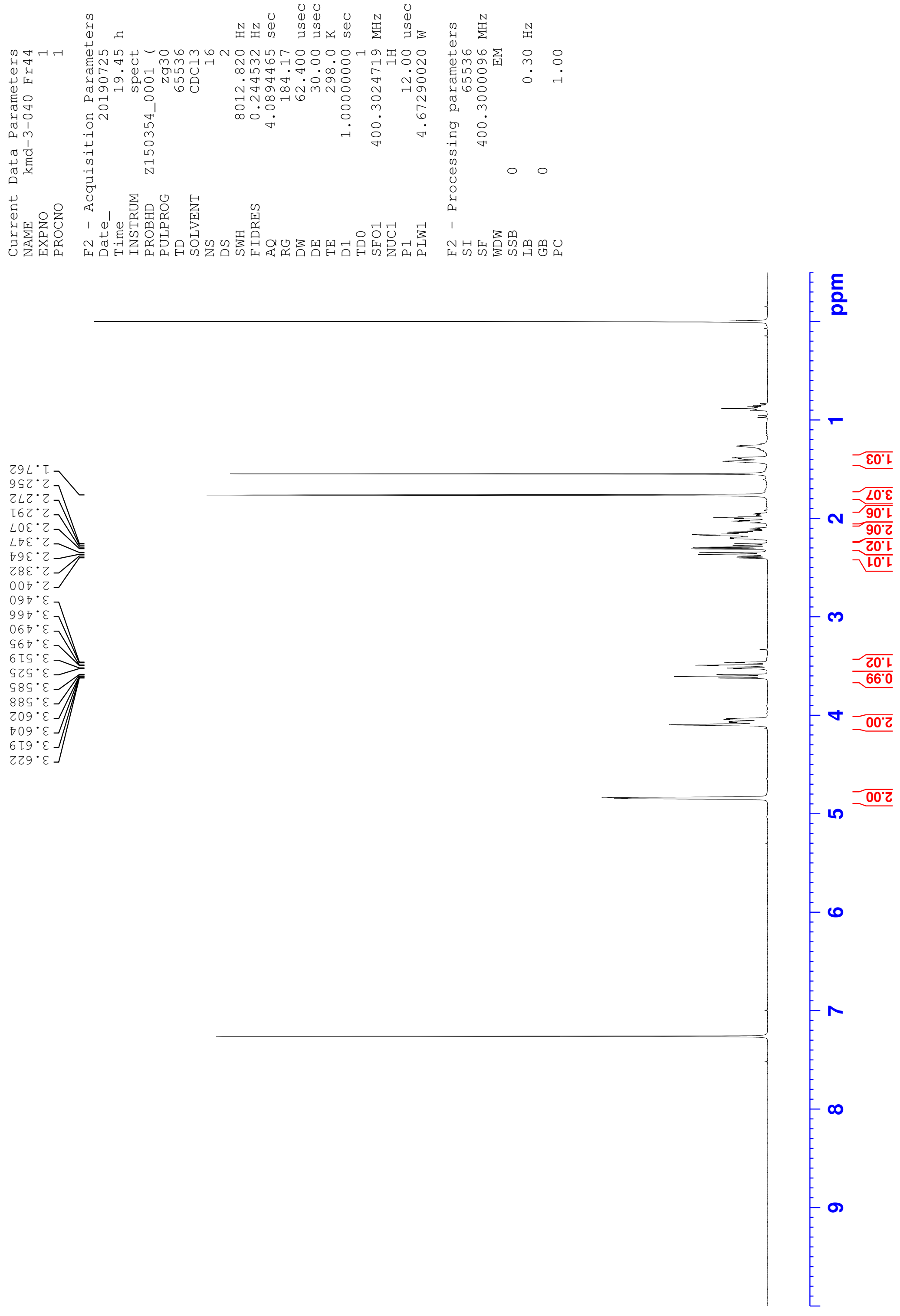
Current Data Parameters
NAME kmd-3-040 Fr44
EXPNO 1
PROCNO 1
F2 - Acquisition Parameters
Date_ 20190725
Time 19.45 h
INSTRUM spect
PROBHD Z150354_0001 (
PULPROG zg30
TD 65536
SOLVENT CDCl3
NS 16
DS 2
SWH 8012.820 Hz
FIDRES 0.244532 Hz
AQ 4.0894465 sec
RG 184.17
DW 62.400 usec
DE 30.00 usec
TE 298.0 K
D1 1.00000000 sec
TD0 1
SFO1 400.3024719 MHz
NUC1 1H
P1 12.00 usec
PLW1 4.67290020 W
F2 - Processing parameters
SI 65536
SF 400.3000096 MHz
WDW EM
SSB 0
LB 0.30 Hz
GB 0
PC 1.00
1.762
2.256
2.272
2.291
2.307
2.347
2.364
2.382
2.400
3.460
3.466
3.490
3.495
3.519
3.525
3.585
3.588
3.602
3.604
3.619
3.622
1.03
3.07
1.06
2.06
1.02
1.01
1.02
0.99
2.00
2.00
ppm
1
2
3
4
5
6
7
8
9

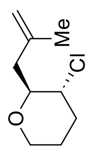
O
Cl
Me

trans-**14b**

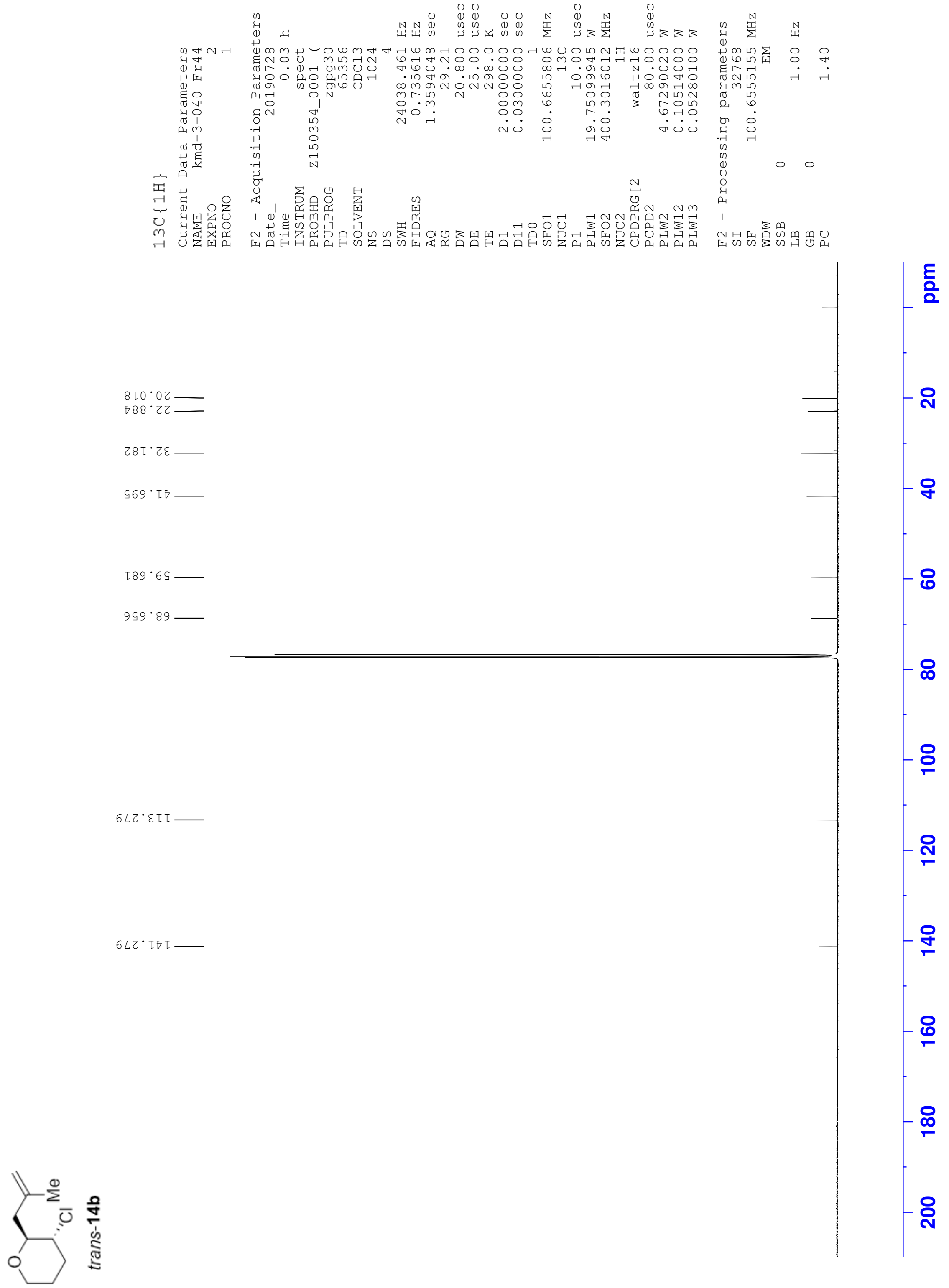
13C{1H}
Current Data Parameters
NAME kmd-3-040 Fr44
EXPNO 2
PROCNO 1
F2 - Acquisition Parameters
Date_ 20190728
Time_ 0.03 h
INSTRUM spect
PROBHD Z150354_0001 (
PULPROG zgpg30
TD 65356
SOLVENT CDCl3
NS 1024
DS 4
SWH 24038.461 Hz
FIDRES 0.735616 Hz
AQ 1.3594048 sec
RG 29.21
DW 20.800 usec
DE 25.00 usec
TE 298.0 K
D1 2.00000000 sec
D11 0.03000000 sec
TD0 1
SFO1 100.6655806 MHz
NUC1 13C
P1 10.00 usec
PLW1 19.75099945 W
SFO2 400.3016012 MHz
NUC2 1H
CPDPRG[2 waltz16
PCPD2 80.00 usec
PLW2 4.67290020 W
PLW12 0.10514000 W
PLW13 0.05280100 W
F2 - Processing parameters
SI 32768
SF 100.6555155 MHz
WDW EM
SSB 0
LB 1.00 Hz
GB 0
PC 1.40
20.018
22.884
32.182
41.695
59.681
68.656
113.279
141.279
ppm
20
40
60
80
100
120
140
160
180
200
Me
Cl
O
trans-14b

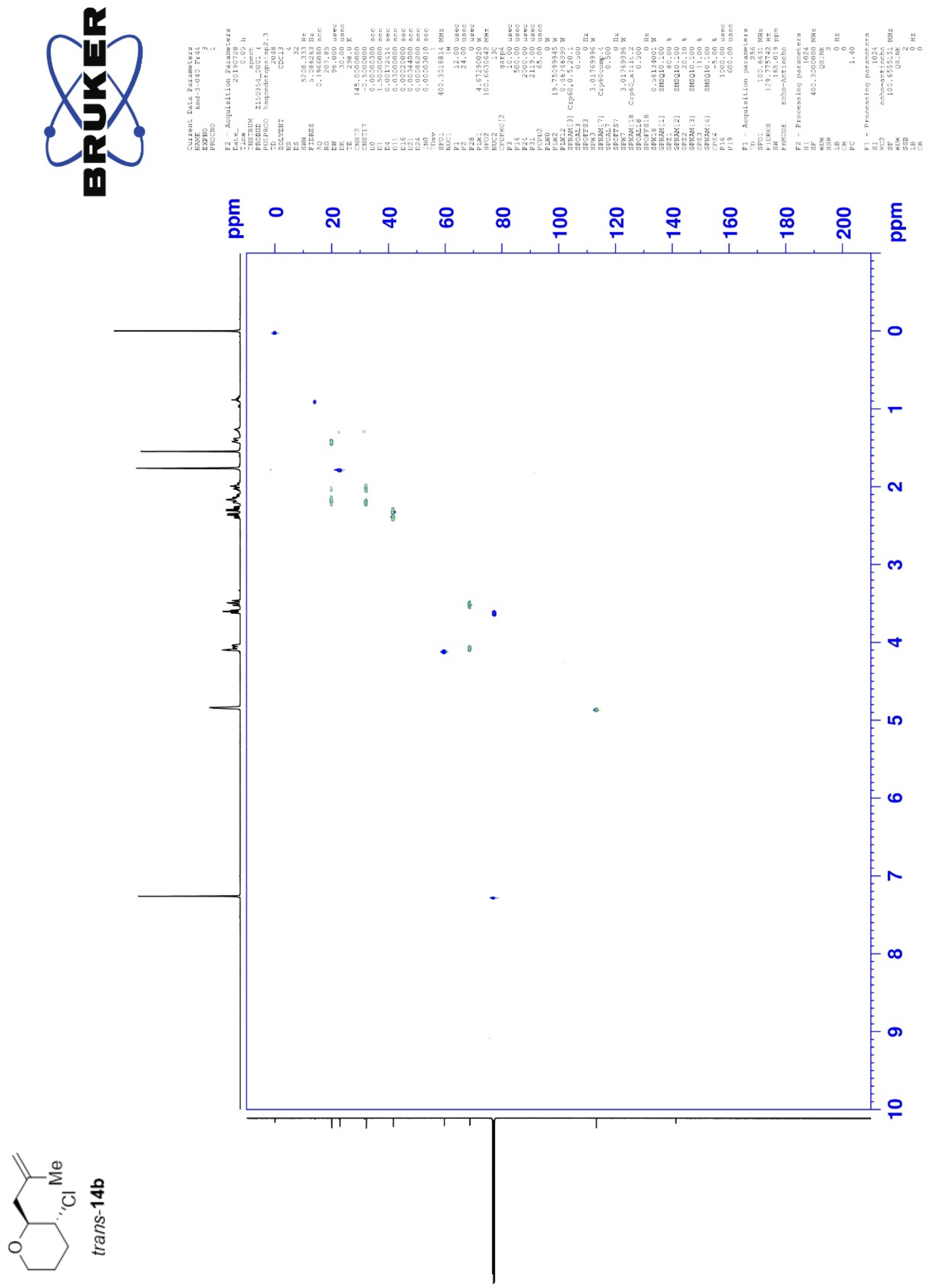

trans-**14b**

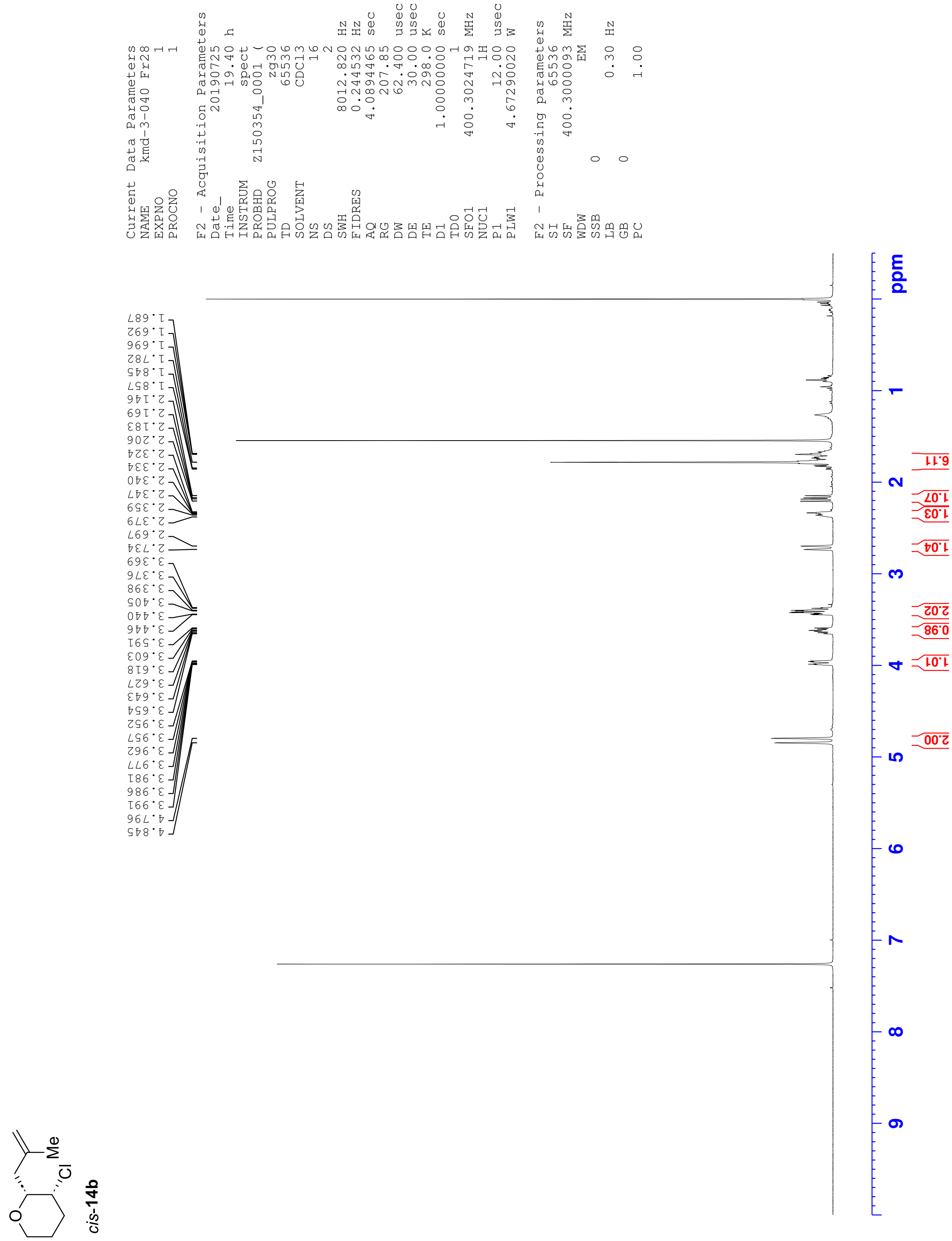
Current Data Parameters
NAME kmd-3-040 Fr28
EXPNO 1
PROCNO 1
F2 - Acquisition Parameters
Date_ 20190725
Time 19.40 h
INSTRUM spect
PROBHD Z150354_0001 (
PULPROG zg30
TD 65536
SOLVENT CDCl3
NS 16
DS 2
SWH 8012.820 Hz
FIDRES 0.244532 Hz
AQ 4.0894465 sec
RG 207.85
DW 62.400 usec
DE 30.00 usec
TE 298.0 K
D1 1.00000000 sec
TD0 1
SFO1 400.3024719 MHz
NUC1 1H
P1 12.00 usec
PLW1 4.67290020 W
F2 - Processing parameters
SI 65536
SF 400.3000093 MHz
WDW EM
SSB 0
LB 0.30 Hz
GB 0
PC 1.00
ppm

cis-**14b**

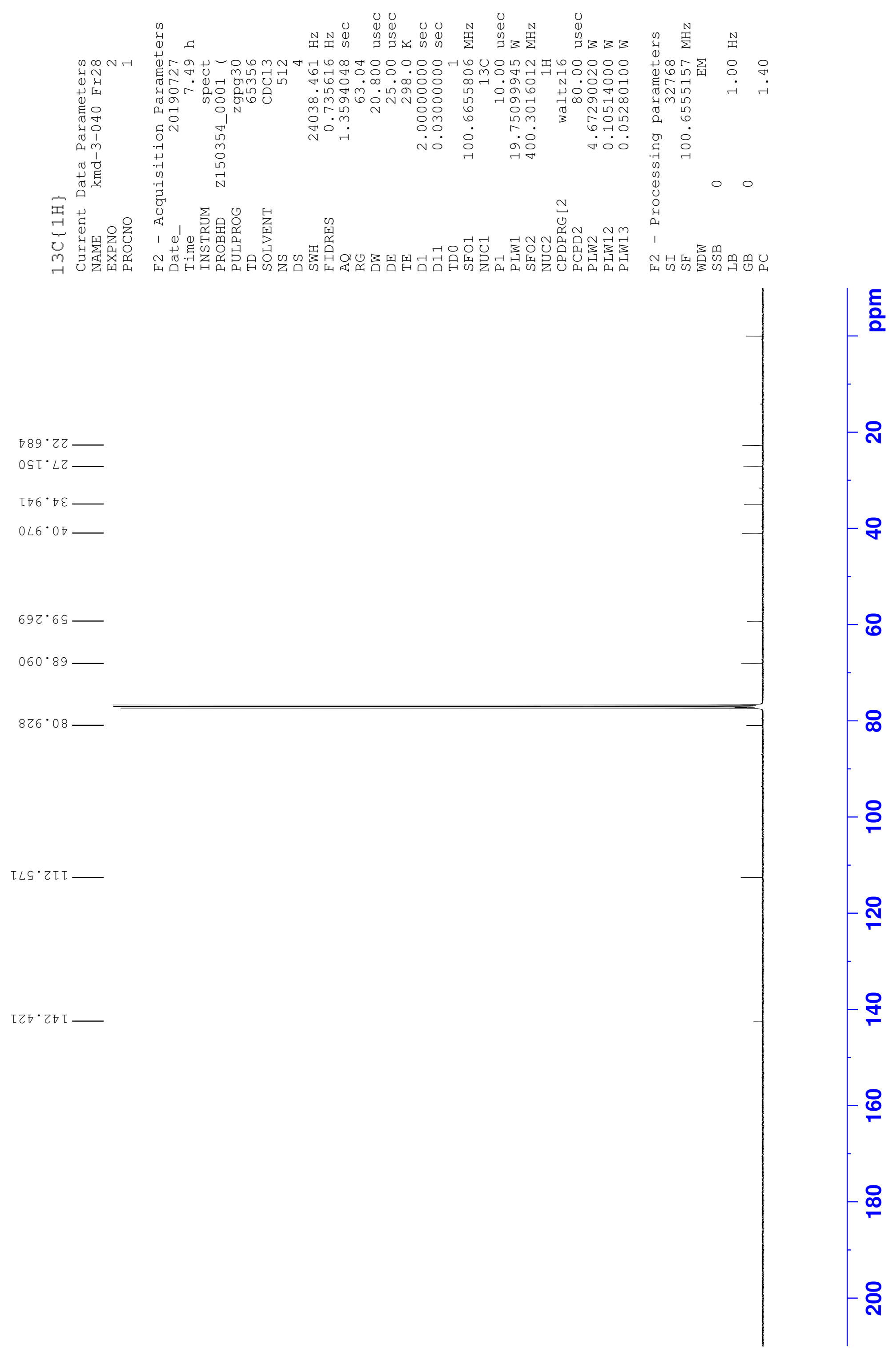
13C{1H}
Current Data Parameters
NAME kmd-3-040 Fr28
EXPNO 2
PROCNO 1
F2 - Acquisition Parameters
Date_ 20190727
Time_ 7.49 h
INSTRUM spect
PROBHD Z150354_0001 (
PULPROG zgpg30
TD 65356
SOLVENT CDCl3
NS 512
DS 4
SWH 24038.461 Hz
FIDRES 0.735616 Hz
AQ 1.3594048 sec
RG 63.04
DW 20.800 usec
DE 25.00 usec
TE 298.0 K
D1 2.00000000 sec
D11 0.03000000 sec
TD0 1
SFO1 100.6655806 MHz
NUC1 13C
P1 10.00 usec
PLW1 19.75099945 W
SFO2 400.3016012 MHz
NUC2 1H
CPDPRG[2 waltz16
PCPD2 80.00 usec
PLW2 4.67290020 W
PLW12 0.10514000 W
PLW13 0.05280100 W
F2 - Processing parameters
SI 32768
SF 100.6555157 MHz
WDW EM
SSB 0
LB 1.00 Hz
GB 0
PC 1.40
22.684
27.150
34.941
40.970
59.269
68.090
80.928
112.571
142.421
ppm
20
40
60
80
100
120
140
160
180
200

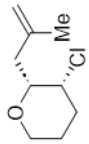
O
Cl
Me

cis-**14b**

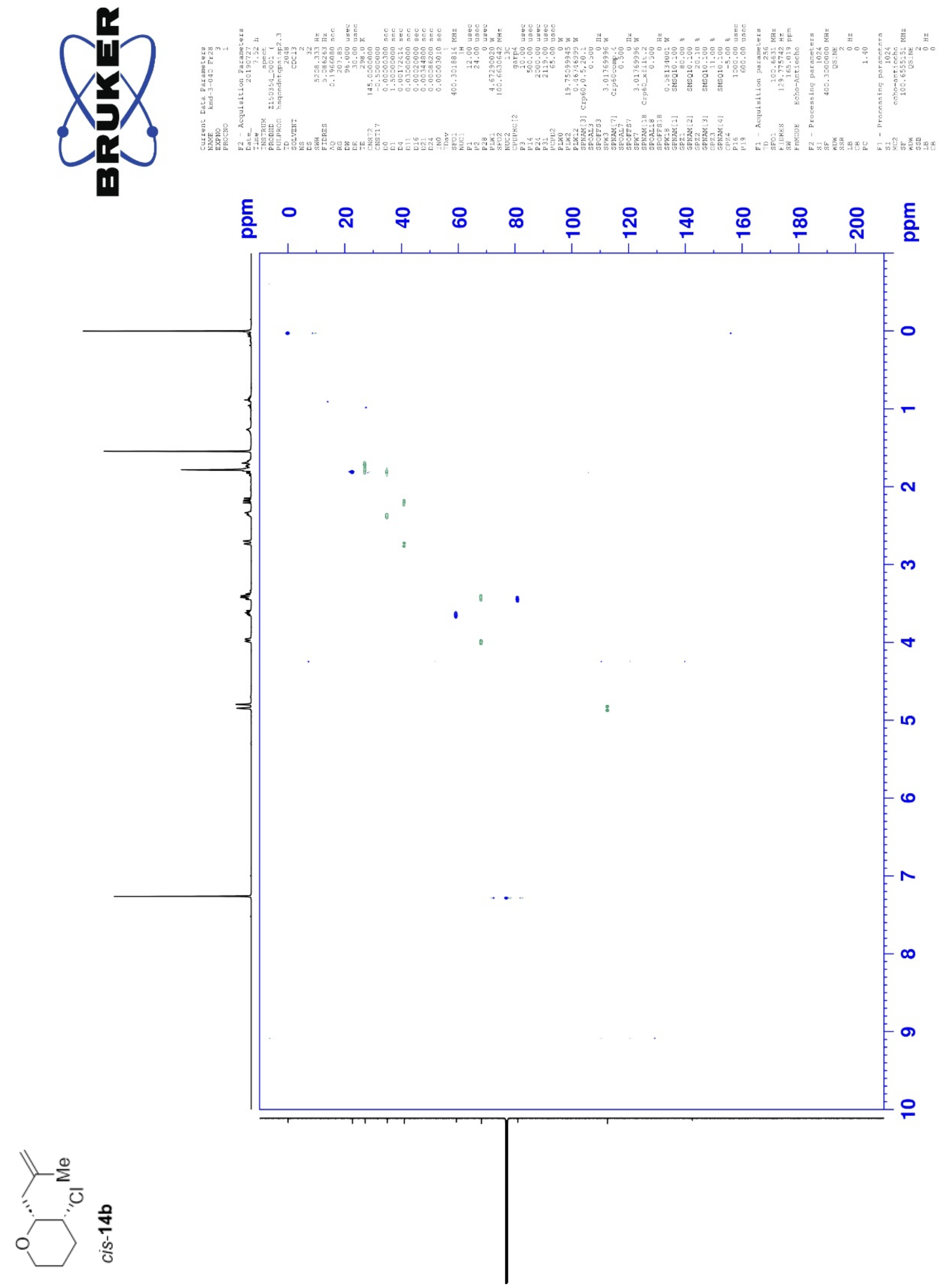

cis-**14b**

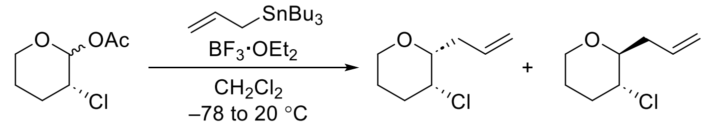

7 cis-14a trans-14a

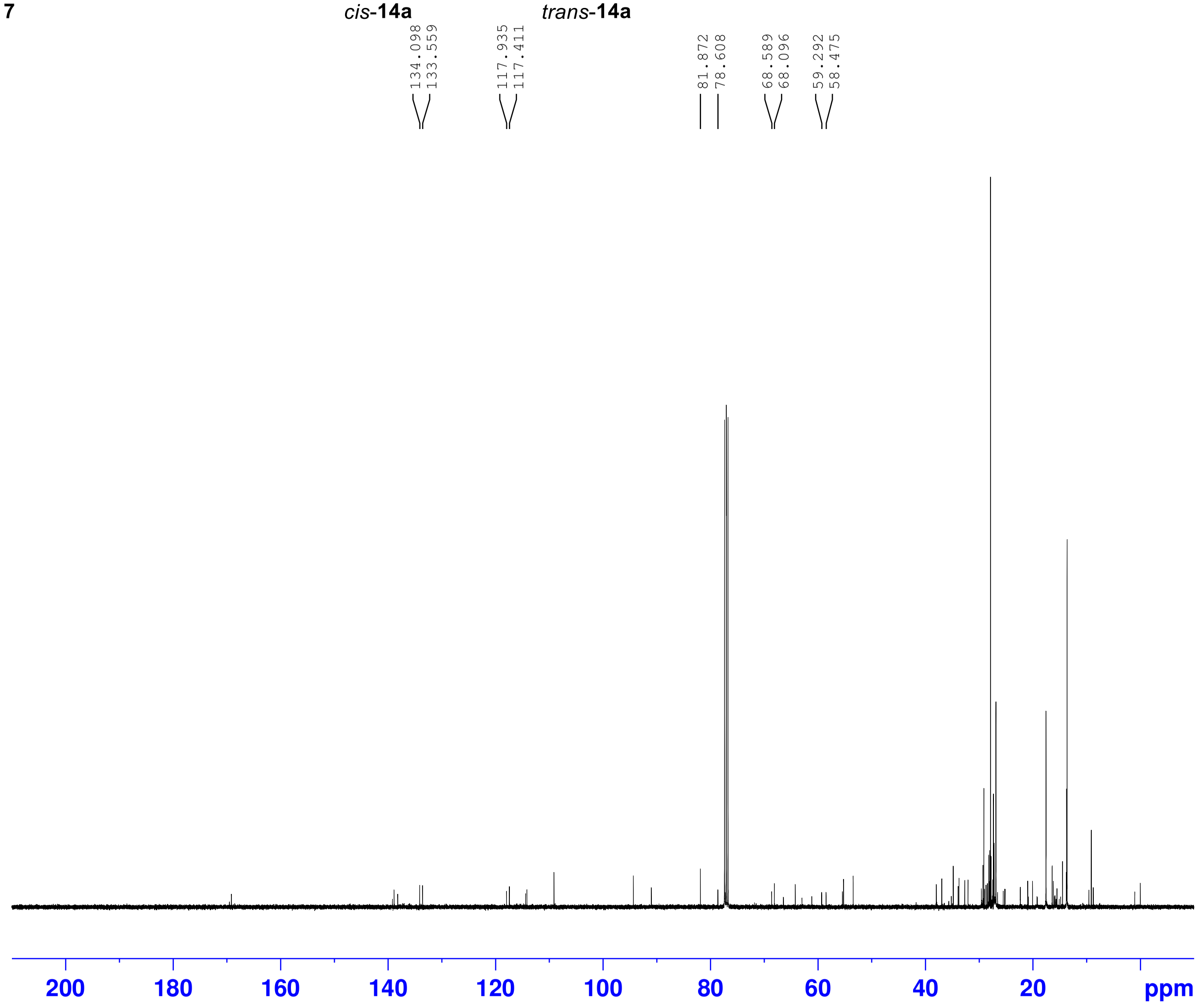


```
13C{1H}

Current Data Parameters
NAME            kmd-6-176
EXPNO                   3
PROCNO                  1

F2 - Acquisition Parameters
Date_            20220303
Time                10.52 h
INSTRUM             spect
PROBHD   Z150354_0001 (
PULPROG           zgpg30
TD                  65356
SOLVENT             CDCl3
NS                    256
DS                      4
SWH             24038.461 Hz
FIDRES           0.735616 Hz
AQ              1.3594048 sec
RG                  25.77
DW                 20.800 usec
DE                  25.00 usec
TE                  298.0 K
D1             2.00000000 sec
D11            0.03000000 sec
TD0                     1
SFO1          100.6655806 MHz
NUC1                  13C
P1                  10.00 usec
PLW1          17.99699974 W
SFO2          400.3016012 MHz
NUC2                   1H
CPDPRG[2          waltz16
PCPD2               80.00 usec
PLW2           4.64209986 W
PLW12          0.10445000 W
PLW13          0.05245300 W

F2 - Processing parameters
SI                 131072
SF            100.6555151 MHz
WDW                    EM
SSB      0
LB       0 Hz
GB       0
PC                   1.40
```

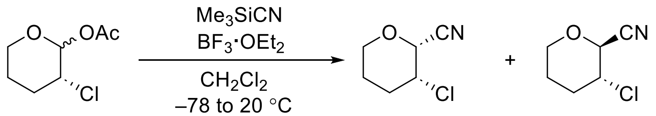

7 → *cis*-**14c** + *trans*-**14c**

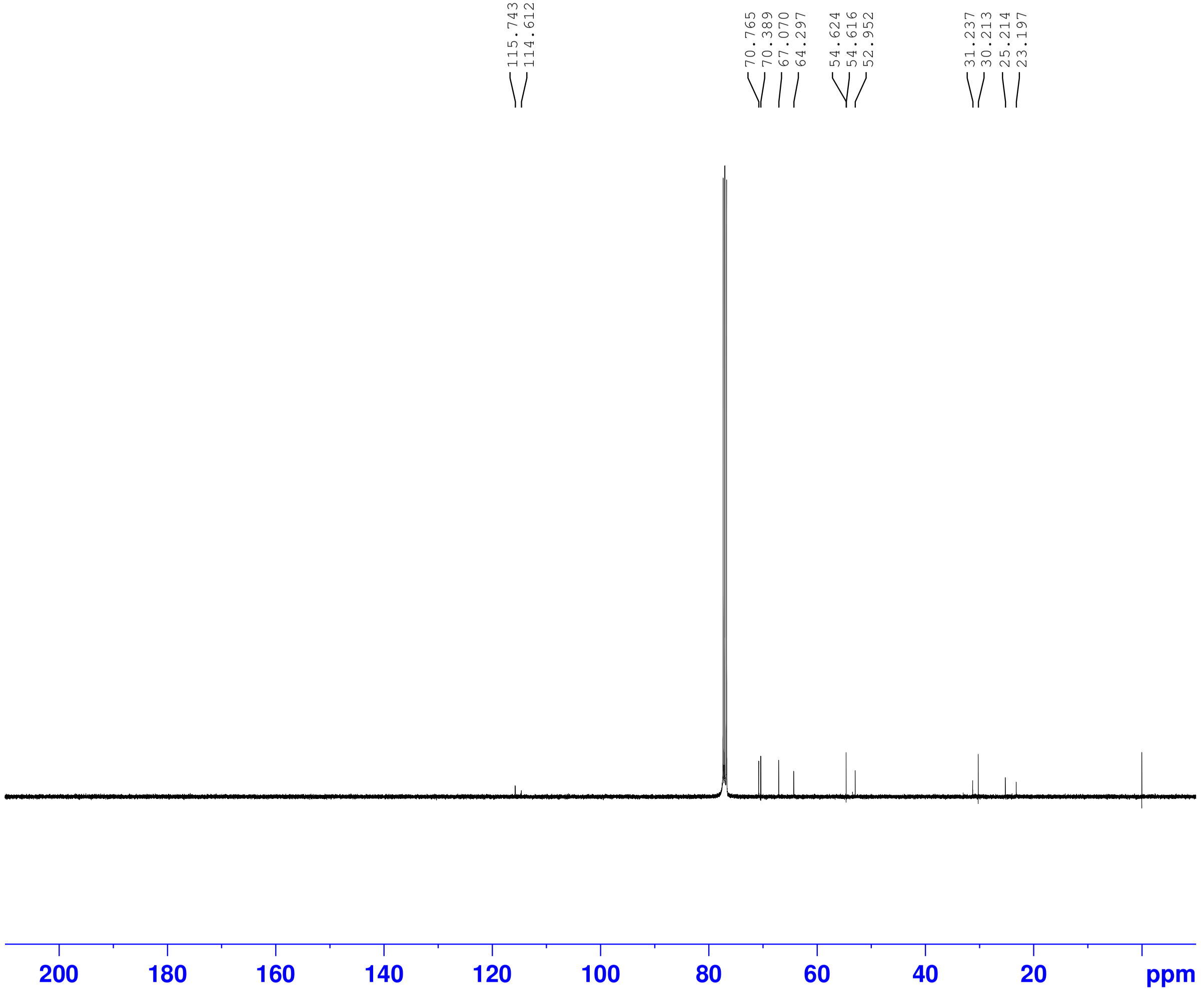

13C{1H}

Current Data Parameters
NAME kmd-6-206
EXPNO 4
PROCNO 1

F2 - Acquisition Parameters
Date_ 20220327
Time 2.03 h
INSTRUM spect
PROBHD Z150354_0001 (
PULPROG zgpg30
TD 65356
SOLVENT CDCl3
NS 1024
DS 4
SWH 24038.461 Hz
FIDRES 0.735616 Hz
AQ 1.3594048 sec
RG 25.77
DW 20.800 usec
DE 25.00 usec
TE 298.0 K
D1 2.00000000 sec
D11 0.03000000 sec
TD0 1
SFO1 100.6655806 MHz
NUC1 13C
P1 10.00 usec
PLW1 17.99699974 W
SFO2 400.3016012 MHz
NUC2 1H
CPDPRG[2 waltz16
PCPD2 80.00 usec
PLW2 4.64209986 W
PLW12 0.10445000 W
PLW13 0.05245300 W

F2 - Processing parameters
SI 131072
SF 100.6555156 MHz
WDW EM
SSB 0
LB 0 Hz
GB 0
PC 1.40

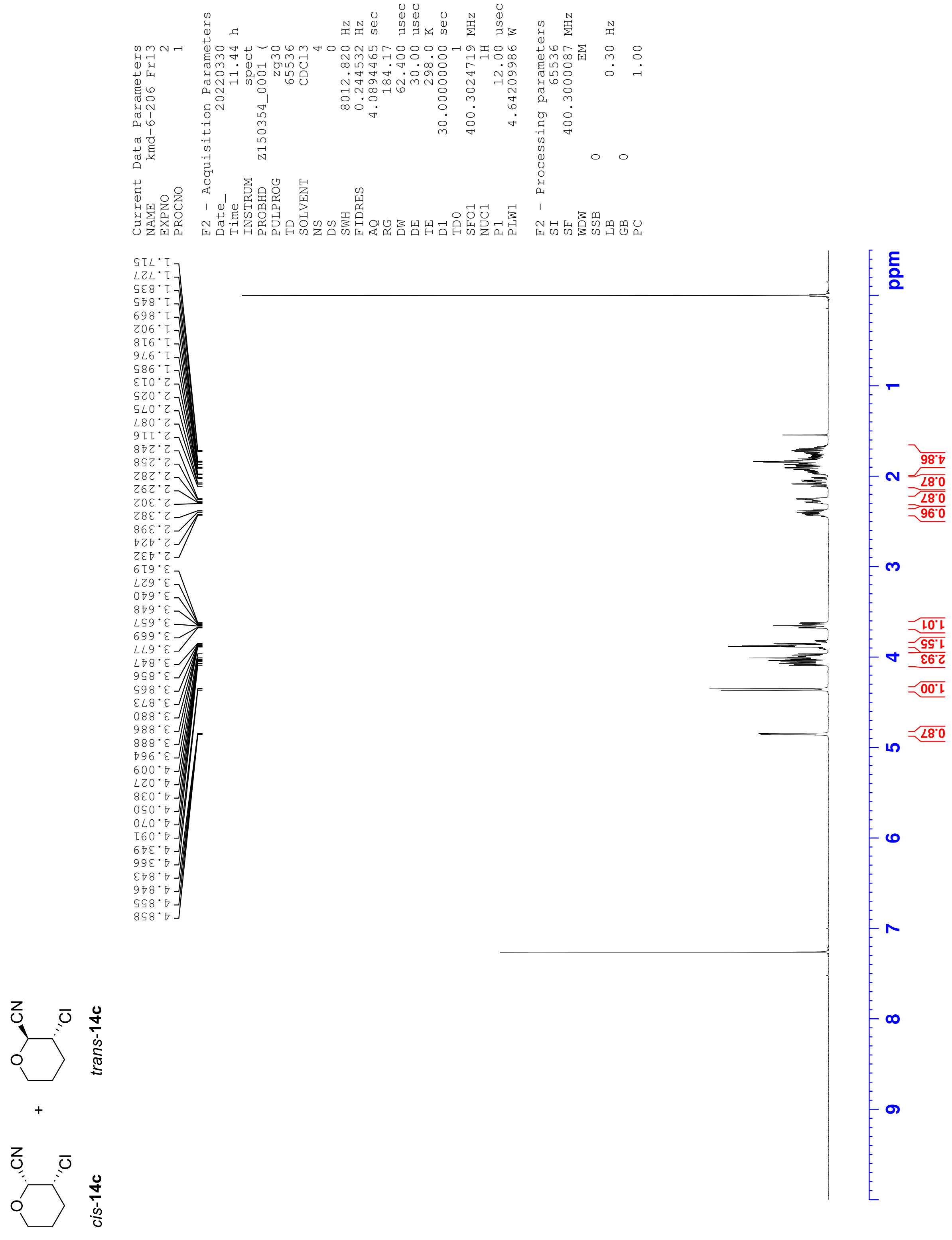
cis-14c
trans-14c
CN
Cl
O
+
Current Data Parameters
NAME kmd-6-206 Fr13
EXPNO 2
PROCNO 1
F2 - Acquisition Parameters
Date_ 20220330
Time 11.44 h
INSTRUM spect
PROBHD Z150354_0001 (
PULPROG zg30
TD 65536
SOLVENT CDCl3
NS 4
DS 0
SWH 8012.820 Hz
FIDRES 0.244532 Hz
AQ 4.0894465 sec
RG 184.17
DW 62.400 usec
DE 30.00 usec
TE 298.0 K
D1 30.00000000 sec
TD0 1
SFO1 400.3024719 MHz
NUC1 1H
P1 12.00 usec
PLW1 4.64209986 W
F2 - Processing parameters
SI 65536
SF 400.3000087 MHz
WDW EM
SSB 0
LB 0.30 Hz
GB 0
PC 1.00
1.715
1.727
1.835
1.845
1.869
1.902
1.918
1.976
1.985
2.013
2.025
2.075
2.087
2.116
2.248
2.258
2.282
2.292
2.302
2.382
2.398
2.424
2.432
3.619
3.627
3.640
3.648
3.657
3.669
3.677
3.847
3.856
3.865
3.873
3.880
3.886
3.888
3.964
4.009
4.027
4.038
4.050
4.070
4.091
4.349
4.366
4.843
4.846
4.855
4.858
4.86
0.87
0.87
0.96
1.01
1.55
2.93
1.00
0.87
ppm
1
2
3
4
5
6
7
8
9

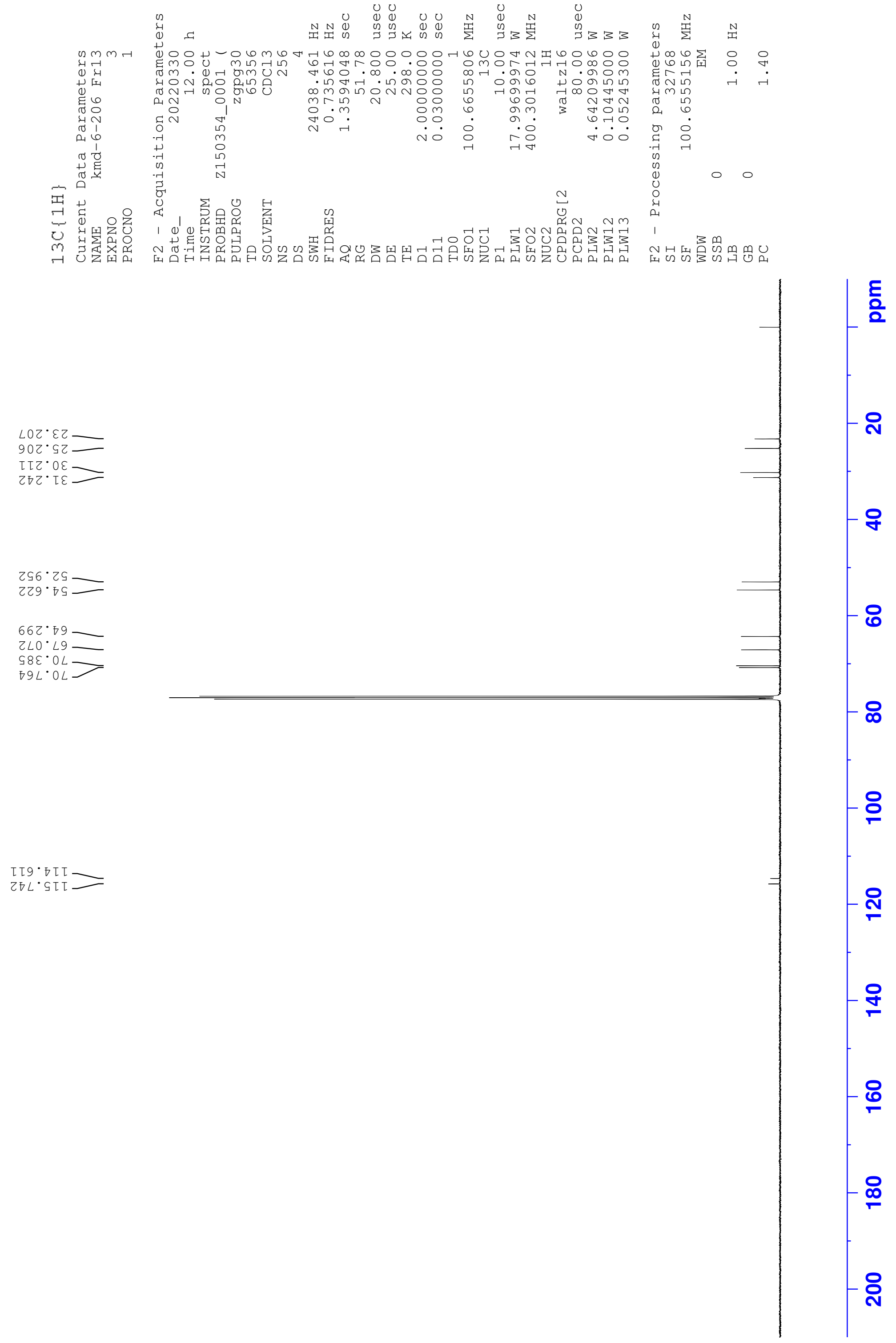

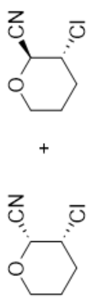

cis-**14c** *trans*-**14c**

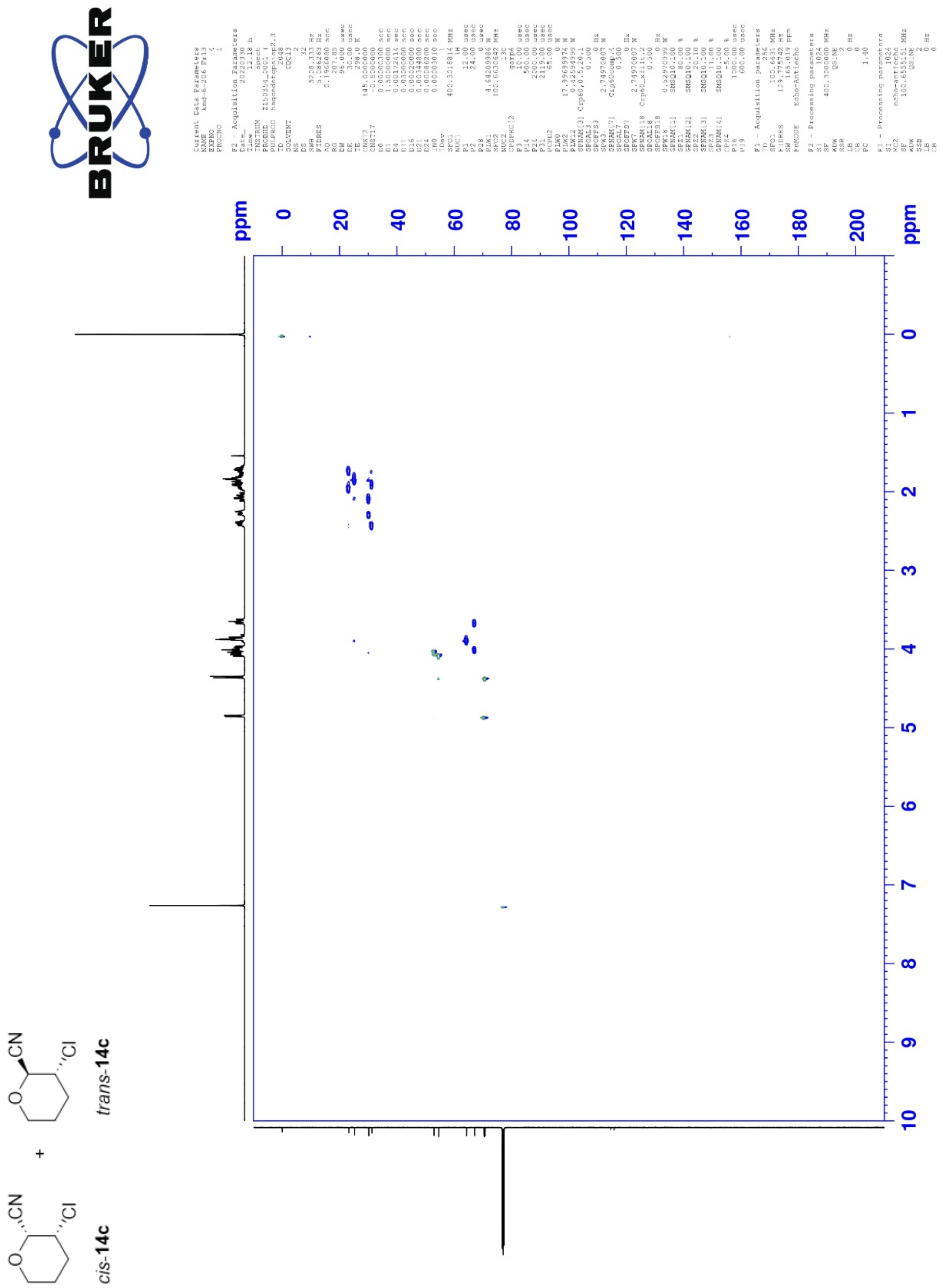
cis-14c
trans-14c
ppm

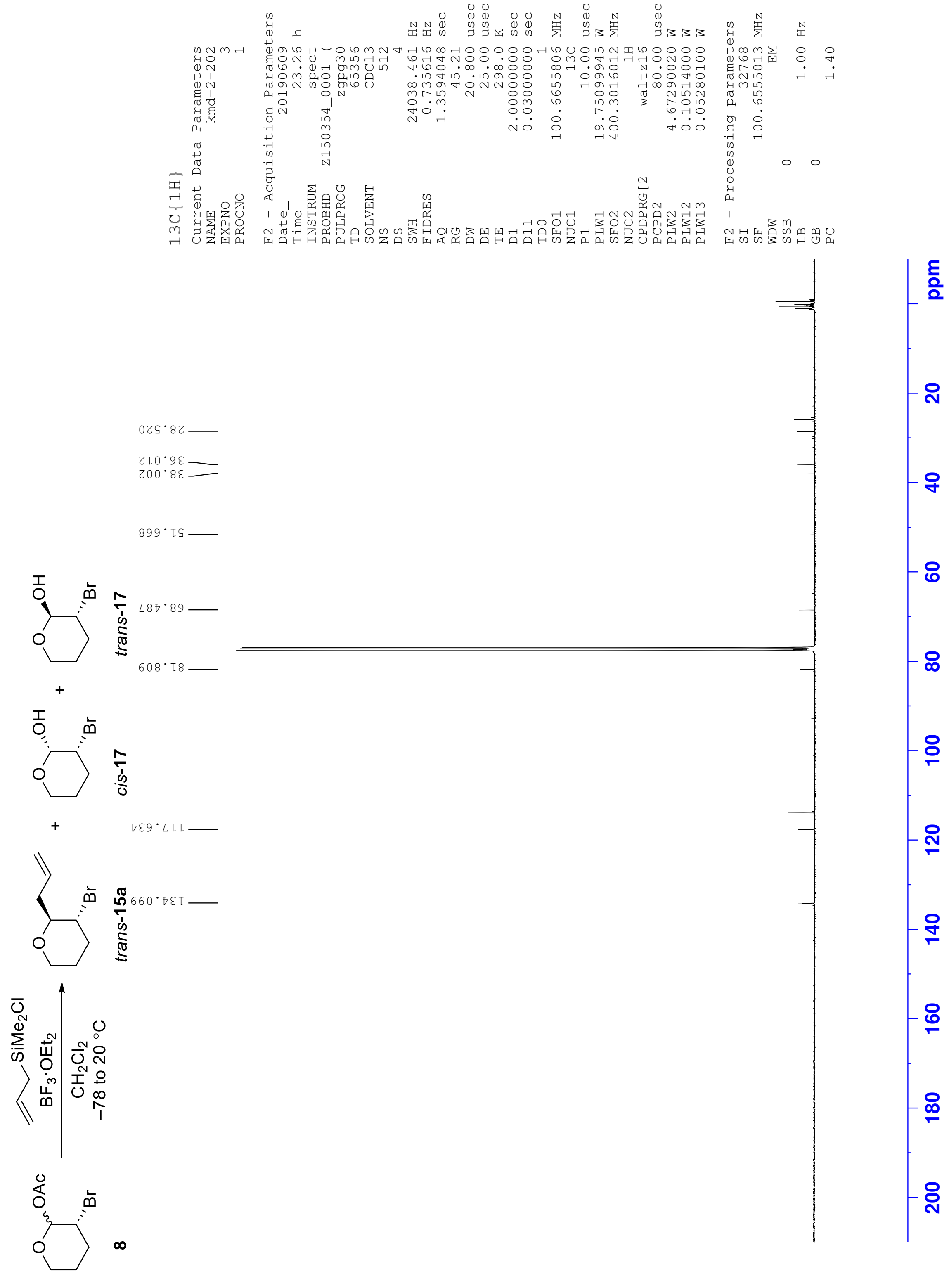
SiMe2Cl
BF3·OEt2
CH2Cl2
–78 to 20 °C
OAc
Br
8
trans-15a
OH
cis-17
trans-17
134.099
117.634
81.809
68.487
51.668
38.002
36.012
28.520
13C{1H}
Current Data Parameters
NAME kmd-2-202
EXPNO 3
PROCNO 1
F2 - Acquisition Parameters
Date_ 20190609
Time 23.26 h
INSTRUM spect
PROBHD Z150354_0001 (
PULPROG zgpg30
TD 65356
SOLVENT CDCl3
NS 512
DS 4
SWH 24038.461 Hz
FIDRES 0.735616 Hz
AQ 1.3594048 sec
RG 45.21
DW 20.800 usec
DE 25.00 usec
TE 298.0 K
D1 2.00000000 sec
D11 0.03000000 sec
TD0 1
SFO1 100.6655806 MHz
NUC1 13C
P1 10.00 usec
PLW1 19.75099945 W
SFO2 400.3016012 MHz
NUC2 1H
CPDPRG[2 waltz16
PCPD2 80.00 usec
PLW2 4.67290020 W
PLW12 0.10514000 W
PLW13 0.05280100 W
F2 - Processing parameters
SI 32768
SF 100.6555013 MHz
WDW EM
SSB 0
LB 1.00 Hz
GB 0
PC 1.40
200
180
160
140
120
100
80
60
40
20
ppm

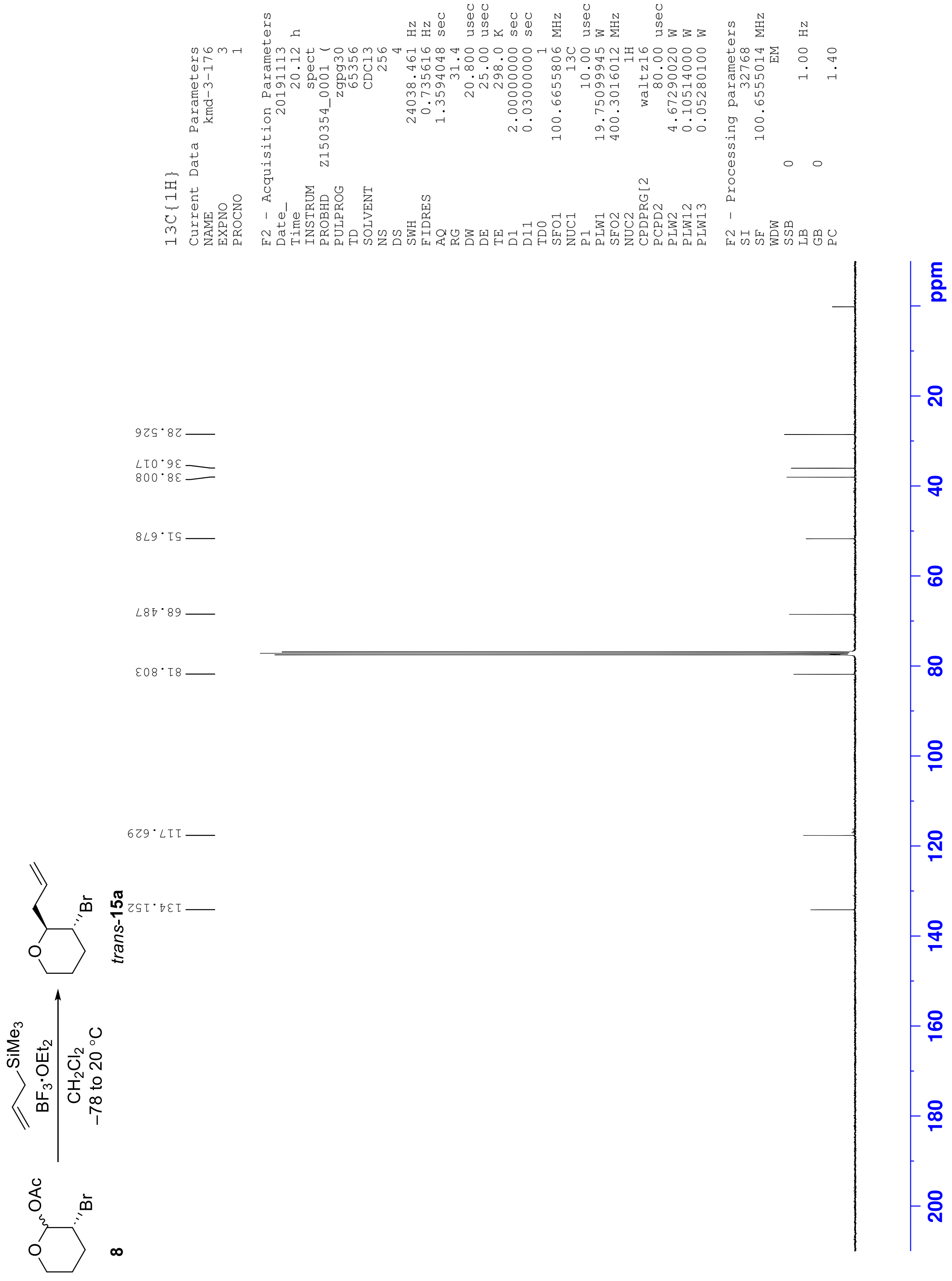
OAc
Br
8
SiMe3
BF3·OEt2
CH2Cl2
−78 to 20 °C
Br
trans-15a
134.152
117.629
81.803
68.487
51.678
38.008
36.017
28.526
13C{1H}
Current Data Parameters
NAME kmd-3-176
EXPNO 3
PROCNO 1
F2 - Acquisition Parameters
Date_ 20191113
Time_ 20.12 h
INSTRUM spect
PROBHD Z150354_0001 (
PULPROG zgpg30
TD 65356
SOLVENT CDCl3
NS 256
DS 4
SWH 24038.461 Hz
FIDRES 0.735616 Hz
AQ 1.3594048 sec
RG 31.4
DW 20.800 usec
DE 25.00 usec
TE 298.0 K
D1 2.00000000 sec
D11 0.03000000 sec
TD0 1
SFO1 100.6655806 MHz
NUC1 13C
P1 10.00 usec
PLW1 19.75099945 W
SFO2 400.3016012 MHz
NUC2 1H
CPDPRG[2 waltz16
PCPD2 80.00 usec
PLW2 4.67290020 W
PLW12 0.10514000 W
PLW13 0.05280100 W
F2 - Processing parameters
SI 32768
SF 100.6555014 MHz
WDW EM
SSB 0
LB 1.00 Hz
GB 0
PC 1.40
ppm
20
40
60
80
100
120
140
160
180
200

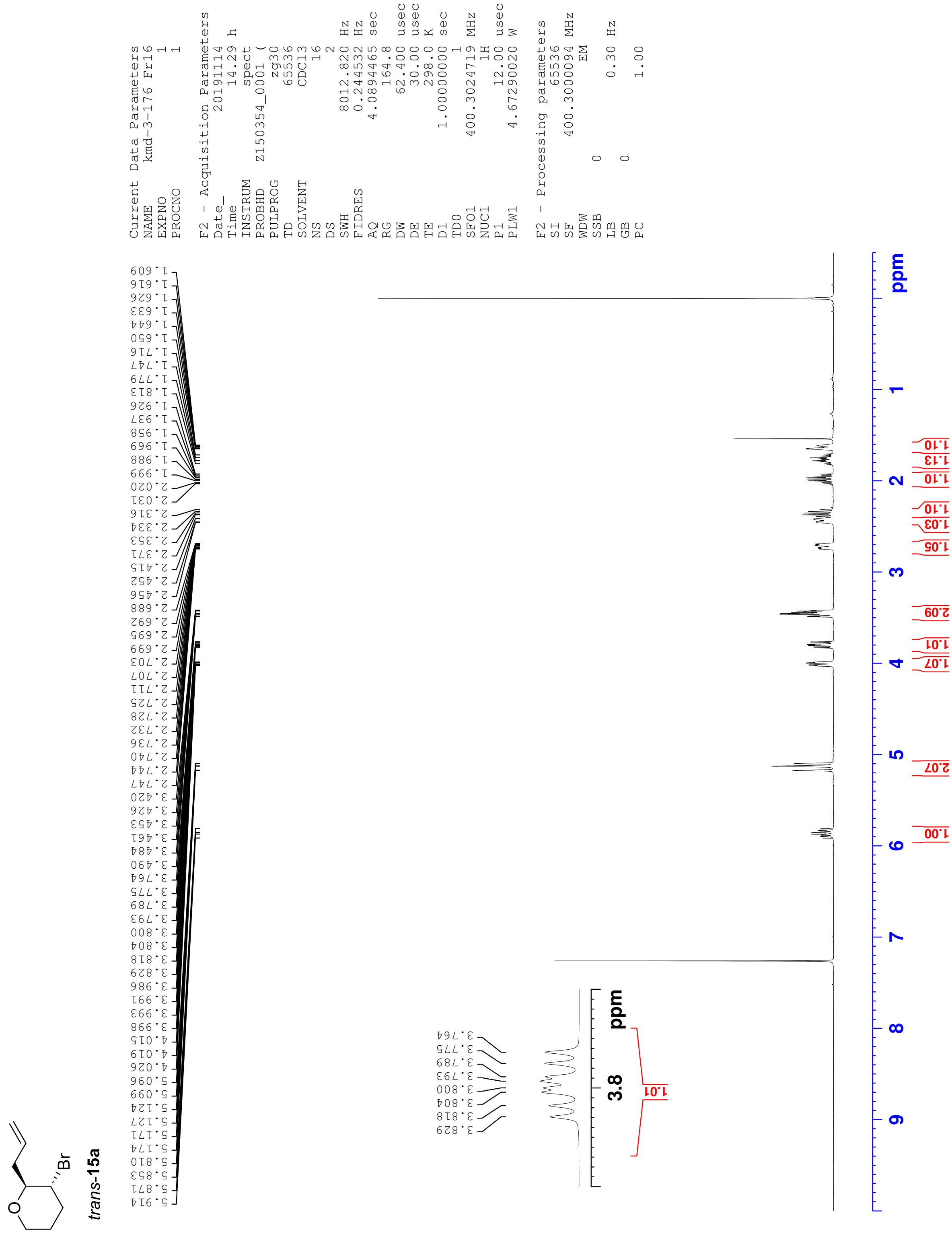
Current Data Parameters
NAME kmd-3-176 Fr16
EXPNO 1
PROCNO 1
F2 - Acquisition Parameters
Date_ 20191114
Time 14.29 h
INSTRUM spect
PROBHD Z150354_0001 (
PULPROG zg30
TD 65536
SOLVENT CDCl3
NS 16
DS 2
SWH 8012.820 Hz
FIDRES 0.244532 Hz
AQ 4.0894465 sec
RG 164.8
DW 62.400 usec
DE 30.00 usec
TE 298.0 K
D1 1.00000000 sec
TD0 1
SFO1 400.3024719 MHz
NUC1 1H
P1 12.00 usec
PLW1 4.67290020 W
F2 - Processing parameters
SI 65536
SF 400.3000094 MHz
WDW EM
SSB 0
LB 0.30 Hz
GB 0
PC 1.00
Br
ppm
3.8
1.01

trans-15a

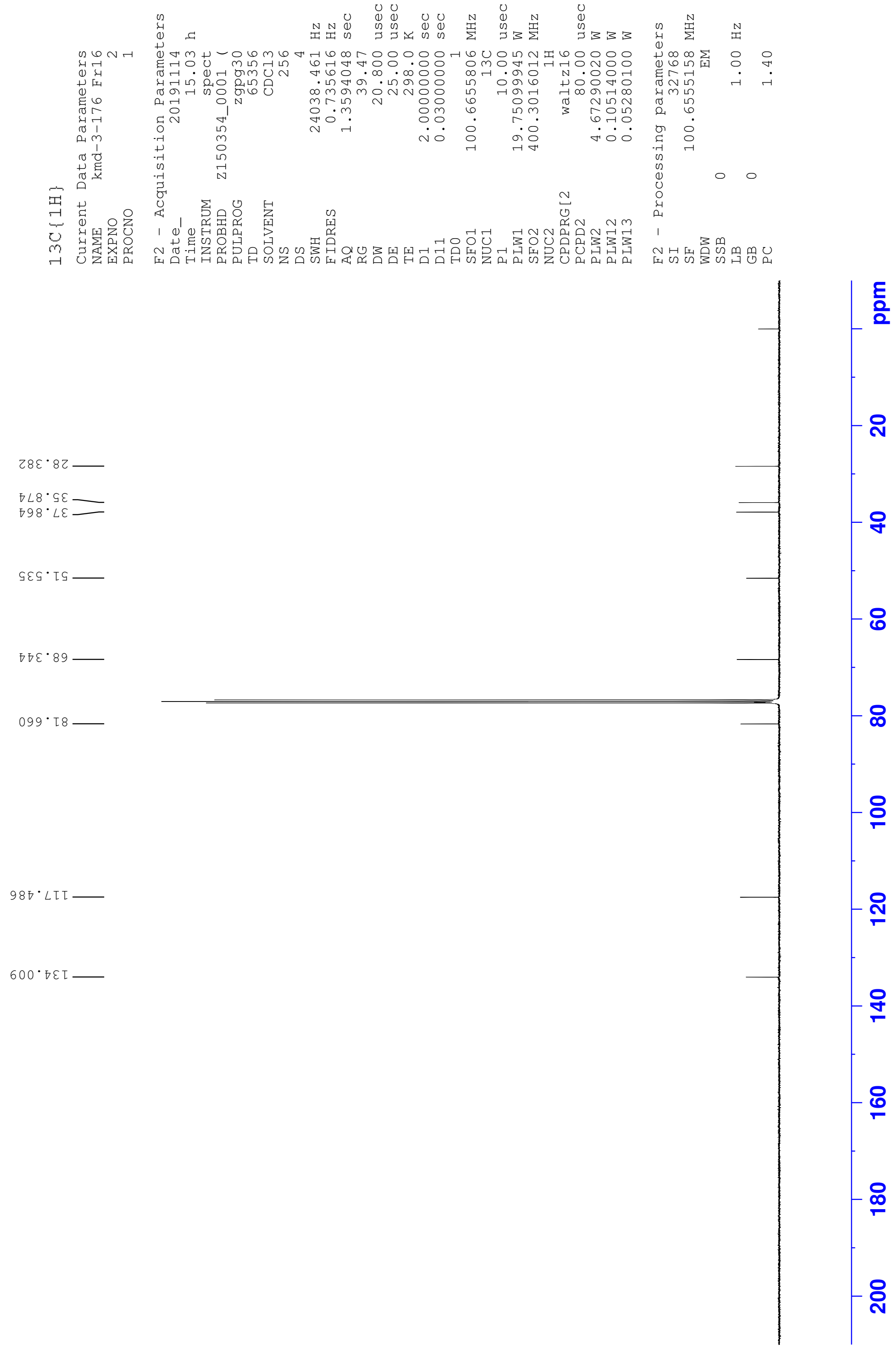
13C{1H}
Current Data Parameters
NAME kmd-3-176 Fr16
EXPNO 2
PROCNO 1
F2 - Acquisition Parameters
Date_ 20191114
Time_ 15.03 h
INSTRUM spect
PROBHD Z150354_0001 (
PULPROG zgpg30
TD 65536
SOLVENT CDCl3
NS 256
DS 4
SWH 24038.461 Hz
FIDRES 0.733616 Hz
AQ 1.3594048 sec
RG 39.47
DW 20.800 usec
DE 25.00 usec
TE 298.0 K
D1 2.00000000 sec
D11 0.03000000 sec
TD0 1
SFO1 100.6655806 MHz
NUC1 13C
P1 10.00 usec
PLW1 19.75099945 W
SFO2 400.3016012 MHz
NUC2 1H
CPDPRG[2 waltz16
PCPD2 80.00 usec
PLW2 4.67290020 W
PLW12 0.10514000 W
PLW13 0.05280100 W
F2 - Processing parameters
SI 32768
SF 100.6555158 MHz
WDW EM
SSB 0
LB 1.00 Hz
GB 0
PC 1.40
28.382
35.874
37.864
51.535
68.344
81.660
117.486
134.009
ppm
20
40
60
80
100
120
140
160
180
200

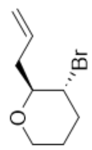
O
Br

trans-**15a**

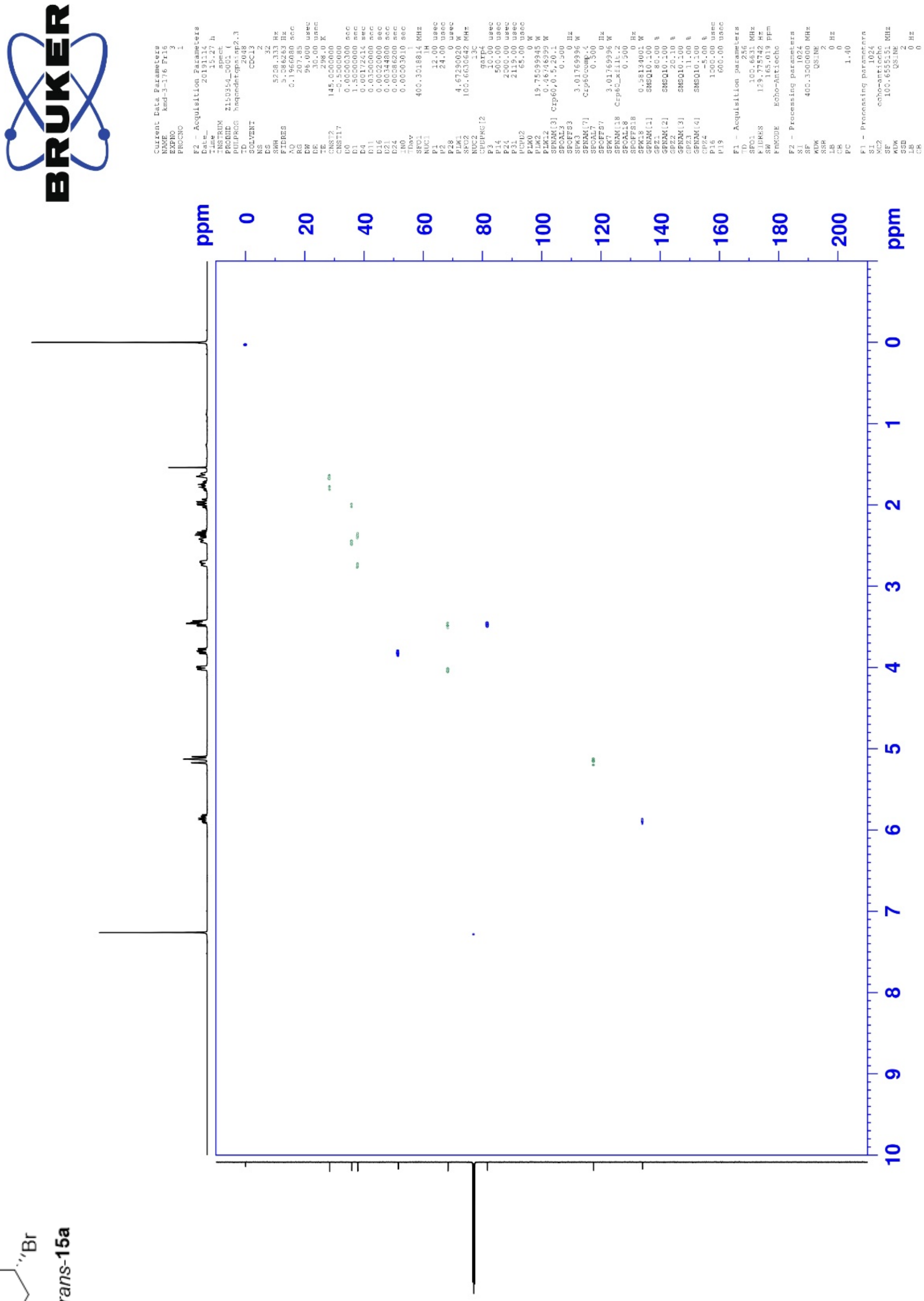

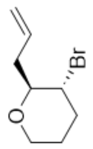

trans-**15a**

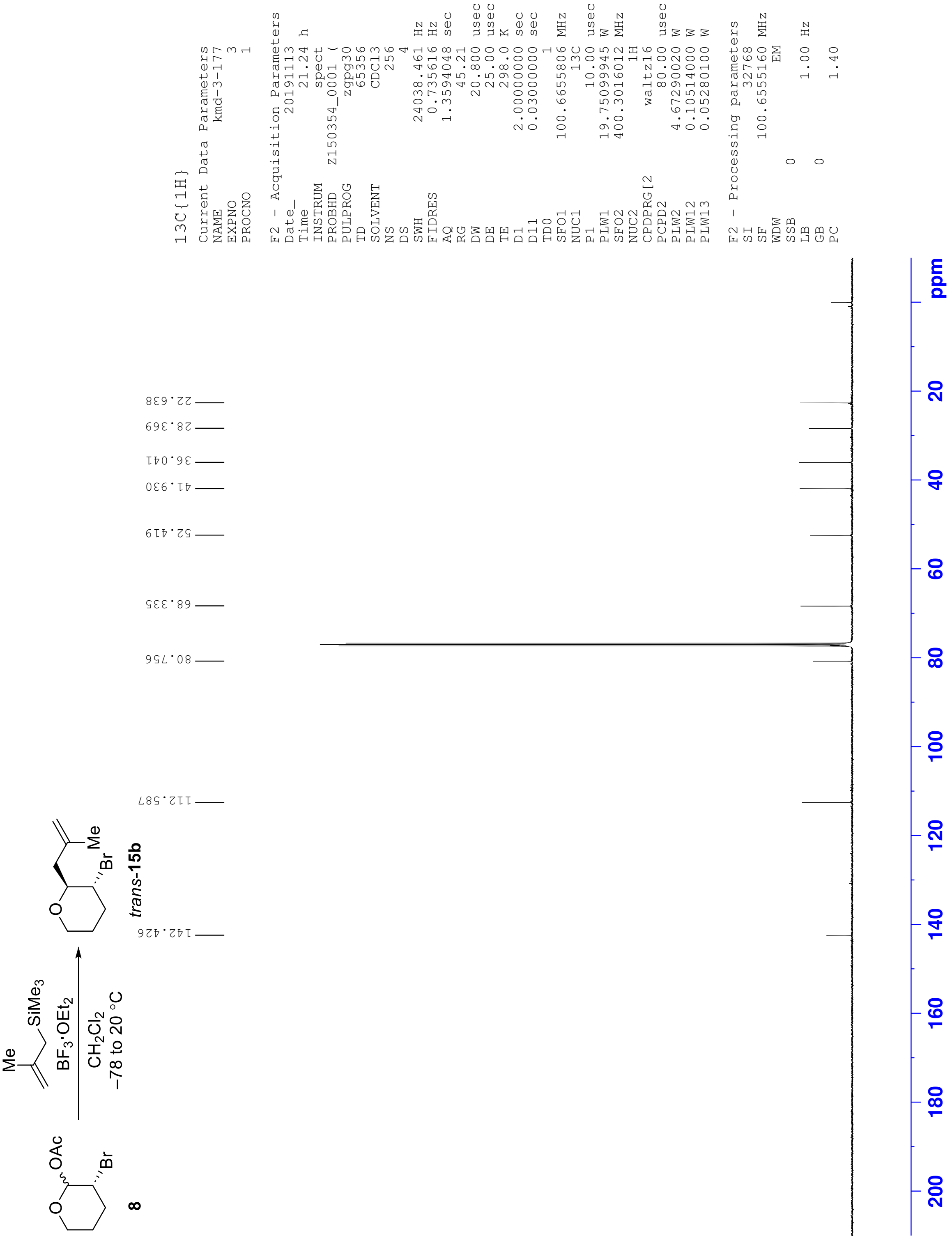
8
Me
SiMe3
BF3·OEt2
CH2Cl2
−78 to 20 °C
OAc
Br
O
Me
trans-15b
22.638
28.369
36.041
41.930
52.419
68.335
80.756
112.587
142.426
13C{1H}
Current Data Parameters
NAME kmd-3-177
EXPNO 3
PROCNO 1
F2 - Acquisition Parameters
Date_ 20191113
Time_ 21.24 h
INSTRUM spect
PROBHD Z150354_0001 (
PULPROG zgpg30
TD 65356
SOLVENT CDCl3
NS 256
DS 4
SWH 24038.461 Hz
FIDRES 0.735616 Hz
AQ 1.3594048 sec
RG 45.21
DW 20.800 usec
DE 25.00 usec
TE 298.0 K
D1 2.00000000 sec
D11 0.03000000 sec
TD0 1
SFO1 100.6655806 MHz
NUC1 13C
P1 10.00 usec
PLW1 19.75099945 W
SFO2 400.3016012 MHz
NUC2 1H
CPDPRG[2 waltz16
PCPD2 80.00 usec
PLW2 4.67290020 W
PLW12 0.10514000 W
PLW13 0.05280100 W
F2 - Processing parameters
SI 32768
SF 100.6555160 MHz
WDW EM
SSB 0
LB 1.00 Hz
GB 0
PC 1.40
ppm
20
40
60
80
100
120
140
160
180
200

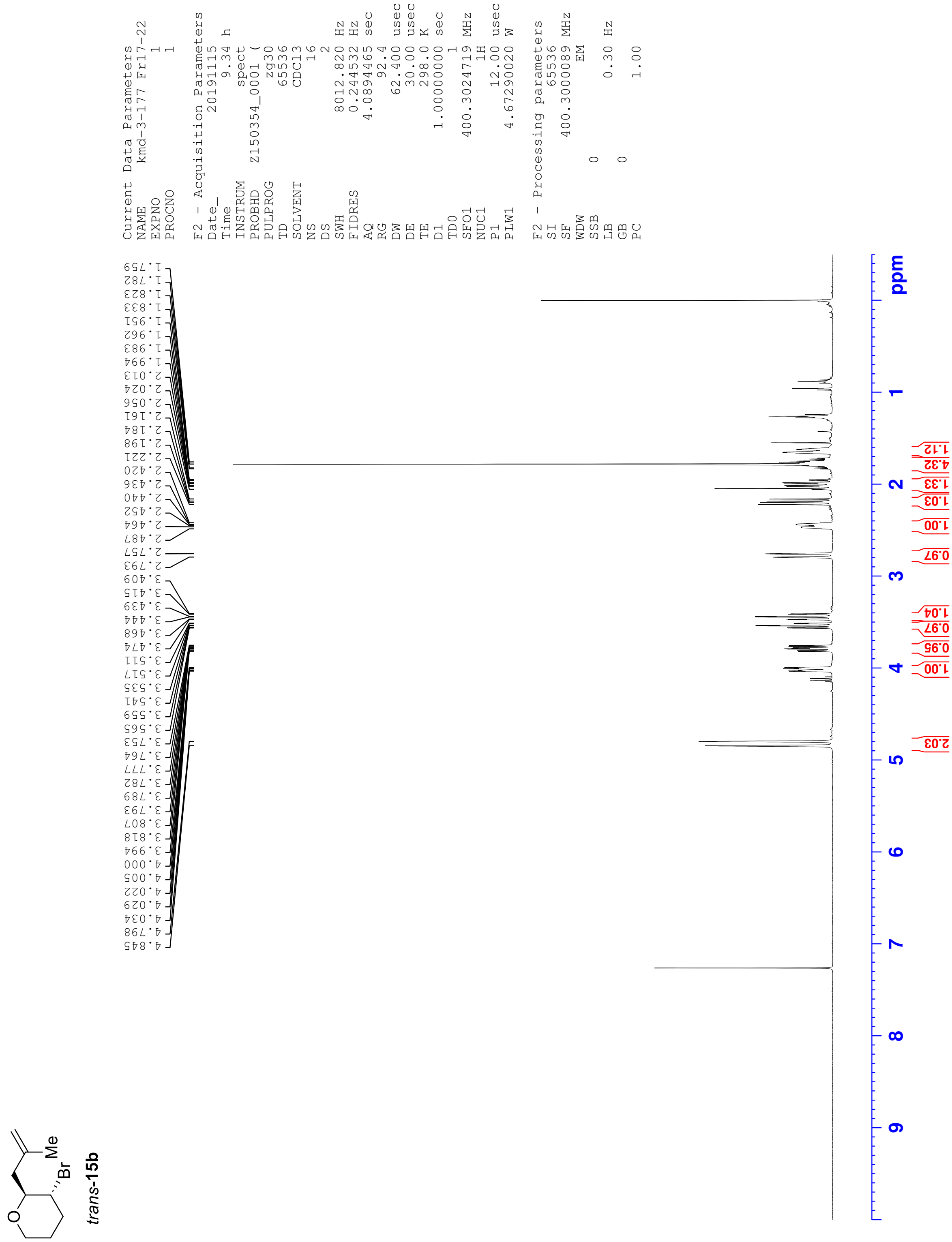
Current Data Parameters
NAME kmd-3-177 Fr17-22
EXPNO 1
PROCNO 1
F2 - Acquisition Parameters
Date_ 20191115
Time 9.34 h
INSTRUM spect
PROBHD Z150354_0001 (
PULPROG zg30
TD 65536
SOLVENT CDCl3
NS 16
DS 2
SWH 8012.820 Hz
FIDRES 0.244532 Hz
AQ 4.0894465 sec
RG 92.4
DW 62.400 usec
DE 30.00 usec
TE 298.0 K
D1 1.00000000 sec
TD0 1
SFO1 400.3024719 MHz
NUC1 1H
P1 12.00 usec
PLW1 4.67290020 W
F2 - Processing parameters
SI 65536
SF 400.3000089 MHz
WDW EM
SSB 0
LB 0.30 Hz
GB 0
PC 1.00
ppm
Me
Br
O

trans-**15b**

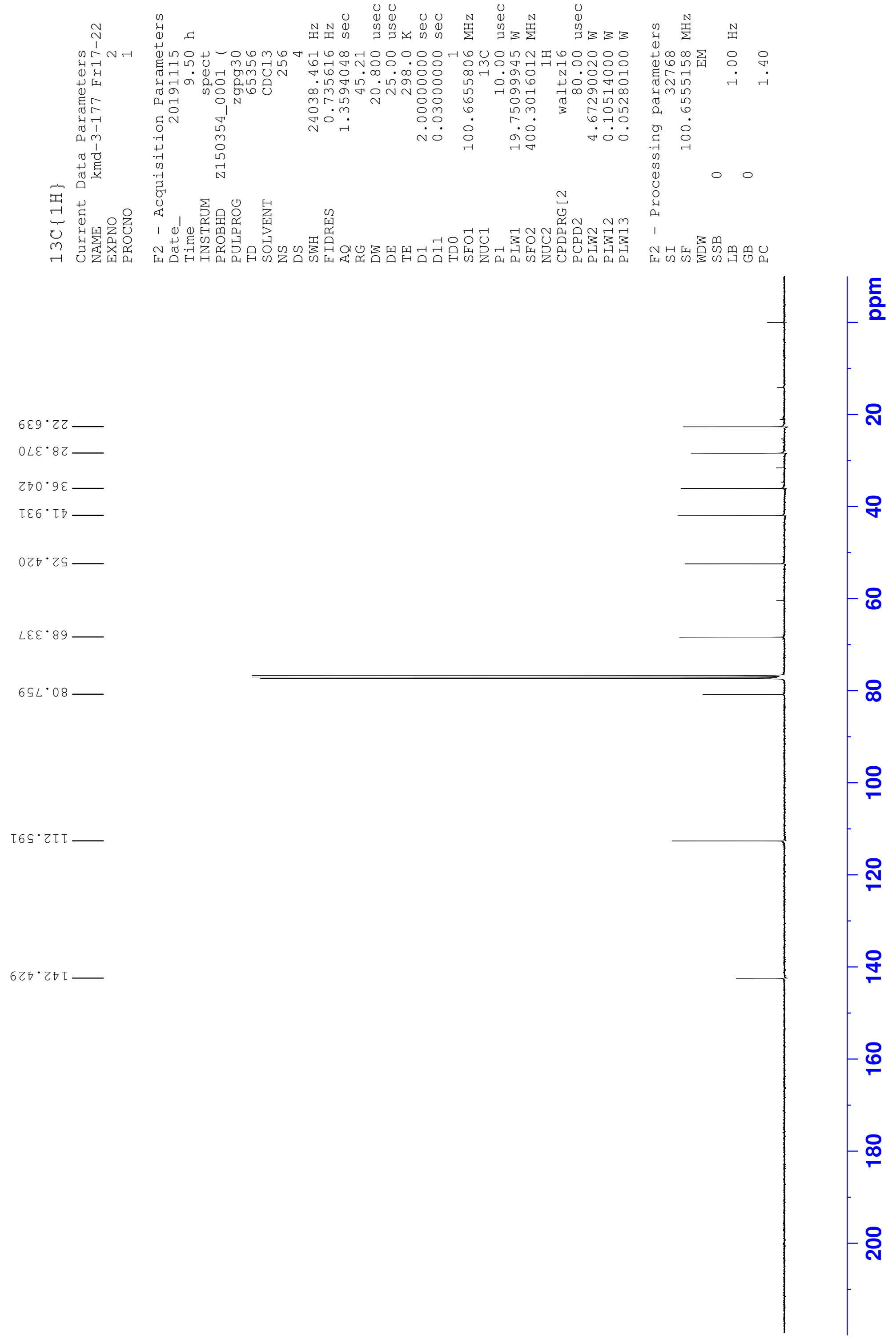
13C{1H}
Current Data Parameters
NAME kmd-3-177 Fr17-22
EXPNO 2
PROCNO 1
F2 - Acquisition Parameters
Date_ 20191115
Time_ 9.50 h
INSTRUM spect
PROBHD Z150354_0001 (
PULPROG zgpg30
TD 65356
SOLVENT CDCl3
NS 256
DS 4
SWH 24038.461 Hz
FIDRES 0.735616 Hz
AQ 1.3594048 sec
RG 45.21
DW 20.800 usec
DE 25.00 usec
TE 298.0 K
D1 2.00000000 sec
D11 0.03000000 sec
TD0 1
SFO1 100.6655806 MHz
NUC1 13C
P1 10.00 usec
PLW1 19.75099945 W
SFO2 400.3016012 MHz
NUC2 1H
CPDPRG[2 waltz16
PCPD2 80.00 usec
PLW2 4.67290020 W
PLW12 0.10514000 W
PLW13 0.05280100 W
F2 - Processing parameters
SI 32768
SF 100.6555158 MHz
WDW EM
SSB 0
LB 1.00 Hz
GB 0
PC 1.40
22.639
28.370
36.042
41.931
52.420
68.337
80.759
112.591
142.429
ppm
20
40
60
80
100
120
140
160
180
200

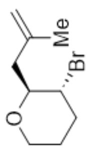
O
Me
Br

trans-**15b**

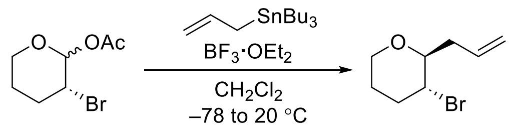

8 *trans*-**15a**

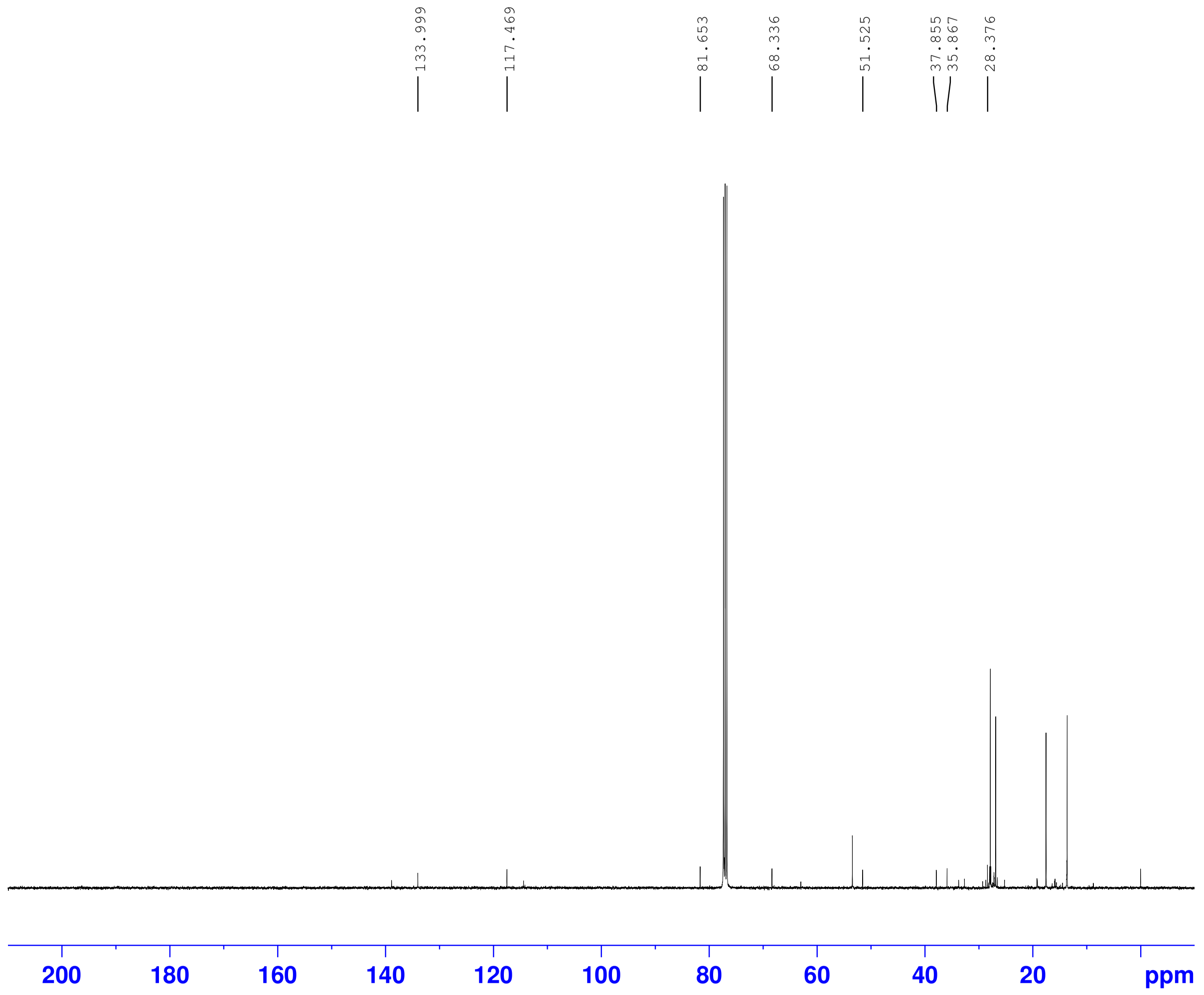


```
13C{1H}

Current Data Parameters
NAME            kmd-6-157
EXPNO                   3
PROCNO                  1

F2 - Acquisition Parameters
Date_            20220215
Time                14.22 h
INSTRUM             spect
PROBHD   Z150354_0001 (
PULPROG           zgpg30
TD                  65356
SOLVENT             CDCl3
NS                    256
DS                      4
SWH             24038.461 Hz
FIDRES           0.735616 Hz
AQ              1.3594048 sec
RG                  29.21
DW                 20.800 usec
DE                  25.00 usec
TE                  298.0 K
D1             2.00000000 sec
D11            0.03000000 sec
TD0                     1
SFO1          100.6655806 MHz
NUC1                  13C
P1                  10.00 usec
PLW1          17.99699974 W
SFO2          400.3016012 MHz
NUC2                   1H
CPDPRG[2          waltz16
PCPD2               80.00 usec
PLW2           4.64209986 W
PLW12          0.10445000 W
PLW13          0.05245300 W

F2 - Processing parameters
SI                  32768
SF            100.6555165 MHz
WDW                    EM
SSB      0
LB                   1.00 Hz
GB       0
PC                   1.40
```

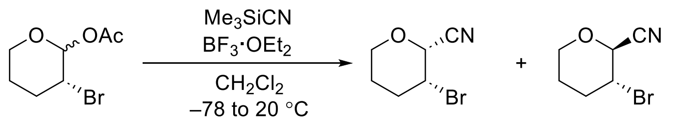

8 *cis*-**15c** *trans*-**15c**

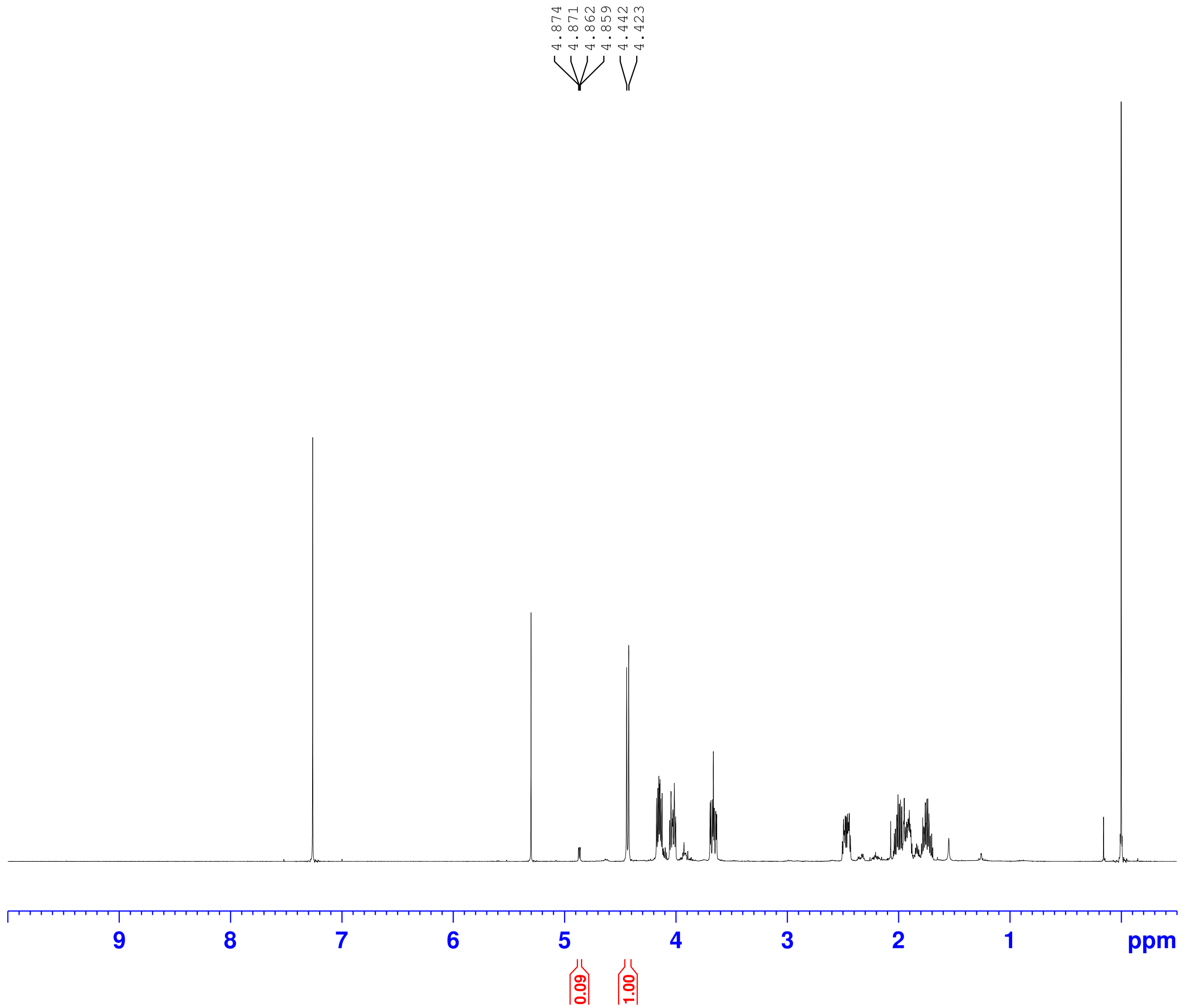


```
Current Data Parameters
NAME          kmd-7-002
EXPNO                 2
PROCNO                1

F2 - Acquisition Parameters
Date_          20220330
Time              10.44 h
INSTRUM           spect
PROBHD   Z150354_0001 (
PULPROG            zg30
TD                65536
SOLVENT           CDCl3
NS                    4
DS                    0
SWH            8012.820 Hz
FIDRES         0.244532 Hz
AQ            4.0894465 sec
RG               141.61
DW               62.400 usec
DE                30.00 usec
TE                298.0 K
D1          30.00000000 sec
TD0                   1
SFO1        400.3024719 MHz
NUC1                 1H
P1                12.00 usec
PLW1         4.64209986 W

F2 - Processing parameters
SI                65536
SF          400.3000087 MHz
WDW                  EM
SSB       0
LB                 0.30 Hz
GB        0
PC                 1.00
```

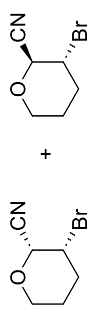

cis-**15c** *trans*-**15c**

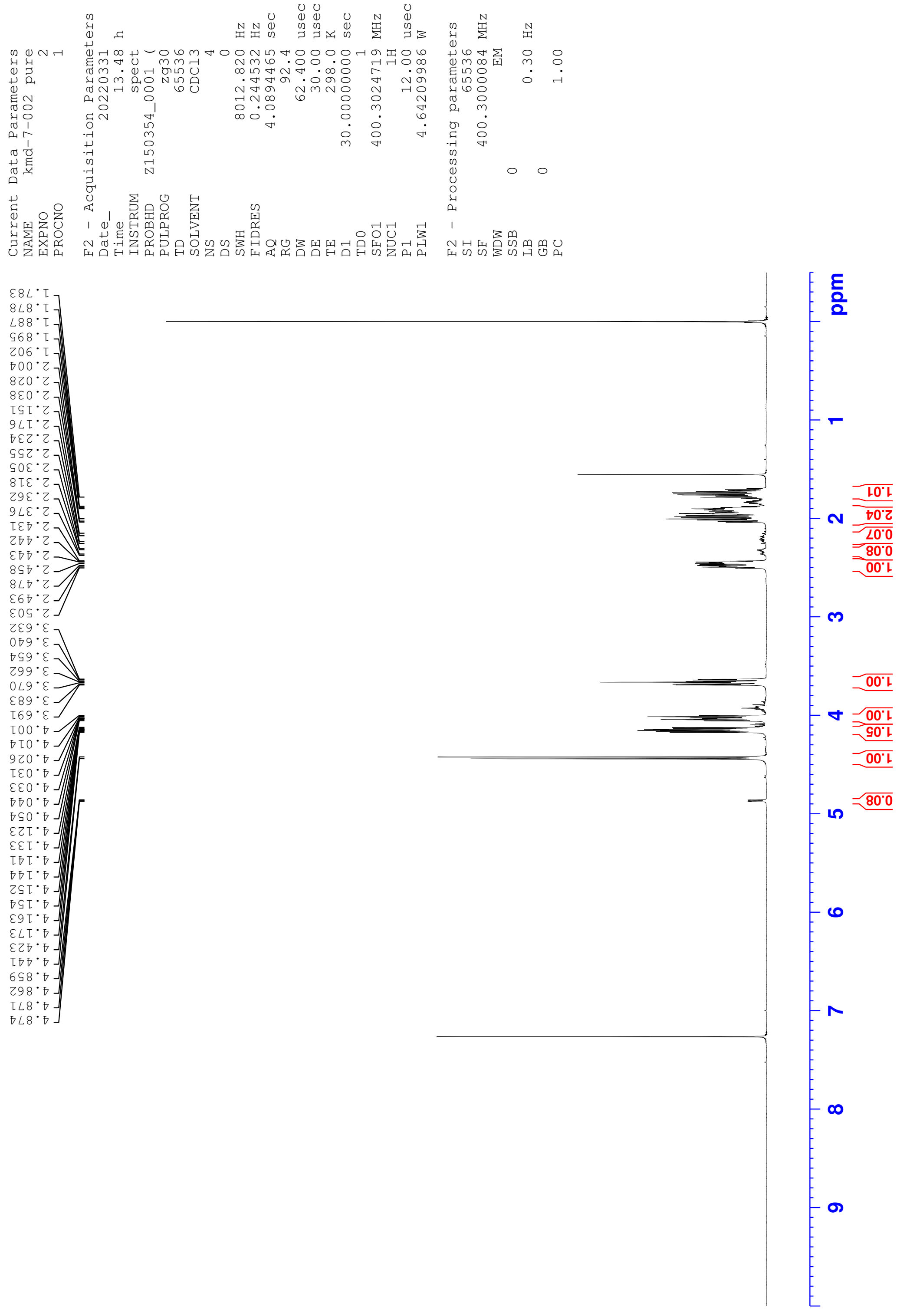

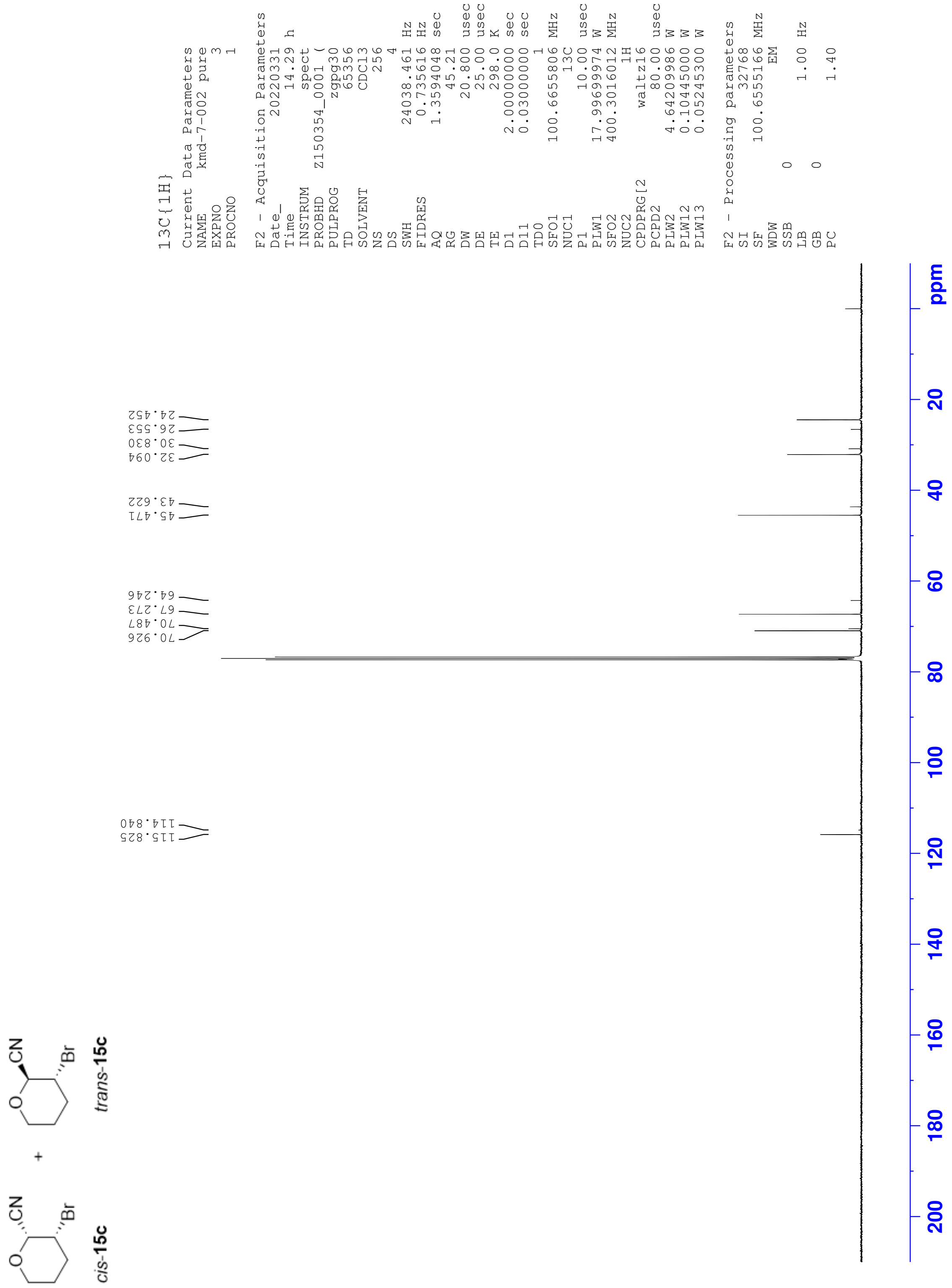
cis-15c
trans-15c
13C{1H}
Current Data Parameters
NAME kmd-7-002 pure
EXPNO 3
PROCNO 1
F2 - Acquisition Parameters
Date_ 20220331
Time_ 14.29 h
INSTRUM spect
PROBHD Z150354_0001 (
PULPROG zgpg30
TD 65356
SOLVENT CDCl3
NS 256
DS 4
SWH 24038.461 Hz
FIDRES 0.735616 Hz
AQ 1.3594048 sec
RG 45.21
DW 20.800 usec
DE 25.00 usec
TE 298.0 K
D1 2.00000000 sec
D11 0.03000000 sec
TD0 1
SFO1 100.6655806 MHz
NUC1 13C
P1 10.00 usec
PLW1 17.99699974 W
SFO2 400.3016012 MHz
NUC2 1H
CPDPRG[2 waltz16
PCPD2 80.00 usec
PLW2 4.64209986 W
PLW12 0.10445000 W
PLW13 0.05245300 W
F2 - Processing parameters
SI 32768
SF 100.6555166 MHz
WDW EM
SSB 0
LB 1.00 Hz
GB 0
PC 1.40
24.452
26.553
30.830
32.094
43.622
45.471
64.246
67.273
70.487
70.926
114.840
115.825
ppm
20
40
60
80
100
120
140
160
180
200

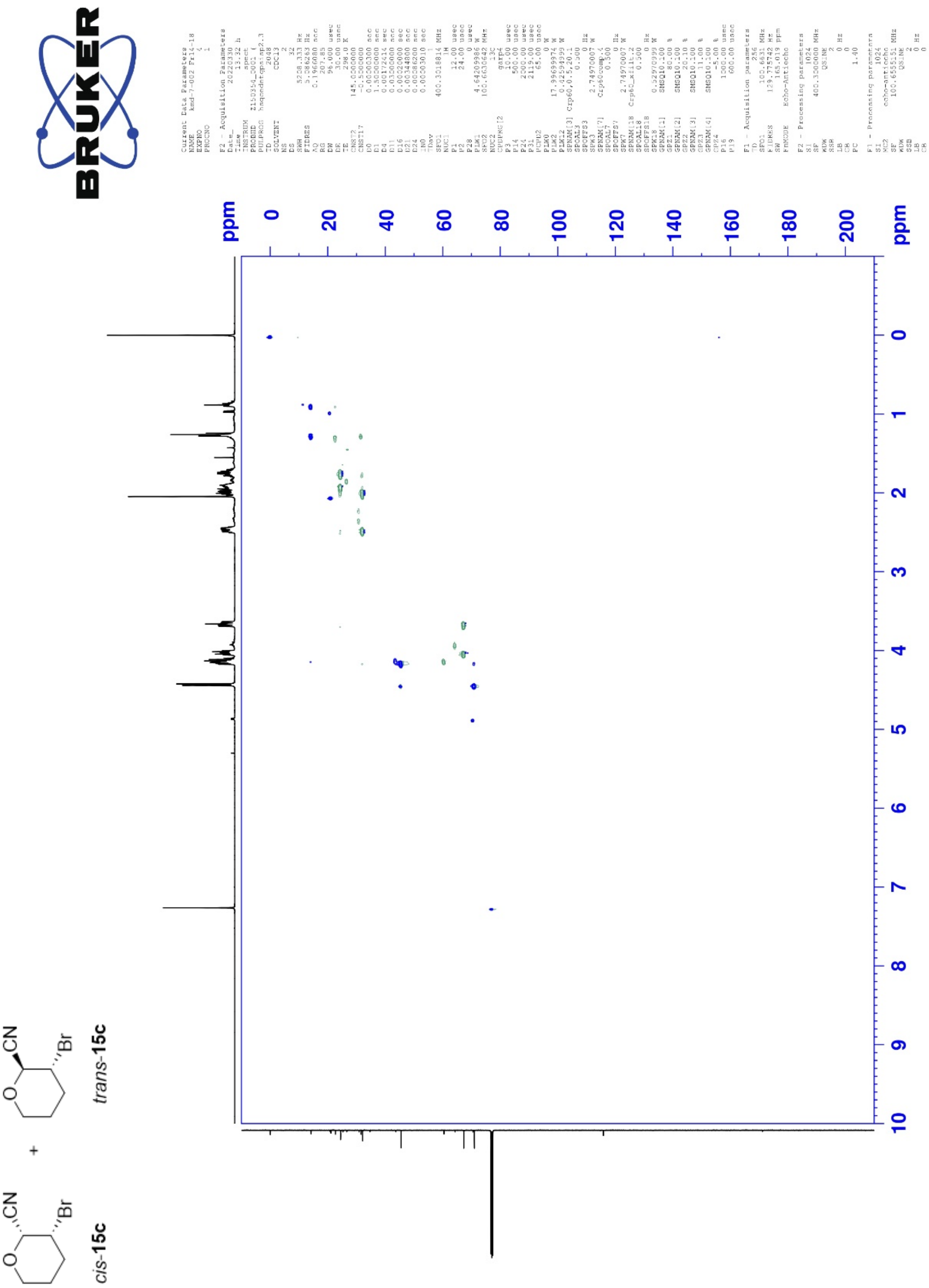

cis-**15c** + *trans*-**15c**

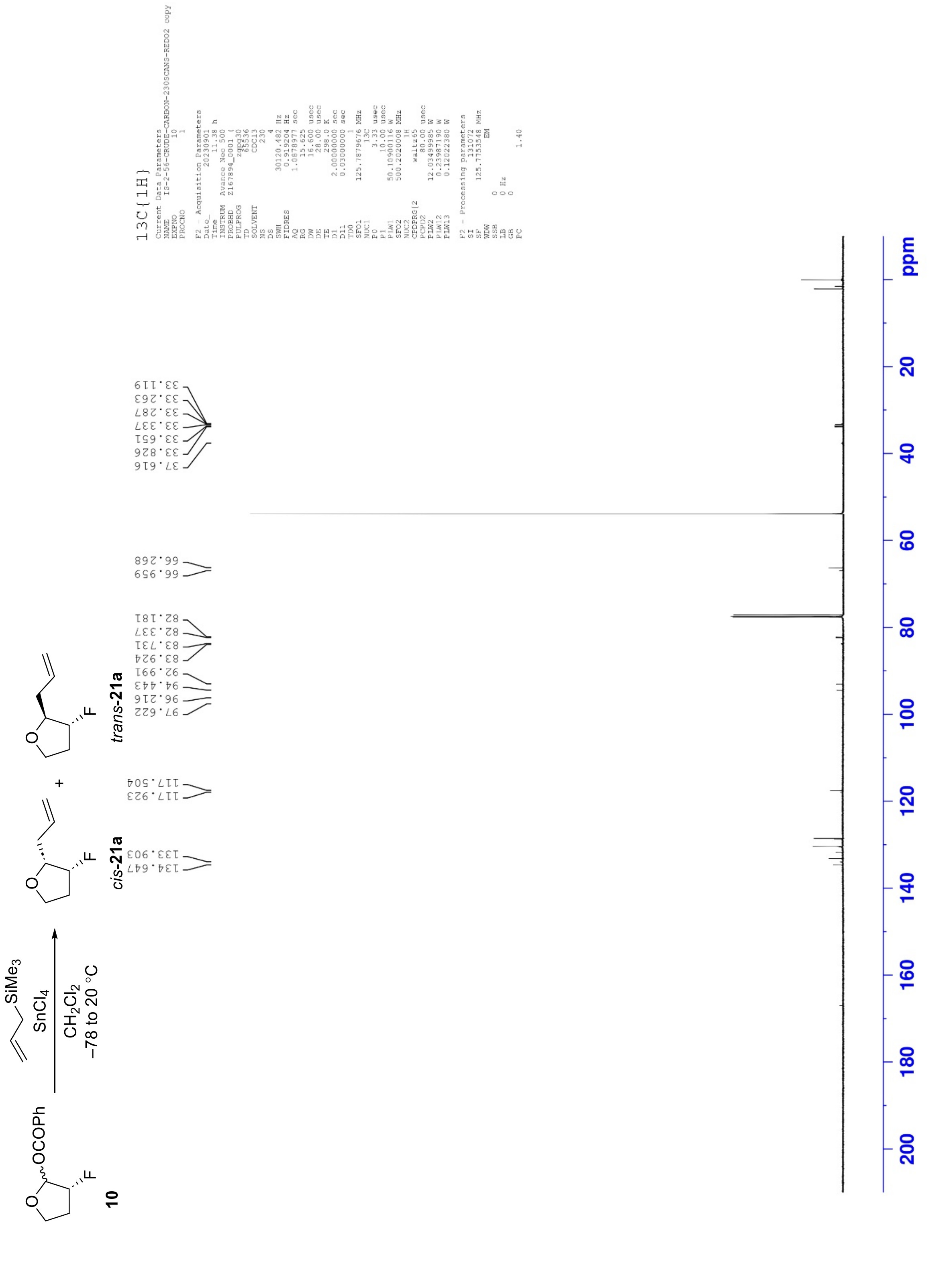
13C{1H}
33.119
33.263
33.287
33.337
33.651
33.826
37.616
66.268
66.959
82.181
82.337
83.731
83.924
92.991
94.443
96.216
97.622
117.504
117.923
133.903
134.647
SiMe3
SnCl4
CH2Cl2
−78 to 20 °C
OCOPh
F
10
cis-21a
trans-21a
ppm
20
40
60
80
100
120
140
160
180
200

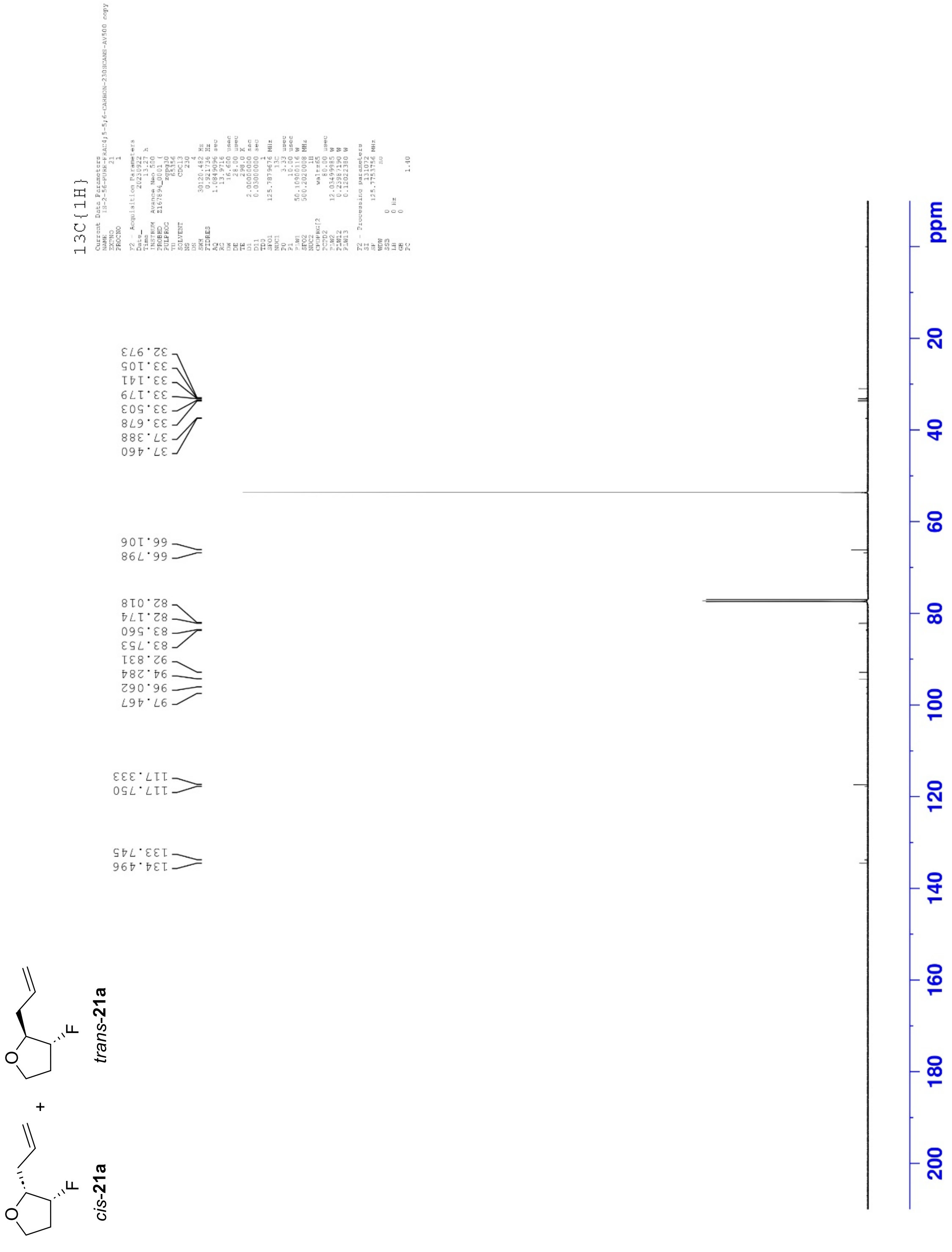
13C{1H}
32.973
33.105
33.141
33.179
33.503
33.678
37.388
37.460
66.106
66.798
82.018
82.174
83.560
83.753
92.831
94.284
96.062
97.467
117.333
117.750
133.745
134.496
cis-21a
trans-21a
ppm

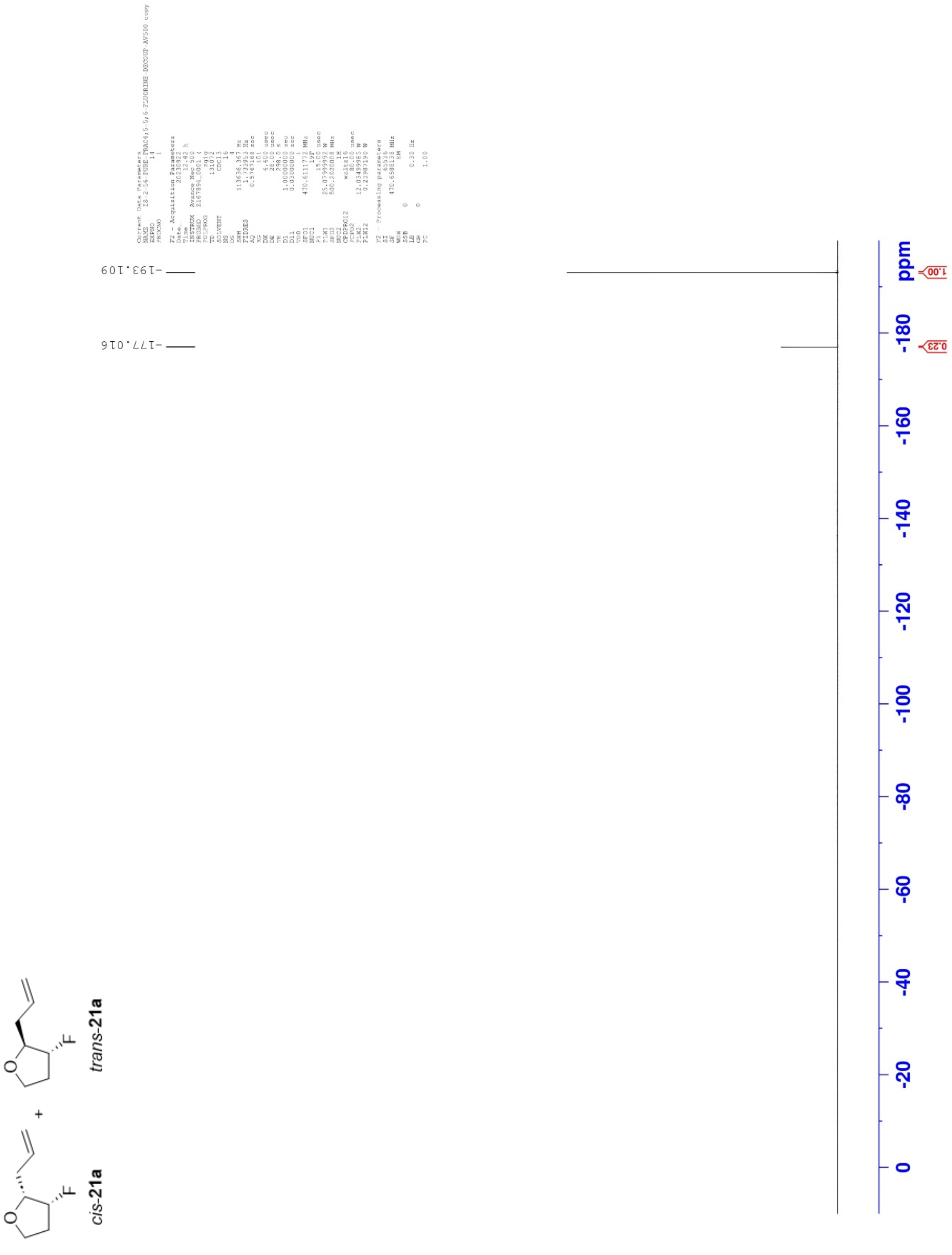
-193.109
-177.016
ppm
-180
-160
-140
-120
-100
-80
-60
-40
-20
0
1.00
0.23
cis-21a
trans-21a

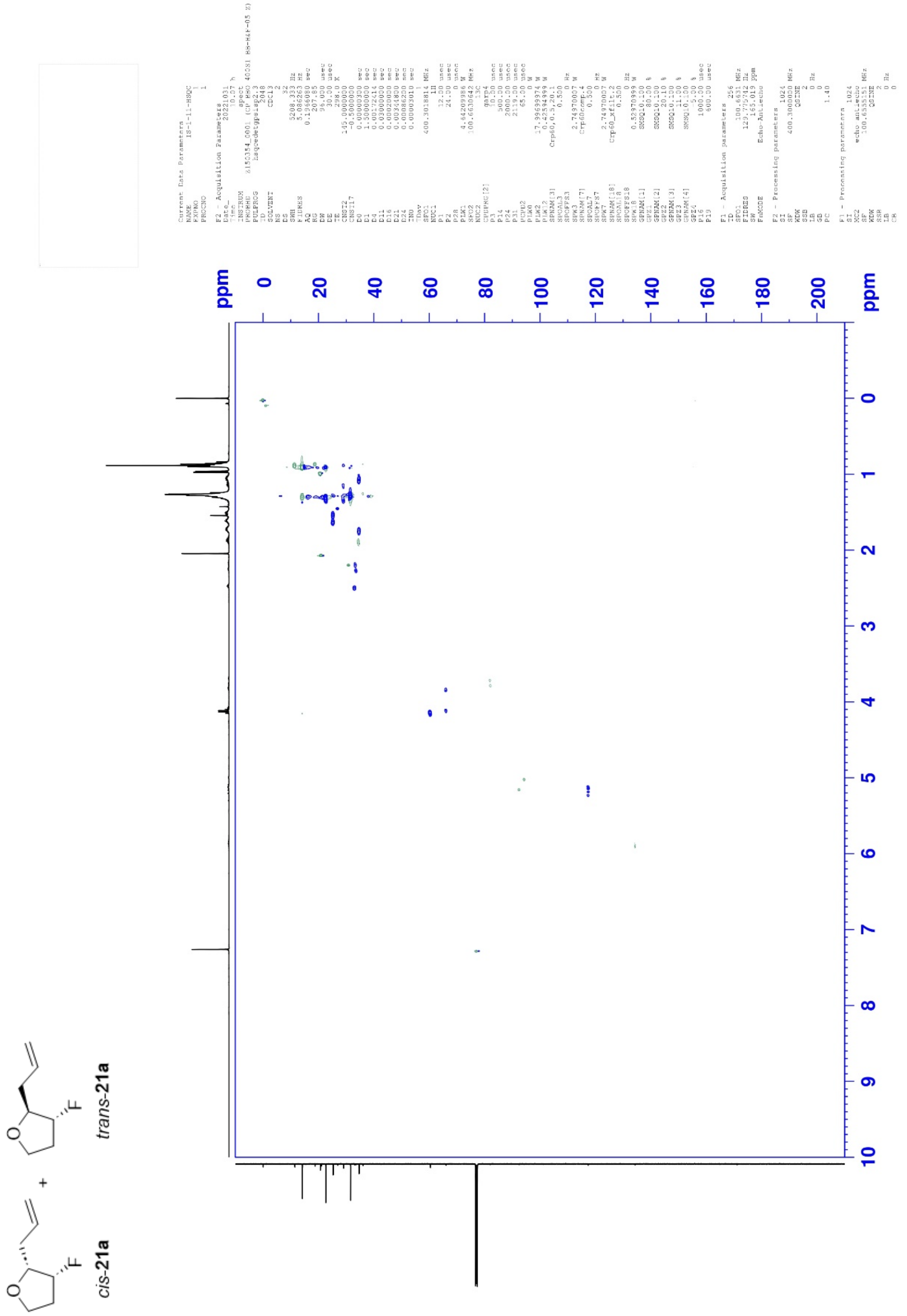
cis-21a
trans-21a

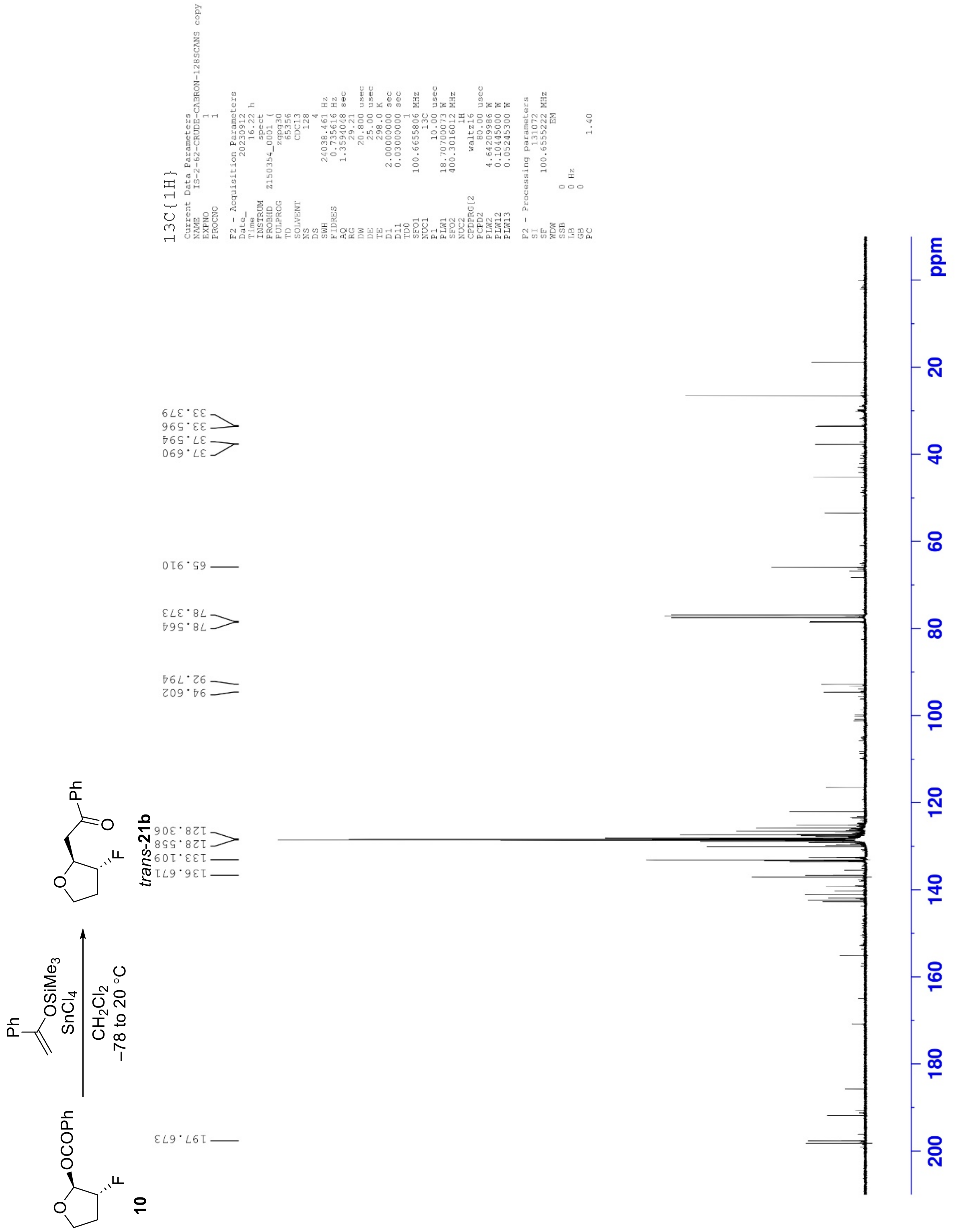
10
OCOPh
F
Ph
OSiMe3
SnCl4
CH2Cl2
−78 to 20 °C
Ph
O
F
trans-21b
13C{1H}
197.673
136.671
133.109
128.558
128.306
94.602
92.794
78.564
78.373
65.910
37.690
37.594
33.596
33.379
ppm

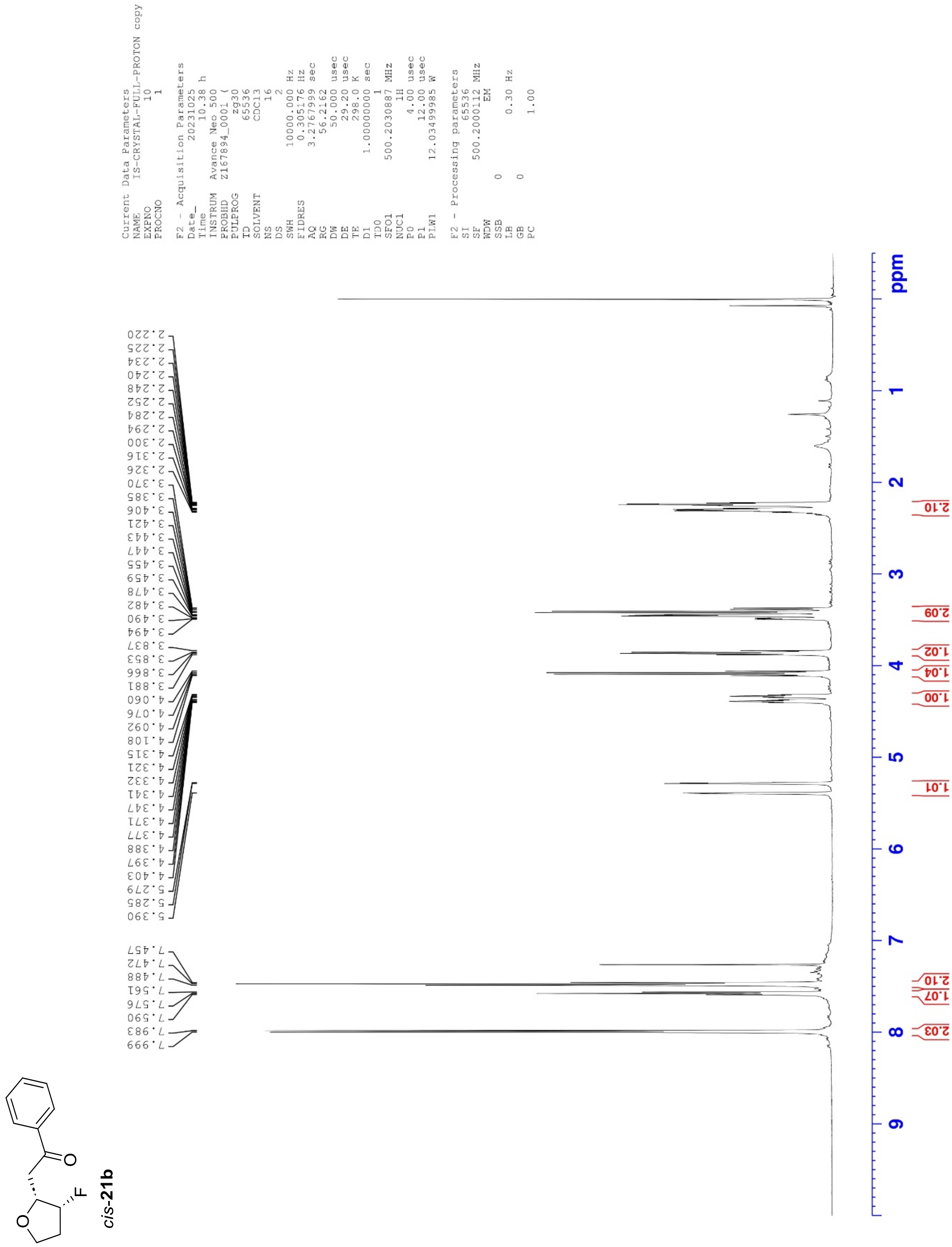
Current Data Parameters
NAME IS-CRYSTAL-FULL-PROTON copy
EXPNO 10
PROCNO 1
F2 - Acquisition Parameters
Date_ 20231025
Time 10.38 h
INSTRUM Avance Neo 500
PROBHD Z167894_0001 (
PULPROG zg30
TD 65536
SOLVENT CDCl3
NS 16
DS 2
SWH 10000.000 Hz
FIDRES 0.305176 Hz
AQ 3.2767999 sec
RG 56.2162
DW 50.000 usec
DE 29.20 usec
TE 298.0 K
D1 1.00000000 sec
TD0 1
SFO1 500.2030887 MHz
NUC1 1H
P0 4.00 usec
P1 12.00 usec
PLW1 12.03499985 W
F2 - Processing parameters
SI 65536
SF 500.2000112 MHz
WDW EM
SSB 0
LB 0.30 Hz
GB 0
PC 1.00
ppm

cis-**21b**

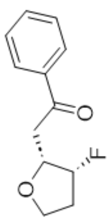

cis-**21b**

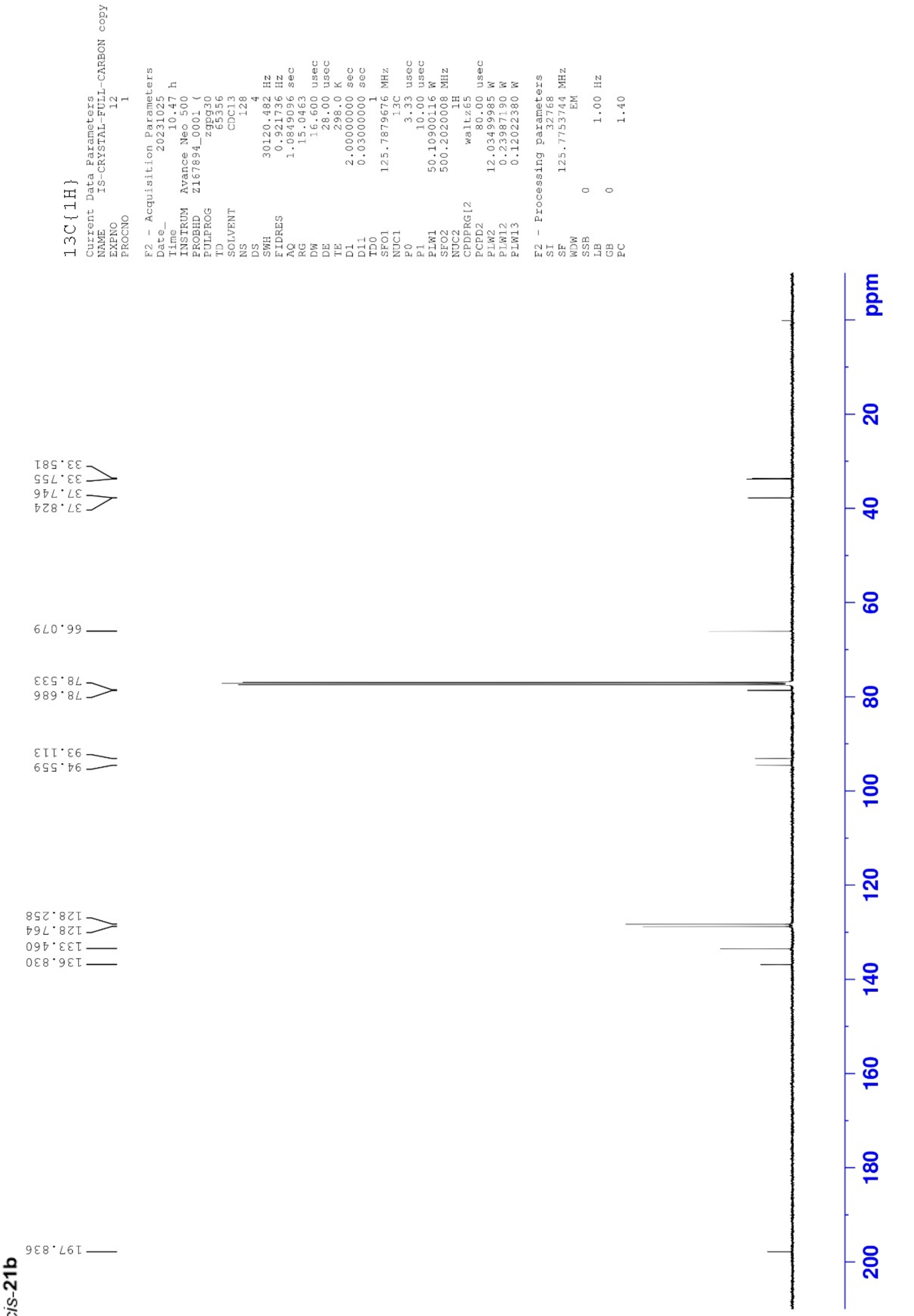
197.836
136.830
133.460
128.764
128.258
94.559
93.113
78.686
78.533
66.079
37.824
37.746
33.755
33.581
13C{1H}
Current Data Parameters
NAME IS-CRYSTAL-FULL-CARBON copy
EXPNO 12
PROCNO 1
F2 - Acquisition Parameters
Date_ 20231025
Time 10.47 h
INSTRUM Avance Neo 500
PROBHD Z167894_0001 (
PULPROG zgpg30
TD 65356
SOLVENT CDCl3
NS 128
DS 4
SWH 30120.482 Hz
FIDRES 0.921736 Hz
AQ 1.0849096 sec
RG 15.0463
DW 16.600 usec
DE 28.00 usec
TE 298.0 K
D1 2.00000000 sec
D11 0.03000000 sec
TD0 1
SFO1 125.7879676 MHz
NUC1 13C
P0 3.33 usec
P1 10.00 usec
PLW1 50.10900116 W
SFO2 500.2020008 MHz
NUC2 1H
CPDPRG[2 waltz65
PCPD2 80.00 usec
PLW2 12.03499985 W
PLW12 0.23987190 W
PLW13 0.12022380 W
F2 - Processing parameters
SI 32768
SF 125.7753744 MHz
WDW EM
SSB 0
LB 1.00 Hz
GB 0
PC 1.40
200
180
160
140
120
100
80
60
40
20
ppm

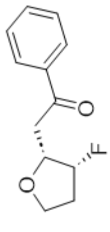

cis-**21b**

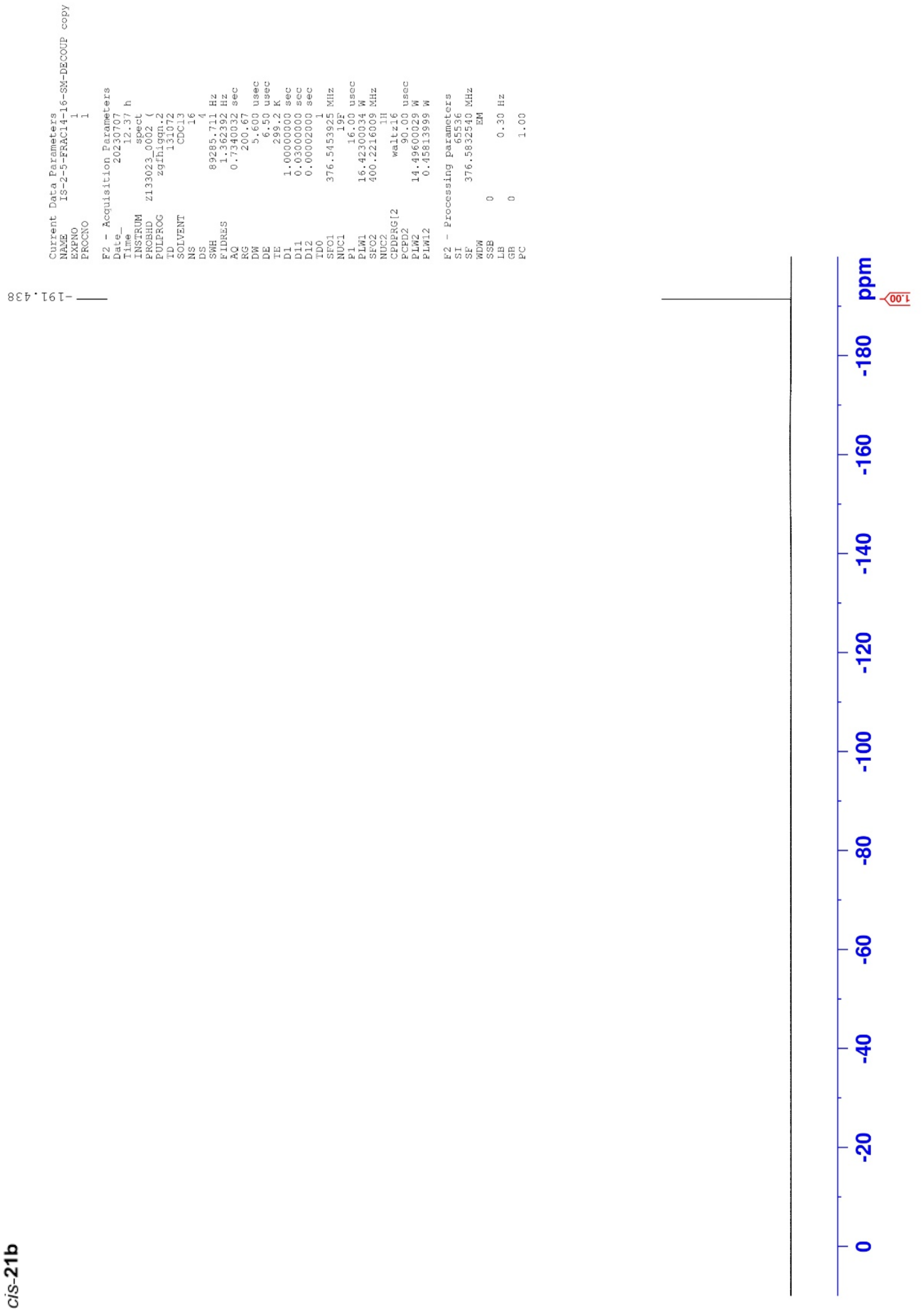
Current Data Parameters
NAME IS-2-5-FRAC14-16-SM-DECOUP copy
EXPNO 1
PROCNO 1
F2 - Acquisition Parameters
Date_ 20230707
Time 12.37 h
INSTRUM spect
PROBHD Z133023_0002 (
PULPROG zgfhigqn.2
TD 131072
SOLVENT CDCl3
NS 16
DS 4
SWH 89285.711 Hz
FIDRES 1.362392 Hz
AQ 0.7340032 sec
RG 200.67
DW 5.600 usec
DE 6.50 usec
TE 298.2 K
D1 1.00000000 sec
D11 0.03000000 sec
D12 0.00002000 sec
TD0 1
SFO1 376.5453925 MHz
NUC1 19F
P1 16.00 usec
PLW1 16.42300034 W
SFO2 400.2216009 MHz
NUC2 1H
CPDPRG[2 waltz16
PCPD2 90.00 usec
PLW2 14.49600029 W
PLW12 0.45813999 W
F2 - Processing parameters
SI 65536
SF 376.5832540 MHz
WDW EM
SSB 0
LB 0.30 Hz
GB 0
PC 1.00
-191.438
1.00
ppm
-180
-160
-140
-120
-100
-80
-60
-40
-20
0

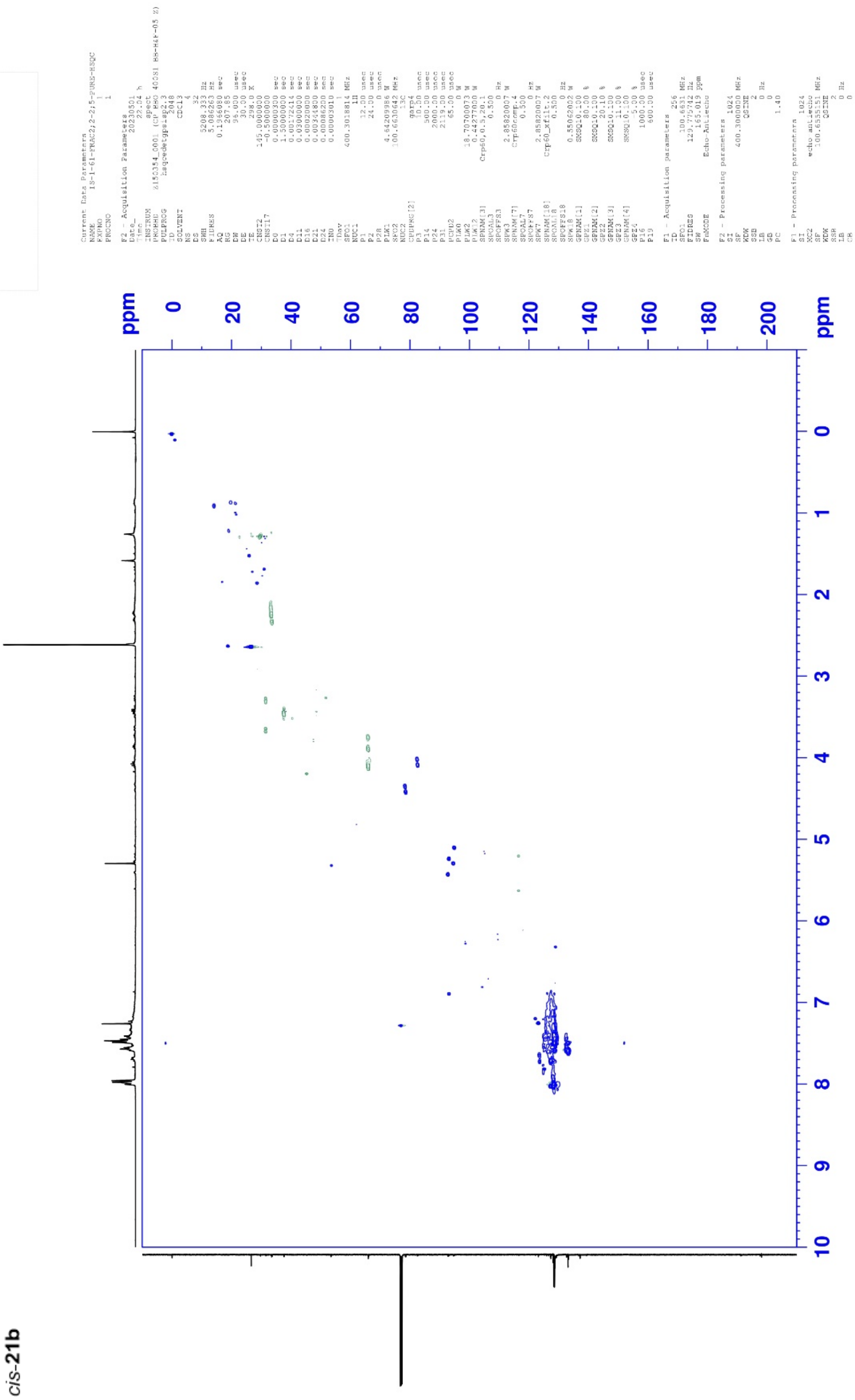

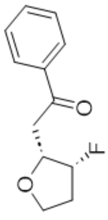

cis-**21b**

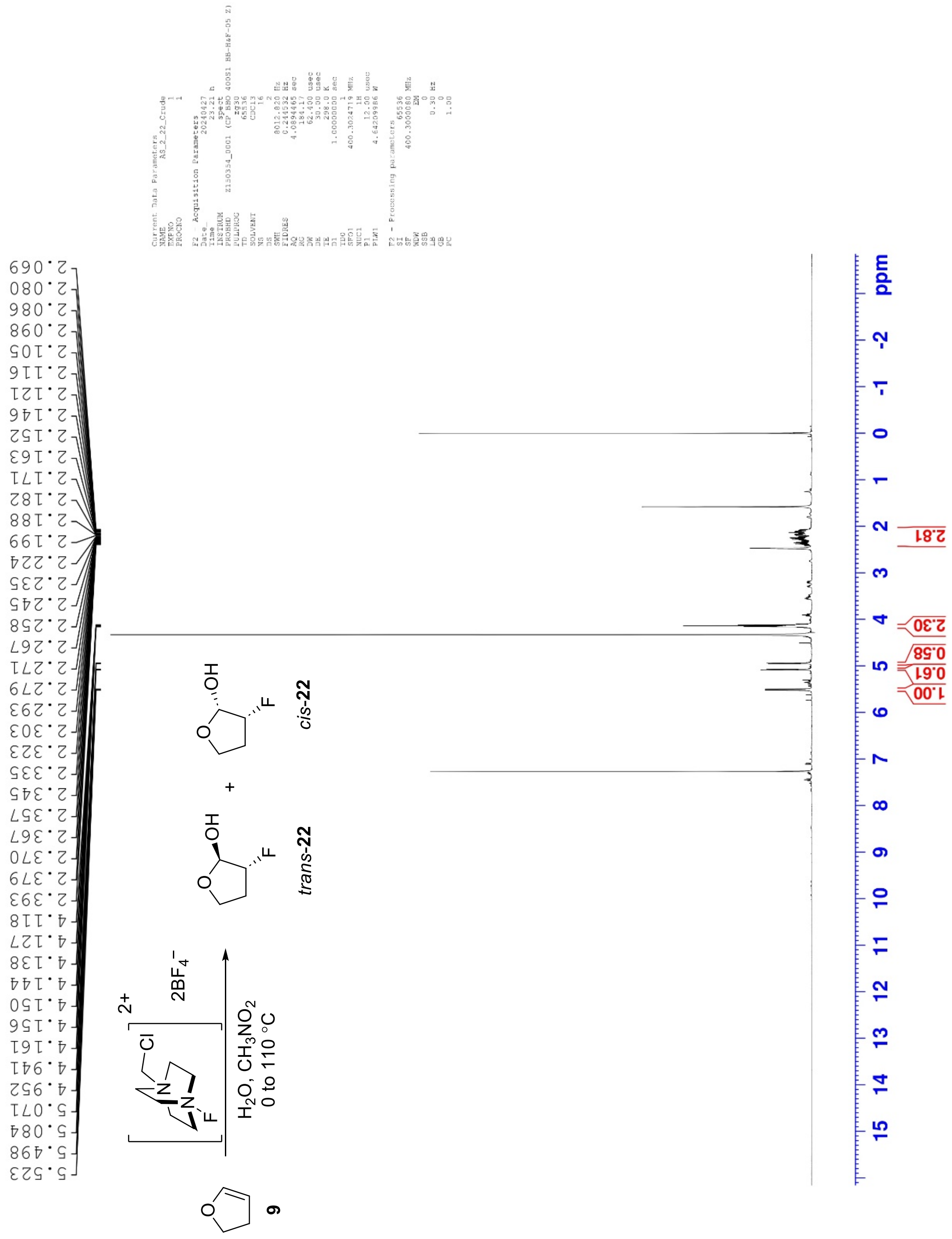
2.069
2.080
2.086
2.098
2.105
2.116
2.121
2.146
2.152
2.163
2.171
2.182
2.188
2.199
2.224
2.235
2.245
2.258
2.267
2.271
2.279
2.293
2.303
2.323
2.335
2.345
2.357
2.367
2.370
2.379
2.393
4.118
4.127
4.138
4.144
4.150
4.156
4.161
4.941
4.952
5.071
5.084
5.498
5.523
9
2BF4−
H2O, CH3NO2
0 to 110 °C
trans-22
cis-22
2.81
2.30
0.58
0.61
1.00
ppm

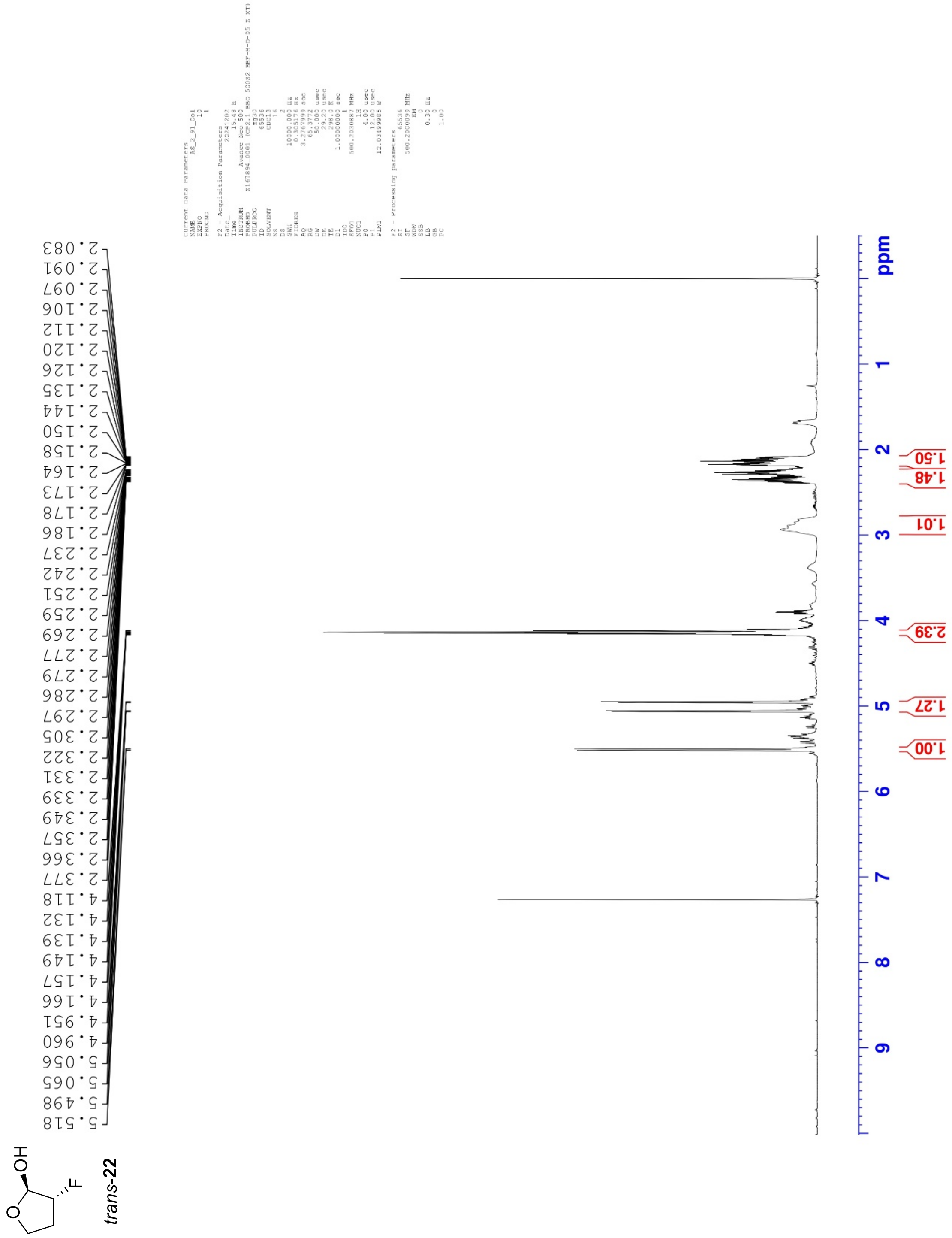
2.083
2.091
2.097
2.106
2.112
2.120
2.126
2.135
2.144
2.150
2.158
2.164
2.173
2.178
2.186
2.237
2.242
2.251
2.259
2.269
2.277
2.279
2.286
2.297
2.305
2.322
2.331
2.339
2.349
2.357
2.366
2.377
4.118
4.132
4.139
4.149
4.157
4.166
4.951
4.960
5.056
5.065
5.498
5.518
1.50
1.48
1.01
2.39
1.27
1.00
ppm
1
2
3
4
5
6
7
8
9
trans-22

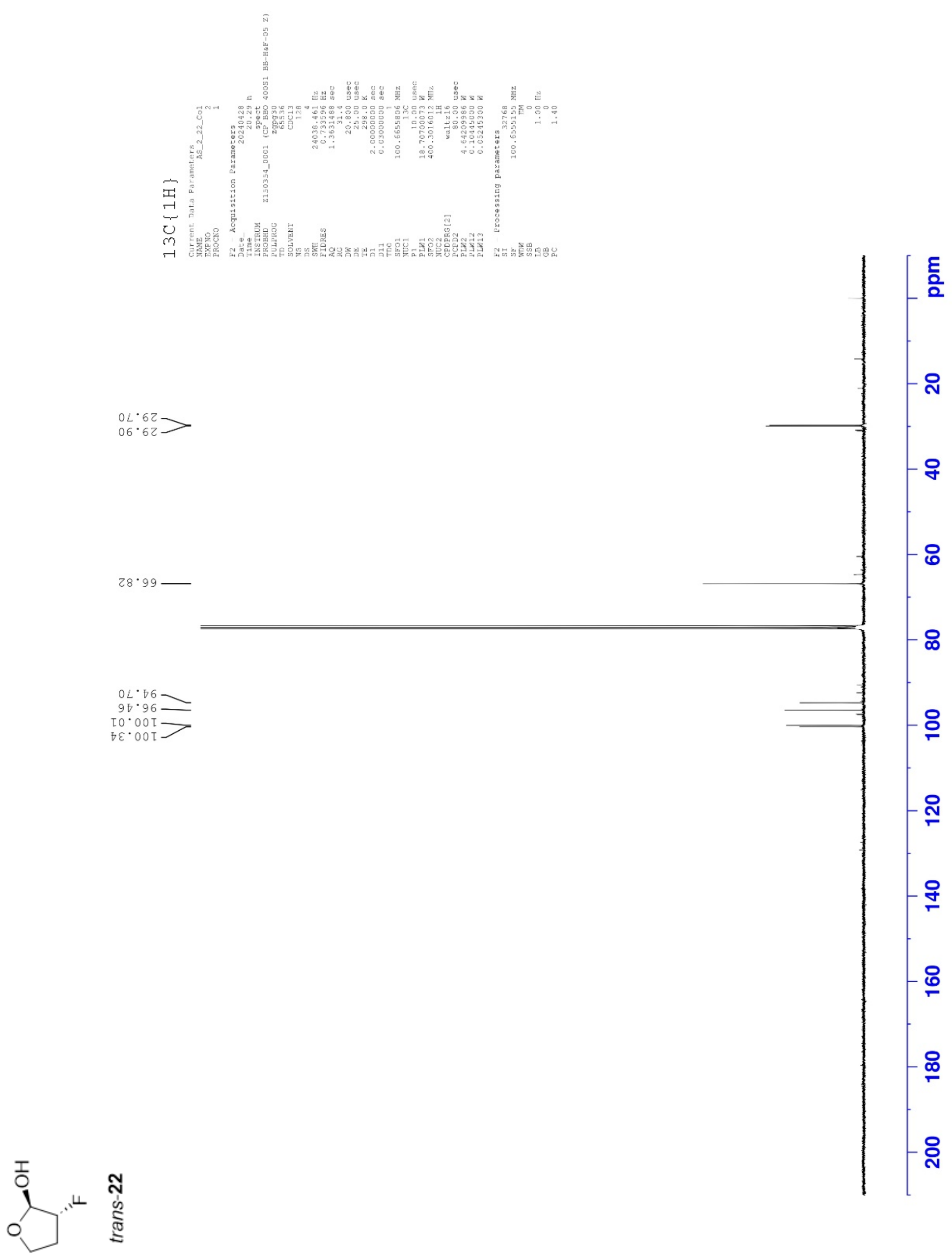
13C{1H}
29.70
29.90
66.82
94.70
96.46
100.01
100.34
ppm
20
40
60
80
100
120
140
160
180
200
trans-22

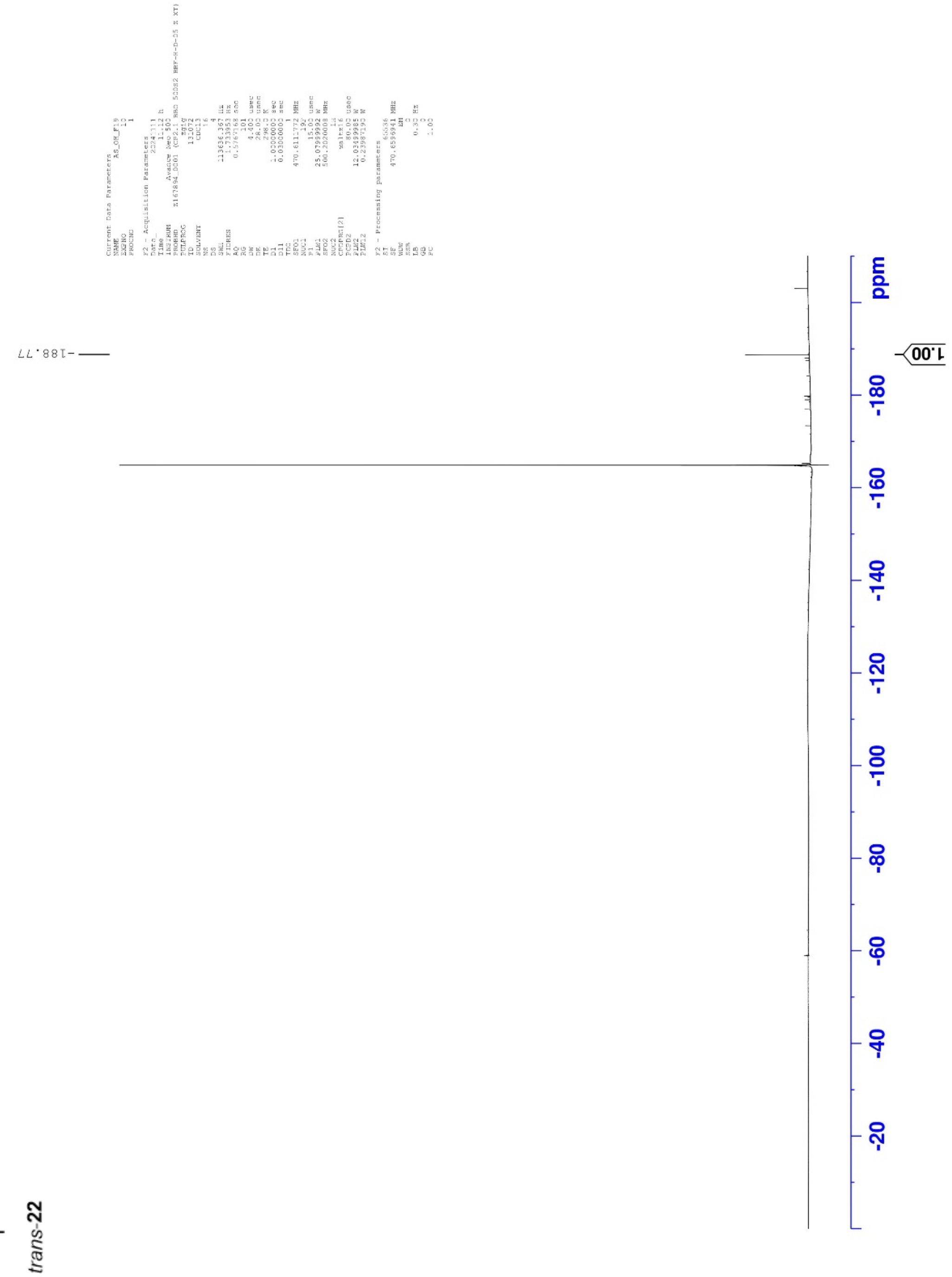

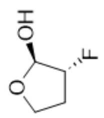

trans-**22**

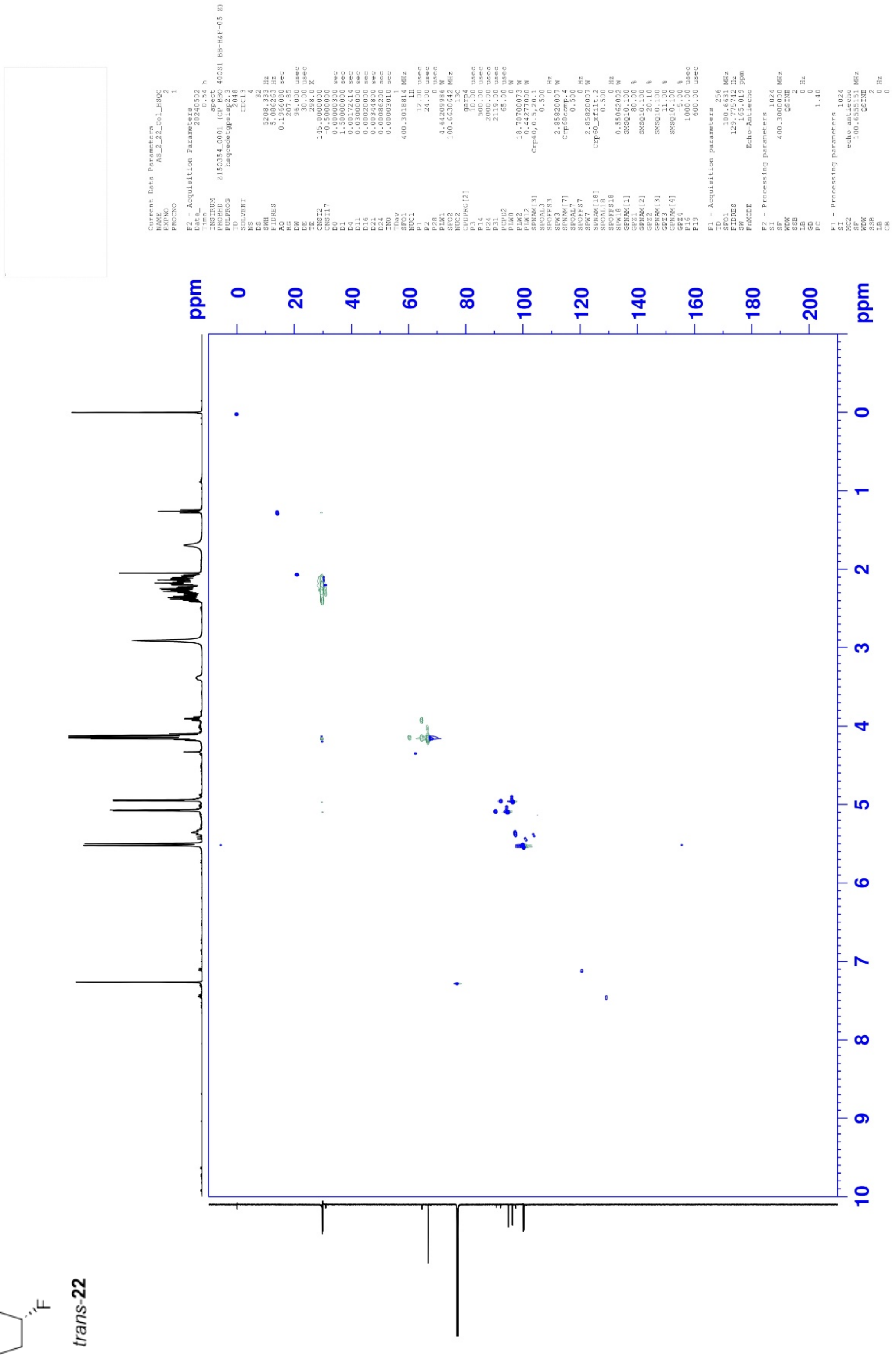

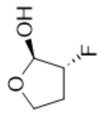

trans-**22**

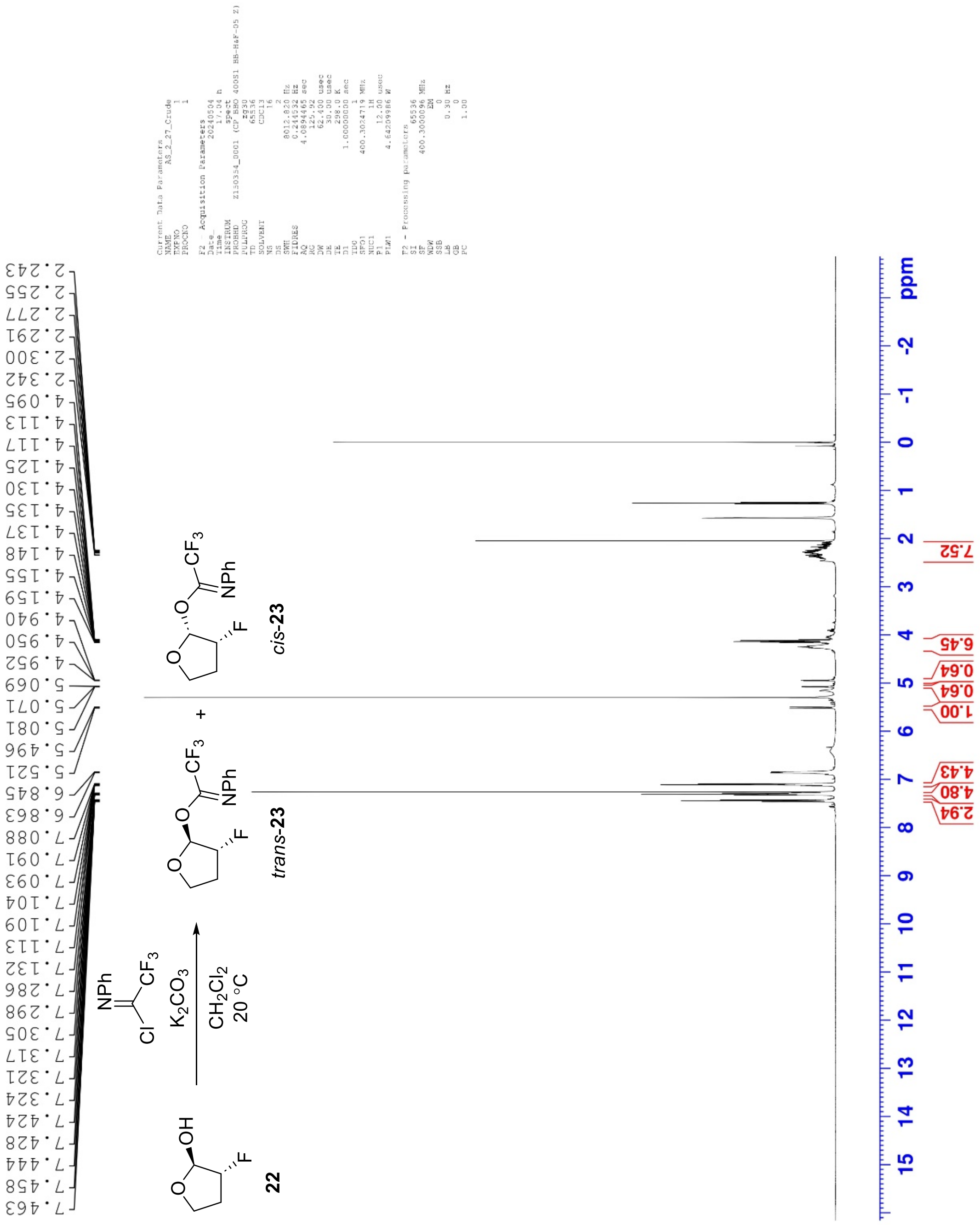
22
trans-23
cis-23
K2CO3
CH2Cl2
20 °C
ppm

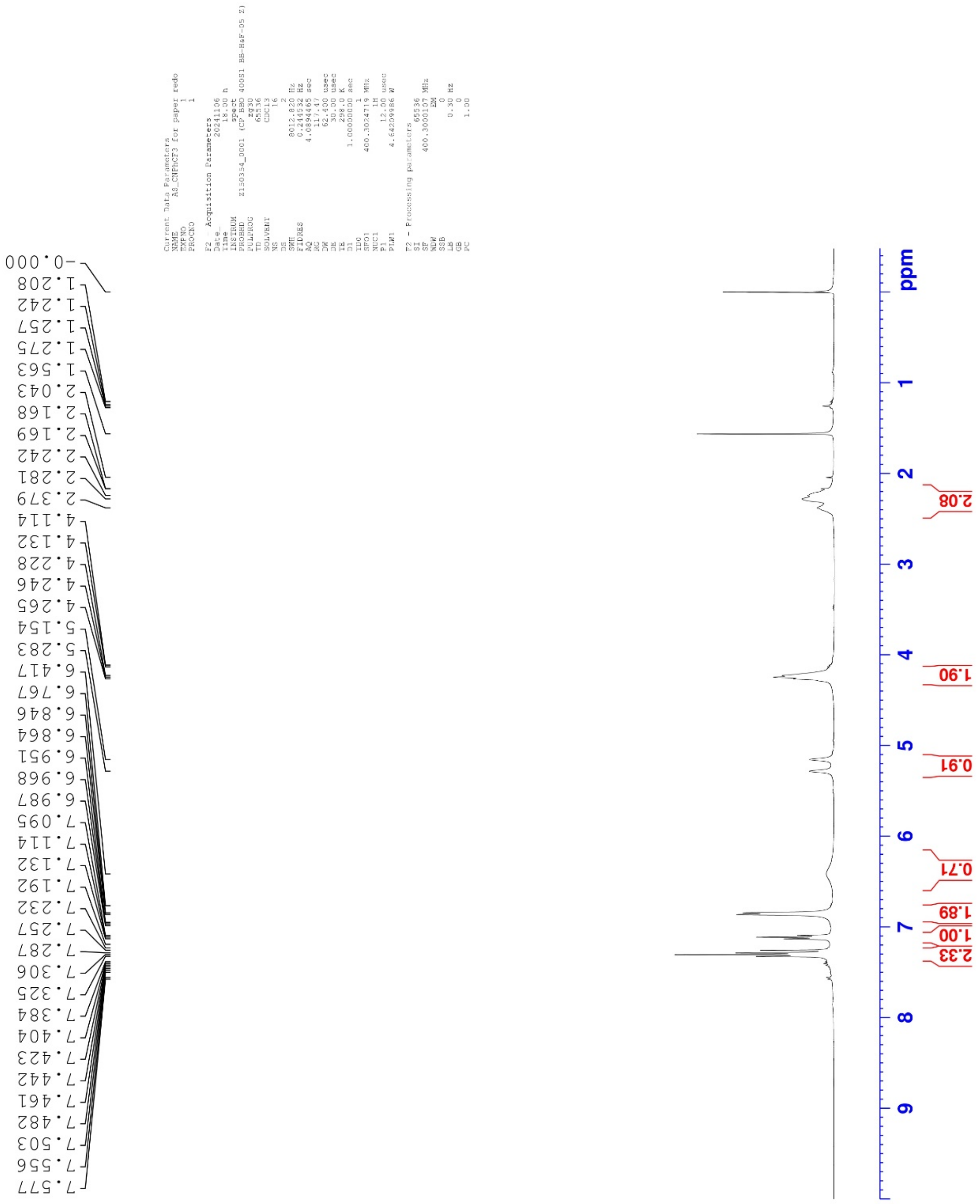

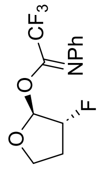

trans-**23**

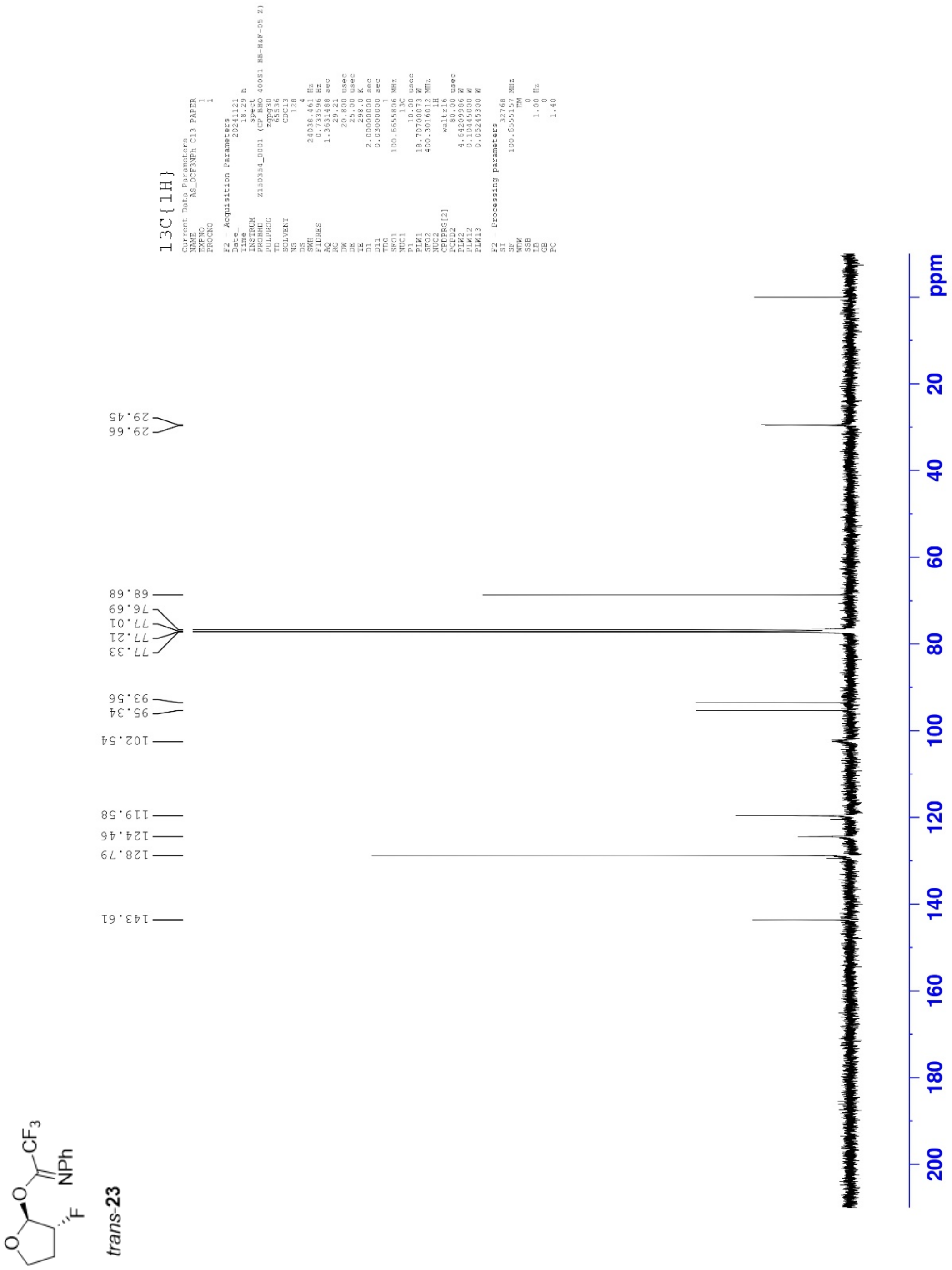
13C{1H}
29.45
29.66
68.68
76.69
77.01
77.21
77.33
93.56
95.34
102.54
119.58
124.46
128.79
143.61
ppm
CF3
NPh
F

trans-23

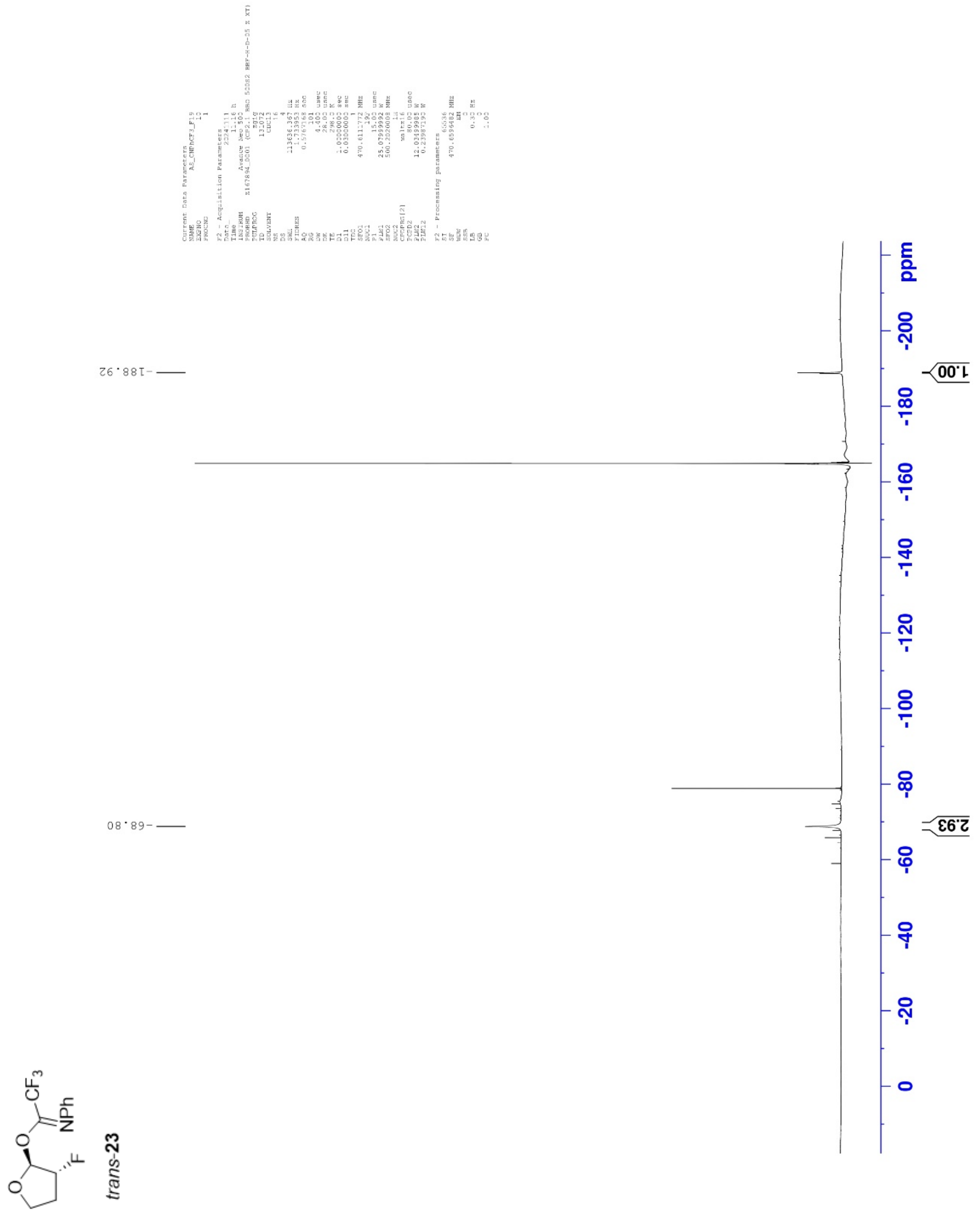
-188.92
-68.80
1.00
2.93
ppm
-200
-180
-160
-140
-120
-100
-80
-60
-40
-20
0
CF3
NPh
O
F

trans-23

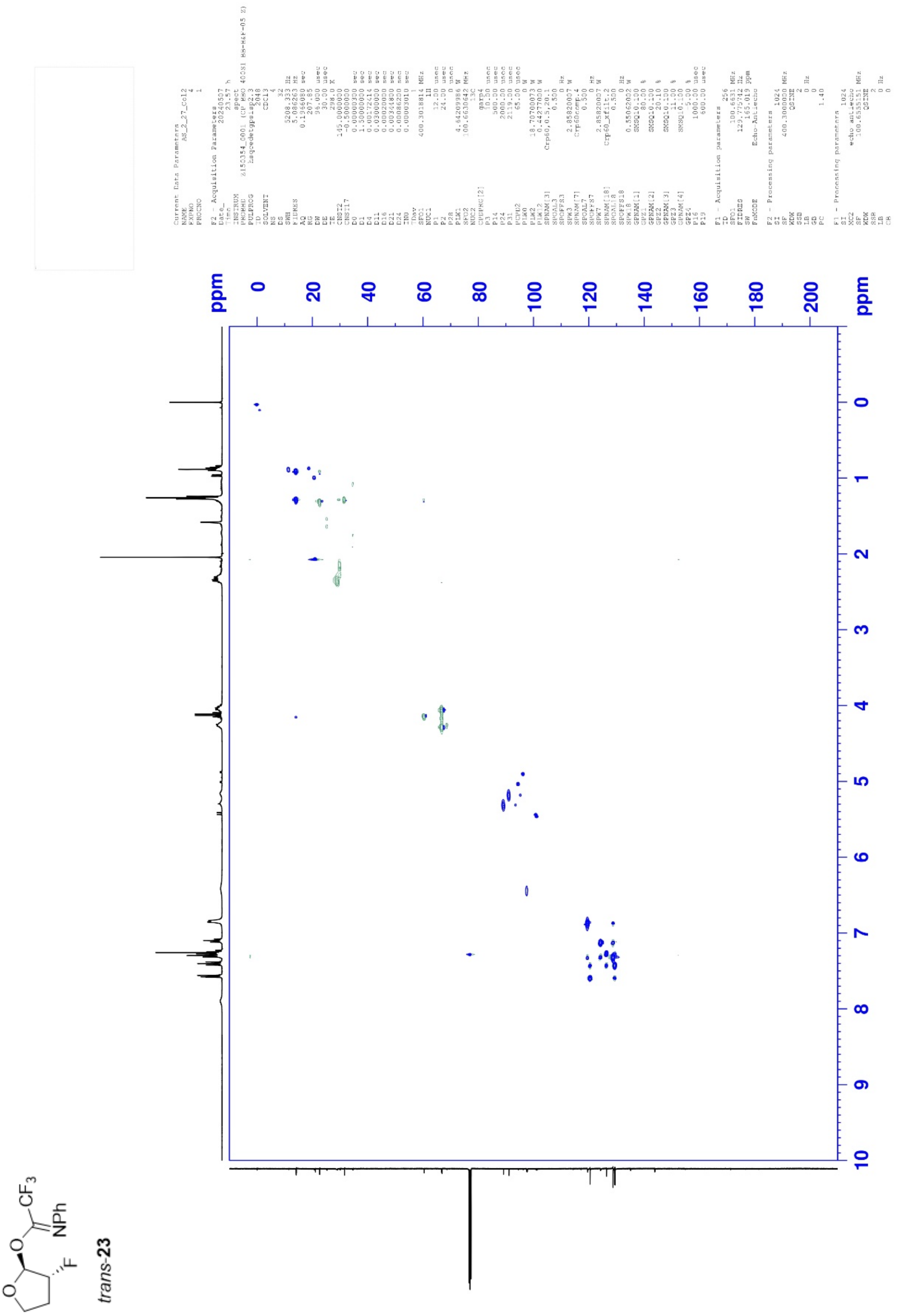
trans-23
ppm

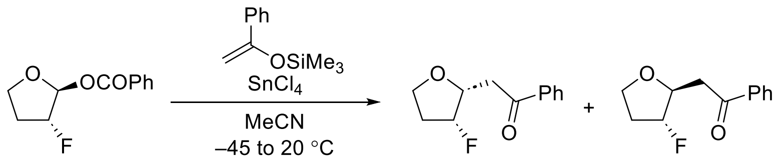

10 *cis*-**21b** *trans*-**21b**

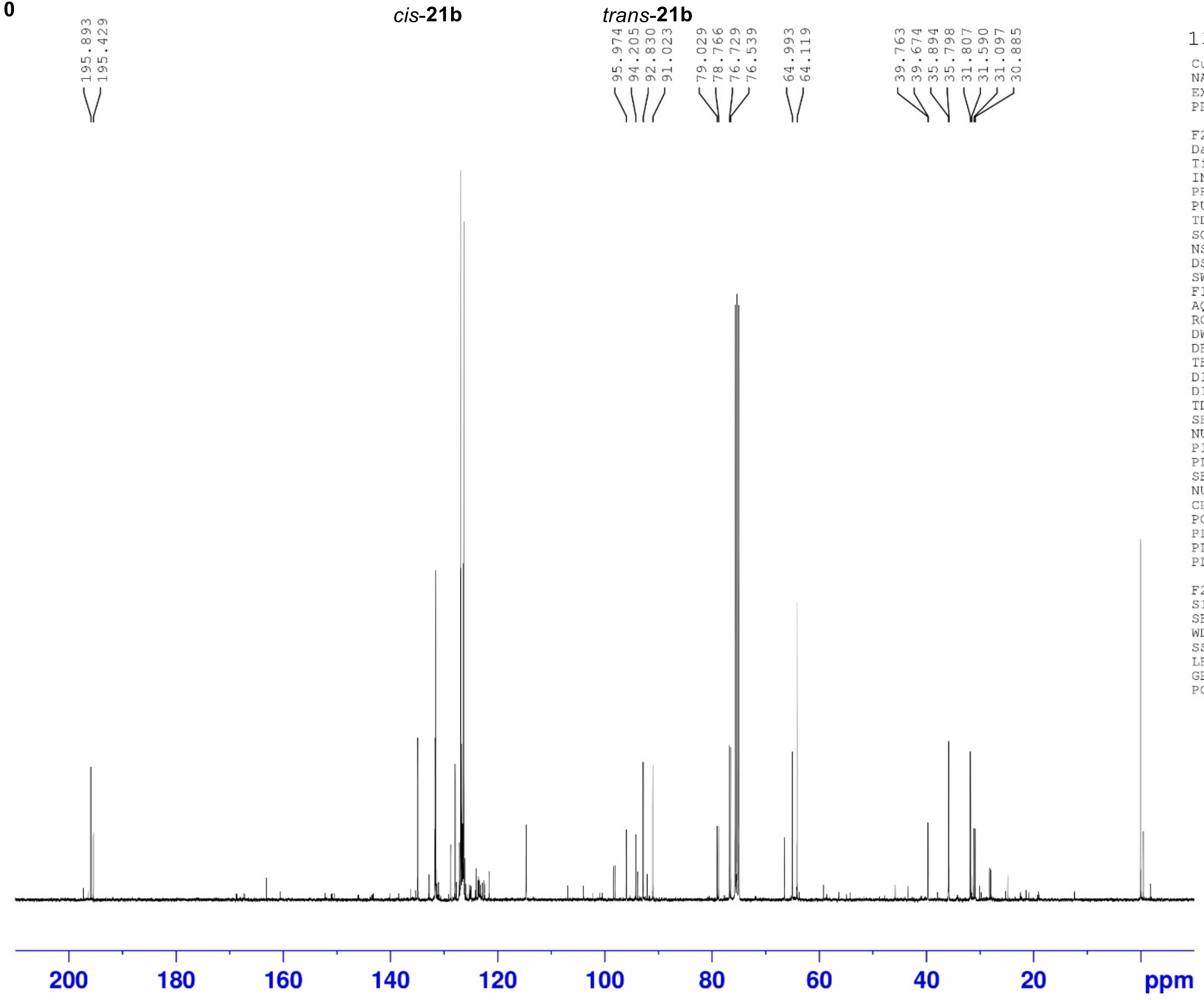

13C{1H}

```
Current Data Parameters
NAME      IS-2-21-CRUDE-CARBON copy
EXPNO                  1
PROCNO                 1

F2 - Acquisition Parameters
Date_           20230526
Time               17.36 h
INSTRUM            spect
PROBHD    Z150354_0001 (
PULPROG          zgpg30
TD                 65356
SOLVENT            CDCl3
NS                   128
DS                     4
SWH            24038.461 Hz
FIDRES          0.735616 Hz
AQ             1.3594048 sec
RG                  31.4
DW                20.800 usec
DE                 25.00 usec
TE                 298.0 K
D1            2.00000000 sec
D11           0.03000000 sec
TD0                    1
SFO1         100.6655806 MHz
NUC1                 13C
P1                 10.00 usec
PLW1         18.70700073 W
SFO2         400.3016012 MHz
NUC2                  1H
CPDPRG[2         waltz16
PCPD2              80.00 usec
PLW2          4.64209986 W
PLW12         0.10445000 W
PLW13         0.05245300 W

F2 - Processing parameters
SI                131072
SF           100.6556975 MHz
WDW                   EM
SSB       0
LB                  1.00 Hz
GB        0
PC                  1.40
```

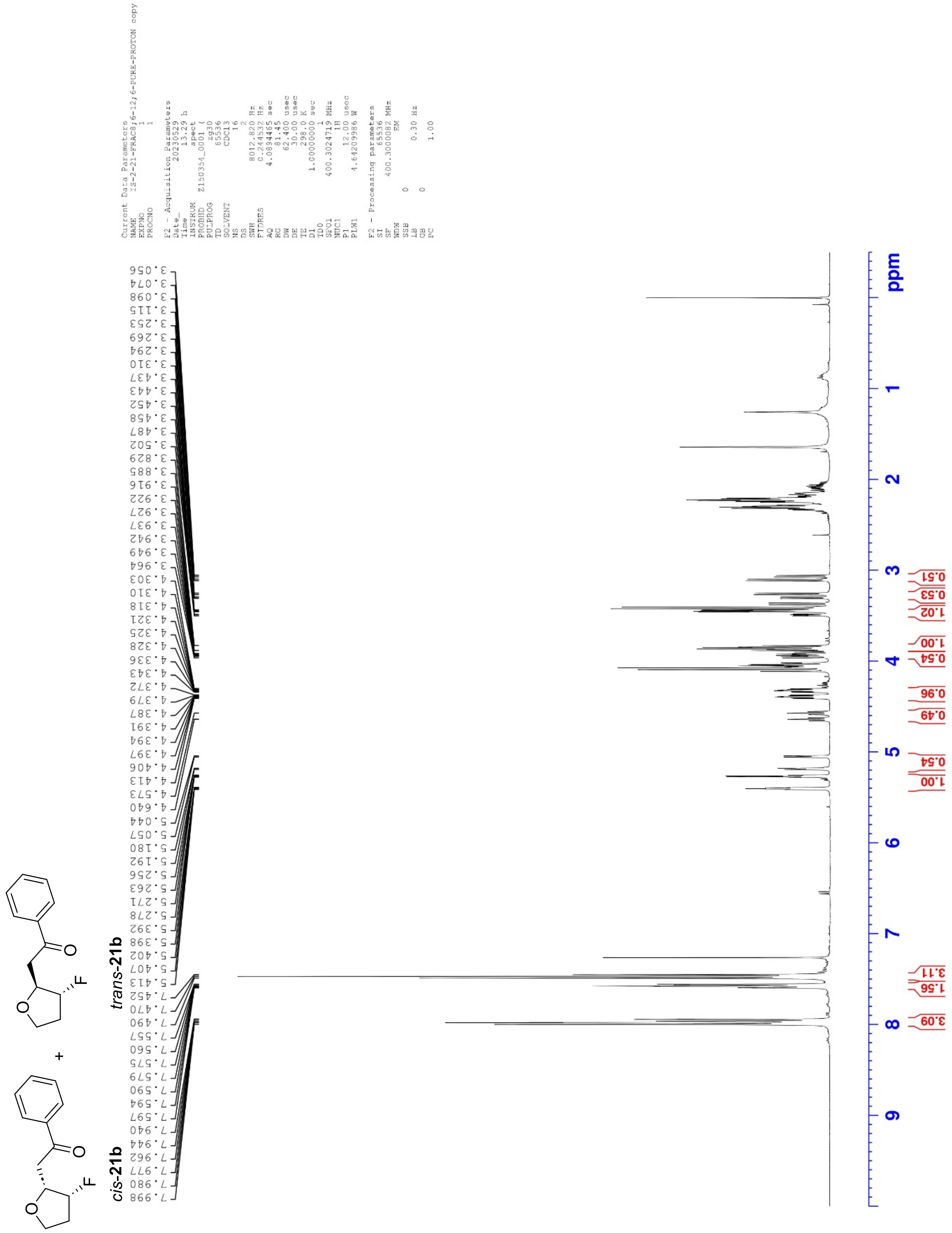
cis-21b
trans-21b

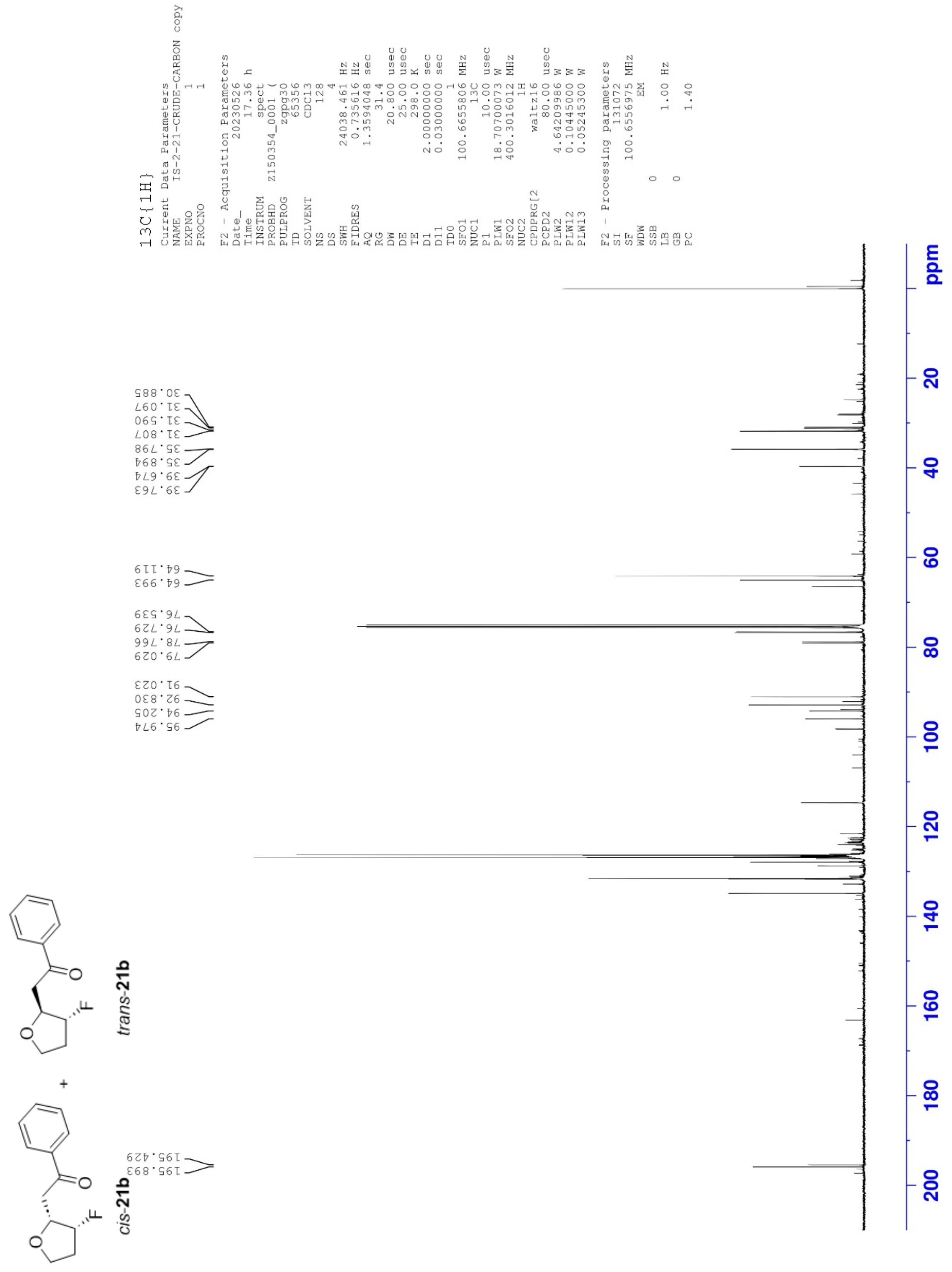
cis-21b
trans-21b
195.893
195.429
95.974
94.205
92.830
91.023
79.029
78.766
76.729
76.539
64.993
64.119
39.763
39.674
35.894
35.798
31.807
31.590
31.097
30.885
13C{1H}
Current Data Parameters
NAME IS-2-21-CRUDE-CARBON copy
EXPNO 1
PROCNO 1
F2 - Acquisition Parameters
Date_ 20230526
Time 17.36 h
INSTRUM spect
PROBHD Z150354_0001 (
PULPROG zgpg30
TD 65356
SOLVENT CDCl3
NS 128
DS 4
SWH 24038.461 Hz
FIDRES 0.735616 Hz
AQ 1.3594048 sec
RG 31.4
DW 20.800 usec
DE 25.00 usec
TE 298.0 K
D1 2.00000000 sec
D11 0.03000000 sec
TD0 1
SFO1 100.6655806 MHz
NUC1 13C
P1 10.00 usec
PLW1 18.70700073 W
SFO2 400.3016012 MHz
NUC2 1H
CPDPRG[2 waltz16
PCPD2 80.00 usec
PLW2 4.64209986 W
PLW12 0.10445000 W
PLW13 0.05245300 W
F2 - Processing parameters
SI 131072
SF 100.6556975 MHz
WDW EM
SSB 0
LB 1.00 Hz
GB 0
PC 1.40
200
180
160
140
120
100
80
60
40
20
ppm

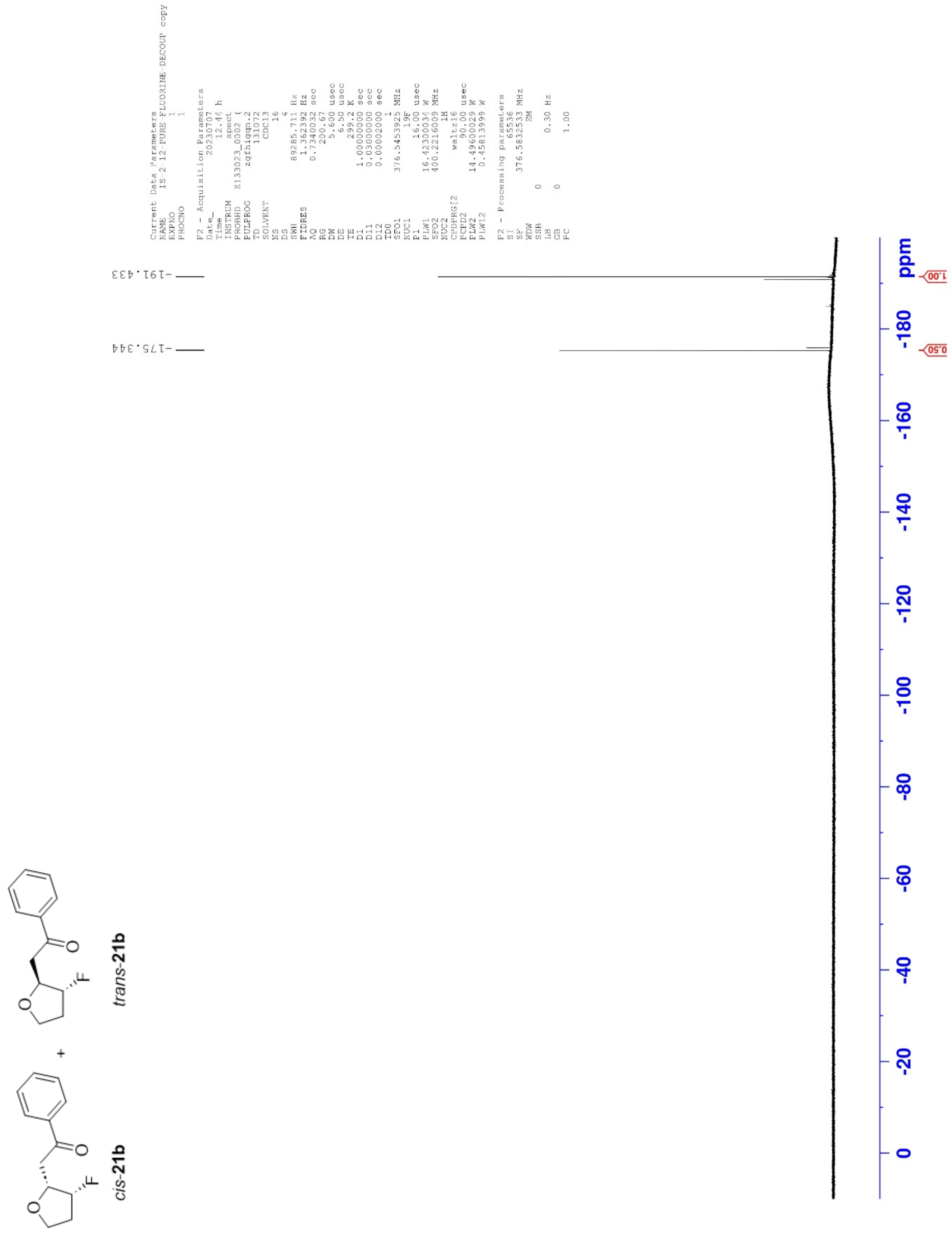
cis-21b
trans-21b
-191.433
-175.344
ppm
-180
-160
-140
-120
-100
-80
-60
-40
-20
0
1.00
0.50

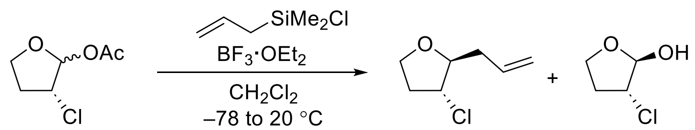

11 → *trans*-**24a** + *trans*-**26**

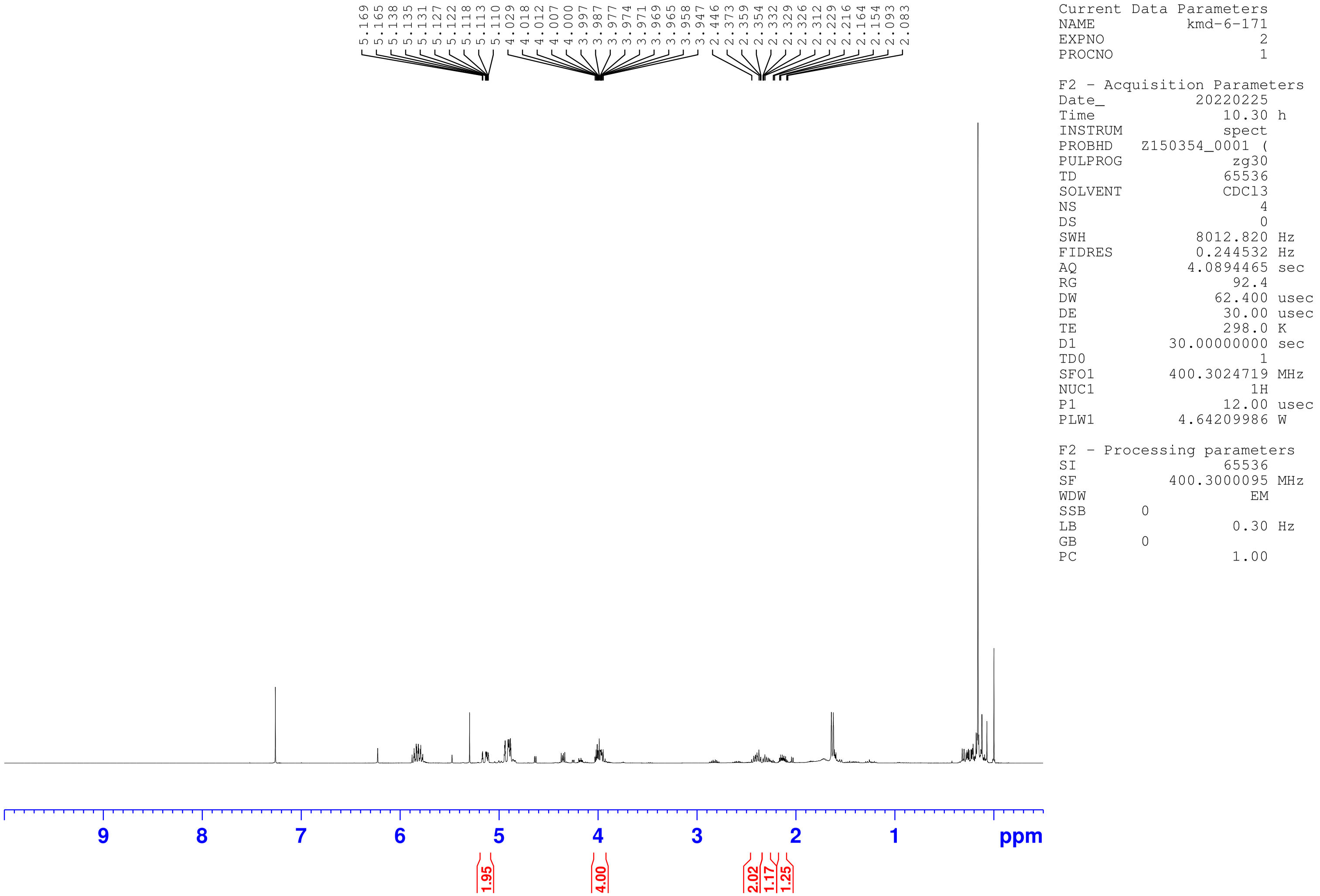

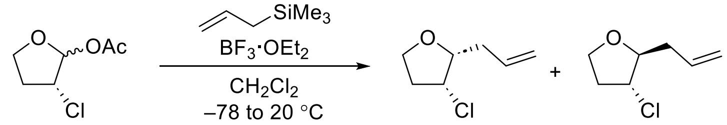

11 → *cis*-**24a** + *trans*-**24a**

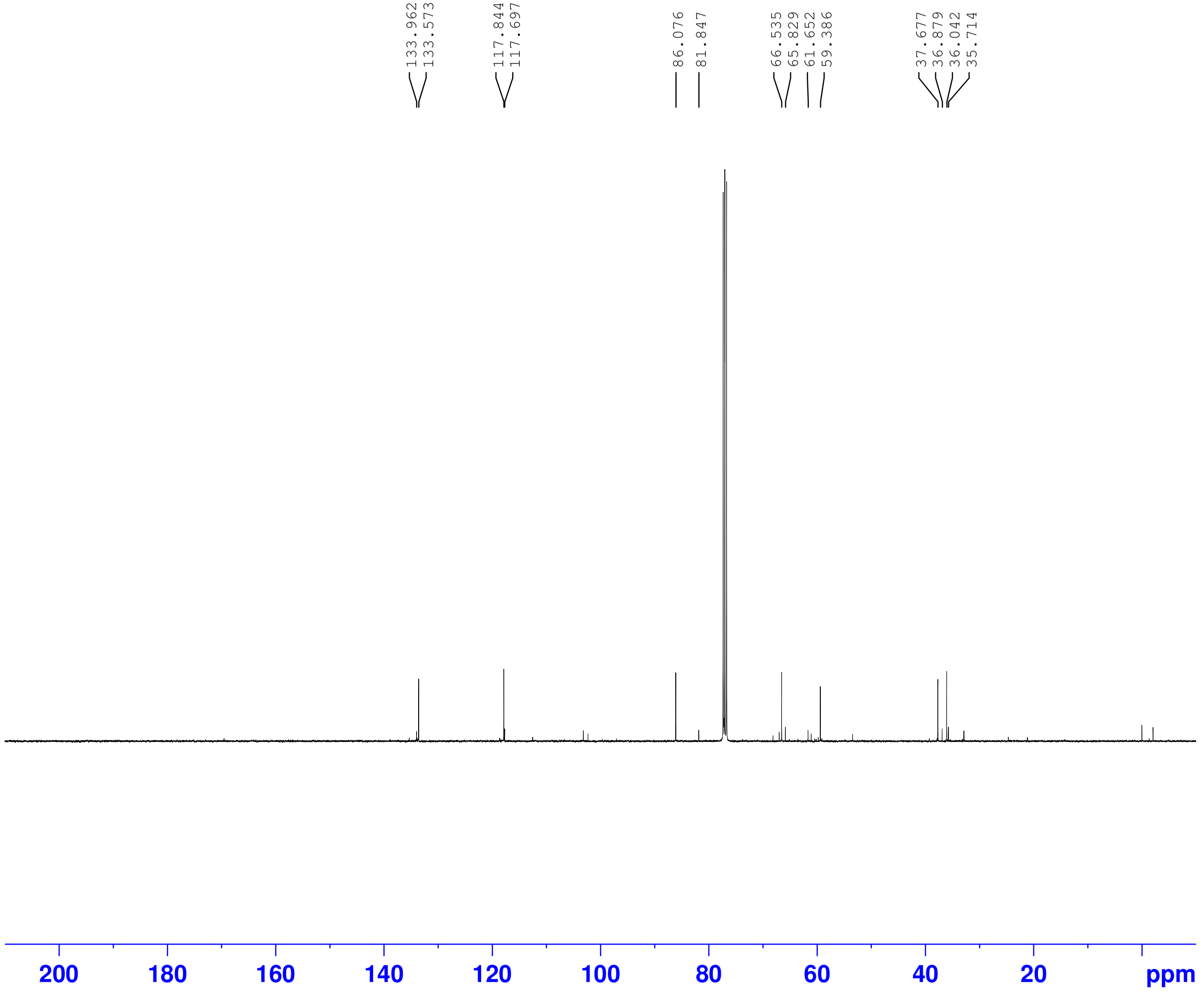


```
13C{1H}

Current Data Parameters
NAME            kmd-7-061
EXPNO                   3
PROCNO                  1

F2 - Acquisition Parameters
Date_            20220616
Time                18.05 h
INSTRUM             spect
PROBHD   Z150354_0001 (
PULPROG           zgpg30
TD                  65356
SOLVENT             CDCl3
NS                    256
DS                      4
SWH             24038.461 Hz
FIDRES           0.735616 Hz
AQ              1.3594048 sec
RG                  45.21
DW                 20.800 usec
DE                  25.00 usec
TE                  298.0 K
D1             2.00000000 sec
D11            0.03000000 sec
TD0                     1
SFO1          100.6655806 MHz
NUC1                  13C
P1                  10.00 usec
PLW1          17.99699974 W
SFO2          400.3016012 MHz
NUC2                   1H
CPDPRG[2          waltz16
PCPD2               80.00 usec
PLW2           4.64209986 W
PLW12          0.10445000 W
PLW13          0.05245300 W

F2 - Processing parameters
SI                  32768
SF            100.6555158 MHz
WDW                    EM
SSB      0
LB                   1.00 Hz
GB       0
PC                   1.40
```

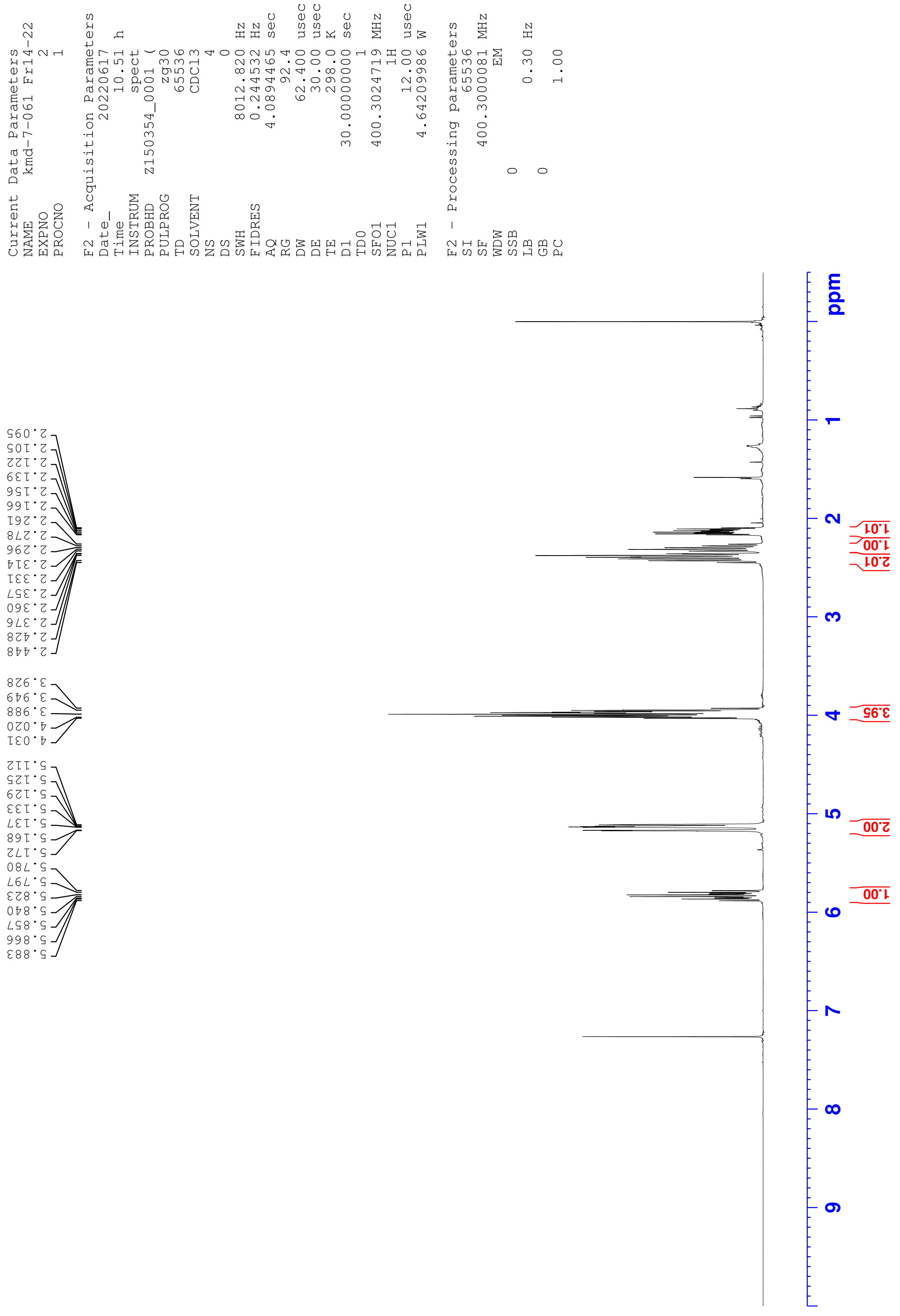
Current Data Parameters
NAME kmd-7-061 Fr14-22
EXPNO 2
PROCNO 1
F2 - Acquisition Parameters
Date_ 20220617
Time 10.51 h
INSTRUM spect
PROBHD Z150354_0001 (
PULPROG zg30
TD 65536
SOLVENT CDCl3
NS 4
DS 0
SWH 8012.820 Hz
FIDRES 0.244532 Hz
AQ 4.0894465 sec
RG 92.4
DW 62.400 usec
DE 30.00 usec
TE 298.0 K
D1 30.00000000 sec
TD0 1
SFO1 400.3024719 MHz
NUC1 1H
P1 12.00 usec
PLW1 4.64209986 W
F2 - Processing parameters
SI 65536
SF 400.3000081 MHz
WDW EM
SSB 0
LB 0.30 Hz
GB 0
PC 1.00
ppm

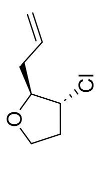

trans-**24a**

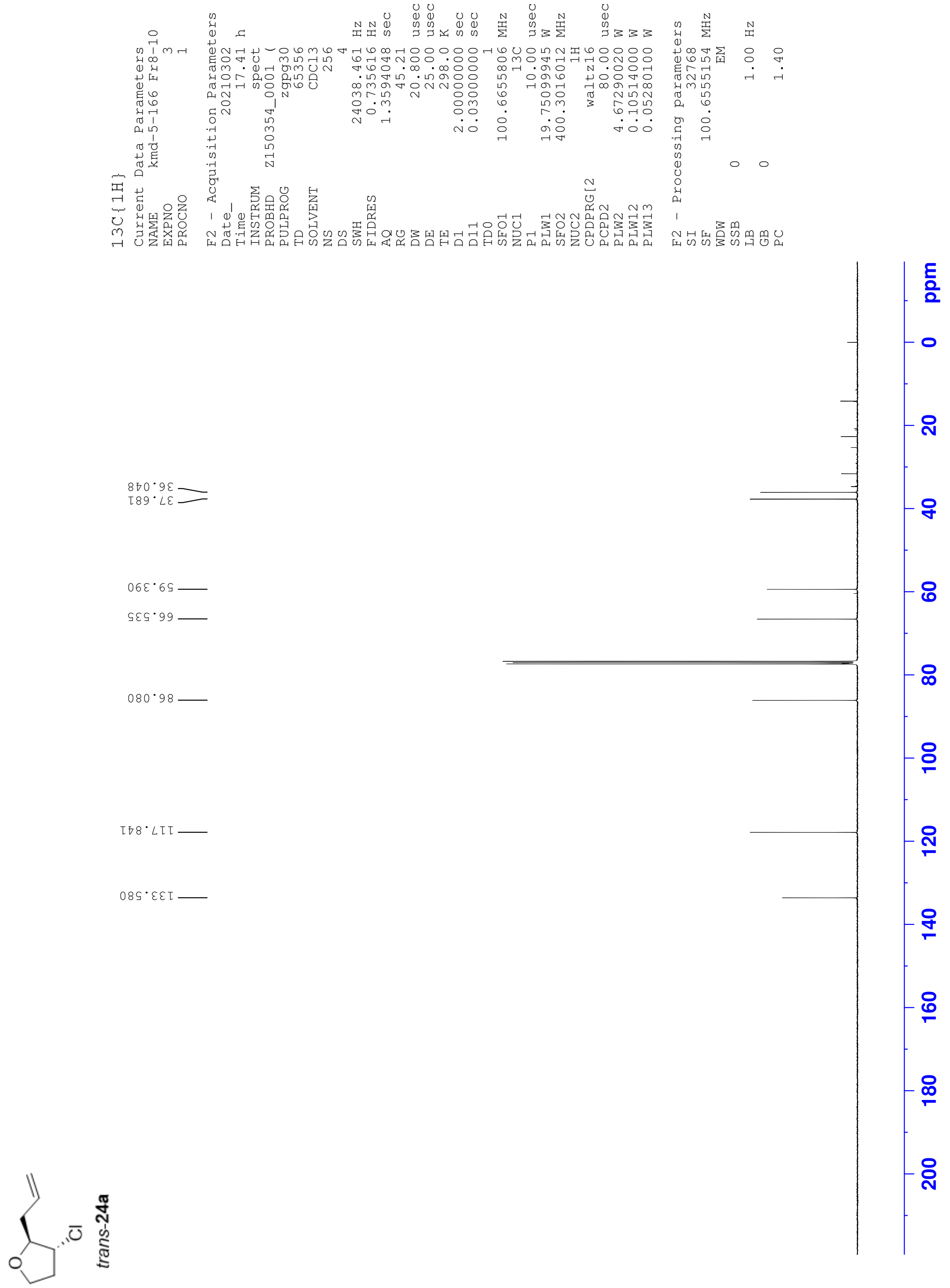
13C{1H}
Current Data Parameters
NAME kmd-5-166 Fr8-10
EXPNO 3
PROCNO 1
F2 - Acquisition Parameters
Date_ 20210302
Time 17.41 h
INSTRUM spect
PROBHD Z150354_0001 (
PULPROG zgpg30
TD 65356
SOLVENT CDCl3
NS 256
DS 4
SWH 24038.461 Hz
FIDRES 0.735616 Hz
AQ 1.3594048 sec
RG 45.21
DW 20.800 usec
DE 25.00 usec
TE 298.0 K
D1 2.00000000 sec
D11 0.03000000 sec
TD0 1
SFO1 100.6655806 MHz
NUC1 13C
P1 10.00 usec
PLW1 19.75099945 W
SFO2 400.3016012 MHz
NUC2 1H
CPDPRG[2 waltz16
PCPD2 80.00 usec
PLW2 4.67290020 W
PLW12 0.10514000 W
PLW13 0.05280100 W
F2 - Processing parameters
SI 32768
SF 100.6555154 MHz
WDW EM
SSB 0
LB 1.00 Hz
GB 0
PC 1.40
133.580
117.841
86.080
66.535
59.390
37.681
36.048
ppm
0
20
40
60
80
100
120
140
160
180
200
O
Cl
trans-24a

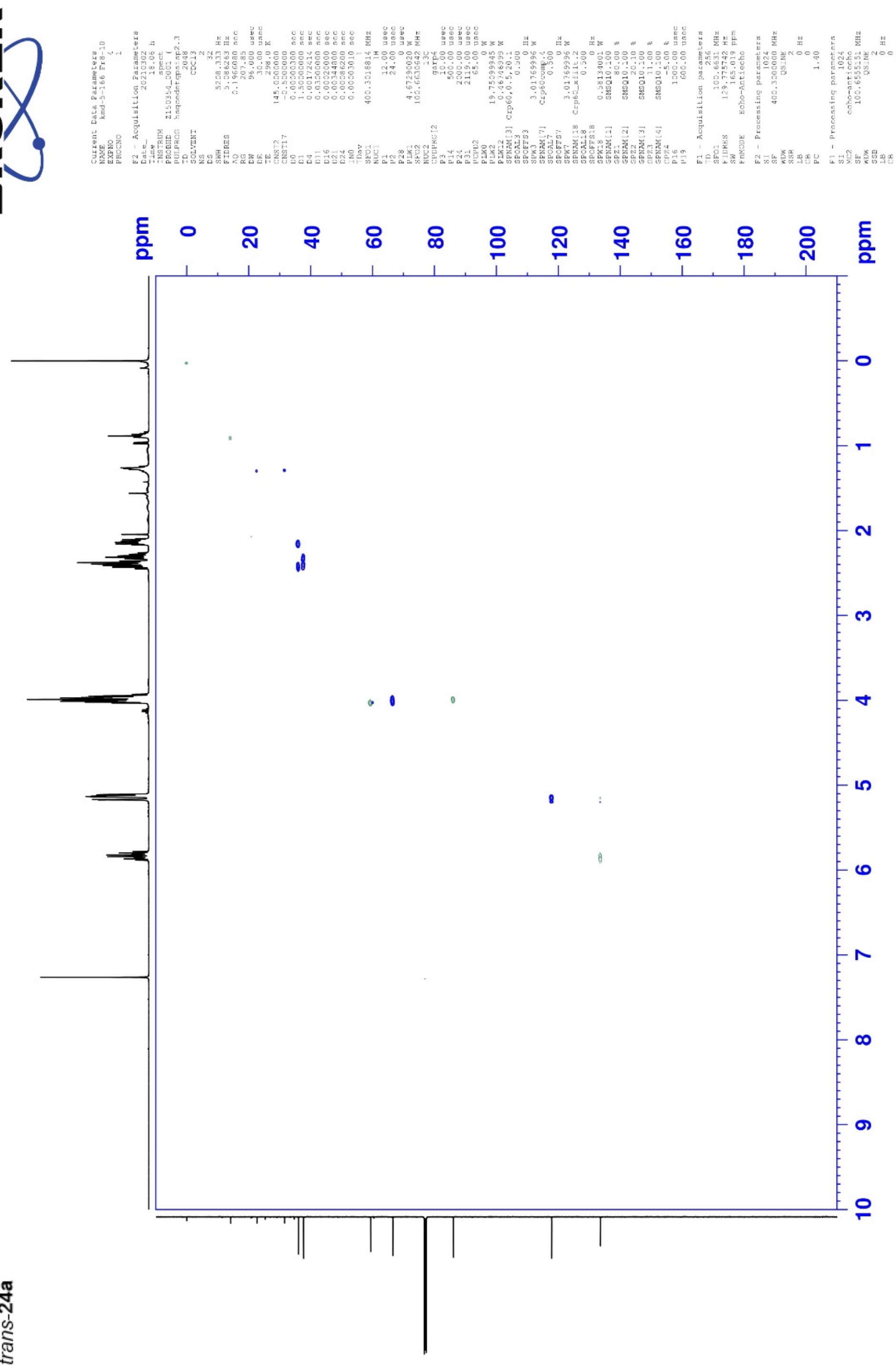

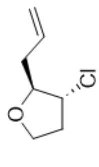

trans-24a

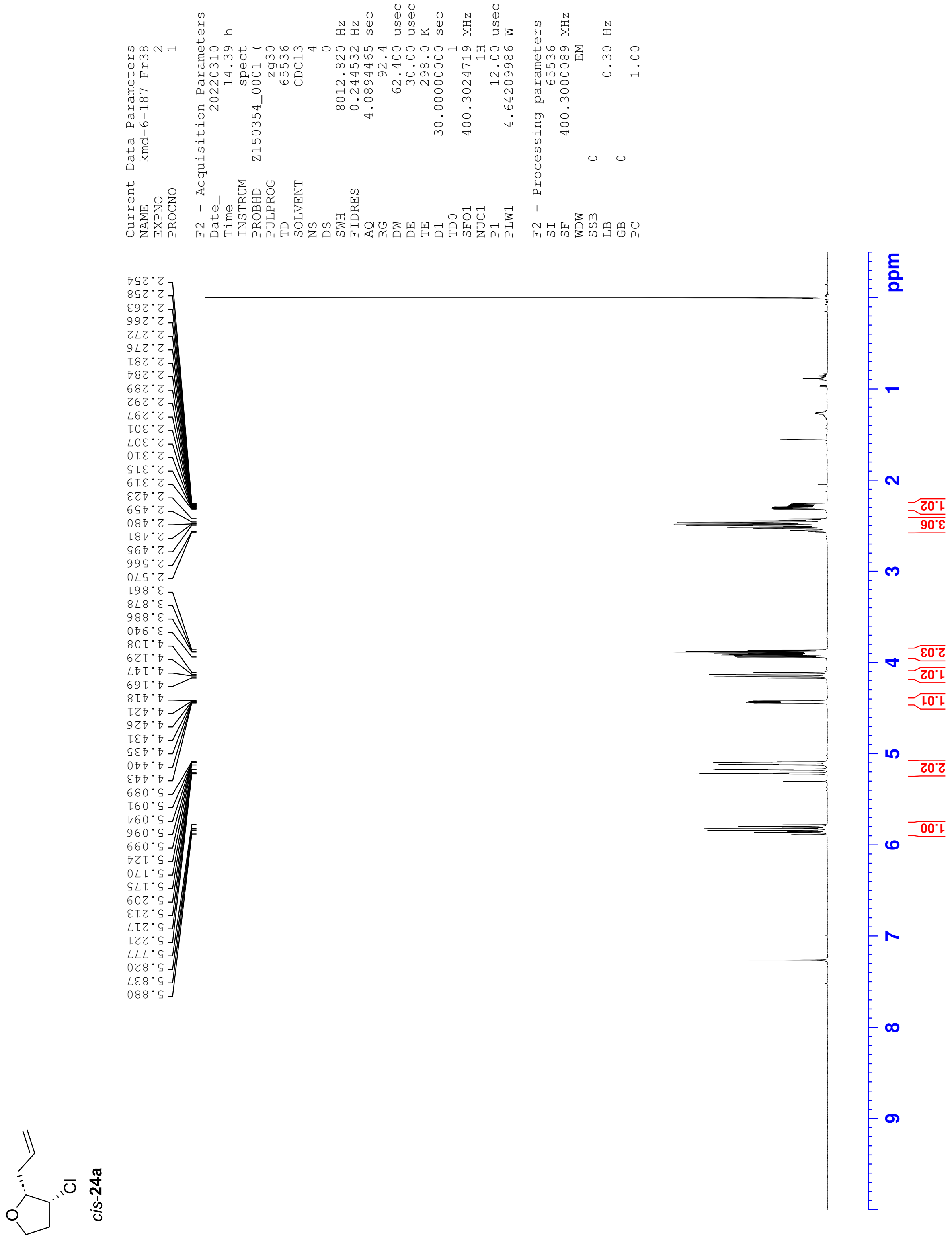
Current Data Parameters
NAME kmd-6-187 Fr38
EXPNO 2
PROCNO 1
F2 - Acquisition Parameters
Date_ 20220310
Time 14.39 h
INSTRUM spect
PROBHD Z150354_0001 (
PULPROG zg30
TD 65536
SOLVENT CDCl3
NS 4
DS 0
SWH 8012.820 Hz
FIDRES 0.244532 Hz
AQ 4.0894465 sec
RG 92.4
DW 62.400 usec
DE 30.00 usec
TE 298.0 K
D1 30.00000000 sec
TD0 1
SFO1 400.3024719 MHz
NUC1 1H
P1 12.00 usec
PLW1 4.64209986 W
F2 - Processing parameters
SI 65536
SF 400.3000089 MHz
WDW EM
SSB 0
LB 0.30 Hz
GB 0
PC 1.00
2.254 2.258 2.263 2.266 2.272 2.276 2.281 2.284 2.289 2.292 2.297 2.301 2.307 2.310 2.315 2.319 2.423 2.459 2.480 2.481 2.495 2.566 2.570 3.861 3.878 3.886 3.940 4.108 4.129 4.147 4.169 4.418 4.421 4.426 4.431 4.435 4.440 4.443 5.089 5.091 5.094 5.096 5.099 5.124 5.170 5.175 5.209 5.213 5.217 5.221 5.777 5.820 5.837 5.880
1.02 3.06 2.03 1.02 1.01 2.02 1.00
ppm
1 2 3 4 5 6 7 8 9
O
Cl

cis-**24a**

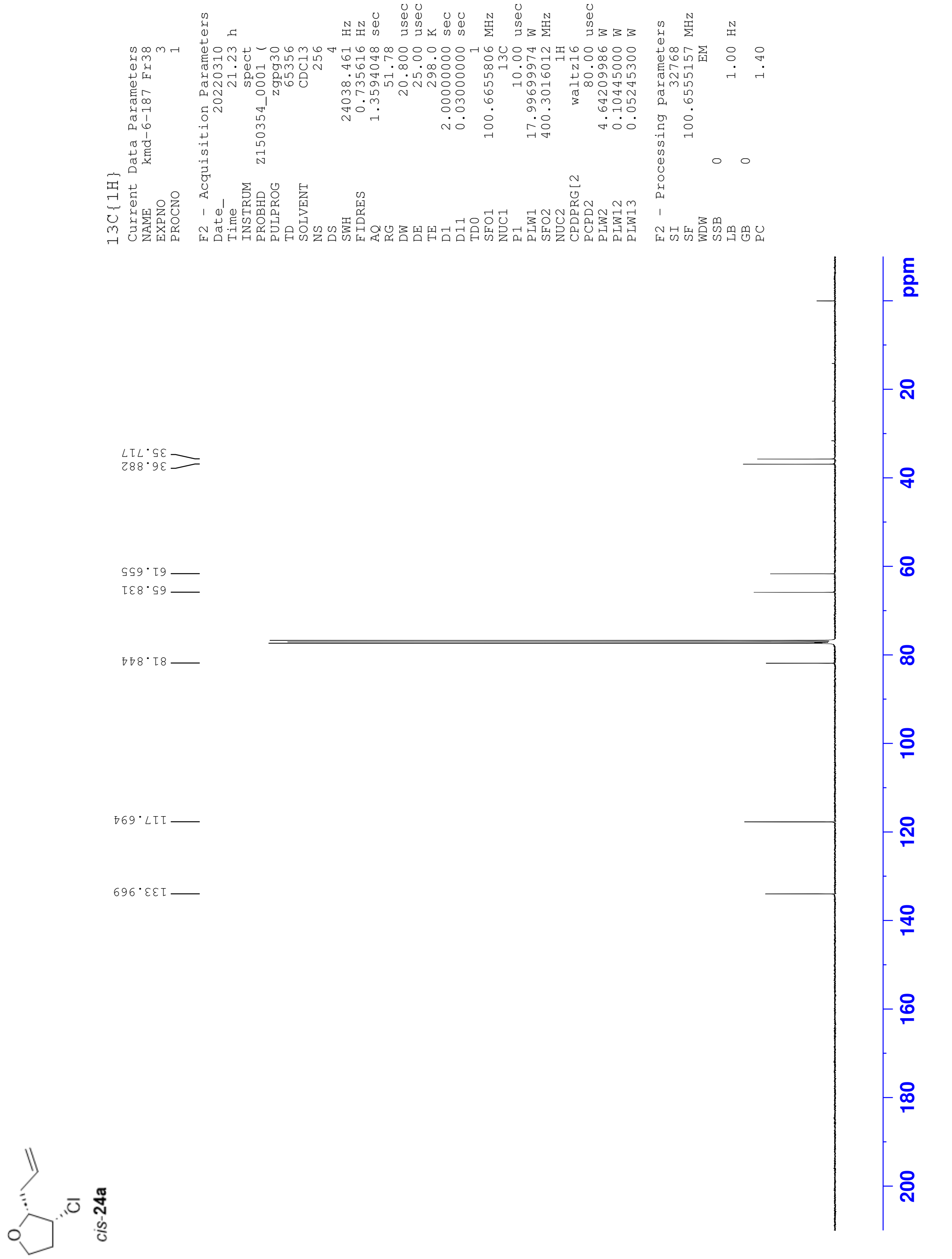
13C{1H}
Current Data Parameters
NAME kmd-6-187 Fr38
EXPNO 3
PROCNO 1
F2 - Acquisition Parameters
Date_ 20220310
Time 21.23 h
INSTRUM spect
PROBHD Z150354_0001 (
PULPROG zgpg30
TD 65356
SOLVENT CDCl3
NS 256
DS 4
SWH 24038.461 Hz
FIDRES 0.735616 Hz
AQ 1.3594048 sec
RG 51.78
DW 20.800 usec
DE 25.00 usec
TE 298.0 K
D1 2.00000000 sec
D11 0.03000000 sec
TD0 1
SFO1 100.6655806 MHz
NUC1 13C
P1 10.00 usec
PLW1 17.99699974 W
SFO2 400.3016012 MHz
NUC2 1H
CPDPRG[2 waltz16
PCPD2 80.00 usec
PLW2 4.64209986 W
PLW12 0.10445000 W
PLW13 0.05245300 W
F2 - Processing parameters
SI 32768
SF 100.6555157 MHz
WDW EM
SSB 0
LB 1.00 Hz
GB 0
PC 1.40
35.717
36.882
61.655
65.831
81.844
117.694
133.969
ppm
20
40
60
80
100
120
140
160
180
200
Cl
O
cis-24a

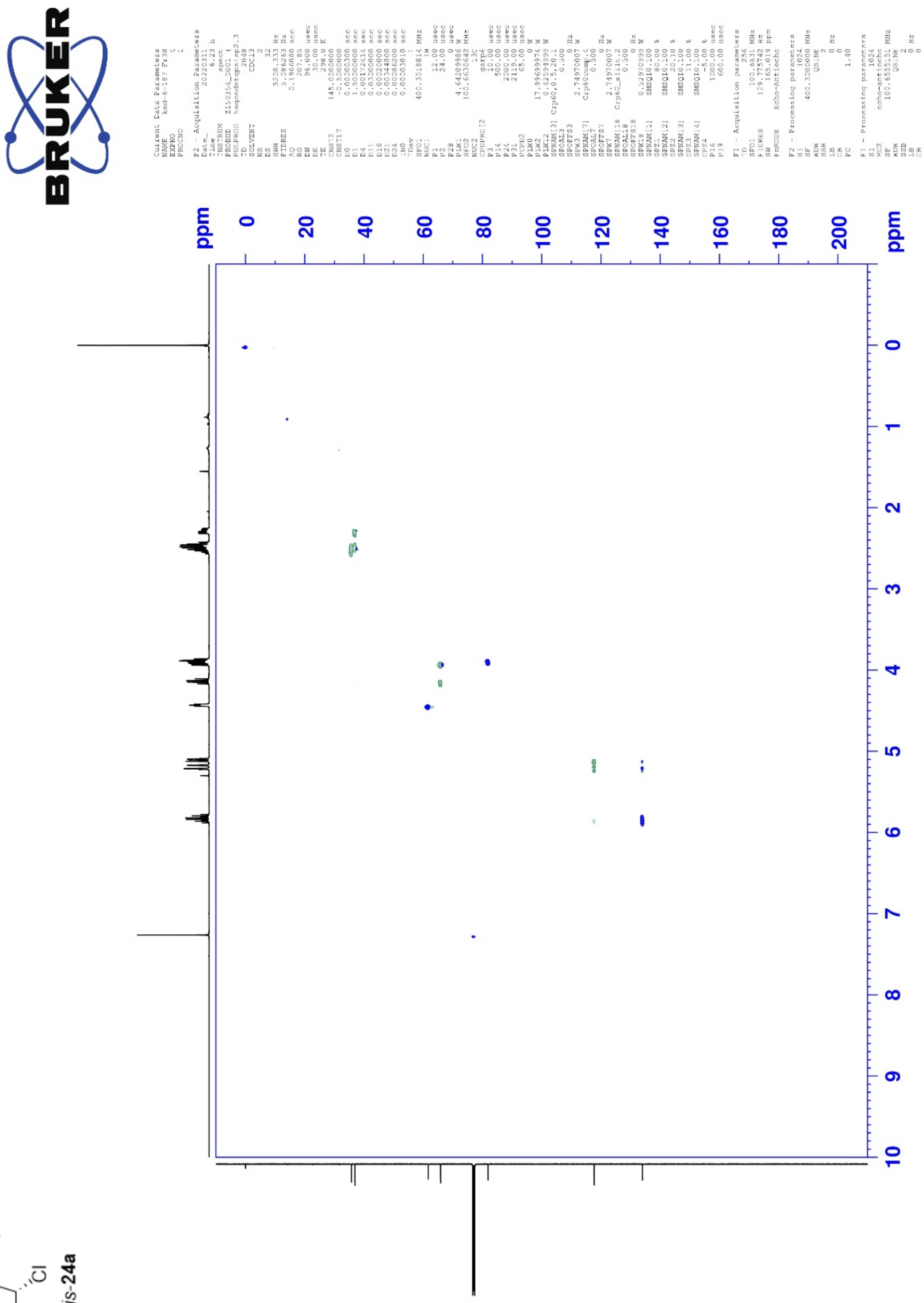

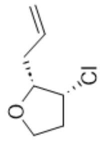

cis-**24a**

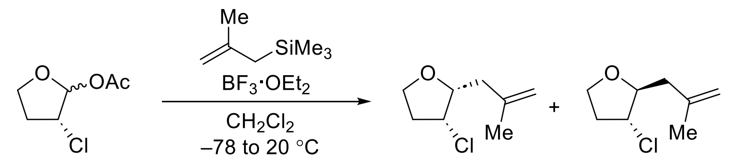

11 *cis*-**24b** *trans*-**24b**

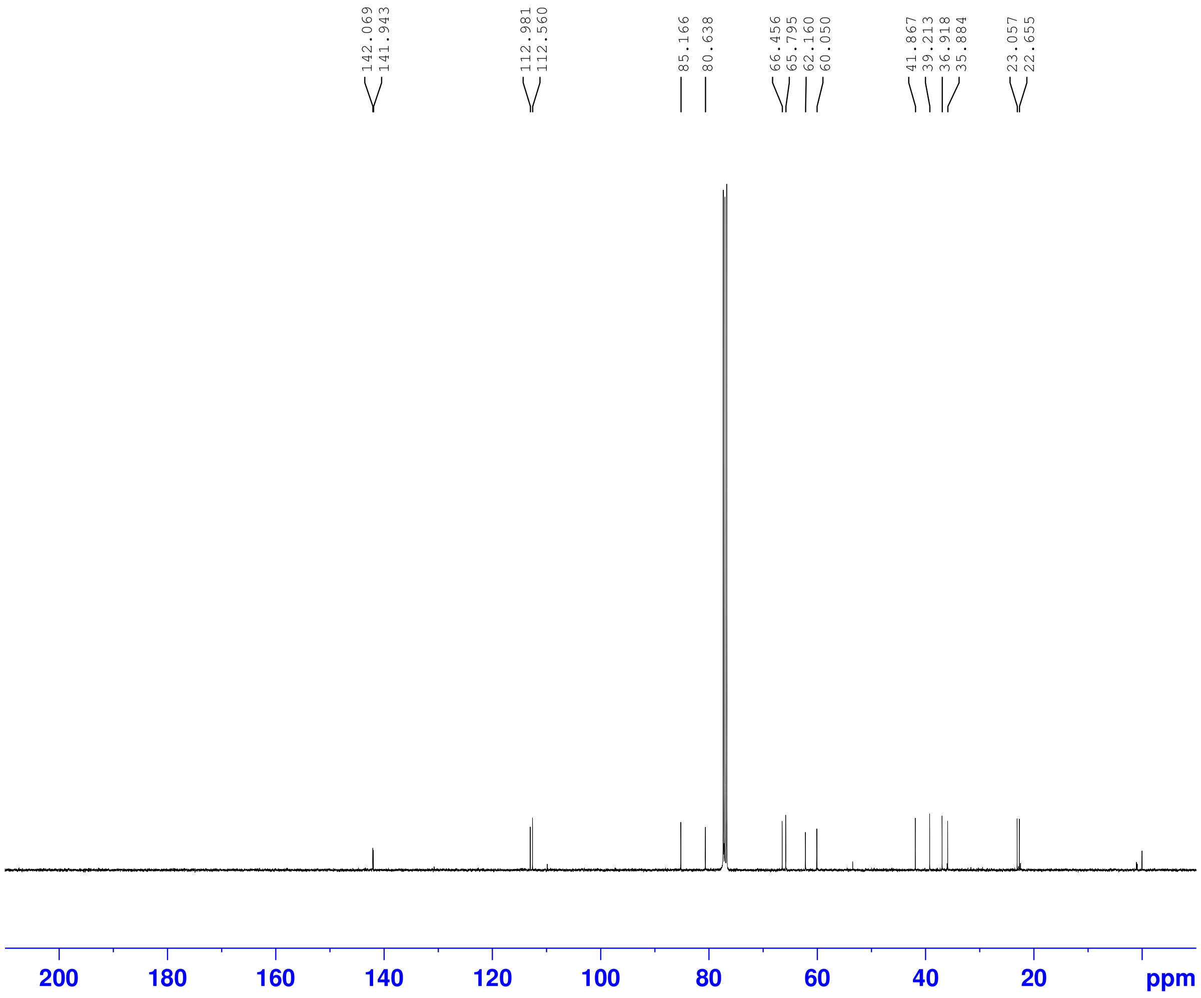


```
13C{1H}
Current Data Parameters
NAME          kmd-3-184
EXPNO                 3
PROCNO                1

F2 - Acquisition Parameters
Date_          20191120
Time              10.45 h
INSTRUM           spect
PROBHD   Z150354_0001 (
PULPROG         zgpg30
TD               65356
SOLVENT          CDCl3
NS                 256
DS                   4
SWH          24038.461 Hz
FIDRES        0.735616 Hz
AQ           1.3594048 sec
RG               29.21
DW              20.800 usec
DE               25.00 usec
TE               298.0 K
D1          2.00000000 sec
D11         0.03000000 sec
TD0                  1
SFO1       100.6655806 MHz
NUC1               13C
P1               10.00 usec
PLW1       19.75099945 W
SFO2       400.3016012 MHz
NUC2                1H
CPDPRG[2       waltz16
PCPD2            80.00 usec
PLW2        4.67290020 W
PLW12       0.10514000 W
PLW13       0.05280100 W

F2 - Processing parameters
SI               32768
SF         100.6555157 MHz
WDW                 EM
SSB      0
LB                1.00 Hz
GB       0
PC                1.40
```

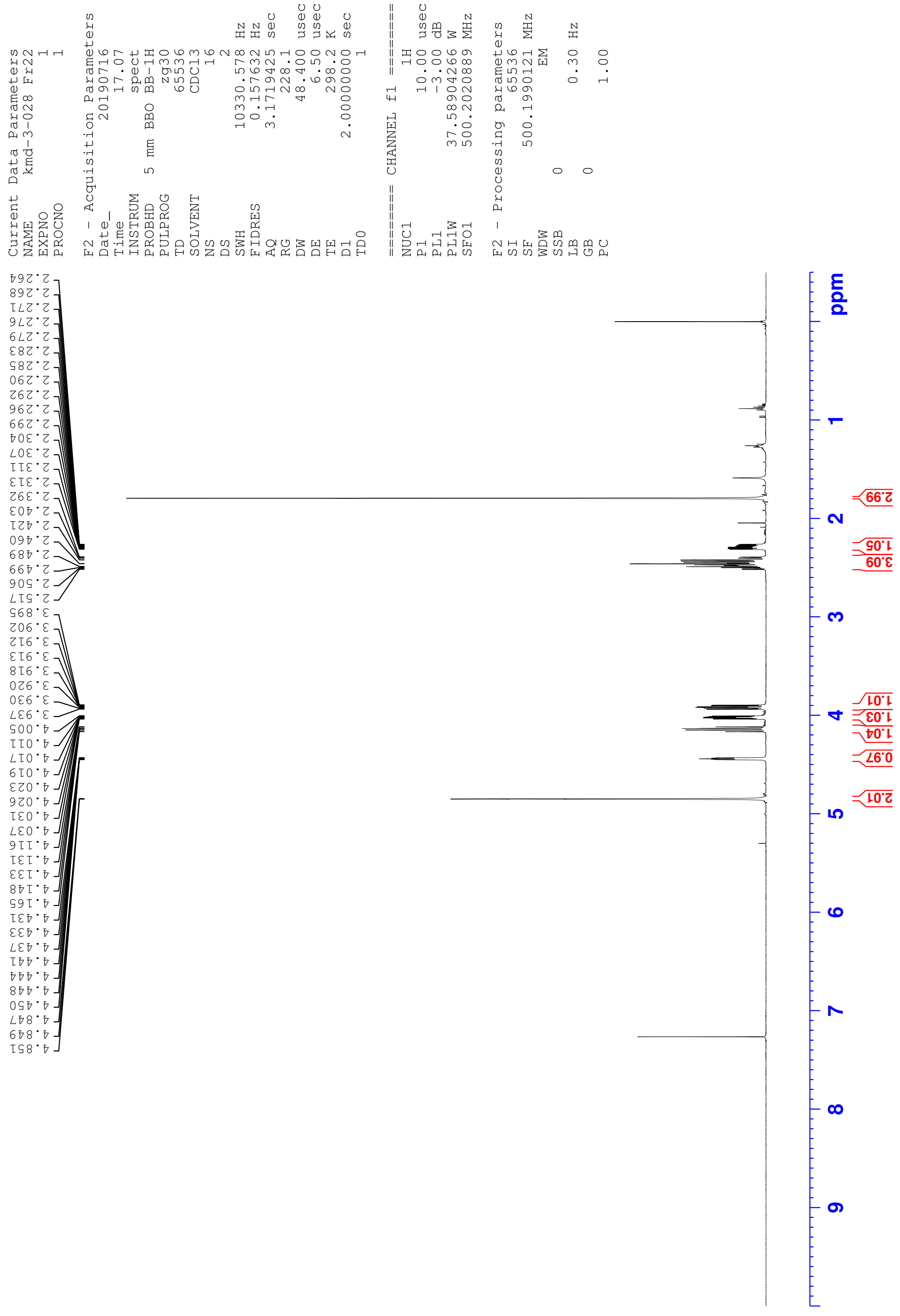
Current Data Parameters
NAME kmd-3-028 Fr22
EXPNO 1
PROCNO 1
F2 - Acquisition Parameters
Date_ 20190716
Time 17.07
INSTRUM spect
PROBHD 5 mm BBO BB-1H
PULPROG zg30
TD 65536
SOLVENT CDCl3
NS 16
DS 2
SWH 10330.578 Hz
FIDRES 0.157632 Hz
AQ 3.1719425 sec
RG 228.1
DW 48.400 usec
DE 6.50 usec
TE 298.2 K
D1 2.00000000 sec
TD0 1
======== CHANNEL f1 ========
NUC1 1H
P1 10.00 usec
PL1 -3.00 dB
PL1W 37.58904266 W
SFO1 500.2020889 MHz
F2 - Processing parameters
SI 65536
SF 500.1990121 MHz
WDW EM
SSB 0
LB 0.30 Hz
GB 0
PC 1.00
2.264 2.268 2.271 2.276 2.279 2.283 2.285 2.290 2.292 2.296 2.299 2.304 2.307 2.311 2.313 2.392 2.403 2.421 2.460 2.489 2.499 2.506 2.517
3.895 3.902 3.912 3.913 3.918 3.920 3.930 3.937 4.005 4.011 4.017 4.019 4.023 4.026 4.031 4.037 4.116 4.131 4.133 4.148 4.165 4.431 4.433 4.437 4.441 4.444 4.448 4.450 4.847 4.849 4.851
2.99 1.05 3.09 1.01 1.03 1.04 0.97 2.01
ppm
9 8 7 6 5 4 3 2 1

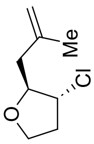
O
Cl
Me
trans-**24b**

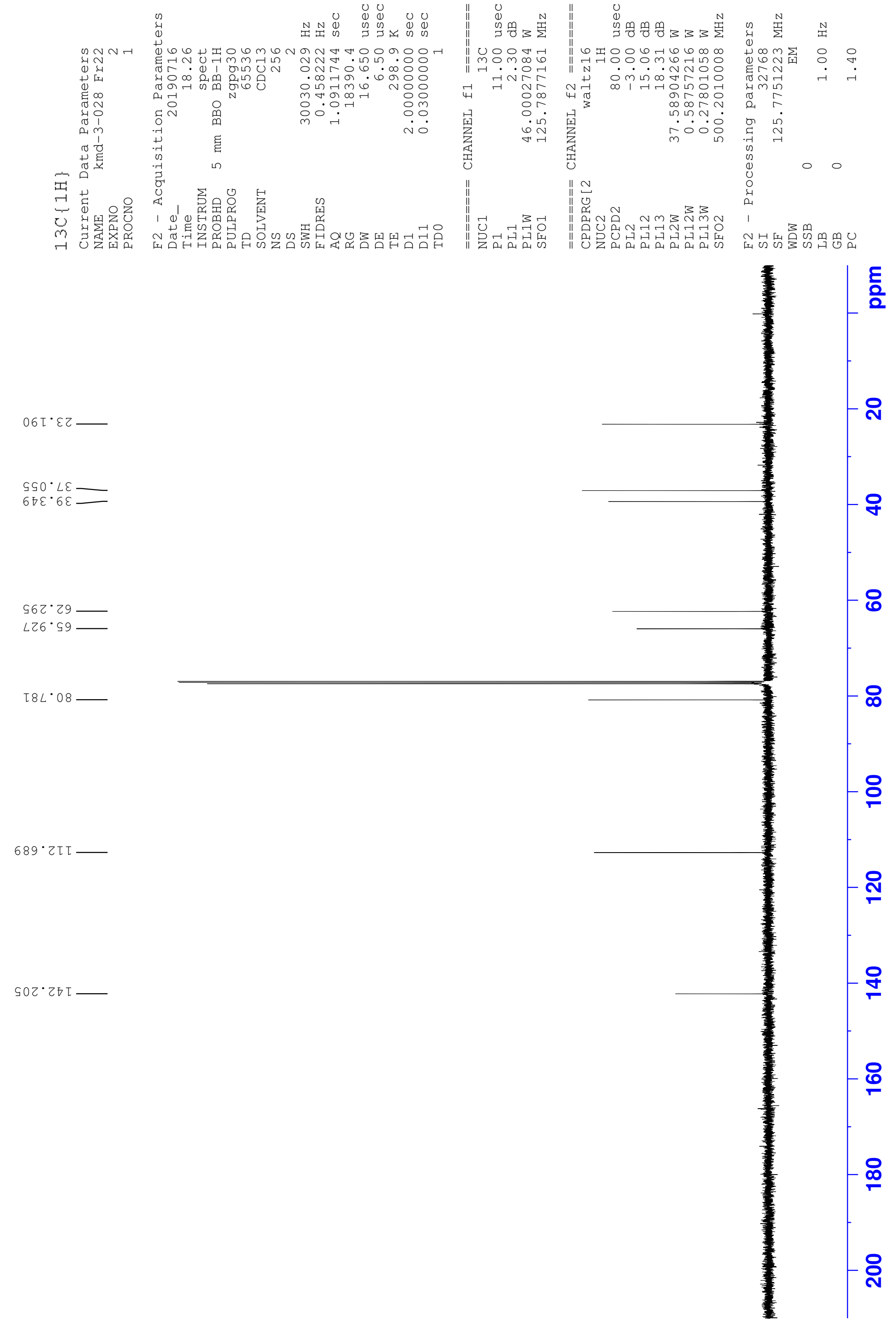
13C{1H}
Current Data Parameters
NAME kmd-3-028 Fr22
EXPNO 2
PROCNO 1
F2 - Acquisition Parameters
Date_ 20190716
Time 18.26
INSTRUM spect
PROBHD 5 mm BBO BB-1H
PULPROG zgpg30
TD 65536
SOLVENT CDCl3
NS 256
DS 2
SWH 30030.029 Hz
FIDRES 0.458222 Hz
AQ 1.0911744 sec
RG 18390.4
DW 16.650 usec
DE 6.50 usec
TE 298.9 K
D1 2.00000000 sec
D11 0.03000000 sec
TD0 1
======== CHANNEL f1 ========
NUC1 13C
P1 11.00 usec
PL1 2.30 dB
PL1W 46.00027084 W
SFO1 125.7877161 MHz
======== CHANNEL f2 ========
CPDPRG[2 waltz16
NUC2 1H
PCPD2 80.00 usec
PL2 -3.00 dB
PL12 15.06 dB
PL13 18.31 dB
PL2W 37.58904266 W
PL12W 0.58757216 W
PL13W 0.27801058 W
SFO2 500.2010008 MHz
F2 - Processing parameters
SI 32768
SF 125.7751223 MHz
WDW EM
SSB 0
LB 1.00 Hz
GB 0
PC 1.40
23.190
37.055
39.349
62.295
65.927
80.781
112.689
142.205
20
40
60
80
100
120
140
160
180
200
ppm

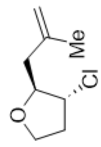
O
Cl
Me
trans-**24b**

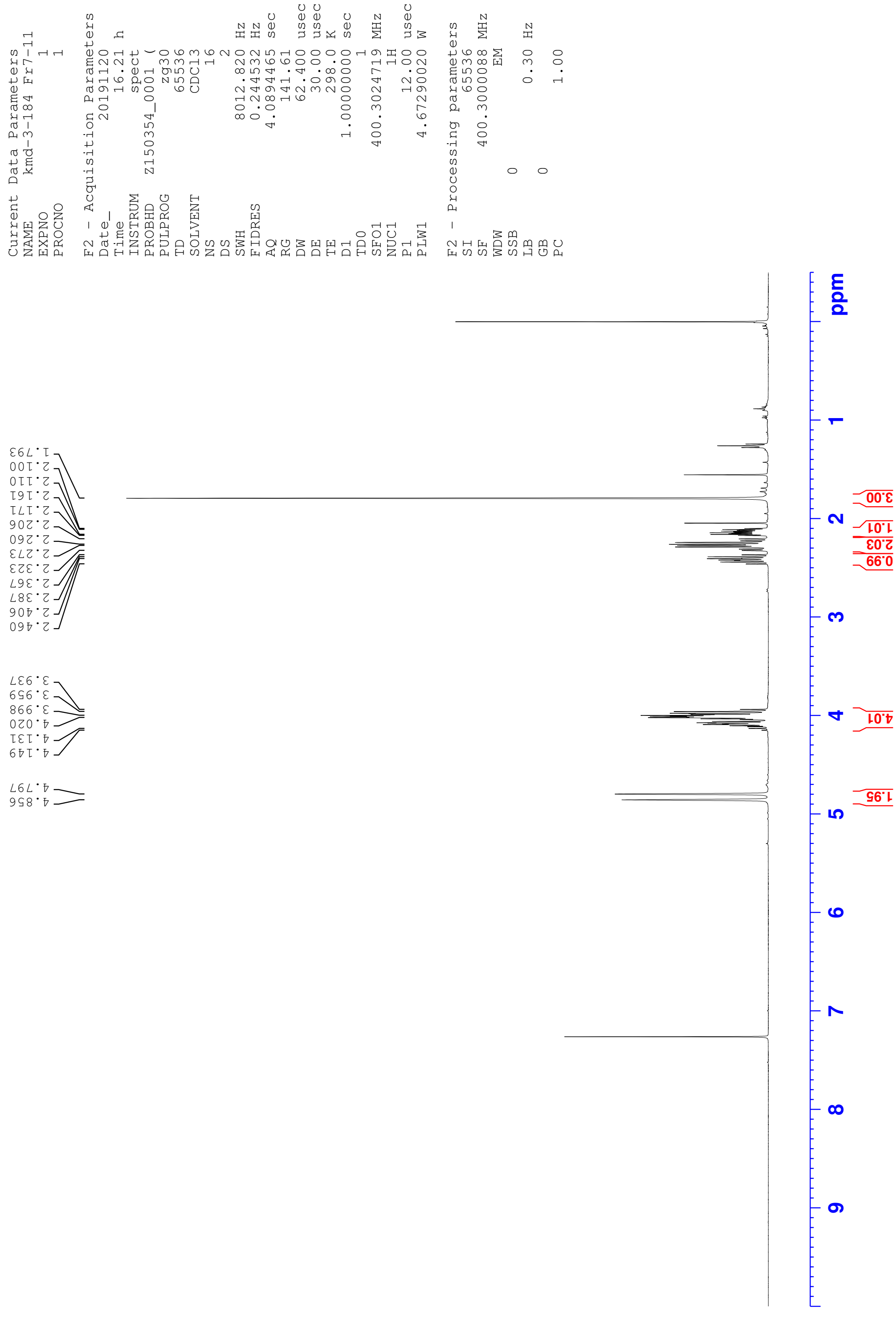
Current Data Parameters
NAME kmd-3-184 Fr7-11
EXPNO 1
PROCNO 1
F2 - Acquisition Parameters
Date_ 20191120
Time 16.21 h
INSTRUM spect
PROBHD Z150354_0001 (
PULPROG zg30
TD 65536
SOLVENT CDCl3
NS 16
DS 2
SWH 8012.820 Hz
FIDRES 0.244532 Hz
AQ 4.0894465 sec
RG 141.61
DW 62.400 usec
DE 30.00 usec
TE 298.0 K
D1 1.00000000 sec
TD0 1
SFO1 400.3024719 MHz
NUC1 1H
P1 12.00 usec
PLW1 4.67290020 W
F2 - Processing parameters
SI 65536
SF 400.3000088 MHz
WDW EM
SSB 0
LB 0.30 Hz
GB 0
PC 1.00
1.793
2.100
2.110
2.161
2.171
2.206
2.260
2.273
2.323
2.367
2.387
2.406
2.460
3.937
3.959
3.998
4.020
4.131
4.149
4.797
4.856
3.00
1.01
2.03
0.99
4.01
1.95
ppm
1
2
3
4
5
6
7
8
9

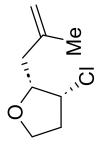
Cl
Me

cis-**24b**

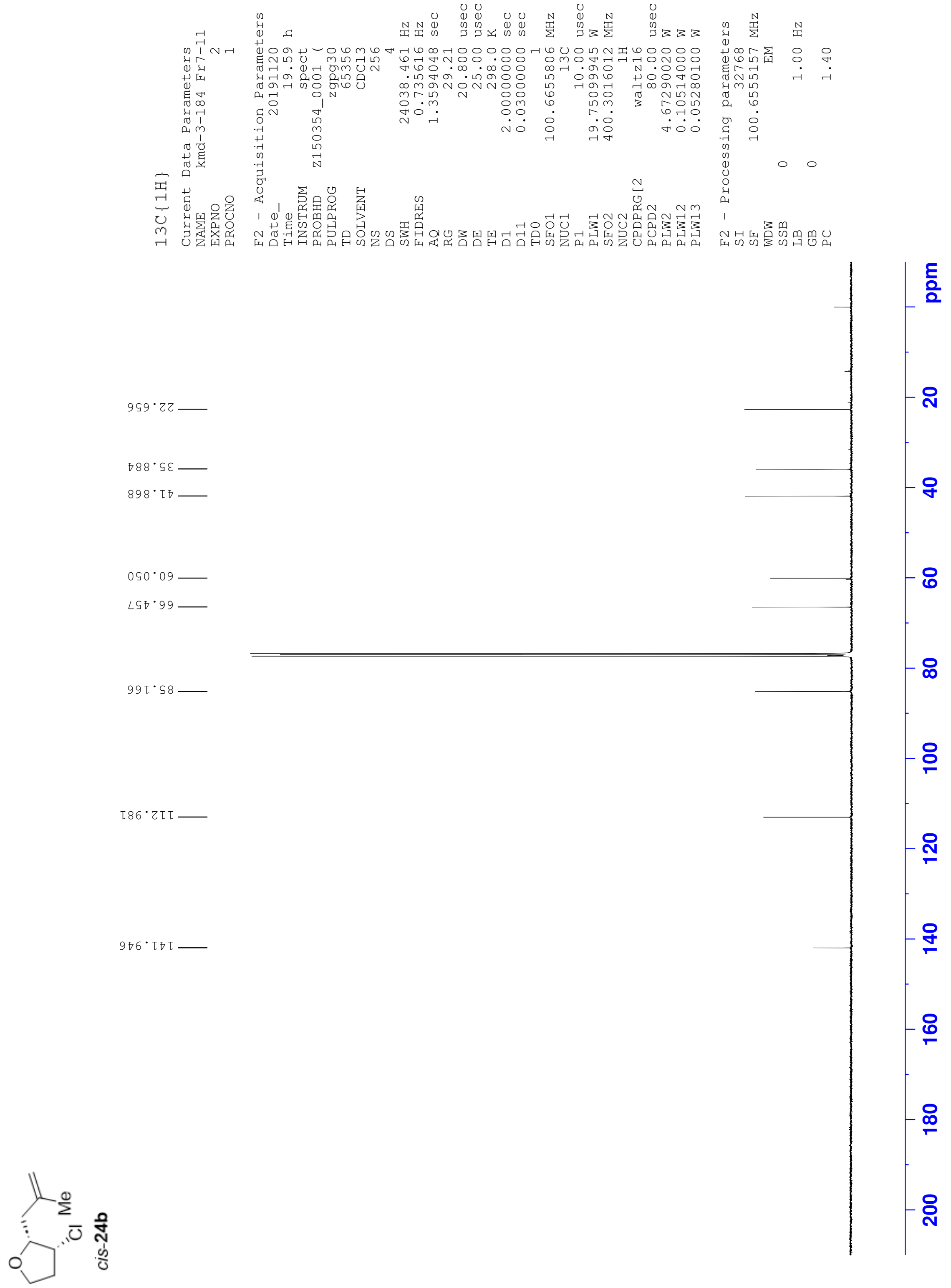
13C{1H}
Current Data Parameters
NAME kmd-3-184 Fr7-11
EXPNO 2
PROCNO 1
F2 - Acquisition Parameters
Date_ 20191120
Time_ 19.59 h
INSTRUM spect
PROBHD Z150354_0001 (
PULPROG zgpg30
TD 65356
SOLVENT CDCl3
NS 256
DS 4
SWH 24038.461 Hz
FIDRES 0.735616 Hz
AQ 1.3594048 sec
RG 29.21
DW 20.800 usec
DE 25.00 usec
TE 298.0 K
D1 2.00000000 sec
D11 0.03000000 sec
TD0 1
SFO1 100.6655806 MHz
NUC1 13C
P1 10.00 usec
PLW1 19.75099945 W
SFO2 400.3016012 MHz
NUC2 1H
CPDPRG[2 waltz16
PCPD2 80.00 usec
PLW2 4.67290020 W
PLW12 0.10514000 W
PLW13 0.05280100 W
F2 - Processing parameters
SI 32768
SF 100.6555157 MHz
WDW EM
SSB 0
LB 1.00 Hz
GB 0
PC 1.40
22.656
35.884
41.868
60.050
66.457
85.166
112.981
141.946
ppm
20
40
60
80
100
120
140
160
180
200
O
Cl
Me
cis-24b

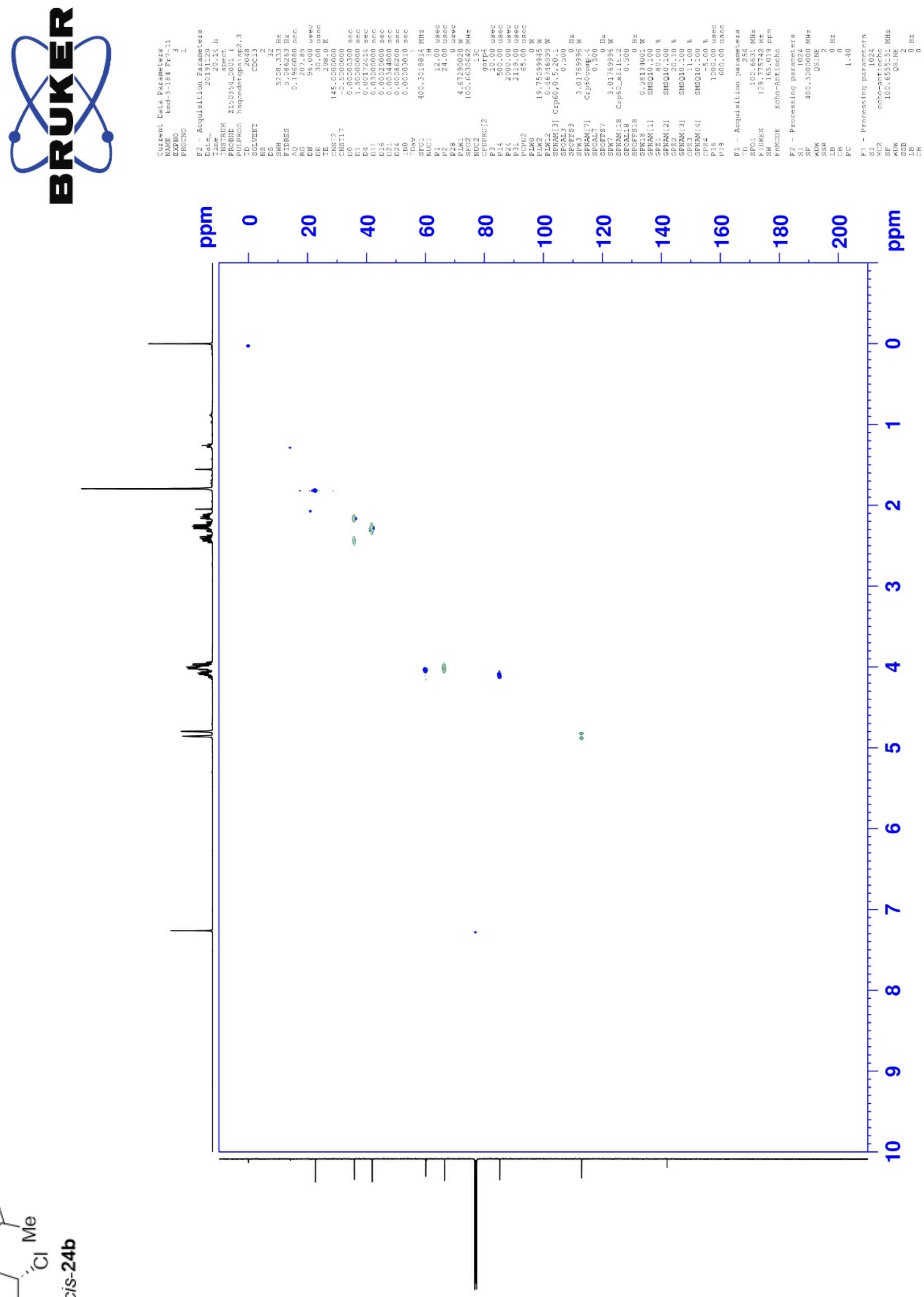

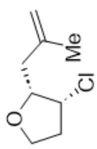

cis-**24b**

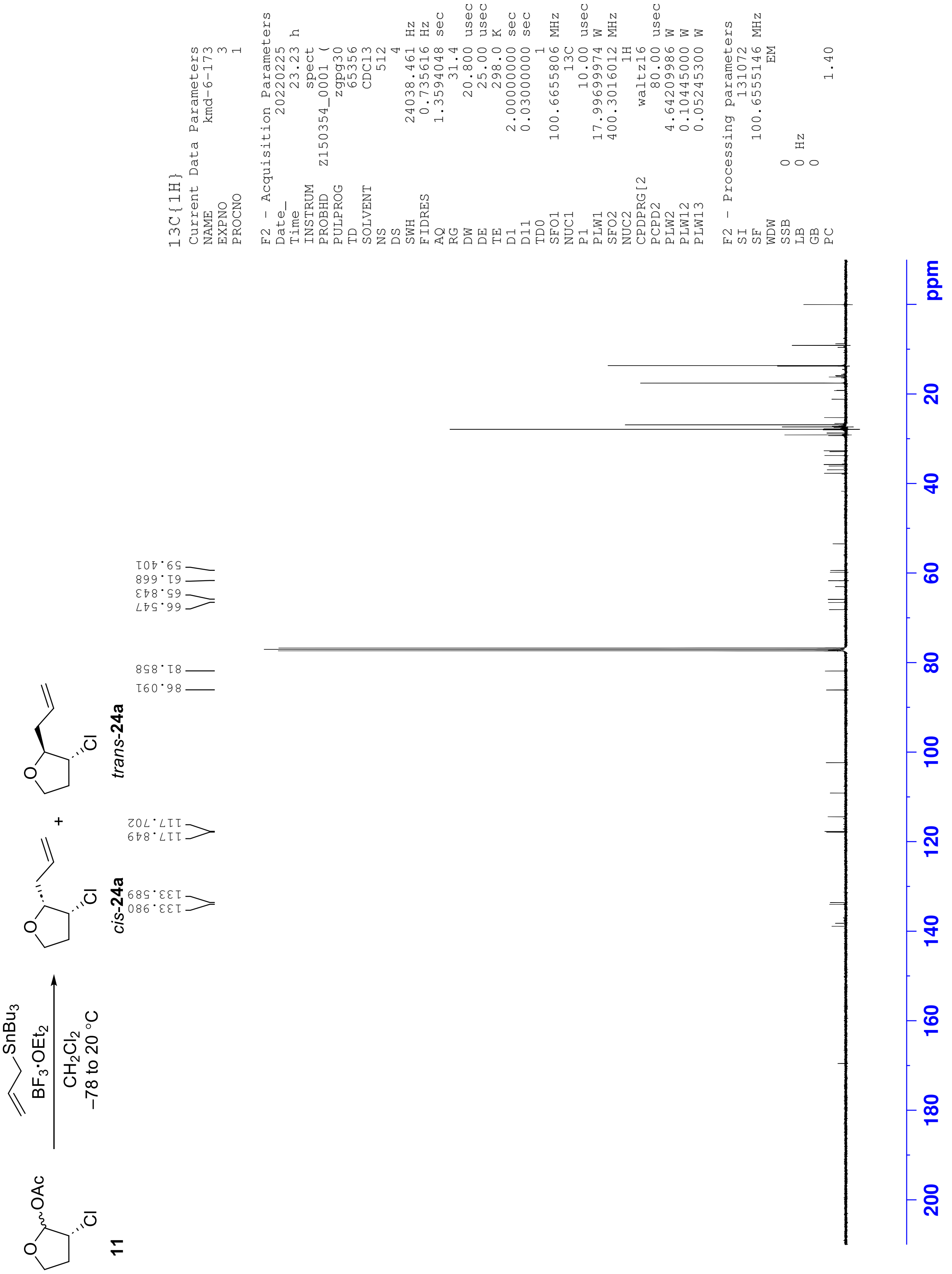
OAc
Cl
11
SnBu3
BF3•OEt2
CH2Cl2
–78 to 20 °C
Cl
cis-24a
+
Cl
trans-24a
133.980
133.589
117.849
117.702
86.091
81.858
66.547
65.843
61.668
59.401
13C{1H}
Current Data Parameters
NAME kmd-6-173
EXPNO 3
PROCNO 1
F2 - Acquisition Parameters
Date_ 20220225
Time 23.23 h
INSTRUM spect
PROBHD Z150354_0001 (
PULPROG zgpg30
TD 65356
SOLVENT CDCl3
NS 512
DS 4
SWH 24038.461 Hz
FIDRES 0.735616 Hz
AQ 1.3594048 sec
RG 31.4
DW 20.800 usec
DE 25.00 usec
TE 298.0 K
D1 2.00000000 sec
D11 0.03000000 sec
TD0 1
SFO1 100.6655806 MHz
NUC1 13C
P1 10.00 usec
PLW1 17.99699974 W
SFO2 400.3016012 MHz
NUC2 1H
CPDPRG[2 waltz16
PCPD2 80.00 usec
PLW2 4.64209986 W
PLW12 0.10445000 W
PLW13 0.05245300 W
F2 - Processing parameters
SI 131072
SF 100.6555146 MHz
WDW EM
SSB 0
LB 0 Hz
GB 0
PC 1.40
200
180
160
140
120
100
80
60
40
20
ppm

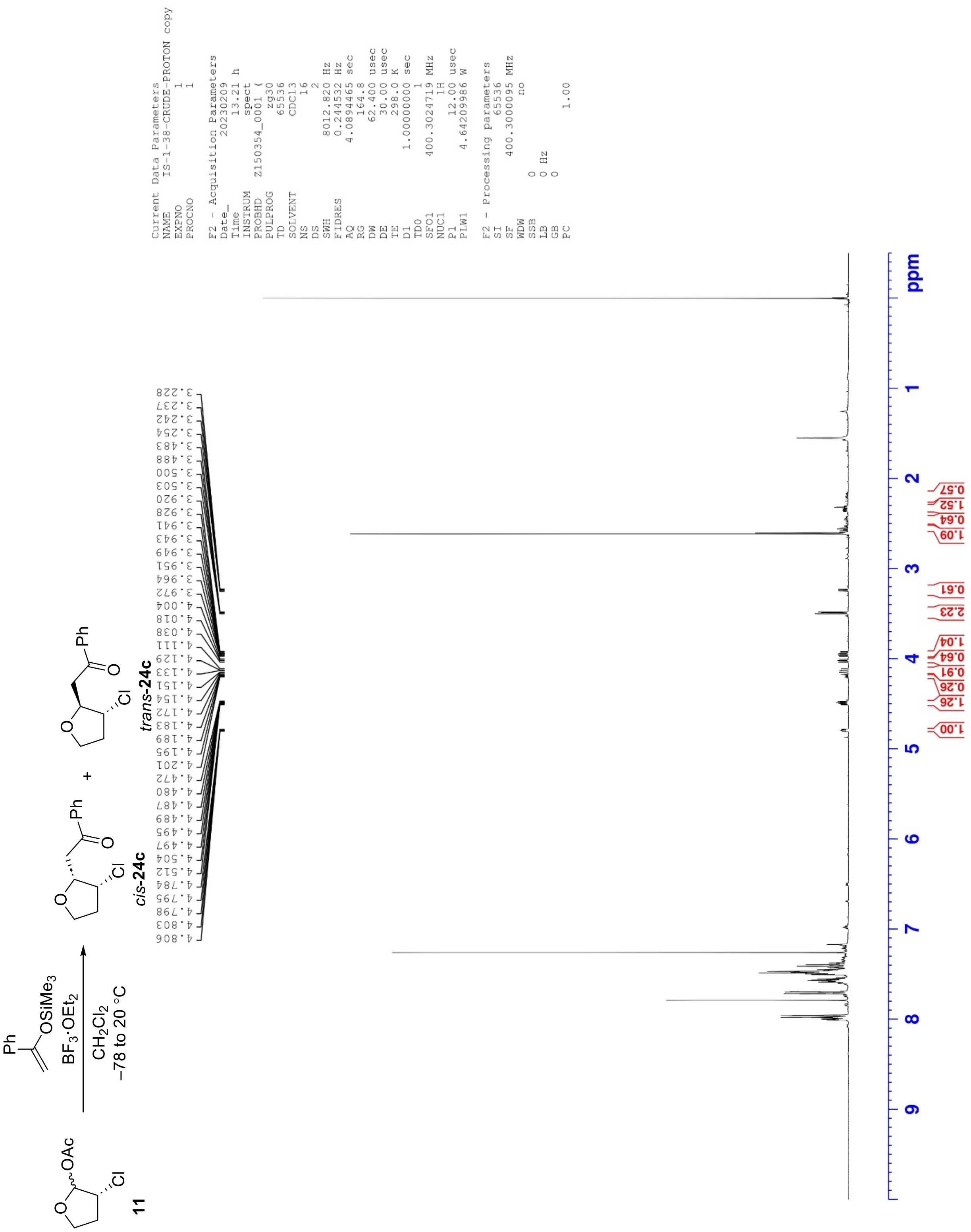
11
BF3·OEt2
CH2Cl2
−78 to 20 °C
OSiMe3
cis-24c
trans-24c
Current Data Parameters
NAME IS-1-38-CRUDE-PROTON copy
EXPNO 1
PROCNO 1
F2 - Acquisition Parameters
Date_ 20230209
Time 13.21 h
INSTRUM spect
PROBHD Z150354_0001 (
PULPROG zg30
TD 65536
SOLVENT CDCl3
NS 16
DS 2
SWH 8012.820 Hz
FIDRES 0.244532 Hz
AQ 4.0894465 sec
RG 164.8
DW 62.400 usec
DE 30.00 usec
TE 298.0 K
D1 1.00000000 sec
TD0 1
SFO1 400.3024719 MHz
NUC1 1H
P1 12.00 usec
PLW1 4.64209986 W
F2 - Processing parameters
SI 65536
SF 400.3000095 MHz
WDW no
SSB 0
LB 0 Hz
GB 0
PC 1.00
ppm

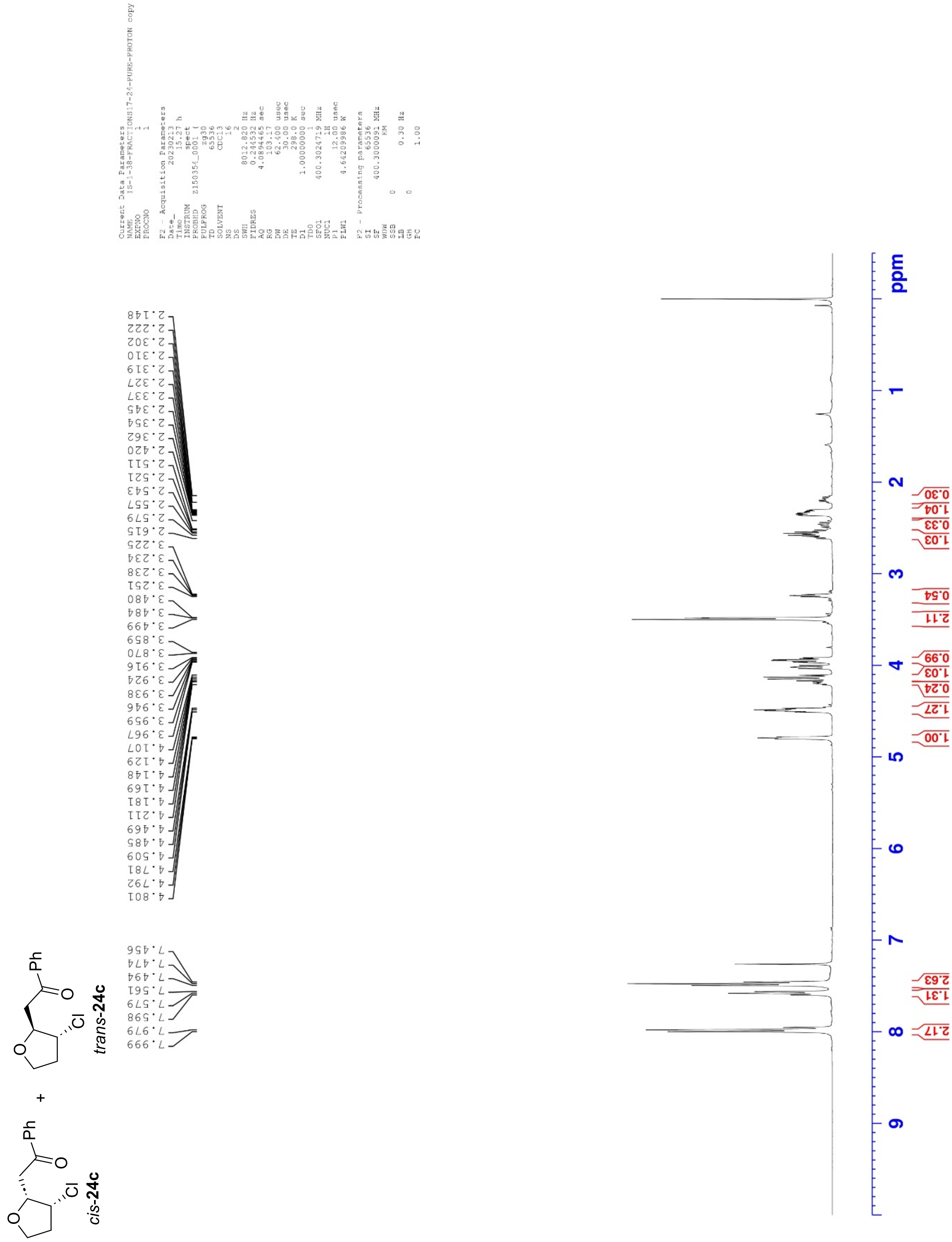
cis-24c
trans-24c
Ph
O
Cl
ppm

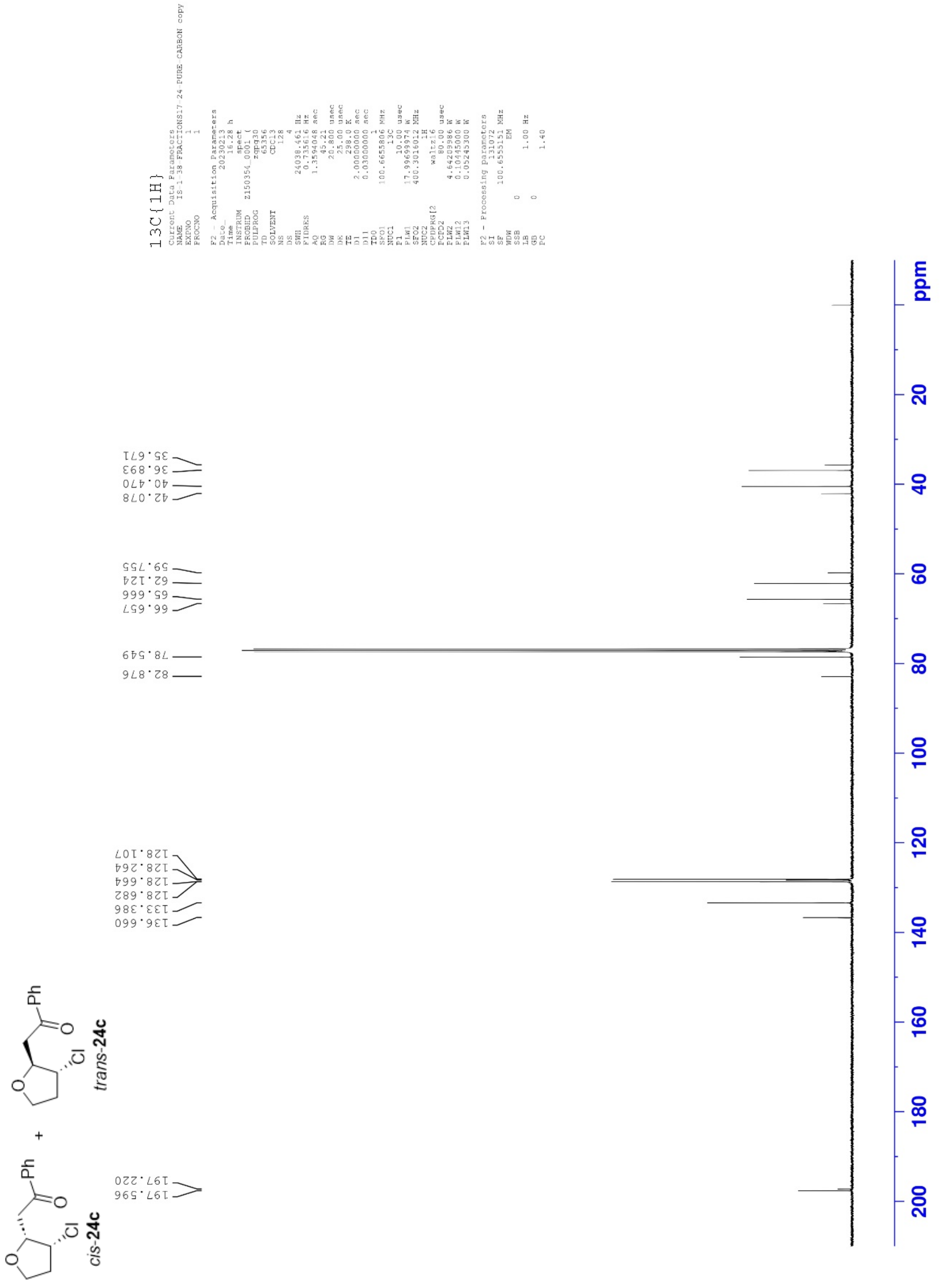
13C{1H}
35.671
36.893
40.470
42.078
59.755
62.124
65.666
66.657
78.549
82.876
128.107
128.264
128.664
128.682
133.386
136.660
197.220
197.596
cis-24c
trans-24c
ppm

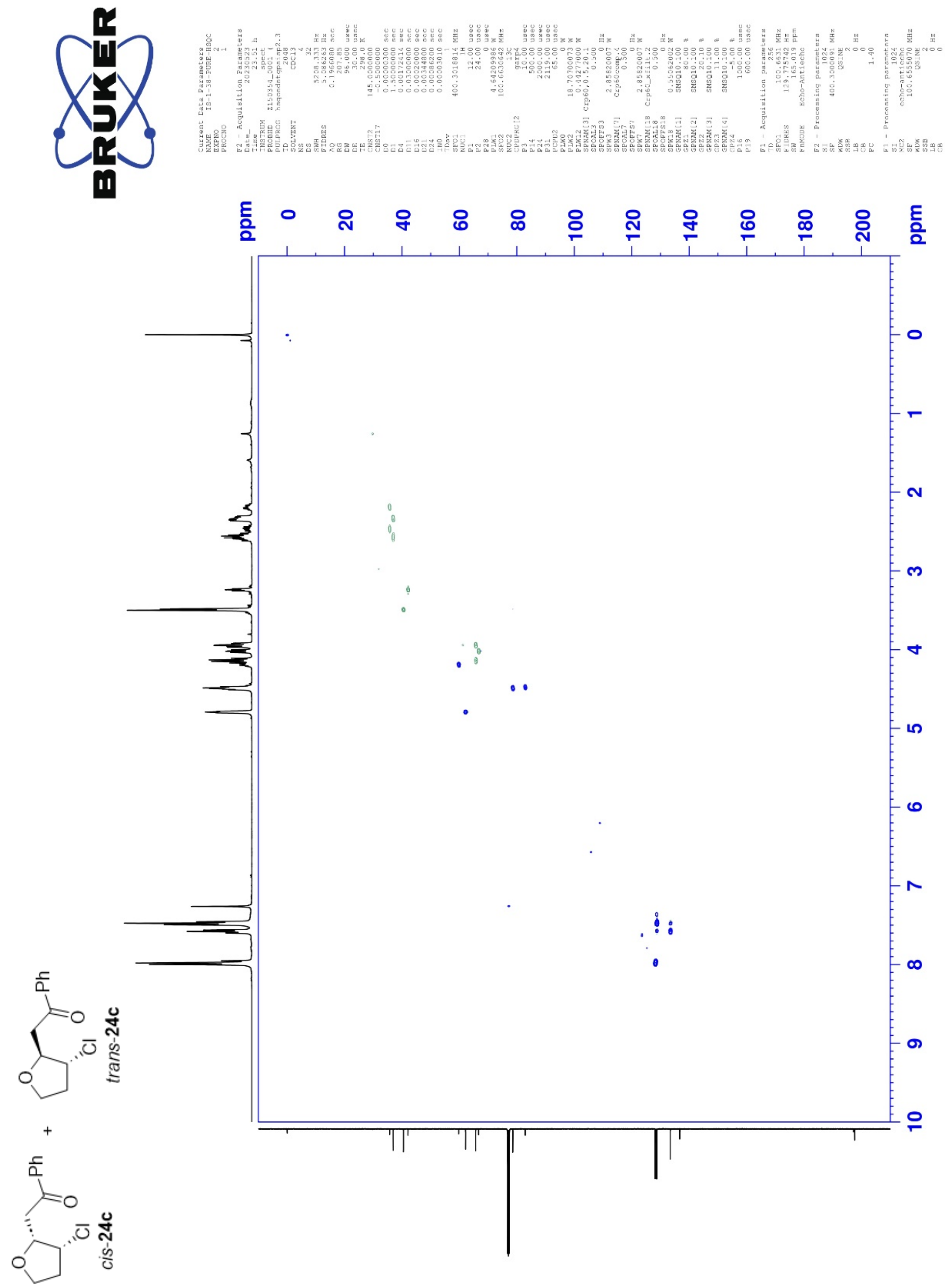
cis-24c
trans-24c
ppm

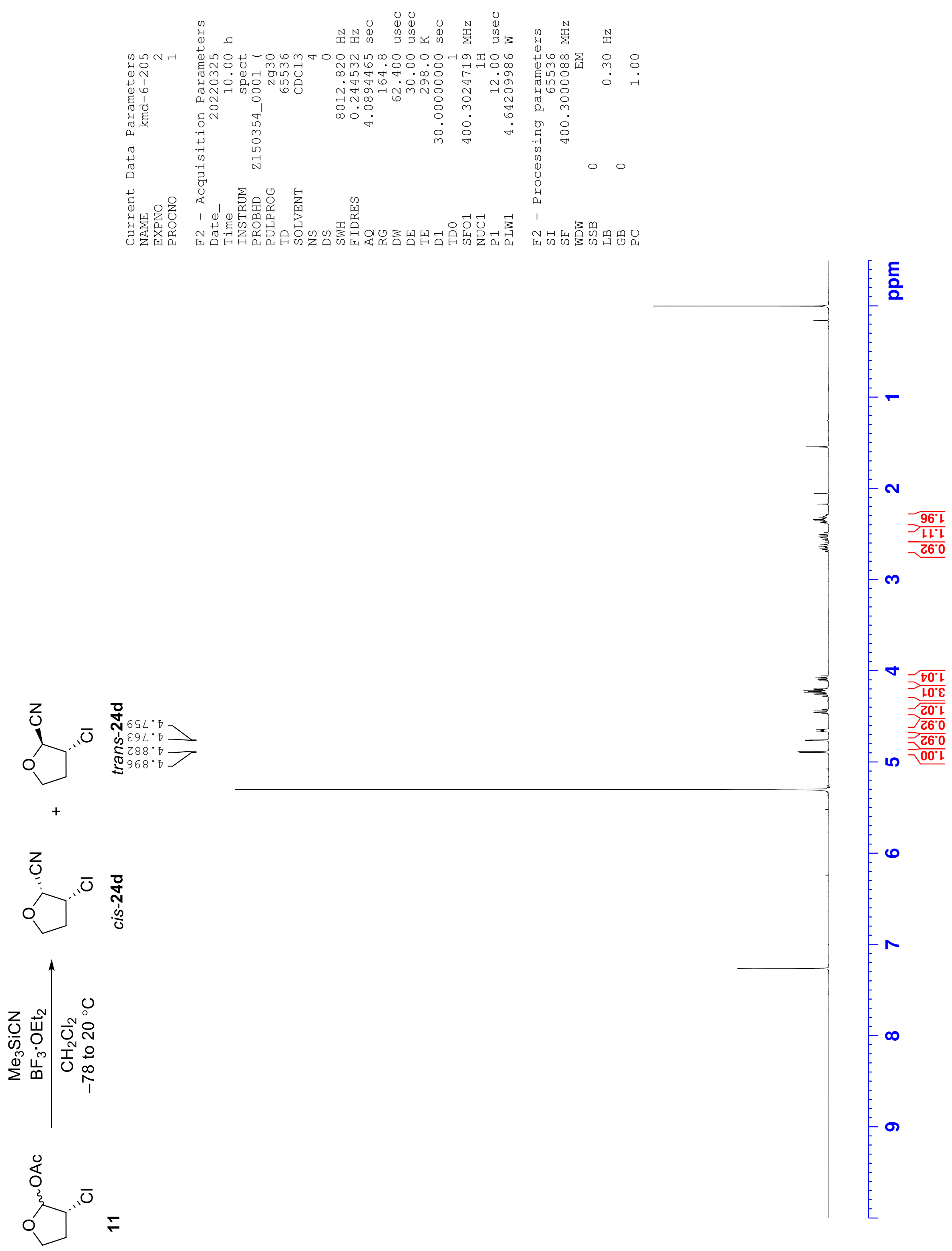

OAc
Cl
11
Me3SiCN
BF3·OEt2
CH2Cl2
−78 to 20 °C
CN
Cl
cis-24d
+
CN
Cl
trans-24d
4.759
4.763
4.882
4.896
Current Data Parameters
NAME kmd-6-205
EXPNO 2
PROCNO 1
F2 - Acquisition Parameters
Date_ 20220325
Time 10.00 h
INSTRUM spect
PROBHD Z150354_0001 (
PULPROG zg30
TD 65536
SOLVENT CDCl3
NS 4
DS 0
SWH 8012.820 Hz
FIDRES 0.244532 Hz
AQ 4.0894465 sec
RG 164.8
DW 62.400 usec
DE 30.00 usec
TE 298.0 K
D1 30.00000000 sec
TD0 1
SFO1 400.3024719 MHz
NUC1 1H
P1 12.00 usec
PLW1 4.64209986 W
F2 - Processing parameters
SI 65536
SF 400.3000088 MHz
WDW EM
SSB 0
LB 0.30 Hz
GB 0
PC 1.00
1.96
1.11
0.92
1.04
3.01
1.02
0.92
0.92
1.00
ppm
1
2
3
4
5
6
7
8
9

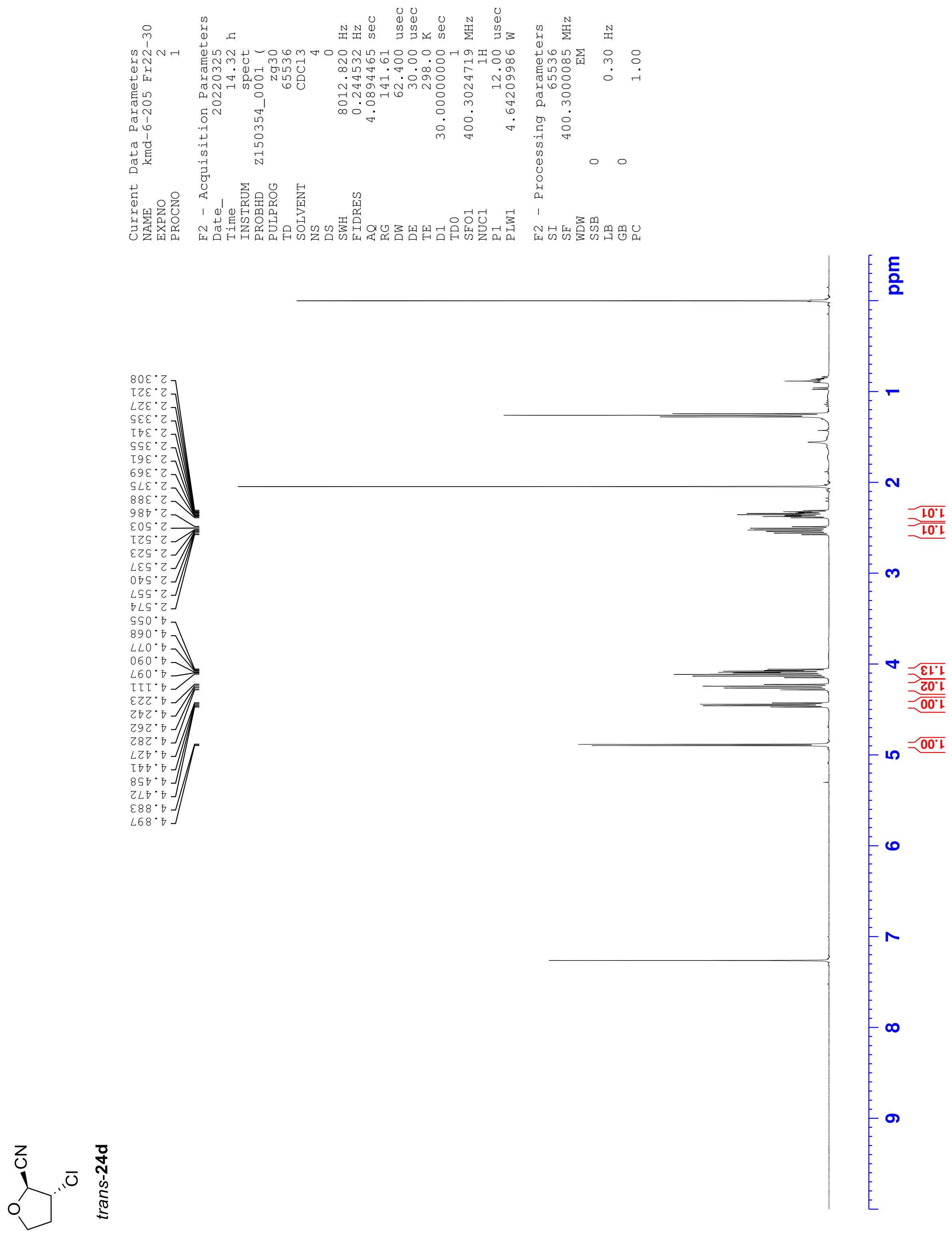
CN
O
Cl
trans-24d
Current Data Parameters
NAME kmd-6-205 Fr22-30
EXPNO 2
PROCNO 1
F2 - Acquisition Parameters
Date_ 20220325
Time 14.32 h
INSTRUM spect
PROBHD Z150354_0001 (
PULPROG zg30
TD 65536
SOLVENT CDCl3
NS 4
DS 0
SWH 8012.820 Hz
FIDRES 0.244532 Hz
AQ 4.0894465 sec
RG 141.61
DW 62.400 usec
DE 30.00 usec
TE 298.0 K
D1 30.00000000 sec
TD0 1
SFO1 400.3024719 MHz
NUC1 1H
P1 12.00 usec
PLW1 4.64209986 W
F2 - Processing parameters
SI 65536
SF 400.3000085 MHz
WDW EM
SSB 0
LB 0.30 Hz
GB 0
PC 1.00
2.308
2.321
2.327
2.335
2.341
2.355
2.361
2.369
2.375
2.388
2.486
2.503
2.521
2.523
2.537
2.540
2.557
2.574
4.055
4.068
4.077
4.090
4.097
4.111
4.223
4.242
4.262
4.282
4.427
4.441
4.458
4.472
4.883
4.897
1.01
1.01
1.13
1.02
1.00
1.00
ppm
1
2
3
4
5
6
7
8
9

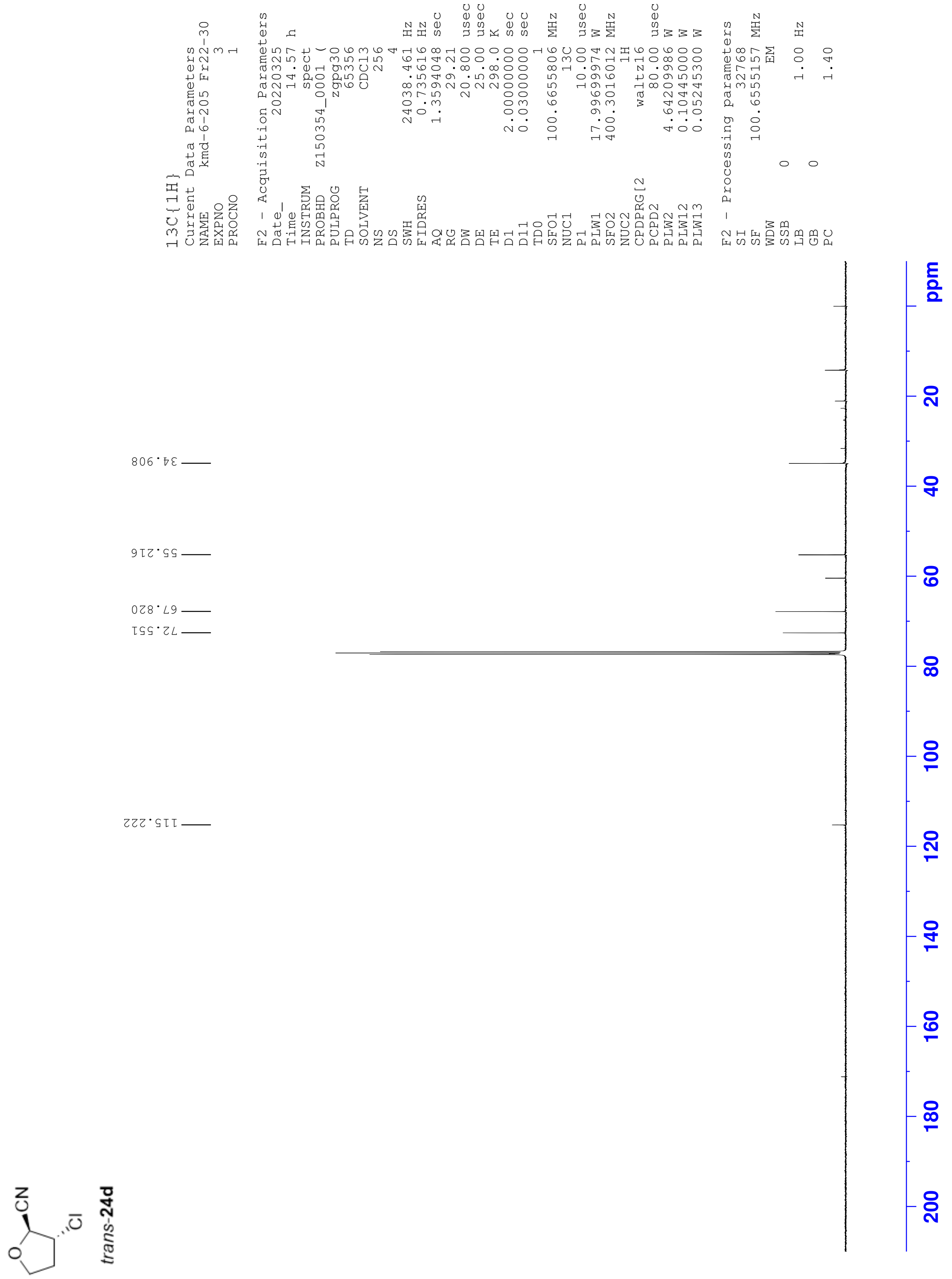
13C{1H}
Current Data Parameters
NAME kmd-6-205 Fr22-30
EXPNO 3
PROCNO 1
F2 - Acquisition Parameters
Date_ 20220325
Time_ 14.57 h
INSTRUM spect
PROBHD Z150354_0001 (
PULPROG zgpg30
TD 65356
SOLVENT CDCl3
NS 256
DS 4
SWH 24038.461 Hz
FIDRES 0.735616 Hz
AQ 1.3594048 sec
RG 29.21
DW 20.800 usec
DE 25.00 usec
TE 298.0 K
D1 2.00000000 sec
D11 0.03000000 sec
TD0 1
SFO1 100.6655806 MHz
NUC1 13C
P1 10.00 usec
PLW1 17.99699974 W
SFO2 400.3016012 MHz
NUC2 1H
CPDPRG[2 waltz16
PCPD2 80.00 usec
PLW2 4.64209986 W
PLW12 0.10445000 W
PLW13 0.05245300 W
F2 - Processing parameters
SI 32768
SF 100.6555157 MHz
WDW EM
SSB 0
LB 1.00 Hz
GB 0
PC 1.40
34.908
55.216
67.820
72.551
115.222
ppm
20
40
60
80
100
120
140
160
180
200
CN
Cl
O
trans-24d

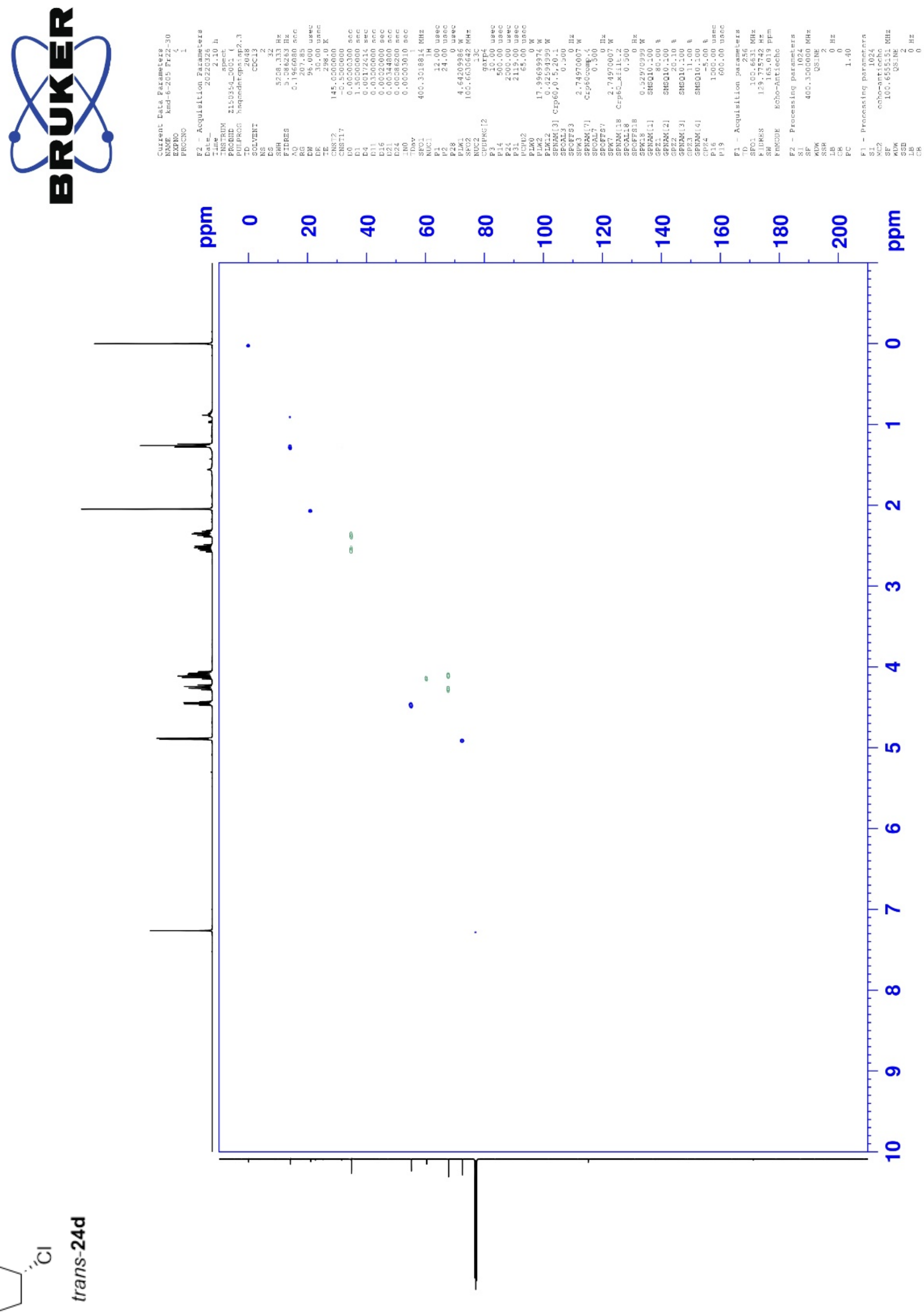
BRUKER
ppm
0
20
40
60
80
100
120
140
160
180
200
ppm
0
1
2
3
4
5
6
7
8
9
10

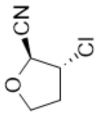

trans-**24d**

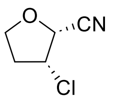

cis-**24d**

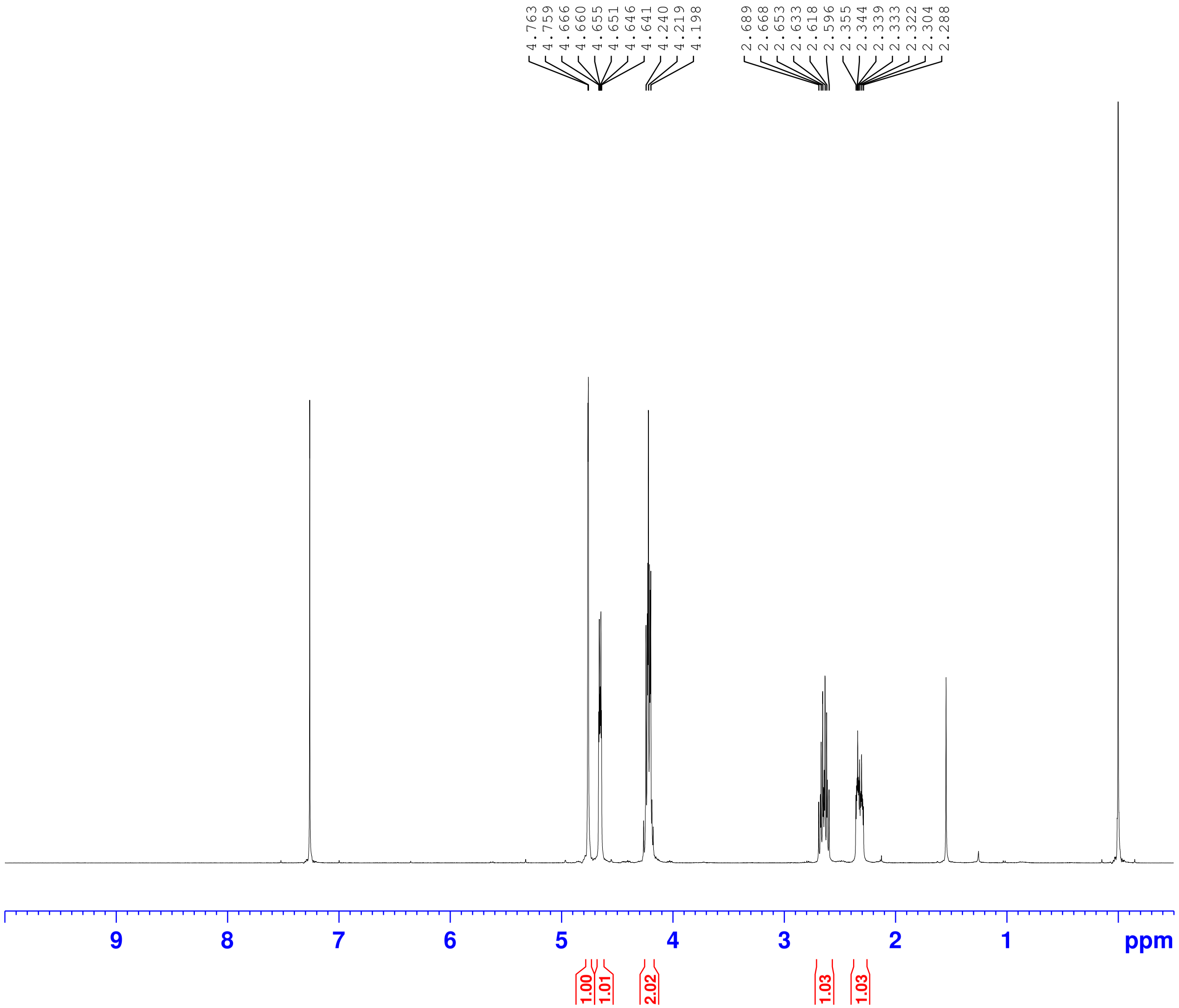


```
Current Data Parameters
NAME       kmd-6-205 Fr10
EXPNO                   2
PROCNO                  1

F2 - Acquisition Parameters
Date_            20220325
Time                18.37 h
INSTRUM             spect
PROBHD    Z150354_0001 (
PULPROG              zg30
TD                  65536
SOLVENT             CDCl3
NS                      4
DS                      0
SWH              8012.820 Hz
FIDRES           0.244532 Hz
AQ              4.0894465 sec
RG                  164.8
DW                 62.400 usec
DE                  30.00 usec
TE                  298.0 K
D1            30.00000000 sec
TD0                     1
SFO1          400.3024719 MHz
NUC1                   1H
P1                  12.00 usec
PLW1           4.64209986 W

F2 - Processing parameters
SI                  65536
SF            400.3000090 MHz
WDW                    EM
SSB       0
LB                   0.30 Hz
GB        0
PC                   1.00
```

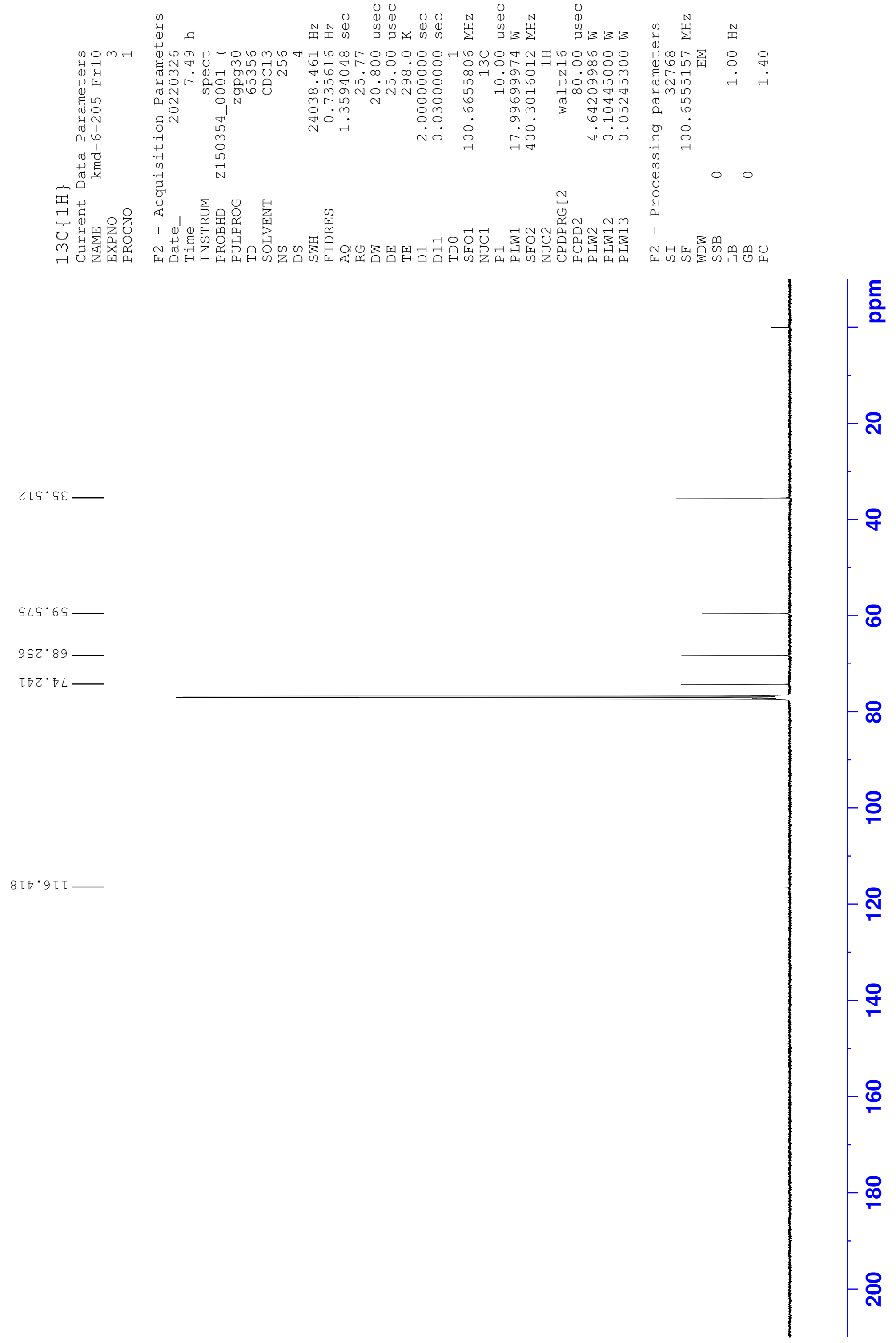
13C{1H}
Current Data Parameters
NAME kmd-6-205 Fr10
EXPNO 3
PROCNO 1
F2 - Acquisition Parameters
Date_ 20220326
Time_ 7.49 h
INSTRUM spect
PROBHD Z150354_0001 (
PULPROG zgpg30
TD 65356
SOLVENT CDCl3
NS 256
DS 4
SWH 24038.461 Hz
FIDRES 0.735616 Hz
AQ 1.3594048 sec
RG 25.77
DW 20.800 usec
DE 25.00 usec
TE 298.0 K
D1 2.00000000 sec
D11 0.03000000 sec
TD0 1
SFO1 100.6655806 MHz
NUC1 13C
P1 10.00 usec
PLW1 17.99699974 W
SFO2 400.3016012 MHz
NUC2 1H
CPDPRG[2 waltz16
PCPD2 80.00 usec
PLW2 4.64209986 W
PLW12 0.10445000 W
PLW13 0.05245300 W
F2 - Processing parameters
SI 32768
SF 100.6555157 MHz
WDW EM
SSB 0
LB 1.00 Hz
GB 0
PC 1.40
35.512
59.575
68.256
74.241
116.418
ppm
20
40
60
80
100
120
140
160
180
200

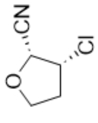
O
CN
Cl

cis-**24d**

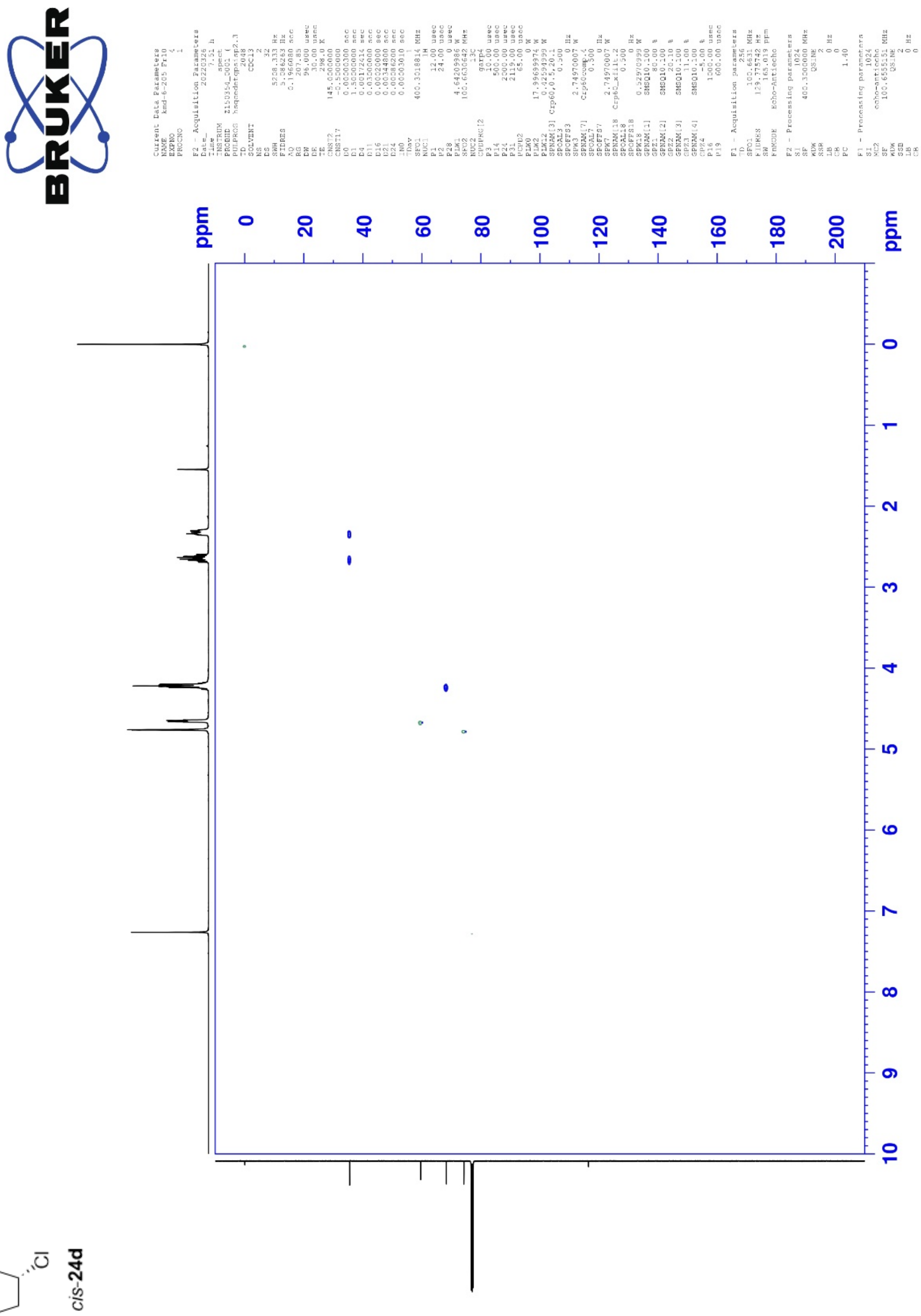

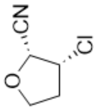

cis-**24d**

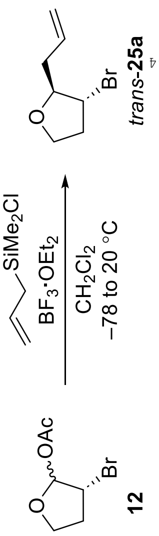
OAc
O
Br
12
SiMe2Cl
BF3·OEt2
CH2Cl2
−78 to 20 °C
O
Br
trans-25a

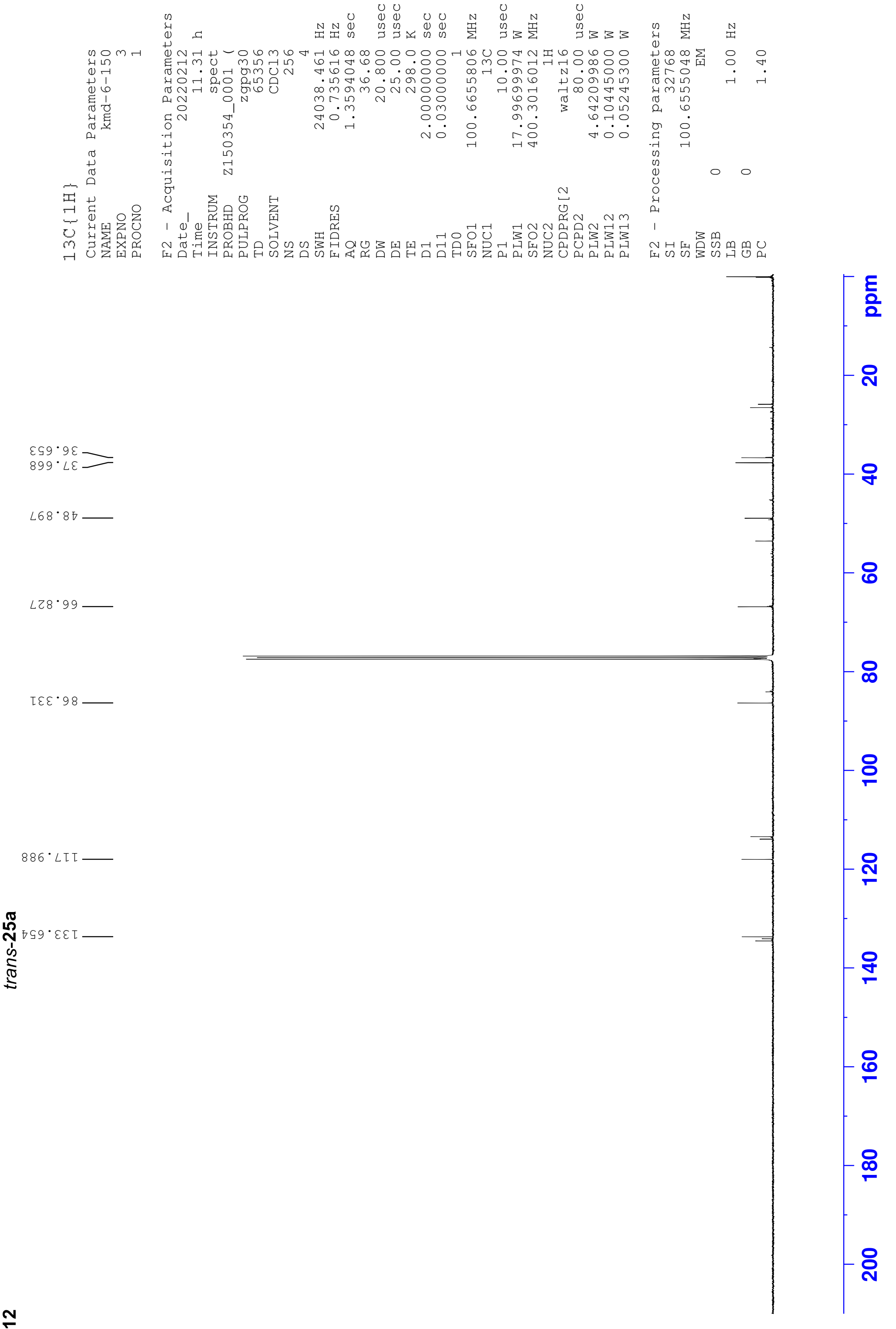
13C{1H}
Current Data Parameters
NAME kmd-6-150
EXPNO 3
PROCNO 1
F2 - Acquisition Parameters
Date_ 20220212
Time_ 11.31 h
INSTRUM spect
PROBHD Z150354_0001 (
PULPROG zgpg30
TD 65356
SOLVENT CDCl3
NS 256
DS 4
SWH 24038.461 Hz
FIDRES 0.735616 Hz
AQ 1.3594048 sec
RG 36.68
DW 20.800 usec
DE 25.00 usec
TE 298.0 K
D1 2.00000000 sec
D11 0.03000000 sec
TD0 1
SFO1 100.6655806 MHz
NUC1 13C
P1 10.00 usec
PLW1 17.99699974 W
SFO2 400.3016012 MHz
NUC2 1H
CPDPRG[2 waltz16
PCPD2 80.00 usec
PLW2 4.64209986 W
PLW12 0.10445000 W
PLW13 0.05245300 W
F2 - Processing parameters
SI 32768
SF 100.6555048 MHz
WDW EM
SSB 0
LB 1.00 Hz
GB 0
PC 1.40
36.653
37.668
48.897
66.827
86.331
117.988
133.654
ppm
20
40
60
80
100
120
140
160
180
200

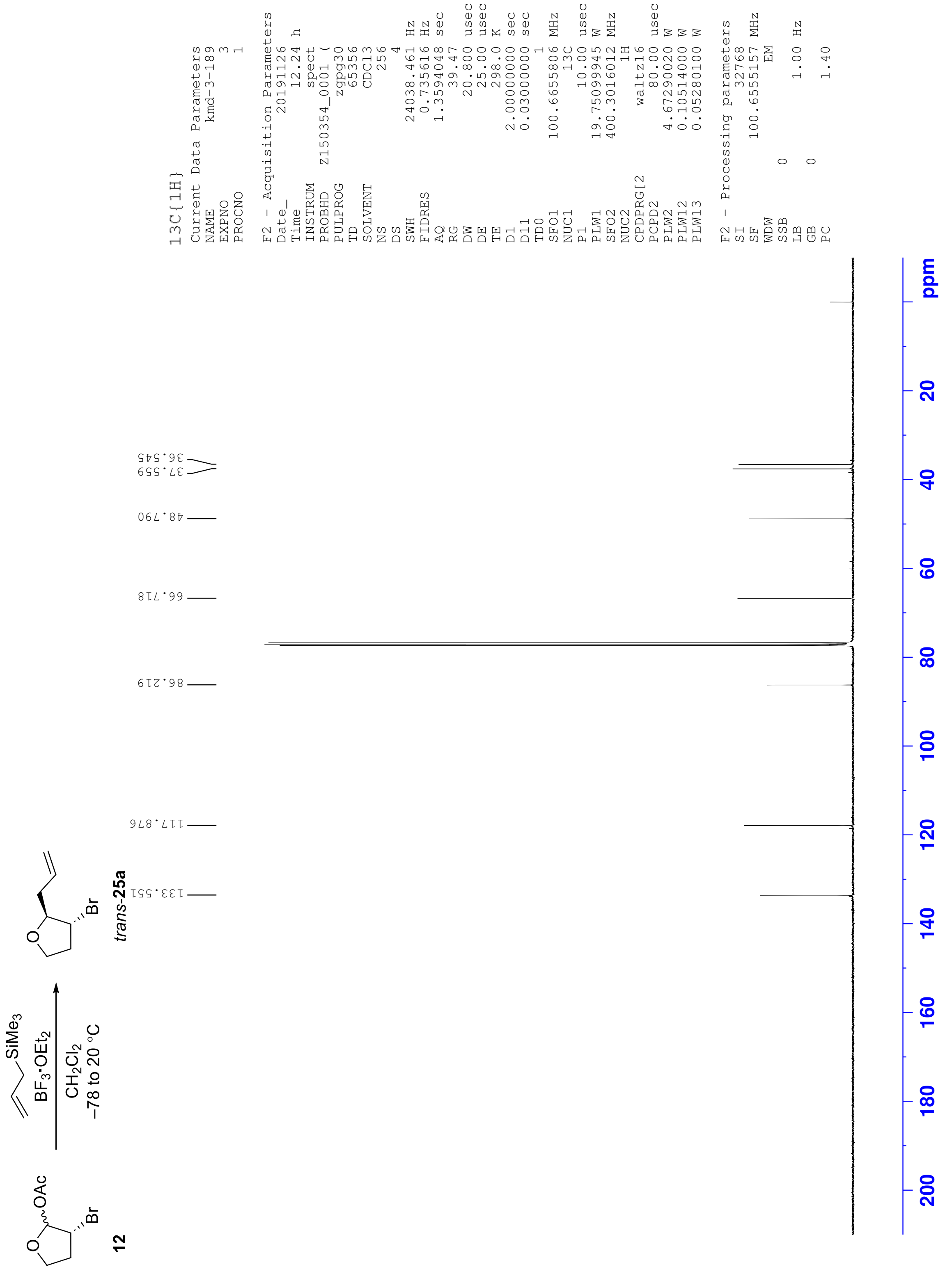
12
BF3•OEt2
CH2Cl2
−78 to 20 °C
trans-25a
13C{1H}
Current Data Parameters
NAME kmd-3-189
EXPNO 3
PROCNO 1
F2 - Acquisition Parameters
Date_ 20191126
Time_ 12.24 h
INSTRUM spect
PROBHD Z150354_0001 (
PULPROG zgpg30
TD 65356
SOLVENT CDCl3
NS 256
DS 4
SWH 24038.461 Hz
FIDRES 0.735616 Hz
AQ 1.3594048 sec
RG 39.47
DW 20.800 usec
DE 25.00 usec
TE 298.0 K
D1 2.00000000 sec
D11 0.03000000 sec
TD0 1
SFO1 100.6655806 MHz
NUC1 13C
P1 10.00 usec
PLW1 19.75099945 W
SFO2 400.3016012 MHz
NUC2 1H
CPDPRG[2 waltz16
PCPD2 80.00 usec
PLW2 4.67290020 W
PLW12 0.10514000 W
PLW13 0.05280100 W
F2 - Processing parameters
SI 32768
SF 100.6555157 MHz
WDW EM
SSB 0
LB 1.00 Hz
GB 0
PC 1.40
36.545
37.559
48.790
66.718
86.219
117.876
133.551
ppm

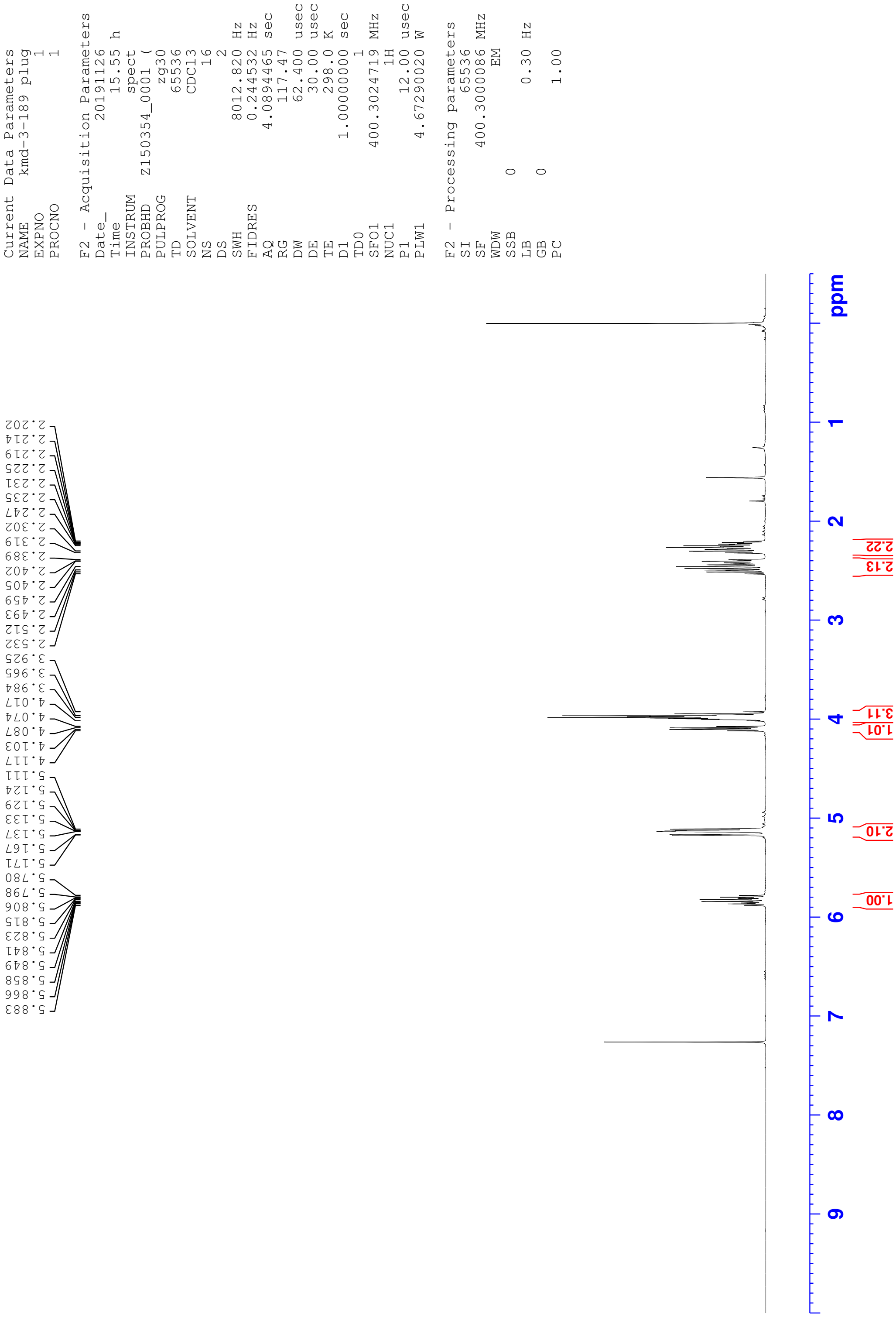
Current Data Parameters
NAME kmd-3-189 plug
EXPNO 1
PROCNO 1
F2 - Acquisition Parameters
Date_ 20191126
Time 15.55 h
INSTRUM spect
PROBHD Z150354_0001 (
PULPROG zg30
TD 65536
SOLVENT CDCl3
NS 16
DS 2
SWH 8012.820 Hz
FIDRES 0.244532 Hz
AQ 4.0894465 sec
RG 117.47
DW 62.400 usec
DE 30.00 usec
TE 298.0 K
D1 1.00000000 sec
TD0 1
SFO1 400.3024719 MHz
NUC1 1H
P1 12.00 usec
PLW1 4.67290020 W
F2 - Processing parameters
SI 65536
SF 400.3000086 MHz
WDW EM
SSB 0
LB 0.30 Hz
GB 0
PC 1.00
5.883
5.866
5.858
5.849
5.841
5.823
5.815
5.806
5.798
5.780
5.171
5.167
5.137
5.133
5.129
5.124
5.111
4.117
4.103
4.087
4.074
4.017
3.984
3.965
3.925
2.532
2.512
2.493
2.459
2.405
2.402
2.389
2.319
2.302
2.247
2.235
2.231
2.225
2.219
2.214
2.202
2.22
2.13
3.11
1.01
2.10
1.00
ppm
1
2
3
4
5
6
7
8
9

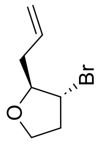
O
Br

trans-**25a**

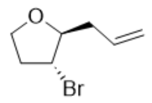

trans-**25a**

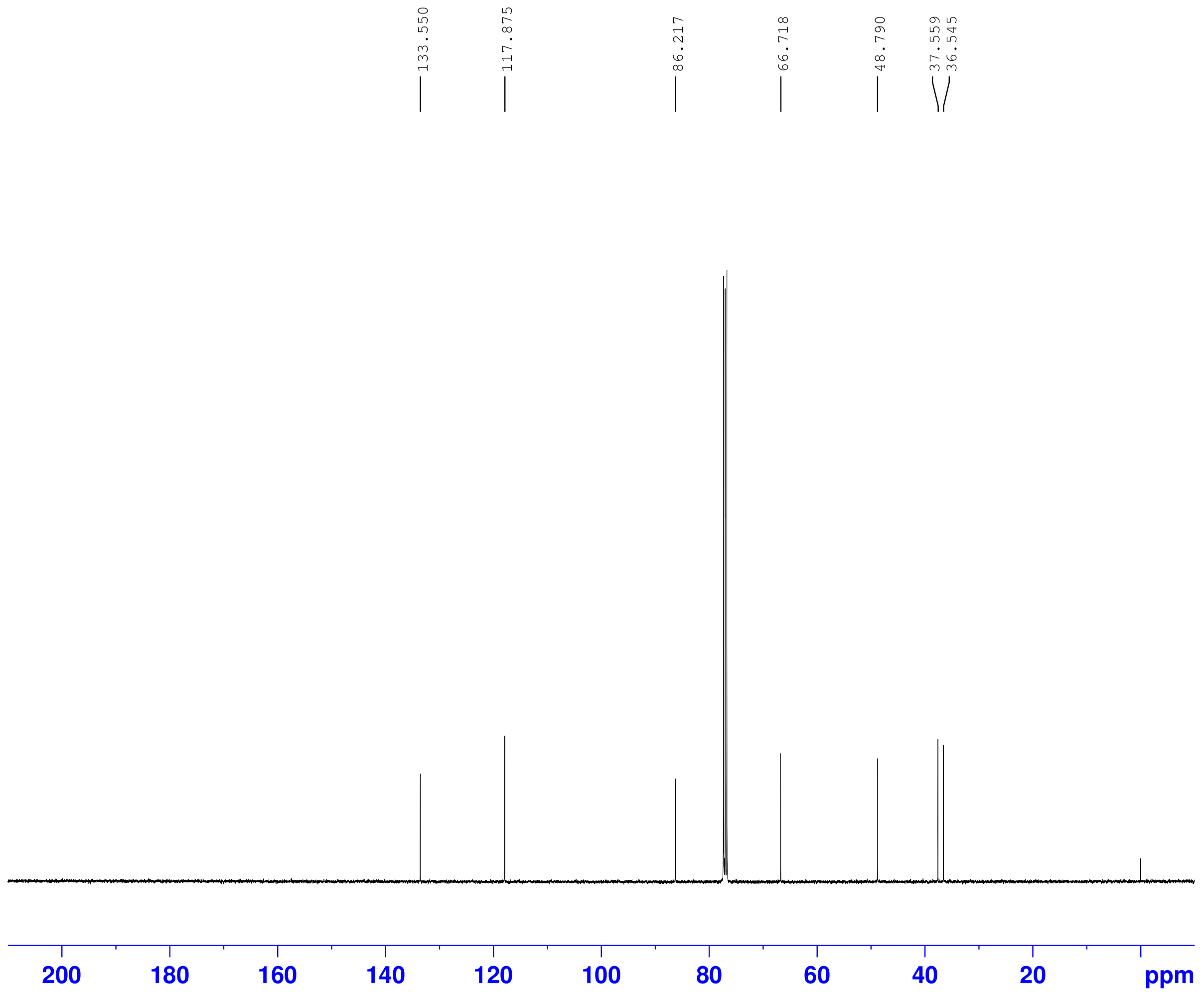


```
13C{1H}
Current Data Parameters
NAME      kmd-3-189 plug
EXPNO                  2
PROCNO                 1

F2 - Acquisition Parameters
Date_           20191126
Time               17.28 h
INSTRUM            spect
PROBHD    Z150354_0001 (
PULPROG          zgpg30
TD                65356
SOLVENT           CDCl3
NS                  128
DS                    4
SWH           24038.461 Hz
FIDRES         0.735616 Hz
AQ            1.3594048 sec
RG                25.77
DW               20.800 usec
DE                25.00 usec
TE                298.0 K
D1           2.00000000 sec
D11          0.03000000 sec
TD0                   1
SFO1        100.6655806 MHz
NUC1                13C
P1                10.00 usec
PLW1        19.75099945 W
SFO2        400.3016012 MHz
NUC2                 1H
CPDPRG[2        waltz16
PCPD2             80.00 usec
PLW2         4.67290020 W
PLW12        0.10514000 W
PLW13        0.05280100 W

F2 - Processing parameters
SI                32768
SF          100.6555158 MHz
WDW                  EM
SSB      0
LB                 1.00 Hz
GB       0
PC                 1.40
```

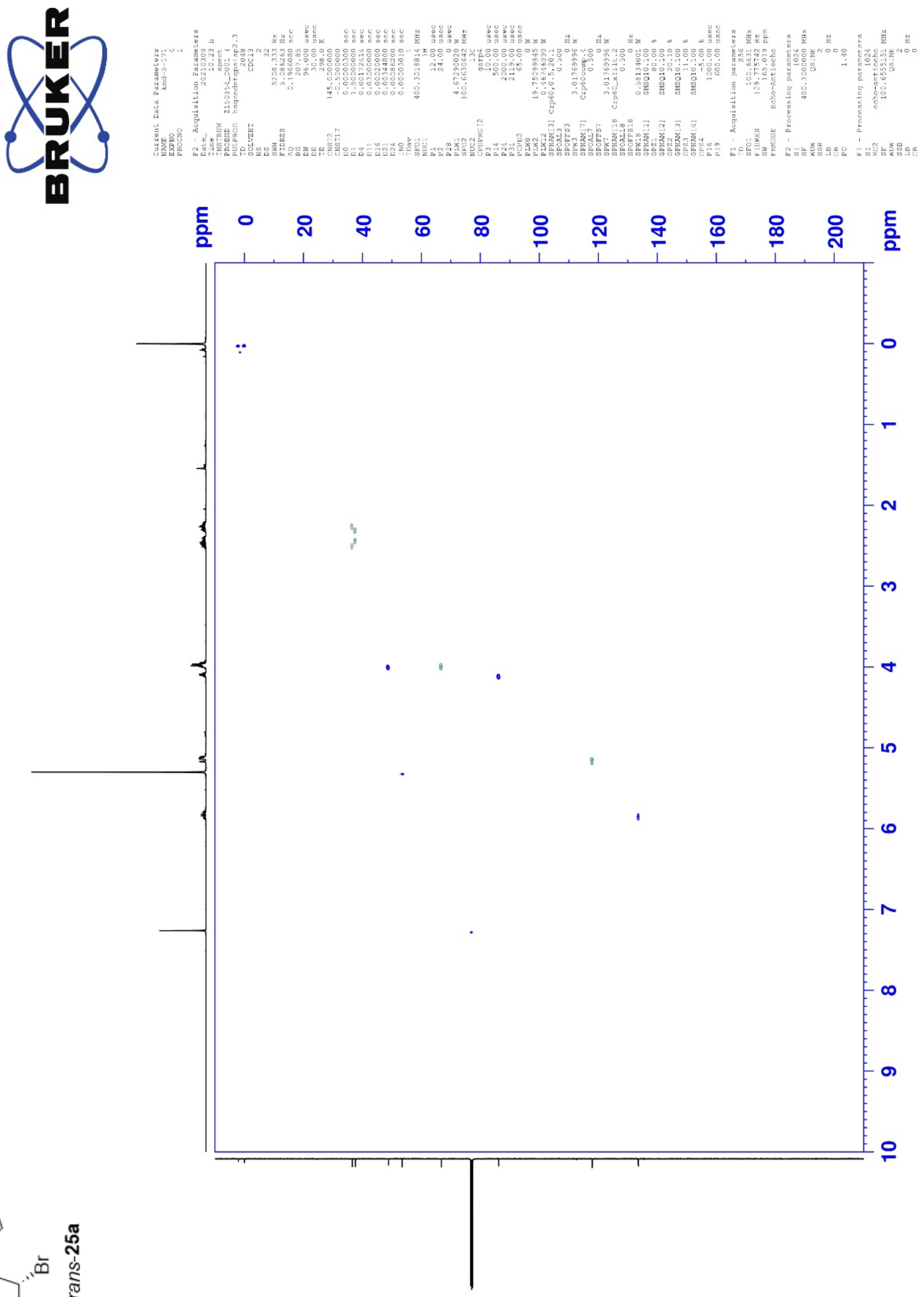
BRUKER
ppm
0
20
40
60
80
100
120
140
160
180
200
ppm
0
1
2
3
4
5
6
7
8
9
10

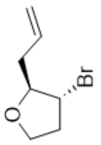

trans-**25a**

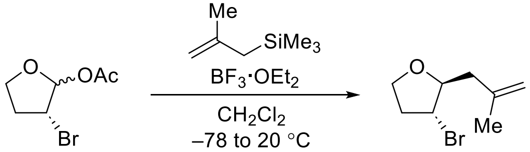

12 → *trans*-**25b**

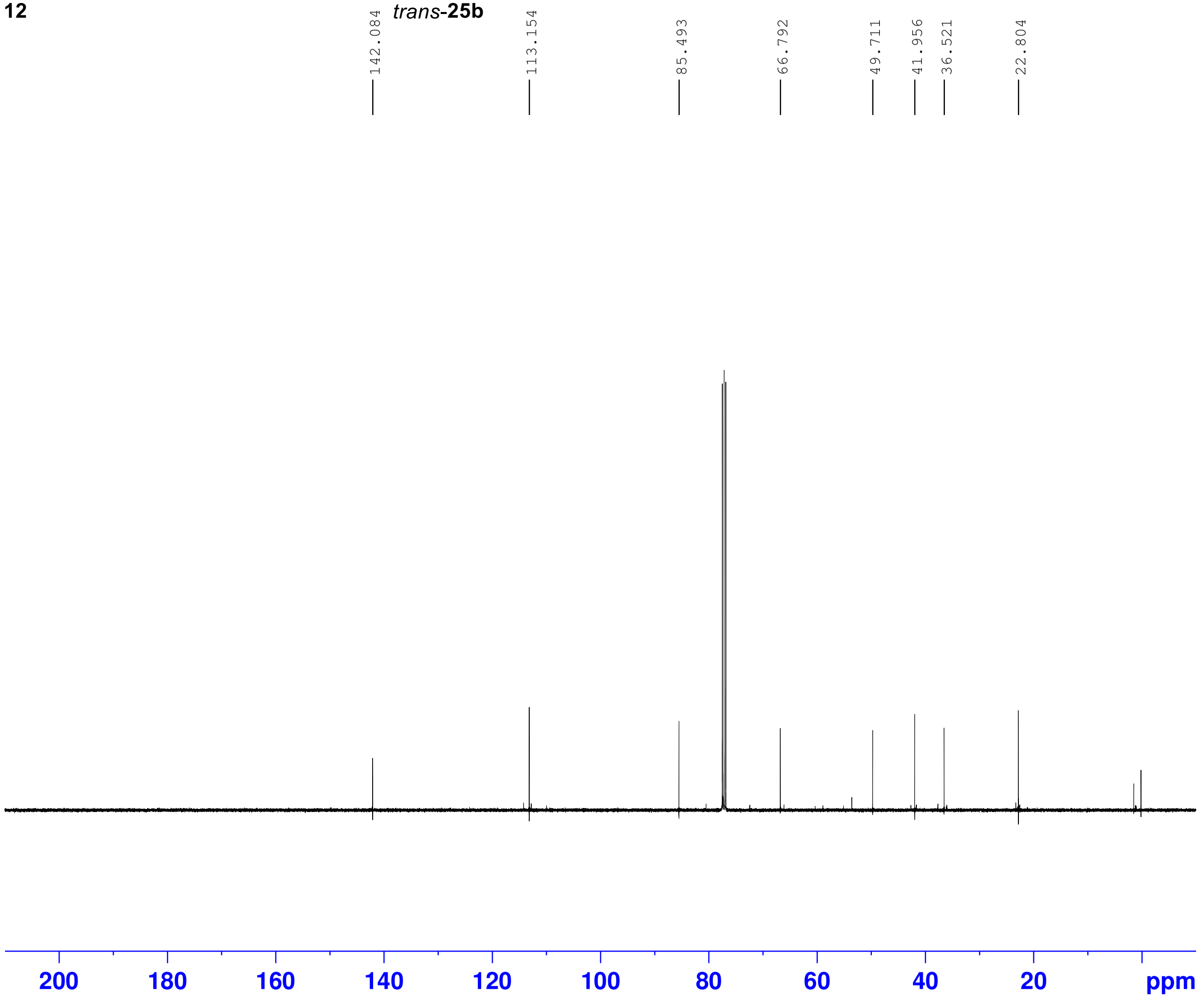


```
13C{1H}
Current Data Parameters
NAME            kmd-3-191
EXPNO                   3
PROCNO                  1

F2 - Acquisition Parameters
Date_            20191126
Time                11.56 h
INSTRUM             spect
PROBHD   Z150354_0001 (
PULPROG           zgpg30
TD                 65356
SOLVENT            CDCl3
NS                   256
DS                     4
SWH            24038.461 Hz
FIDRES          0.735616 Hz
AQ             1.3594048 sec
RG                 29.21
DW                20.800 usec
DE                 25.00 usec
TE                 298.0 K
D1            2.00000000 sec
D11           0.03000000 sec
TD0                    1
SFO1         100.6655806 MHz
NUC1                 13C
P1                 10.00 usec
PLW1         19.75099945 W
SFO2         400.3016012 MHz
NUC2                  1H
CPDPRG[2         waltz16
PCPD2              80.00 usec
PLW2          4.67290020 W
PLW12         0.10514000 W
PLW13         0.05280100 W

F2 - Processing parameters
SI                131072
SF          100.6555014 MHz
WDW                   EM
SSB      0
LB       0 Hz
GB       0
PC                  1.40
```

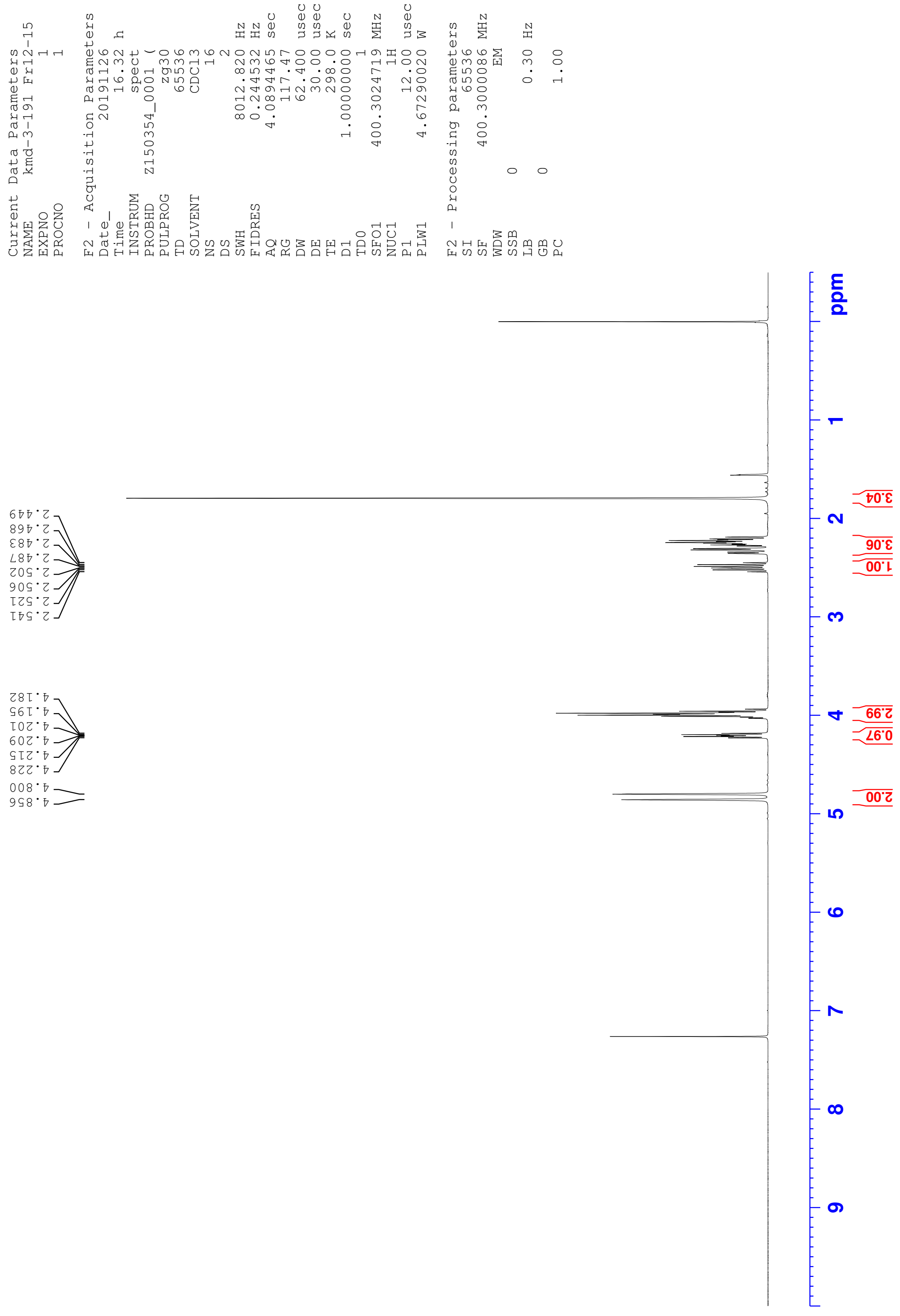
Current Data Parameters
NAME kmd-3-191 Fr12-15
EXPNO 1
PROCNO 1
F2 - Acquisition Parameters
Date_ 20191126
Time 16.32 h
INSTRUM spect
PROBHD Z150354_0001 (
PULPROG zg30
TD 65536
SOLVENT CDCl3
NS 16
DS 2
SWH 8012.820 Hz
FIDRES 0.244532 Hz
AQ 4.0894465 sec
RG 117.47
DW 62.400 usec
DE 30.00 usec
TE 298.0 K
D1 1.00000000 sec
TD0 1
SFO1 400.3024719 MHz
NUC1 1H
P1 12.00 usec
PLW1 4.67290020 W
F2 - Processing parameters
SI 65536
SF 400.3000086 MHz
WDW EM
SSB 0
LB 0.30 Hz
GB 0
PC 1.00
2.449
2.468
2.483
2.487
2.502
2.506
2.521
2.541
4.182
4.195
4.201
4.209
4.215
4.228
4.800
4.856
3.04
3.06
1.00
2.99
0.97
2.00
ppm

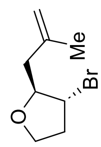
O
Br
Me
trans-**25b**

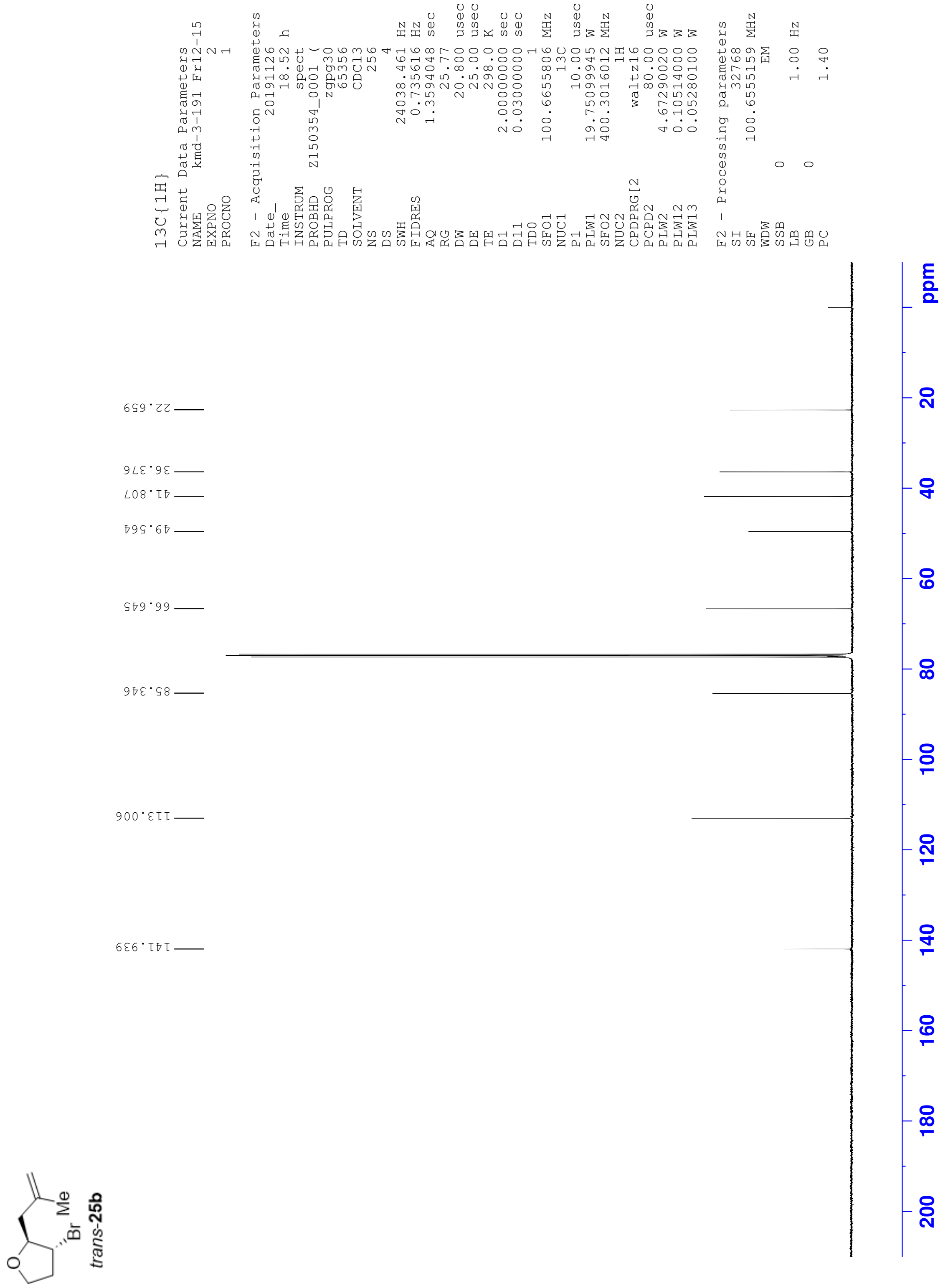
13C{1H}
Current Data Parameters
NAME kmd-3-191 Fr12-15
EXPNO 2
PROCNO 1
F2 - Acquisition Parameters
Date_ 20191126
Time_ 18.52 h
INSTRUM spect
PROBHD Z150354_0001 (
PULPROG zgpg30
TD 65356
SOLVENT CDCl3
NS 256
DS 4
SWH 24038.461 Hz
FIDRES 0.735616 Hz
AQ 1.3594048 sec
RG 25.77
DW 20.800 usec
DE 25.00 usec
TE 298.0 K
D1 2.00000000 sec
D11 0.03000000 sec
TD0 1
SFO1 100.6655806 MHz
NUC1 13C
P1 10.00 usec
PLW1 19.75099945 W
SFO2 400.3016012 MHz
NUC2 1H
CPDPRG[2 waltz16
PCPD2 80.00 usec
PLW2 4.67290020 W
PLW12 0.10514000 W
PLW13 0.05280100 W
F2 - Processing parameters
SI 32768
SF 100.6555159 MHz
WDW EM
SSB 0
LB 1.00 Hz
GB 0
PC 1.40
22.659
36.376
41.807
49.564
66.645
85.346
113.006
141.939
ppm
20
40
60
80
100
120
140
160
180
200
O
Br
Me
trans-**25b**

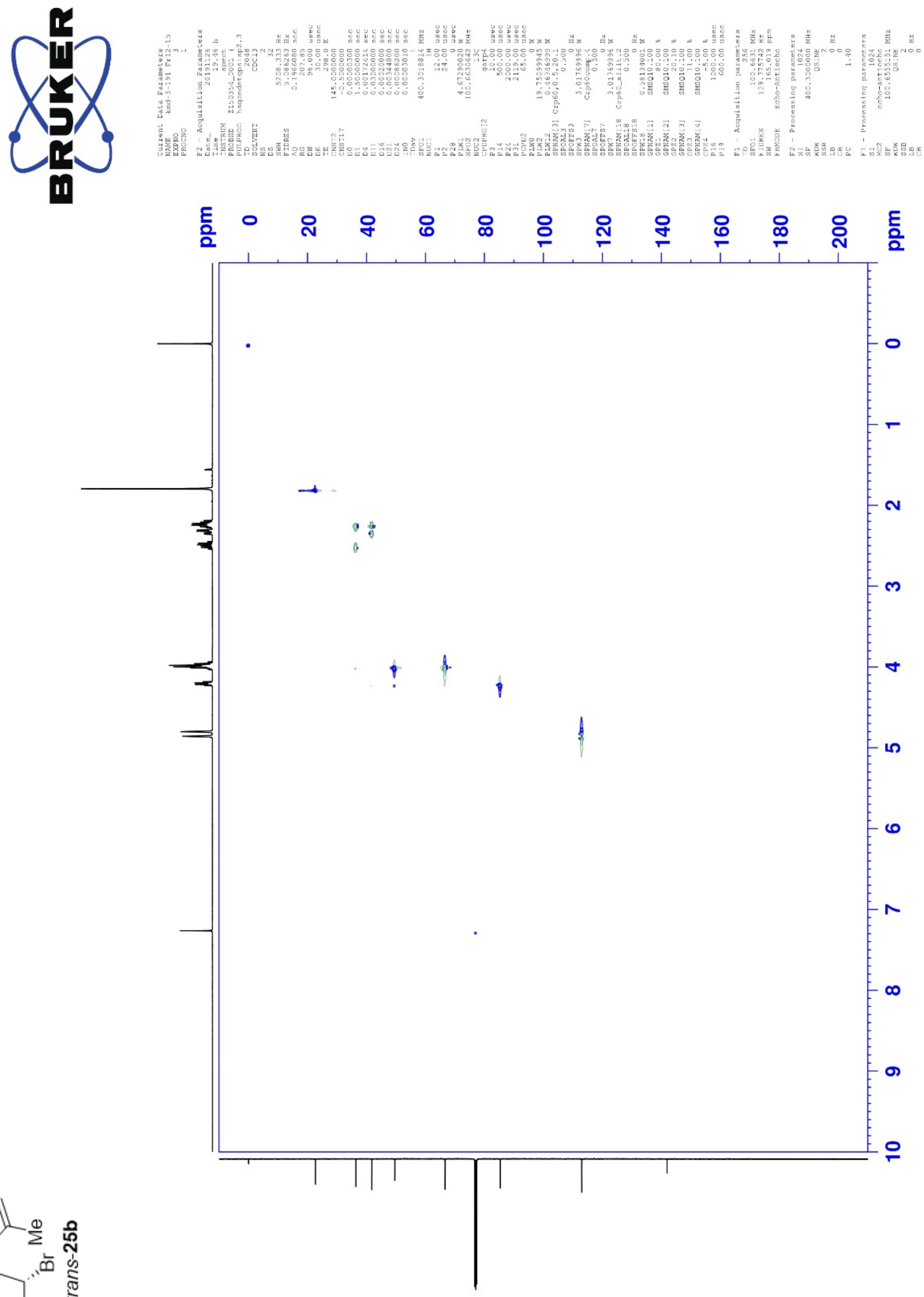

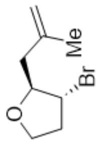

trans-**25b**

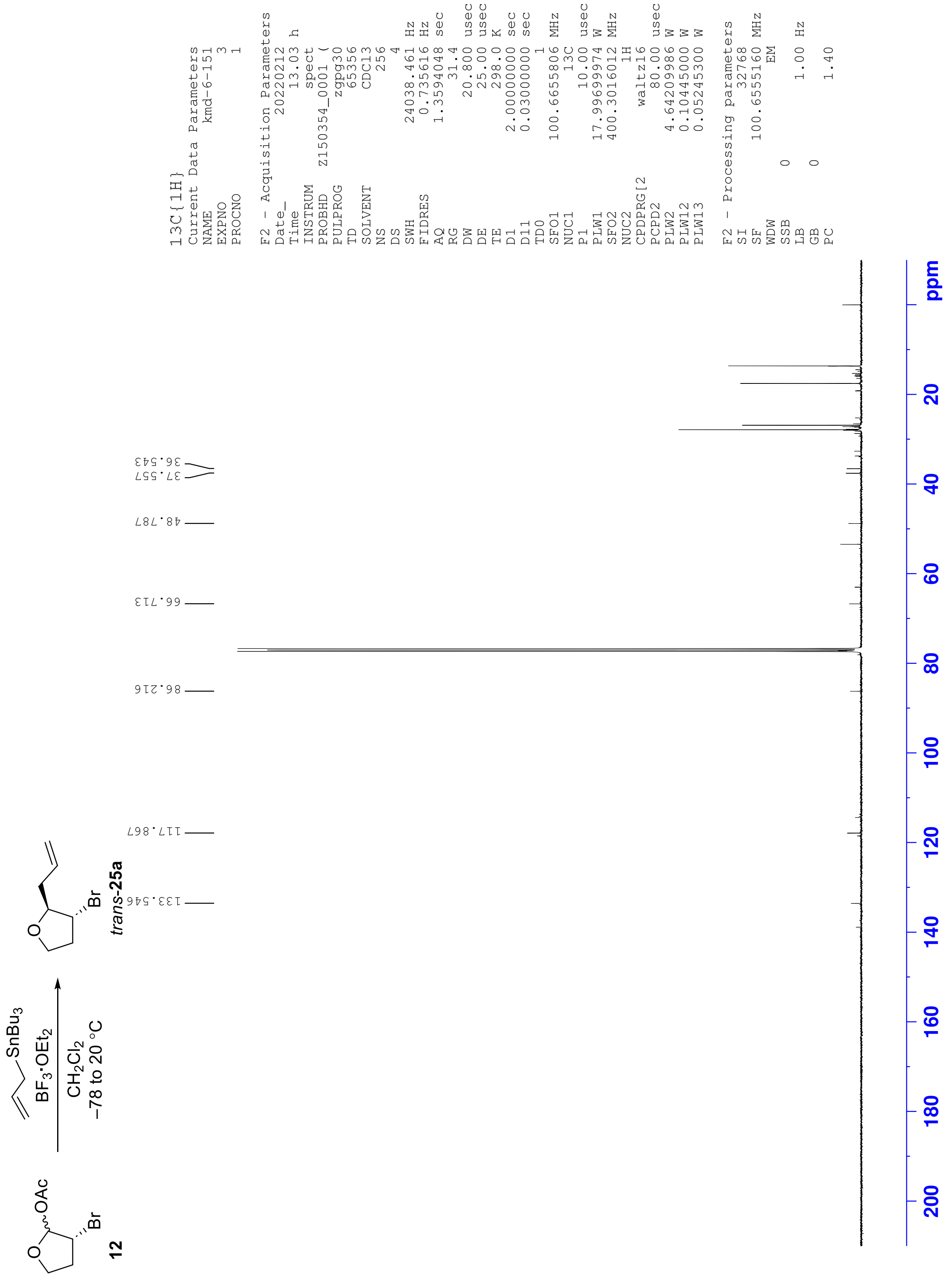

OAc
Br
12
SnBu3
BF3·OEt2
CH2Cl2
−78 to 20 °C
O
Br
trans-25a
133.546
117.867
86.216
66.713
48.787
37.557
36.543
13C{1H}
Current Data Parameters
NAME kmd-6-151
EXPNO 3
PROCNO 1
F2 - Acquisition Parameters
Date_ 20220212
Time_ 13.03 h
INSTRUM spect
PROBHD Z150354_0001 (
PULPROG zgpg30
TD 65356
SOLVENT CDCl3
NS 256
DS 4
SWH 24038.461 Hz
FIDRES 0.735616 Hz
AQ 1.3594048 sec
RG 31.4
DW 20.800 usec
DE 25.00 usec
TE 298.0 K
D1 2.00000000 sec
D11 0.03000000 sec
TD0 1
SFO1 100.6655806 MHz
NUC1 13C
P1 10.00 usec
PLW1 17.99699974 W
SFO2 400.3016012 MHz
NUC2 1H
CPDPRG[2 waltz16
PCPD2 80.00 usec
PLW2 4.64209986 W
PLW12 0.10445000 W
PLW13 0.05245300 W
F2 - Processing parameters
SI 32768
SF 100.6555160 MHz
WDW EM
SSB 0
LB 1.00 Hz
GB 0
PC 1.40
200
180
160
140
120
100
80
60
40
20
ppm

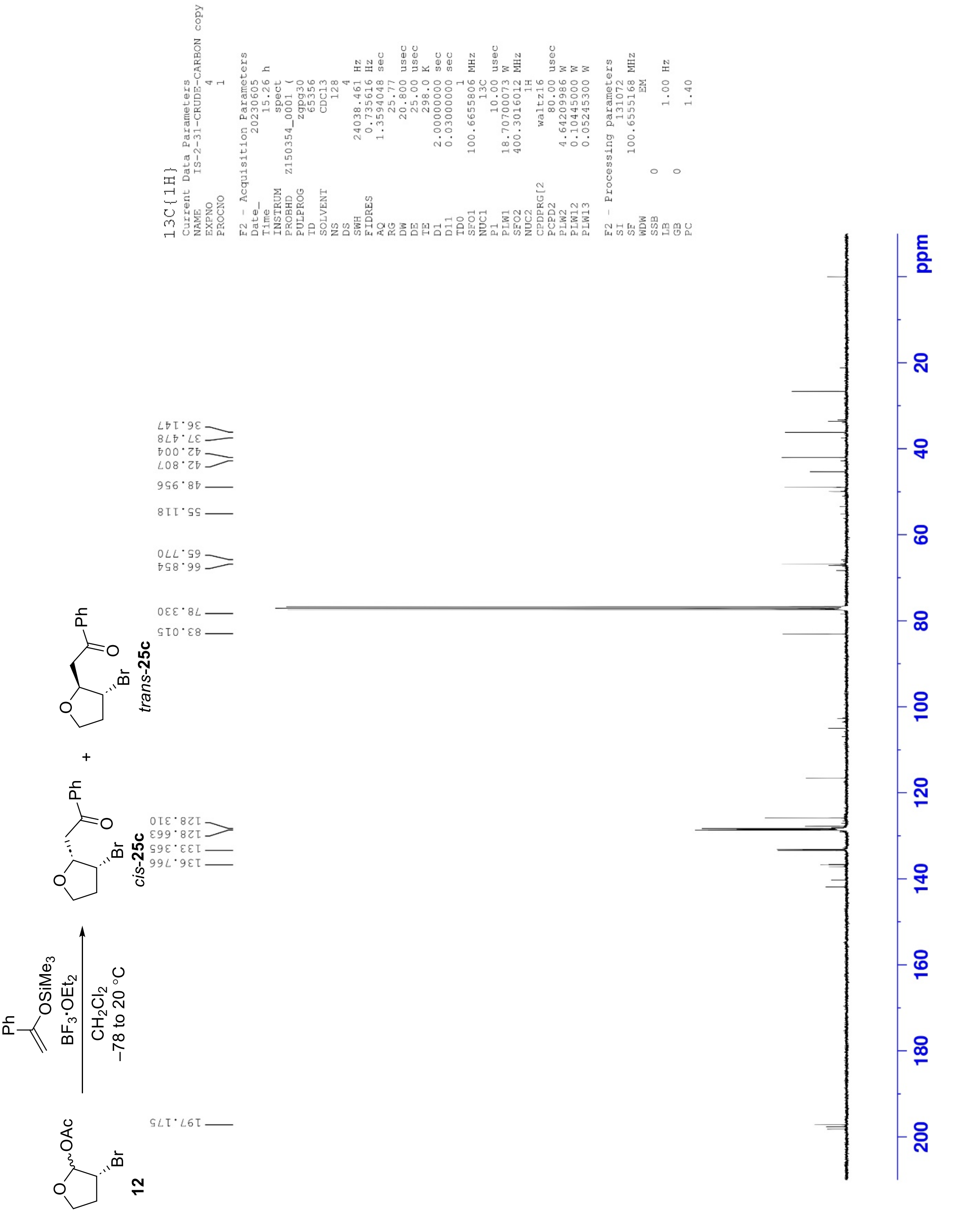
12
OAc
Br
Ph
OSiMe3
BF3·OEt2
CH2Cl2
−78 to 20 °C
cis-25c
trans-25c
Ph
O
Br
+
13C{1H}
Current Data Parameters
NAME IS-2-31-CRUDE-CARBON copy
EXPNO 4
PROCNO 1
F2 - Acquisition Parameters
Date_ 20230605
Time 15.26 h
INSTRUM spect
PROBHD Z150354_0001 (
PULPROG zgpg30
TD 65536
SOLVENT CDCl3
NS 128
DS 4
SWH 24038.461 Hz
FIDRES 0.735616 Hz
AQ 1.3594048 sec
RG 25.77
DW 20.800 usec
DE 25.00 usec
TE 298.0 K
D1 2.00000000 sec
D11 0.03000000 sec
TD0 1
SFO1 100.6655806 MHz
NUC1 13C
P1 10.00 usec
PLW1 18.70700073 W
SFO2 400.3016012 MHz
NUC2 1H
CPDPRG[2 waltz16
PCPD2 80.00 usec
PLW2 4.64209986 W
PLW12 0.10445000 W
PLW13 0.05245300 W
F2 - Processing parameters
SI 131072
SF 100.6555168 MHz
WDW EM
SSB 0
LB 1.00 Hz
GB 0
PC 1.40
36.147
37.478
42.004
42.807
48.956
55.118
65.770
66.854
78.330
83.015
128.310
128.663
133.365
136.766
197.175
ppm
20
40
60
80
100
120
140
160
180
200

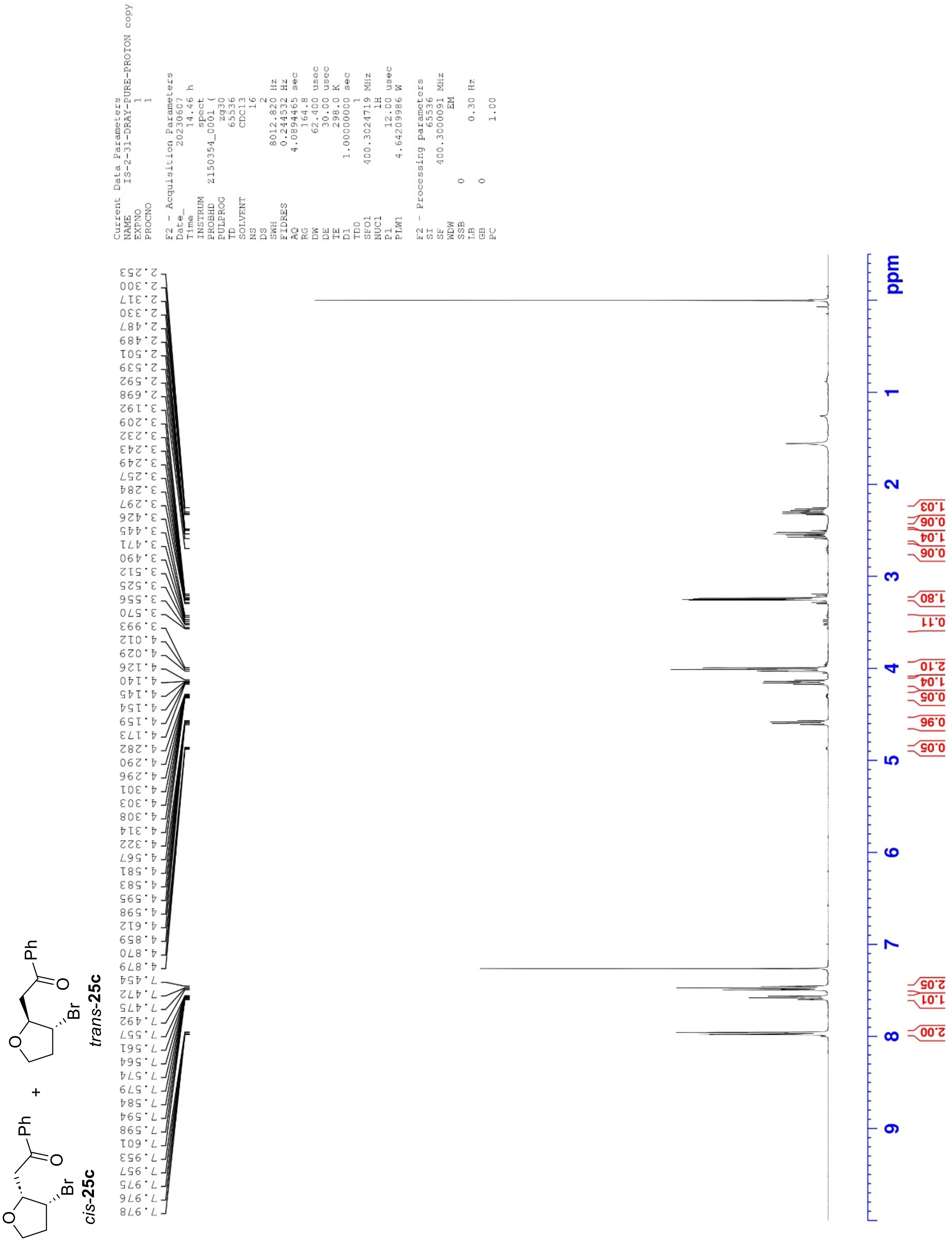
cis-25c
trans-25c
Current Data Parameters
NAME IS-2-31-DRAY-PURE-PROTON copy
EXPNO 1
PROCNO 1
F2 - Acquisition Parameters
Date_ 20230607
Time 14.46 h
INSTRUM spect
PROBHD Z150354_0001 (
PULPROG zg30
TD 65536
SOLVENT CDCl3
NS 16
DS 2
SWH 8012.820 Hz
FIDRES 0.244532 Hz
AQ 4.0894465 sec
RG 164.8
DW 62.400 usec
DE 30.00 usec
TE 298.0 K
D1 1.00000000 sec
TD0 1
SFO1 400.3024719 MHz
NUC1 1H
P1 12.00 usec
PLW1 4.64209986 W
F2 - Processing parameters
SI 65536
SF 400.3000091 MHz
WDW EM
SSB 0
LB 0.30 Hz
GB 0
PC 1.00
ppm

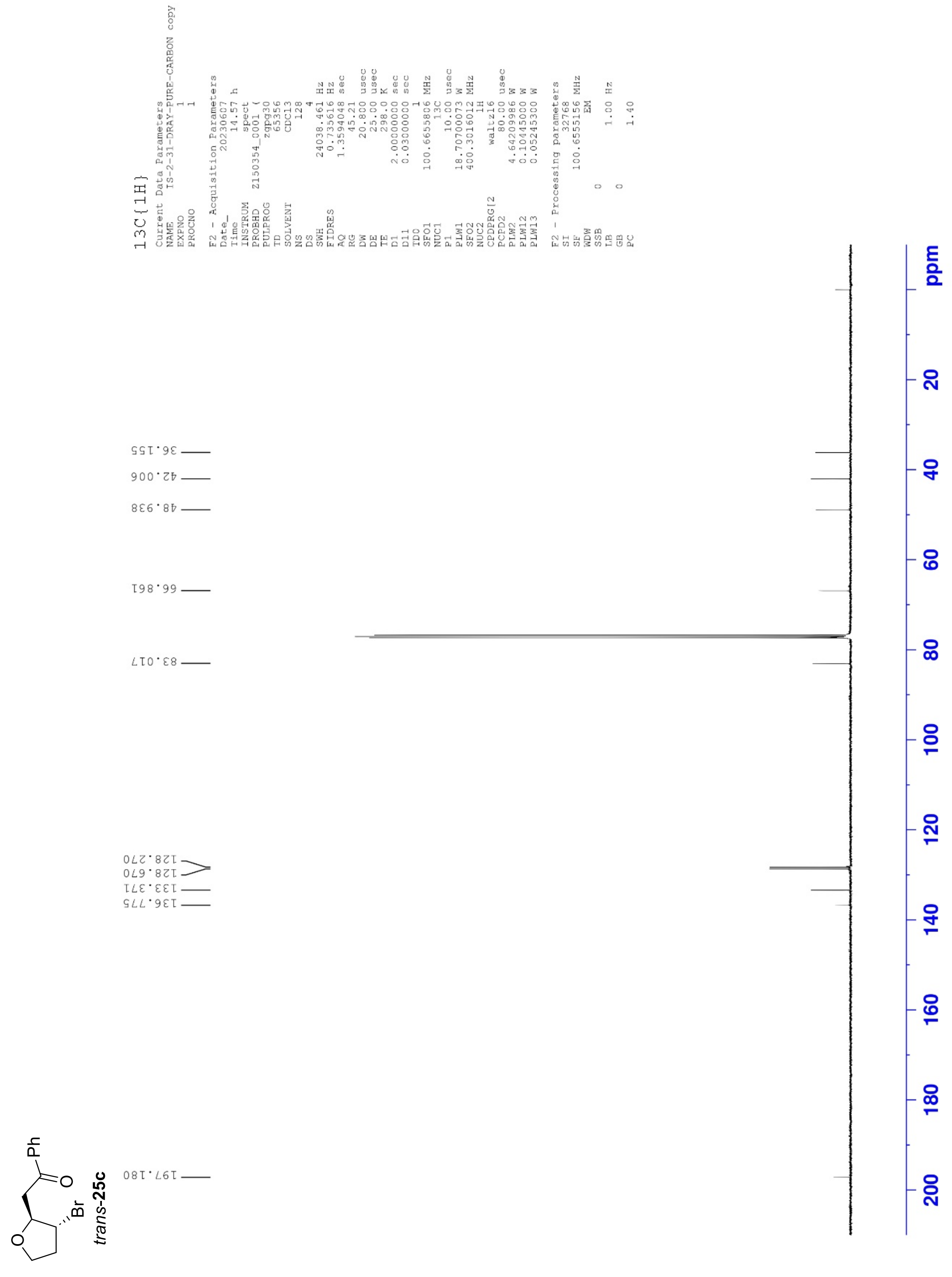
13C{1H}
Current Data Parameters
NAME IS-2-31-DRAY-PURE-CARBON copy
EXPNO 1
PROCNO 1
F2 - Acquisition Parameters
Date_ 20230607
Time 14.57 h
INSTRUM spect
PULPROG zgpg30
TD 65356
SOLVENT CDCl3
NS 128
DS 4
SWH 24038.461 Hz
FIDRES 0.735616 Hz
AQ 1.3594048 sec
RG 45.21
DW 20.800 usec
DE 25.00 usec
TE 298.0 K
D1 2.00000000 sec
D11 0.03000000 sec
TD0 1
SFO1 100.6655806 MHz
NUC1 13C
P1 10.00 usec
PLW1 18.70700073 W
SFO2 400.3016012 MHz
NUC2 1H
CPDPRG[2 waltz16
PCPD2 80.00 usec
PLW2 4.64209986 W
PLW12 0.10445000 W
PLW13 0.05245300 W
F2 - Processing parameters
SI 32768
SF 100.6555156 MHz
WDW EM
SSB 0
LB 1.00 Hz
GB 0
PC 1.40
36.155
42.006
48.938
66.861
83.017
128.270
128.670
133.371
136.775
197.180
Ph
O
Br
ppm
20
40
60
80
100
120
140
160
180
200
trans-**25c**

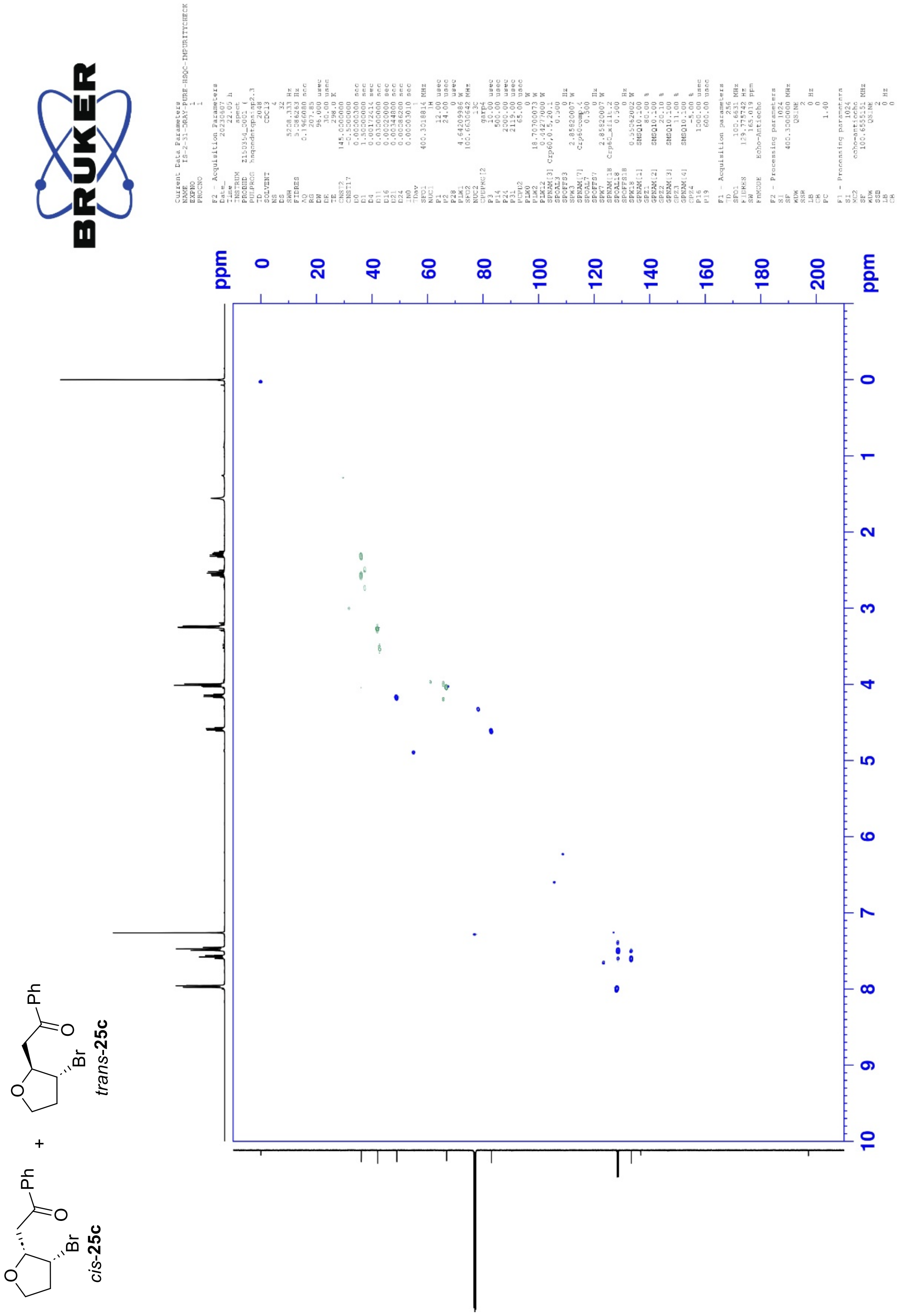

cis-**25c** + *trans*-**25c**

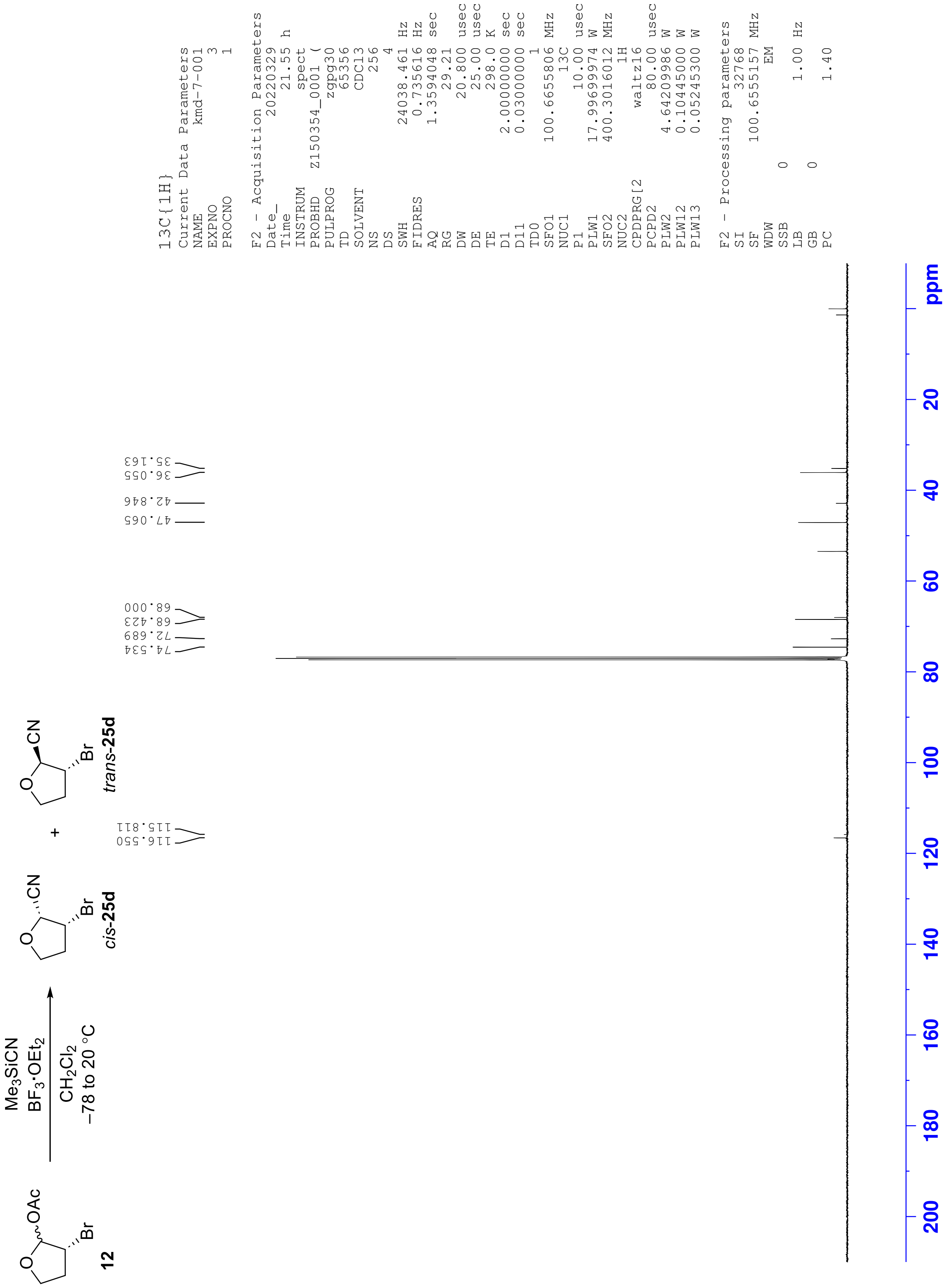
12
Me3SiCN
BF3·OEt2
CH2Cl2
−78 to 20 °C
cis-25d
trans-25d
OAc
Br
CN
O
+
116.550
115.811
74.534
72.689
68.423
68.000
47.065
42.846
36.055
35.163
13C{1H}
Current Data Parameters
NAME kmd-7-001
EXPNO 3
PROCNO 1
F2 - Acquisition Parameters
Date_ 20220329
Time_ 21.55 h
INSTRUM spect
PROBHD Z150354_0001 (
PULPROG zgpg30
TD 65356
SOLVENT CDCl3
NS 256
DS 4
SWH 24038.461 Hz
FIDRES 0.735616 Hz
AQ 1.3594048 sec
RG 29.21
DW 20.800 usec
DE 25.00 usec
TE 298.0 K
D1 2.00000000 sec
D11 0.03000000 sec
TD0 1
SFO1 100.6655806 MHz
NUC1 13C
P1 10.00 usec
PLW1 17.99699974 W
SFO2 400.3016012 MHz
NUC2 1H
CPDPRG[2 waltz16
PCPD2 80.00 usec
PLW2 4.64209986 W
PLW12 0.10445000 W
PLW13 0.05245300 W
F2 - Processing parameters
SI 32768
SF 100.6555157 MHz
WDW EM
SSB 0
LB 1.00 Hz
GB 0
PC 1.40
ppm
200
180
160
140
120
100
80
60
40
20

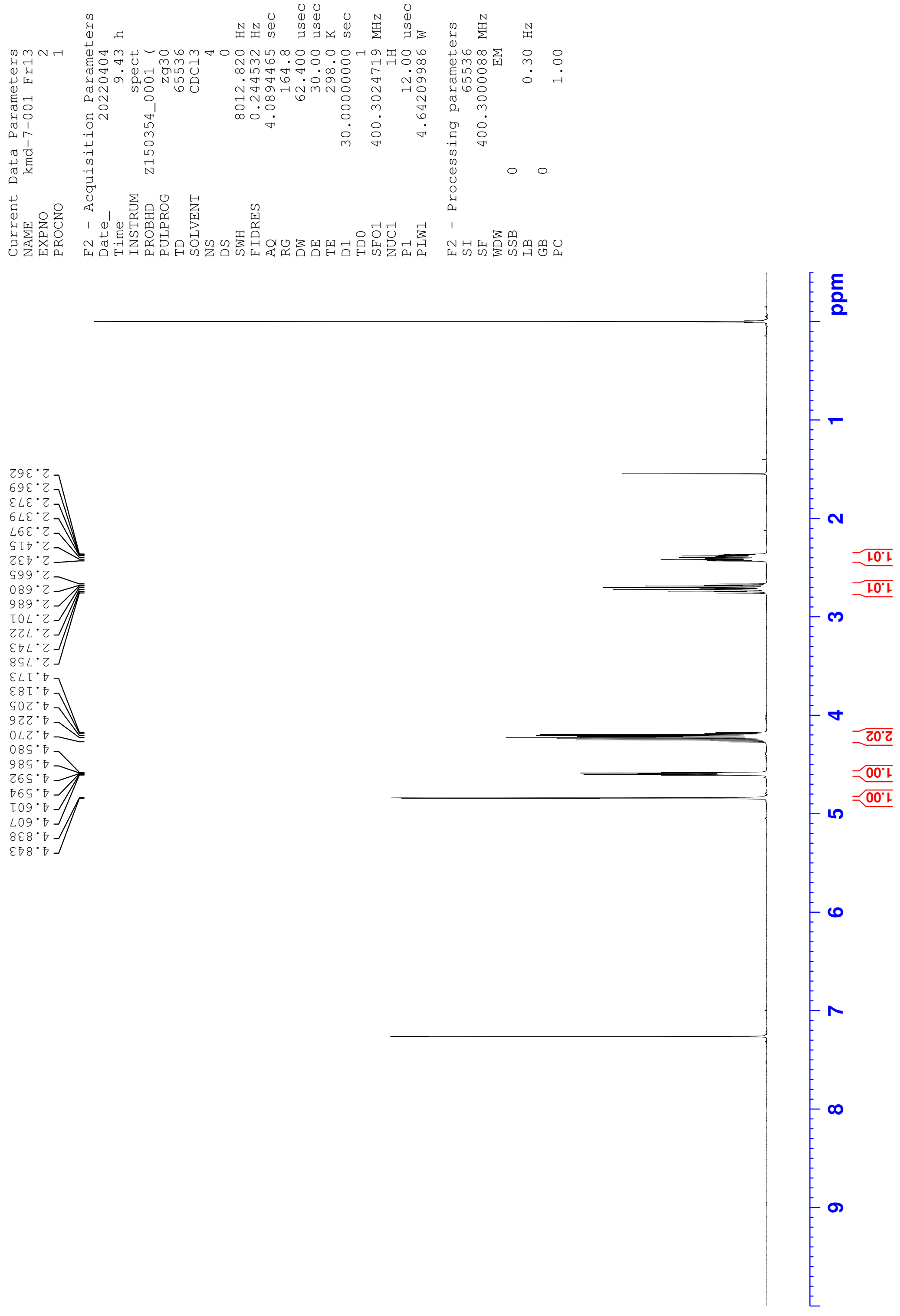
Current Data Parameters
NAME kmd-7-001 Fr13
EXPNO 2
PROCNO 1
F2 - Acquisition Parameters
Date_ 20220404
Time 9.43 h
INSTRUM spect
PROBHD Z150354_0001 (
PULPROG zg30
TD 65536
SOLVENT CDCl3
NS 4
DS 0
SWH 8012.820 Hz
FIDRES 0.244532 Hz
AQ 4.0894465 sec
RG 164.8
DW 62.400 usec
DE 30.00 usec
TE 298.0 K
D1 30.00000000 sec
TD0 1
SFO1 400.3024719 MHz
NUC1 1H
P1 12.00 usec
PLW1 4.64209986 W
F2 - Processing parameters
SI 65536
SF 400.3000088 MHz
WDW EM
SSB 0
LB 0.30 Hz
GB 0
PC 1.00
2.362
2.369
2.373
2.379
2.397
2.415
2.432
2.665
2.680
2.686
2.701
2.722
2.743
2.758
4.173
4.183
4.205
4.226
4.270
4.580
4.586
4.592
4.594
4.601
4.607
4.838
4.843
1.01
1.01
2.02
1.00
1.00
ppm
1
2
3
4
5
6
7
8
9

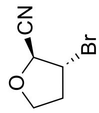
O
CN
Br

trans-**25d**

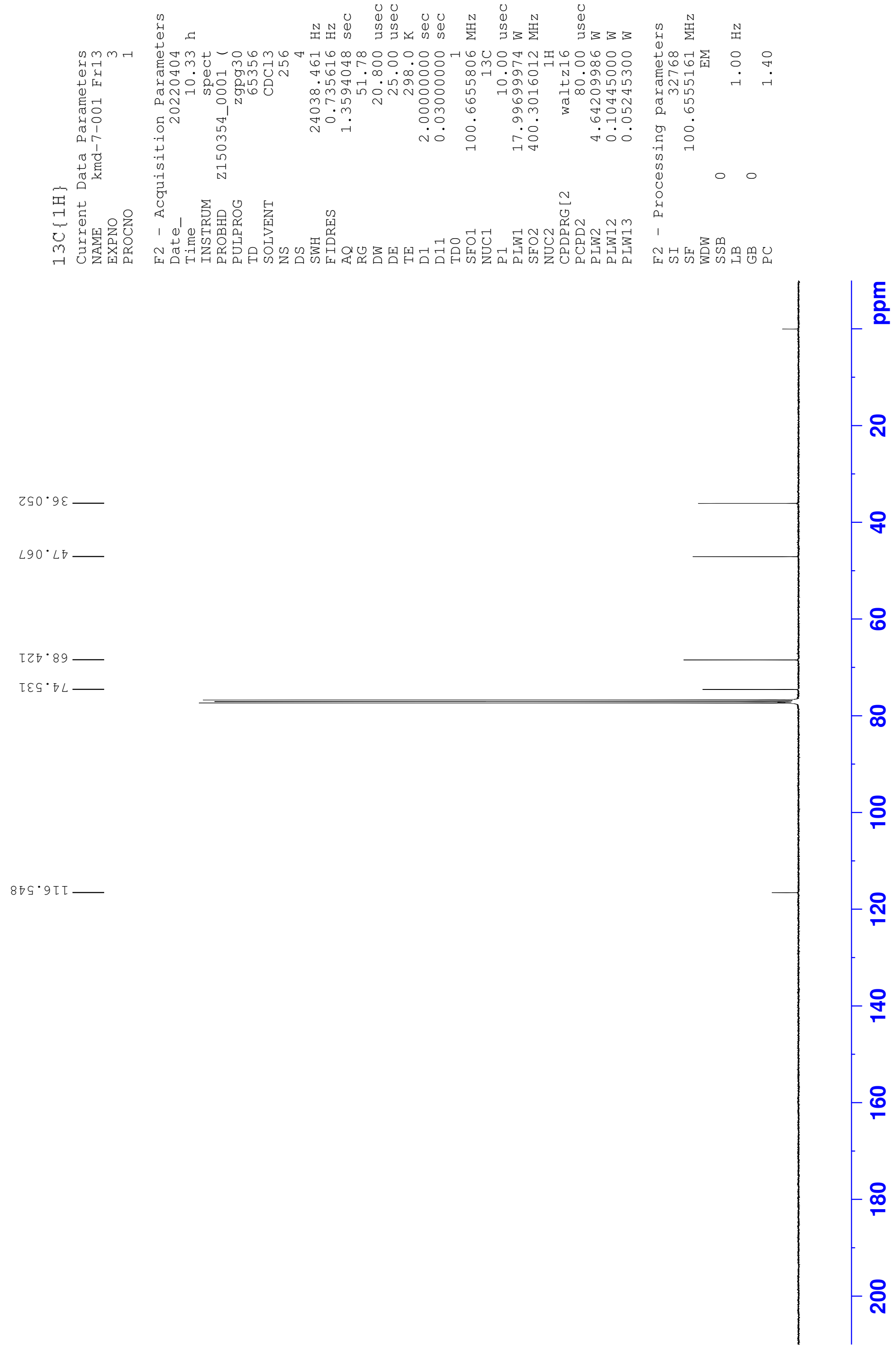
13C{1H}
Current Data Parameters
NAME kmd-7-001 Fr13
EXPNO 3
PROCNO 1
F2 - Acquisition Parameters
Date_ 20220404
Time_ 10.33 h
INSTRUM spect
PROBHD Z150354_0001 (
PULPROG zgpg30
TD 65356
SOLVENT CDCl3
NS 256
DS 4
SWH 24038.461 Hz
FIDRES 0.735616 Hz
AQ 1.3594048 sec
RG 51.78
DW 20.800 usec
DE 25.00 usec
TE 298.0 K
D1 2.00000000 sec
D11 0.03000000 sec
TD0 1
SFO1 100.6655806 MHz
NUC1 13C
P1 10.00 usec
PLW1 17.99699974 W
SFO2 400.3016012 MHz
NUC2 1H
CPDPRG[2 waltz16
PCPD2 80.00 usec
PLW2 4.64209986 W
PLW12 0.10445000 W
PLW13 0.05245300 W
F2 - Processing parameters
SI 32768
SF 100.6555161 MHz
WDW EM
SSB 0
LB 1.00 Hz
GB 0
PC 1.40
36.052
47.067
68.421
74.531
116.548
ppm
20
40
60
80
100
120
140
160
180
200

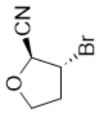
O
CN
Br

trans-**25d**

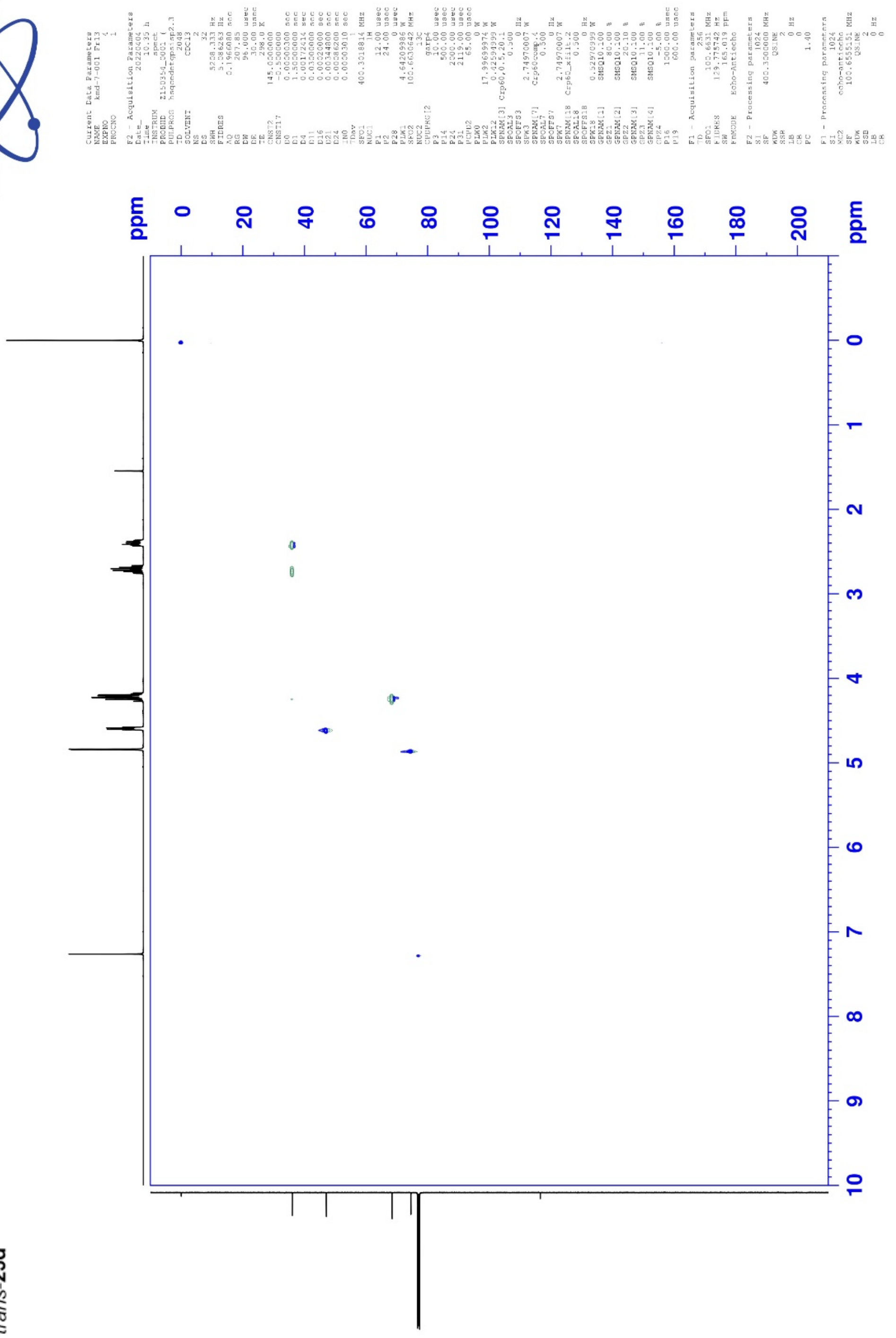

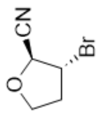

trans-**25d**

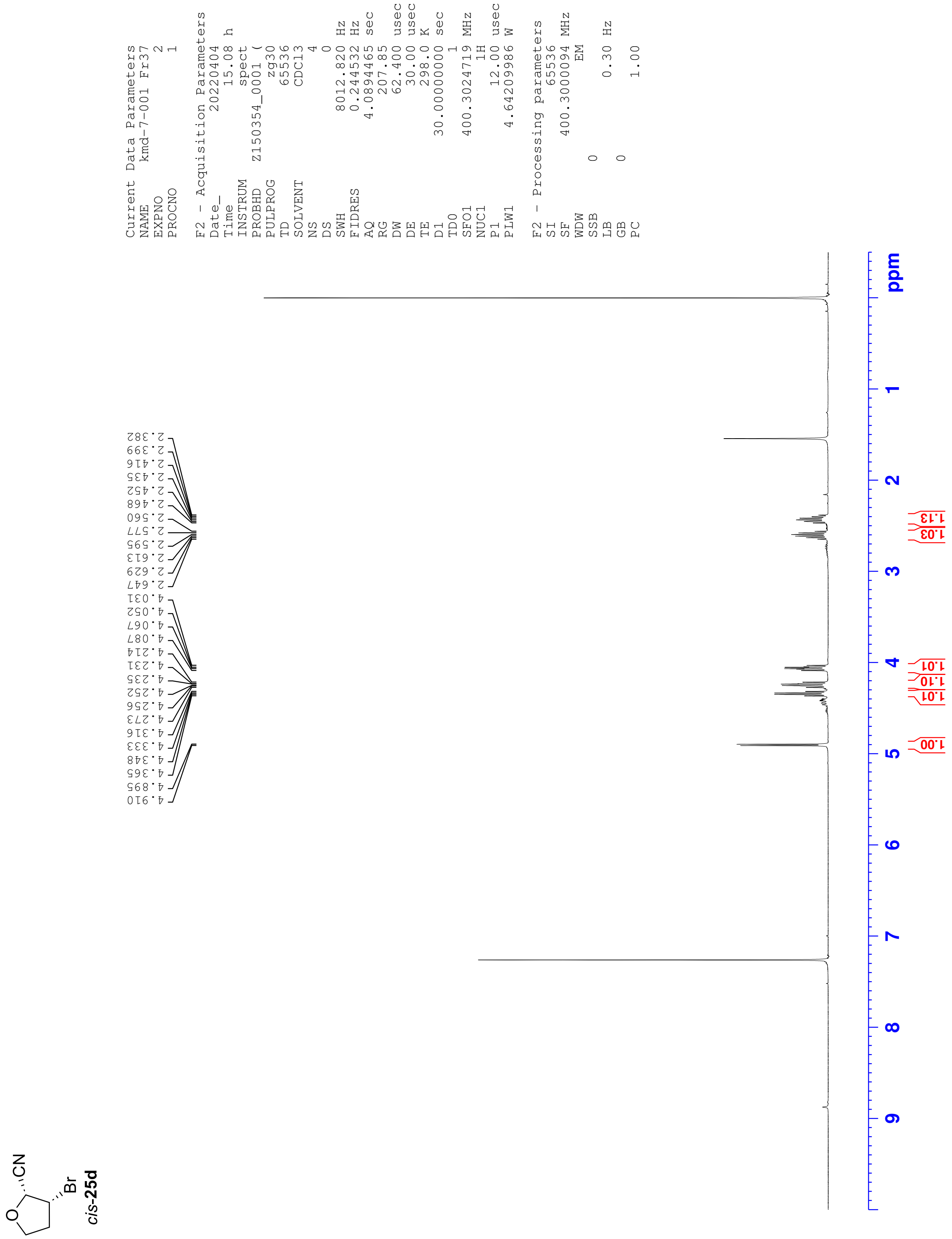
Current Data Parameters
NAME kmd-7-001 Fr37
EXPNO 2
PROCNO 1
F2 - Acquisition Parameters
Date_ 20220404
Time 15.08 h
INSTRUM spect
PROBHD Z150354_0001 (
PULPROG zg30
TD 65536
SOLVENT CDCl3
NS 4
DS 0
SWH 8012.820 Hz
FIDRES 0.244532 Hz
AQ 4.0894465 sec
RG 207.85
DW 62.400 usec
DE 30.00 usec
TE 298.0 K
D1 30.00000000 sec
TD0 1
SFO1 400.3024719 MHz
NUC1 1H
P1 12.00 usec
PLW1 4.64209986 W
F2 - Processing parameters
SI 65536
SF 400.3000094 MHz
WDW EM
SSB 0
LB 0.30 Hz
GB 0
PC 1.00
2.382
2.399
2.416
2.435
2.452
2.468
2.560
2.577
2.595
2.613
2.629
2.647
4.031
4.052
4.067
4.087
4.214
4.231
4.235
4.252
4.256
4.273
4.316
4.333
4.348
4.365
4.895
4.910
1.13
1.03
1.01
1.10
1.01
1.00
ppm
1
2
3
4
5
6
7
8
9
O
CN
Br
cis-**25d**

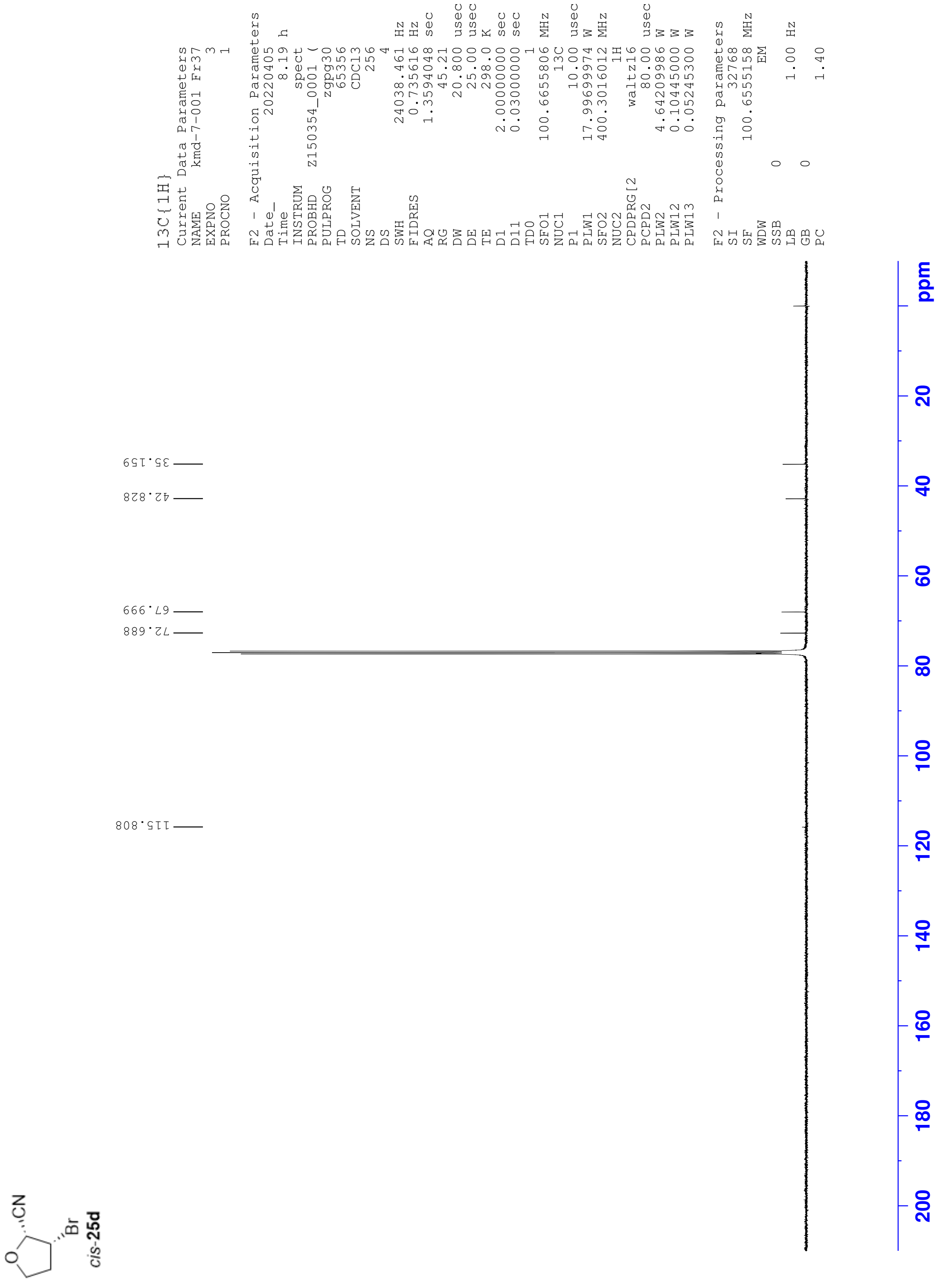
13C{1H}
Current Data Parameters
NAME kmd-7-001 Fr37
EXPNO 3
PROCNO 1
F2 - Acquisition Parameters
Date_ 20220405
Time_ 8.19 h
INSTRUM spect
PROBHD Z150354_0001 (
PULPROG zgpg30
TD 65356
SOLVENT CDCl3
NS 256
DS 4
SWH 24038.461 Hz
FIDRES 0.735616 Hz
AQ 1.3594048 sec
RG 45.21
DW 20.800 usec
DE 25.00 usec
TE 298.0 K
D1 2.00000000 sec
D11 0.03000000 sec
TD0 1
SFO1 100.6655806 MHz
NUC1 13C
P1 10.00 usec
PLW1 17.99699974 W
SFO2 400.3016012 MHz
NUC2 1H
CPDPRG[2 waltz16
PCPD2 80.00 usec
PLW2 4.64209986 W
PLW12 0.10445000 W
PLW13 0.05245300 W
F2 - Processing parameters
SI 32768
SF 100.6555158 MHz
WDW EM
SSB 0
LB 1.00 Hz
GB 0
PC 1.40
35.159
42.828
67.999
72.688
115.808
ppm
20
40
60
80
100
120
140
160
180
200
O
CN
Br
cis-25d

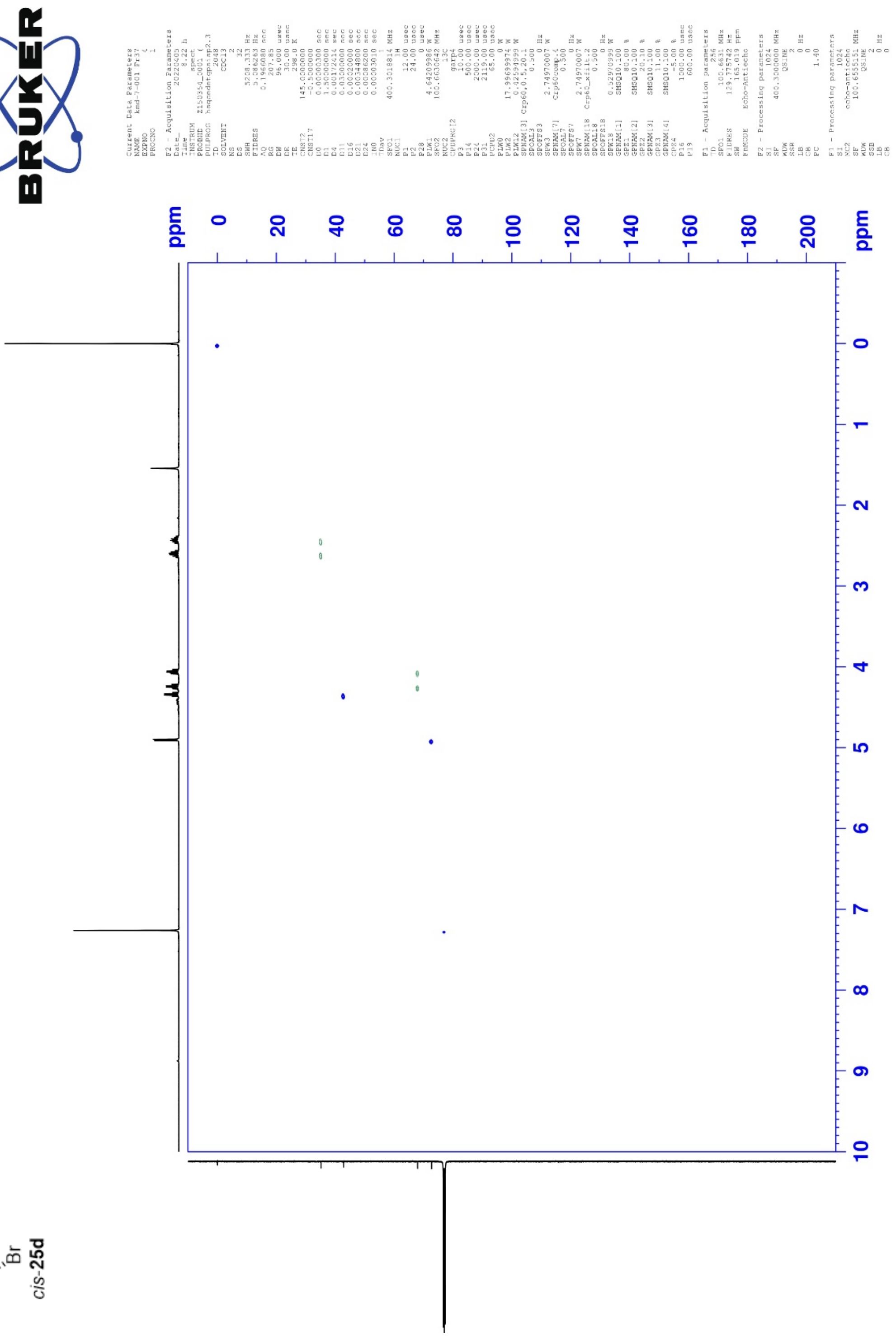

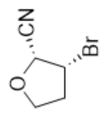

cis-**25d**

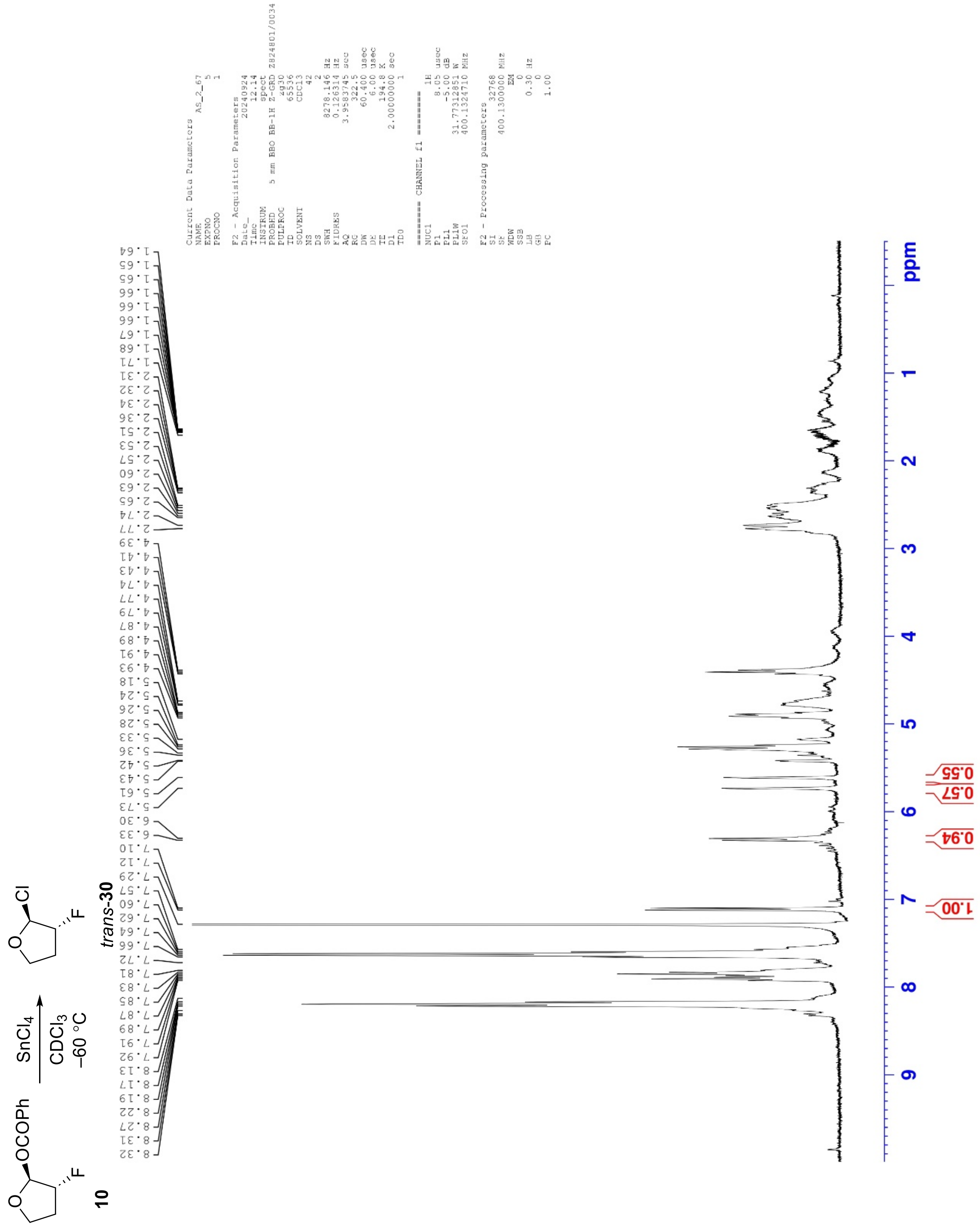
10
SnCl4
CDCl3
−60 °C
trans-30
ppm
